# Organo-catalyzed photoelectrochemical ring-contraction of arylidenecyclobutanols *via* radical cation-triggered semipinacol rearrangement

**DOI:** 10.1039/d5sc07637d

**Published:** 2025-11-28

**Authors:** Yu Zheng, Chunxi Chen, Xuhao Zhou, Guoyang Deng, Yanju Lu, Shenlin Huang

**Affiliations:** a State Key Laboratory for Development and Utilization of Forest Food Resources, Jiangsu Co-Innovation Center of Efficient Processing and Utilization of Forest Resources, Nanjing Forestry University Nanjing 210037 China zhengy@njfu.edu.cn shuang@njfu.edu.cn

## Abstract

The semipinacol rearrangement has proven to be an efficient strategy for converting allylic alcohols into carbonyl compounds bearing an α-quaternary carbon center. Traditionally, this semipinacol rearrangement is initiated through two primary modes, namely electrophile and radical-triggered pathways. Notably, these two strategies predominantly lead to ring-expansion β-functionalized products from cyclic allylic alcohols. In contrast, the radical cation, possessing carbon-centered radical and electrophilic carbocation dual reactivity, potentially triggers ring contraction semipinacol rearrangement of cyclic allylic alcohols, but remains underexplored. This is presumably due to the favorable C–C bond cleavage facilitated by ring-strain release and the challenge associated with overcoming the energy barriers required for ring-contraction. Herein, we report the first photoelectrochemical alkene radical cation-triggered semipinacol rearrangement for the ring-contraction of arylidenecyclobutanols. This methodology enables access to diverse and valuable 1,1-cyclopropane formylketones and diketones under mild and environmentally benign conditions. The resulting products not only contain the cyclopropane motif, which is present in many pharmaceuticals and bioactive molecules, but also serve as useful synthetic intermediates for the preparation of various derivatives, including the key intermediate of cabozantinib.

## Abstract 2

An organo-catalyzed photoelectrochemical alkene radical cation-triggered semipinacol rearrangement for the ring-contraction of arylidenecyclobutanols was developed, which enables access to diverse cyclopropanes under mild conditions.

## Graphical abstract

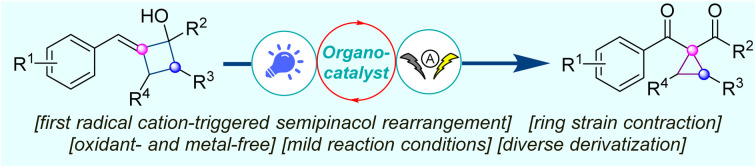

## Introduction

**Fig. 1:**
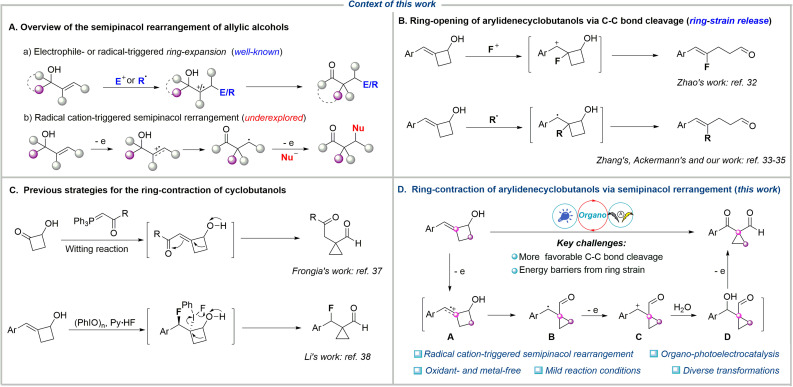
Context of this work. (A) Overview of the semipinacol rearrangement of allylic alcohols. (B) Ring-opening of arylidenecyclobutanols *via* C–C bond cleavage. (C) Previous strategies for the ring-contraction of cyclobutanols. (D) Ring-contraction of arylidenecyclobutanols *via* semipinacol rearrangement (this work).

Rearrangement reactions represent powerful tools for skeletal remodeling. These reactions fundamentally alter the framework by cleaving existing bonds and reorganizing atoms or groups within the molecule. Crucially, rearrangement reactions enable the efficient construction of complex and often difficult-to-access carbon skeletons directly from simple precursors, bypassing multi-step synthetic routes. This capability is particularly valuable in the synthesis of intricate natural products and pharmaceuticals.^1–4^ Among various rearrangement reactions, the semipinacol rearrangement has proven to be an efficient strategy for the transformation of allylic alcohols to carbonyl compounds with an α-quaternary carbon center.^5–7^ Traditionally, the electrophilic attack toward the C=C bond of allylic alcohol generates an electrophilic carbocation, which triggers the subsequent rearrangement, delivering the corresponding carbonyl compounds (Fig. 1A).^8–18^ An alternative strategy involves the radical addition of the C=C bond to form a carbon-centered radical, which is further oxidized to a carbocation intermediate (radical-polar crossover process), thereby triggering semipinacol rearrangement. This strategy has recently emerged as an efficient platform for the reaction of various radical precursors with allylic alcohols to access diverse β-functionalized ketones *via* photocatalysis^19–23^ or electrocatalysis,^24–27^ among others.^28–31^ Furthermore, both electrophilic and radical functionalization/semipinacol rearrangement sequences typically yield ring-expansion β-functionalized products from cyclic allylic alcohols. This is because the β-C−C bond cleavage of cyclic allylic alcohols is more favored than other transformations due to the release of ring strain. For example, the electrophilic fluorination of arylidenecyclobutanols generated a carbocation intermediate, which tended to undergo C–C bond cleavage, thereby delivering the ring-opening products disclosed by Zhao's group in 2017 (Fig. 1B).^32^ More recently, the Ackermann,^33^ Zhang,^34^ and our groups^35^ independently reported that the photocatalytic radical addition of arylidenecyclobutanols led to the same C–C bond cleavage driven by strain release, resulting in the ring-opening products. On the other hand, given the strain energies of 26.3 kcal mol^−1^ for cyclobutanes *versus* 29.0 kcal mol^−1^ for cyclopropanes,^36^ the ring-contraction of cyclobutanols to access cyclopropanes should be thermodynamically challenging. In 2019, Frongia *et al.* reported the synthesis of cyclopropanecarbaldehydes through a tandem Wittig reaction and ring-contraction process at 80 °C over 2 days (Fig. 1C).^37^ Separately, Li and coworkers accomplished a fluorination/semipinacol rearrangement cascade for the ring-contraction of 2-alkylidenecyclobutanol, using a combination of nucleophilic Py·HF and (PhIO)_*n*_.^38^ Furthermore, alkoxy radical mediated β-C−C bond cleavage of cyclobutanols generates carbon-centered radicals capable of participating in diverse transformations.^39–47^ This competing reaction pathway thereby jeopardizes the desired ring-contraction process. To the best of our knowledge, the radical cation, which features carbon-centered radical and electrophilic carbocation dual reactivity and directly triggers the ring-contraction semipinacol rearrangement of cyclic allylic alcohols, remains underexplored.

Photoelectrochemistry leverages the synergistic integration of light and electrical energy to achieve challenging reactivity and selectivity under mild and environmentally benign conditions.^48–55^ This approach significantly broadens the accessible redox window for reaction design while eliminating dependence on chemical redox reagents, establishing a new paradigm for green and precision organic synthesis. Based on recent achievements in alkene radical cation chemistry^56–62^ and combined with our continuous interest in electrochemical synthesis of cyclopropanes,^63^ we envisioned that chemoselective oxidation of the C=C bonds in arylidenecyclobutanols could generate the corresponding alkene radical cation A, which possesses carbon-centered radical and electrophilic carbocation dual reactivity, and thereby might trigger the semipinacol rearrangement to give the ring-contraction intermediate B under suitable conditions (Fig. 1D). Subsequent oxidation of intermediate B followed by nucleophilic addition results in the final cyclopropanes, which are important motifs present in many pharmaceuticals and bioactive molecules.^64–70^ Herein, we report an organo-catalyzed photoelectrochemical ring-contraction of cyclobutanols *via* alkene radical cation triggered-semipinacol rearrangement to access diverse and valuable 1,1-cyclopropane formylketones and diketones with an α-quaternary carbon center under environmentally friendly and mild reaction conditions without the requirement of an oxidant and metal-catalyst.

## Results and discussion

The feasibility of our proposal was investigated by employing 2-benzylidenecyclobutanol 1a as the model substrate under photoelectrochemical conditions (Table 1). After systematically optimizing different reaction parameters, the desired ring-contraction product 1-benzoyl-1-formylcyclopropane 2a was finally obtained in 72% isolated yield under the following optimal conditions: the photoelectrolysis of 1a was performed in the presence of 5 mol% 9-mesityl-10-methyl acridinium perchlorate ([Mes-Acr^+^]ClO_4_^−^) as the catalyst, ^*n*^Bu_4_NPF_6_ as an electrolyte, Pt plates as the electrode materials in a 5 : 1.2 CH_3_CN/H_2_O mixed solvent at a constant current of 5 mA and 40 W blue light irradiation for 6 hours (Table 1, entry 1). The solvent effect had a decisive influence, as no desired product was detected when using other solvents such as dimethylformamide (DMF) or tetrahydrofuran (THF) instead of CH_3_CN (Table 1, entry 2, for more details see SI, Table S1). The electrode materials had a great impact on the reaction efficiency. Switching the cathode material from Pt(−) to Sn(−) or C(−) afforded a decreased yield, whereas on using GF(+) or C(+) as the anode, only a trace amount of the product was observed (Table 1, entries 3 and 4). Among the tested catalysts, including organic dyes, metal photocatalysts, and modifications of [Mes-Acr^+^]ClO_4_^−^, no better results were obtained (Table 1, entries 5 and 6, for more details see SI, Table S1). Performing the photoelectrochemical reaction with 15 W and 35 W blue LEDs resulted in a diminished yield in both cases (Table 1, entry 7). Slight variations in decreasing or increasing the amount of H_2_O had a deleterious influence on the outcome (Table 1, entry 8). The replacement of ^*n*^Bu_4_NPF_6_ with other electrolytes such as Et_4_NPF_6_ or NH_4_PF_6_ led to reduced yields (Table 1, entry 9, for more details see SI, Table S1). In addition, either decreasing or increasing the constant current provided a lower yield (Table 1, entry 10). Furthermore, the addition of trifluoroacetic acid (TFA) or base (K_2_CO_3_) led to no improvement in the yield (Table 1, entry 11). Control experiments indicated that without light or a catalyst, the yield dropped to 31% or 34%, respectively (Table 1, entries 12 and 13). In addition, electricity is crucial to the reaction since the yield was significantly decreased to 12% without the constant current (Table 1, entry 14). These results indicated that electricity, light as well as the catalyst together guarantee the reaction efficiency. Furthermore, the observation of α,β-unsaturated cyclobutanone and 1-benzoylcyclopropane-1-carboxylic acid as the main byproducts under optimal conditions accounted for the relatively low yield of 2a (see the SI for more details).

**Table 1:** Optimization of the reaction conditions

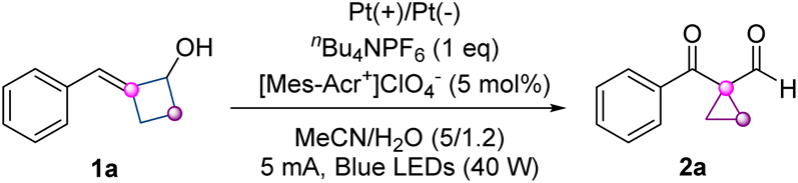		
Entry	Variation from standard conditions	Yield^a^ of 2a [%]
1	None	59 (72)^b^
2	THF or DMF instead of CH_3_CN	n.d.^c^
3	C(−) or Sn(−) instead of Pt(−)	35, 32
4	C(+) or GF(+) instead of Pt(+)	Trace, trace
5	4CzIPN instead of [Mes-Acr^+^]ClO_4_^−^	48
6	Ir(dF(CF_3_)ppy)_2_(dtbbpy)]PF_6_ instead	52
7	15 W or 35 W instead of 40 W	41, 54
8	1.0 mL or 1.5 mL H_2_O instead	34, 50
9	Et_4_NPF_6_ or NH_4_PF_6_ instead	42, 58
10	3 mA or 8 mA instead of 5 mA	54, 37
11	TFA or K_2_CO_3_ as additives	49, 35
12	No light	31
13	No catalyst	34
14	No electricity	12

a GC yields with 1-nitronaphthalene as the internal standard.

b Isolated yield.

c n.d. = No desired product was detected.

**Fig. 2:**
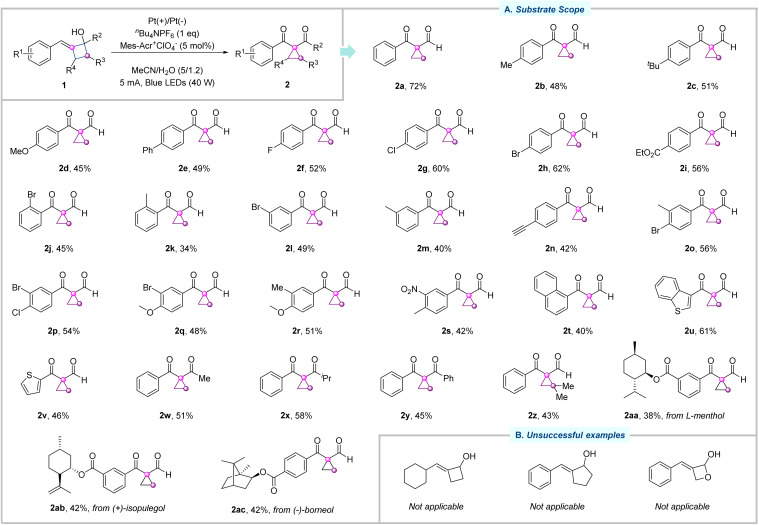
Substrate scope investigations. (A) Substrate scope. (B) Unsuccessful examples.

With the optimal conditions in hand, we moved our attention to the substrate scope of this photoelectrochemical ring-contraction reaction, as illustrated in Fig. 2. First, we investigated the modifications of the substituents at the *para*-position of the aryl ring of arylidenecyclobutanols. In general, both electron-donating (–Me, –^*t*^Bu, –OMe, and –Ph) and electron-withdrawing groups (–F, –Cl, –Br, and –CO_2_Et) all tolerated very well, delivering the corresponding cyclopropanes 2b–2i in good yields. Subsequently, the compatibility of functional groups at the *meta* and *ortho* positions of the aryl ring was evaluated. Both electron-donating (–Me) and electron-withdrawing (–Br) substituents reacted smoothly, affording the desired cyclopropanes 2j–2m in moderate to good yields. In addition, sensitive functional group alkyne was well tolerated (2n). Diverse di-substituted derivatives were applicable to this catalytic reaction, and the desired products (2o–2s) were isolated in moderate to good yields. Arylidenecyclobutanols bearing a naphthyl ring, benzothiophene, and thiophene were competent substrates, giving rise to the desired cyclopropanes 2t–2v. Apart from the above secondary alcohols, tertiary alcohol substrates were subsequently accessed under otherwise identical conditions. Methyl- and isopropyl-substituted arylidenecyclobutanols 1w and 1x were smoothly transformed into 2w and 2x in 51% and 58% yields, respectively. Moreover, phenyl-substituted arylidenecyclobutanol 1y exhibited good reactivity in this photoelectrochemical ring-contraction reaction as well. Arylidenecyclobutanol with a dimethyl group on the four-member ring was suitable under the standard conditions, delivering the cyclopropane derivatives in 43% yield (2z). Notably, the photoelectrochemical conditions allowed us to explore substrates derived from more complex molecules, accessing the target cyclopropanes with L-menthol, (+)-isopulegol, and (−)-borneol (2aa–2ac). It is noteworthy that the relatively low yields were mainly attributable to the formation of oxidation byproducts that were also observed in the model reaction. Meanwhile, the reaction suffers from some limitations. The cyclohexyl-substituted cyclobutanol substrate was not well-tolerated, likely due to the instability of the alkyl radical intermediate. Benzylidenecyclopentanol was unsuccessful under the standard conditions. When the substrate containing an oxetane motif was applied to the reaction, the corresponding product was not observed, with the starting materials being completely consumed, indicating that the oxygen atom migration failed under current conditions.

**Fig. 3:**
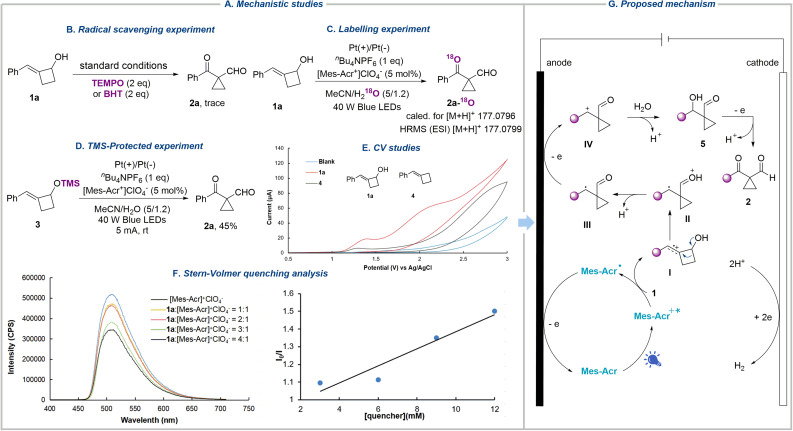
Mechanistic studies and proposal. (A) Mechanistic studies. (B) Radical scavenging experiment. (C) Labelling experiment. (D) TMS-protected experiment. (E) CV studies. (F) Stern–Volmer quenching analysis. (G) Proposed mechanism.

To obtain experimental evidence of the reaction mechanism, we conducted a series of experiments (Fig. 3A). The addition of radical scavengers, including 2,2,6,6-tetramethyl-1-piperidinyloxy (TEMPO) and butylated hydroxytoluene (BHT), both sharply suppressed the formation of the desired product (Fig. 3B). On conducting the reaction with H_2_^18^O instead of H_2_O under standard conditions, the heavy-oxygen-labelled product 2a-^18^O was formed, which was confirmed by high-resolution mass spectrometry (HRMS), suggesting that oxygen comes from water and water serves as the nucleophile in the reaction (Fig. 3C). Since substrate 1a contains a hydroxyl group, which could generate the alkoxy radical under oxidation conditions. This reactive radical can undergo β-scission, radical addition, oxidation, and nucleophilic attack sequences, eventually affording the same product. To rule out this possibility, trimethylsilyl alkenylcyclobutanol 3 was subjected to the reaction, and product 2a was isolated in 45% yield, indicating that the alkoxy radical was not involved in the reaction (Fig. 3D). In addition, cyclic voltammetry was employed to identify the oxidation sequence of the alkene and hydroxyl group. Substrate 1a shows two oxidation peaks at *E* = 1.36 and 2.02 V *vs.* Ag/AgCl, whereas benzylidenecyclobutane 4 presents an oxidation peak at 1.29 V *vs.* Ag/AgCl, demonstrating the preferential oxidation of the alkene bond of 1 (Fig. 3E). Furthermore, the luminescence quenching experiments were performed with 1a and [Mes-Acr^+^]ClO_4_^−^ (Fig. 3F). The results showed that the excited state of [Mes-Acr^+^]ClO_4_^−^ was quenched by 1a, signifying that 1a was oxidized by the excited photocatalyst (see the SI for more details).

Based on the above data and previous work,^71–73^ we propose the mechanism of this photoelectrochemically driven semipinacol-type rearrangement reaction as shown in Fig. 3G. Initially, the excited-state organic dye photocatalyst Mes-Acr^+^* (*E*^red^ = 2.06 V *vs.* SCE in MeCN)^74^ could sufficiently oxidize the double bond of 1 to generate the radical cation I and the acridinyl radical Mes-Acr˙, which is oxidized at the anode to regenerate the ground-state organocatalyst Mes-Acr^+^. The radical cation I, featuring carbon-centered radical and electrophilic carbocation dual reactivity, undergoes semipinacol-type rearrangement to give the oxonium ion intermediate II, which then transforms into the carbon-centered radical III after the loss of a proton. Subsequently, radical III is further oxidized to give the cation intermediate IV, which is attacked by H_2_O acting as a nucleophile, resulting in the formation of 5 followed by deprotonation. Compound 5 was observed during the reaction, which disappeared after the completion of the reaction (see the SI for more details). Finally, the third anodic oxidation delivers cyclopropanes 2.

**Fig. 4:**
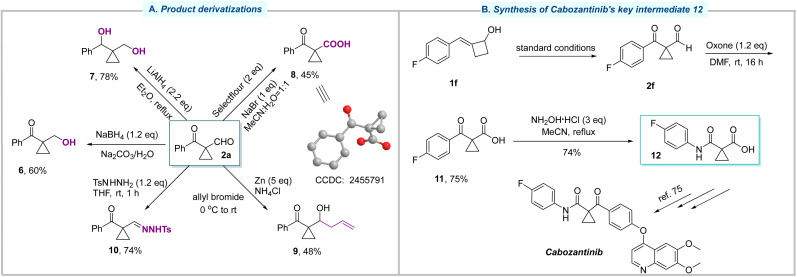
Synthetic applications. (A) Product derivatizations. (B) Synthesis of cabozantinib's key intermediate 12.

As illustrated in Fig. 4A, further transformations of these 1,1-disubstituted cyclopropanes were investigated to highlight their synthetic utility. First, the formyl group in 2a could be chemoselectively reduced by NaBH_4_ to give 6 in 60% yield. Using a stronger reductant, LiAlH_4_, the carbonyl group was also reduced, leading to the formation of diol 7 in 78% yield. On the other hand, the oxidation of 2a proceeded smoothly and afforded the acid 8, whose structure was determined by X-ray crystallographic analysis. The reaction of compound 2a with allyl bromide in the presence of Zn successfully gave rise to allylic alcohol 9. Moreover, the condensation reaction of 2a between TsNHNH_2_ yielded the corresponding tosylhydrazone 10 in 74% yield. Finally, the oxidation of 2f with Oxone afforded compound 11 in 75% yield (Fig. 4B). The subsequent Beckmann rearrangement successfully delivered compound 12, a key intermediate toward the synthesis of cabozantinib.^75^ This alternative synthetic route demonstrated the practicality of this methodology, featuring an advantage that eliminates the use of corrosive and toxic SOCl_2_.

## Conclusions

In conclusion, we have developed the photoelectrochemical ring-contraction of arylidenecyclobutanols for the first time. This strategy provides a mild and environmentally benign route to a wide range of valuable 1,1-cyclopropane formylketones and diketones bearing an α-quaternary carbon center. Mechanistic investigations indicated that the alkene radical cation-triggered semipinacol rearrangements, deprotonation, oxidation, and nucleophilic attack sequences enable the desired cyclopropanes. The obtained products could serve as versatile synthetic intermediates, as demonstrated by their application in synthesizing a key intermediate of cabozantinib.

## Author contributions

Y. Z. and S. H. contributed to the conception and design of the experiments. Y. Z. directed the project and analyzed the data. C. C., X. Z., and G. D. performed the experiments and analyzed the data. Y. Z. wrote the manuscript. Y. L. and S. H. provided valuable suggestions to the project. All authors discussed the results and commented on the manuscript.

## Conflicts of interest

There are no conflicts to declare.

## Supplementary Material

SC-017-D5SC07637D-s001

SC-017-D5SC07637D-s002

## Data Availability

CCDC 2455791 (8) contains the supplementary crystallographic data for this paper.^76^ The data supporting this article have been included as part of the supplementary information (SI). Supplementary information: experimental procedures and characterization data. See DOI: https://doi.org/10.1039/d5sc07637d.

## References

[cit1] Ilardi E. A., Stivala C. E., Zakarian A. (2009). Chem. Soc. Rev..

[cit2] Fernandes R. A., Kattanguru P., Gholapa S. P., Chaudharia D. A. (2017). Org. Biomol. Chem..

[cit3] Liu X.-L., Gong M.-L., Yang X., Tian P., Li Q.-H. (2024). Org. Chem. Front..

[cit4] Chen L., Li G., Zu L. (2022). Org. Chem. Front..

[cit5] Nanda S. K. (2023). Adv. Synth. Catal..

[cit6] Snape T. J. (2007). Chem. Soc. Rev..

[cit7] Song Z.-L., Fan C.-A., Tu Y.-Q. (2011). Chem. Rev..

[cit8] Xing Y., Li C., Meng J. P., Zhang Z., Wang X., Wang Z. C., Ye Y., Sun K. (2021). Adv. Synth. Catal..

[cit9] Zhang X.-M., Li B.-S., Wang S.-H., Zhang K., Zhang F.-M., Tu Y.-Q. (2021). Chem. Sci..

[cit10] Lindner H., Carreira E. M. (2024). Angew. Chem., Int. Ed..

[cit11] Zhao P., Wang W., Gulder T. (2023). Org. Lett..

[cit12] Zhang D.-Y., Zhang Y., Wu H., Gong L.-Z. (2019). Angew. Chem., Int. Ed..

[cit13] Kalomenopoulos P. G., Emayavaramban B., Johnston C. P. (2025). Angew. Chem., Int. Ed..

[cit14] Blackburn M. A. S., Wagen C. C., Bodrogean M. R., Tadross P. M., Bendelsmith A. J., Kutateladze D. A., Jacobsen E. N. (2023). J. Am. Chem. Soc..

[cit15] Yu X., Zheng C., You S.-L. (2024). J. Am. Chem. Soc..

[cit16] Zhao S., Yue W., Yang M., Li X., Chen B., Gao Y., Yu W., Ni H.-L., Hu P., Wang B.-Q., Cao P. (2024). Org. Lett..

[cit17] Zheng T., Chen R., Huang J., Gonçalves T. P., Huang K.-W., Yeung Y.-Y. (2023). Chem.

[cit18] Capel E., Rodríguez-Rodríguez M., Uria U., Pedron M., Tejero T., Vicario J. L., Merino P. (2022). J. Org. Chem..

[cit19] Yao S., Zhang K., Zhou Q.-Q., Zhao Y., Shi D.-Q., Xiao W.-J. (2018). Chem. Commun..

[cit20] Giri R., Patra S., Katayev D. (2023). ChemCatChem.

[cit21] Liu M., Huang H., Chen Y. (2018). Chin. J. Chem..

[cit22] Ma T.-C., Yao S., Qiao M.-M., Yuan F., Shi D.-Q., Xiao W.-J. (2021). Org. Chem. Front..

[cit23] Kodo T., Nagao K., Ohmiya H. (2022). Nat. Commun..

[cit24] Kwon S. J., Kim D. Y. (2016). Org. Lett..

[cit25] Jung H. I., Kim Y., Kim D. Y. (2019). Org. Biomol. Chem..

[cit26] Kang J.-C., Tu Y.-Q., Dong J.-W., Chen C., Zhou J., Ding T.-M., Zai J.-T., Chen Z.-M., Zhang S.-Y. (2019). Org. Lett..

[cit27] Kim Y. J., Kim D. Y. (2019). Org. Lett..

[cit28] Xie Y.-Y., Chen Z.-M., Luo H.-Y., Shao H., Tu Y.-Q., Bao X., Cao R.-F., Zhang S.-Y., Tian J.-M. (2019). Angew. Chem., Int. Ed..

[cit29] Kayal S., Kikuchi J., Shimizu M., Terada M. (2019). ACS Catal..

[cit30] Sahoo B., Li J.-L., Glorius F. (2015). Angew. Chem., Int. Ed..

[cit31] Smyrnov V., Waser J. (2023). Org. Lett..

[cit32] Liu T.-L., Wu J.-E., Zhao Y. (2017). Chem. Sci..

[cit33] Zhang Y., Zhao C., Ma C., Cai Z., Trienes S., Ackermann L. (2023). Angew. Chem., Int. Ed..

[cit34] Zhao C., Ma W., Liu K., Xu R., Ma X., Zhang Y. (2024). Org. Chem. Front..

[cit35] He T., Liang C., Jiang P., Liang H., Liao S., Huang S. (2024). Org. Lett..

[cit36] Khoury P. R., Goddard J. D., Tam W. (2004). Tetrahedron.

[cit37] Cuccu F., Serusi L., Luridiana A., Secci F., Caboni P., Aitken D. J., Frongia A. (2019). Org. Lett..

[cit38] Feng S.-X., Yang S., Tu F.-H., Lin P.-P., Huang L.-L., Wang H., Huang Z.-S., Li Q. (2021). J. Org. Chem..

[cit39] Wu X., Zhang H., Tang N., Wu Z., Wang D., Ji M., Xu Y., Wang M., Zhu C. (2018). Nat. Commun..

[cit40] Wang D., Mao J., Zhu C. (2018). Chem. Sci..

[cit41] Allen B. D. W., Hareram M. D., Seastram A. C., McBride T., Wirth T., Browne D. L., Morrill L. C. (2019). Org. Lett..

[cit42] Zhang K., Chang L., An Q., Wang X., Zuo Z. (2019). J. Am. Chem. Soc..

[cit43] Zhao K., Yamashita K., Carpenter J. E., Sherwood T. C., Ewing W. R., Cheng P. T. W., Knowles R. R. (2019). J. Am. Chem. Soc..

[cit44] Hareram M. D., EI Gehani A. A. M. A., Harnedy J., Seastram A. C., Jones A. C., Burns M., Wirth T., Browne D. L., Morrill L. C. (2022). Org. Lett..

[cit45] Ju M., Lee S., Marvich H. M., Lin S. (2024). J. Am. Chem. Soc..

[cit46] Yang Z., Yang D., Zhang J., Tan C., Li J., Wang S., Zhang H., Huang Z., Lei A. (2022). J. Am. Chem. Soc..

[cit47] Zhao L., Hu P., Tian J., Zhang X., Yang C., Guo L., Xia W. (2024). Org. Lett..

[cit48] Capaldo L., Quadri L. L., Ravelli D. (2019). Angew. Chem., Int. Ed..

[cit49] Barham J. P., Konig B. (2020). Angew. Chem., Int. Ed..

[cit50] Wu S., Kaur J., Karl T. A., Tian X., Barham J. P. (2022). Angew. Chem., Int. Ed..

[cit51] Huang H., Steiniger K. A., Lambert T. H. (2022). J. Am. Chem. Soc..

[cit52] Qian L., Shi M. (2023). Chem. Commun..

[cit53] Lamb M. C., Steiniger K. A., Trigoura L. K., Wu J., Kundu G., Huang H., Lambert T. H. (2024). Chem. Rev..

[cit54] Li L., Yao Y., Fu N. (2024). Chem Catal..

[cit55] Xiong P., Xu H.-C. (2025). Acc. Chem. Res..

[cit56] Luo M.-J., Xiao Q., Li J.-H. (2022). Chem. Soc. Rev..

[cit57] Chen N., Xu H.-C. (2021). Chem. Rec..

[cit58] Xiong P., Long H., Song J., Wang Y., Li J.-F., Xu H.-C. (2018). J. Am. Chem. Soc..

[cit59] Huang H., Lamber T. H. (2021). J. Am. Chem. Soc..

[cit60] Rybicka-Jasińska K., Szeptuch Z., Kubiszewski H., Kowaluk A. (2023). Org. Lett..

[cit61] Yi H., Niu L., Song C., Li Y., Dou B., Singh A. K., Lei A. (2017). Angew. Chem., Int. Ed..

[cit62] Zhang G., Hu X., Chiang C.-W., Yi H., Pei P., Singh A. K., Lei A. (2016). J. Am. Chem. Soc..

[cit63] Yi W., Xu P.-C., He T., Shi S., Huang S. (2024). Nat. Commun..

[cit64] Wessjohann L. A., Brandt W., Thiemann T. (2003). Chem. Rev..

[cit65] Chen D. Y.-K., Pouwer R. H., Richard J.-A. (2012). Chem. Soc. Rev..

[cit66] Talele T. T. (2016). J. Med. Chem..

[cit67] Fan Y.-Y., Gao X.-H., Yue J.-M. (2016). Sci. China Chem..

[cit68] Ebner C., Carreira E. M. (2017). Chem. Rev..

[cit69] Shearer J., Castro J. L., Lawson A. D. G., MacCoss M., Taylor R. D. (2022). J. Med. Chem..

[cit70] Ma S., Mandalapu D., Wang S., Zhang Q. (2022). Nat. Prod. Rep..

[cit71] Yan H., Hou Z.-W., Xu H.-C. (2019). Angew. Chem., Int. Ed..

[cit72] Yan M., Kawamata Y., Baran P. S. (2017). Chem. Rev..

[cit73] Francke R., Little R. D. (2014). Chem. Soc. Rev..

[cit74] Fukuzumi S., Kotani H., Ohkubo K., Ogo S., Tkachenko N. V., Lemmetyinen H. (2004). J. Am. Chem. Soc..

[cit75] Chen D., Wang Y., Ma Y., Xiong B., Ai J., Chen Y., Geng M., Shen J. (2012). ChemMedChem.

[cit76] Chen C. CCDC 2455791: Experimental Crystal Structure Determination, 2025, 10.5517/ccdc.csd.cc2nfg2n

