# Supplementary material for: Organo-catalyzed photoelectrochemical ring-contraction of arylidenecyclobutanols via radical cation-triggered semipinacol rearrangement
Source: Chem Sci. 2025 Nov 28;17(4):2295–301. doi: 10.1039/d5sc07637d (PMC12679509; doi:10.1039/d5sc07637d)
Supplement: SC-017-D5SC07637D-s001 [file SC-017-D5SC07637D-s001.pdf]

Supplementary Information (SI) for Chemical Science.
This journal is © The Royal Society of Chemistry 2025

Supporting information

Organo-catalyzed photoelectrochemical ring-contraction of arylidenecyclobutanols via radical cation-triggered semipinacol rearrangement

Yu Zheng,[1]* Chunxi Chen,[1] Xuhao Zhou,[1] Guoyang Deng,[1] Yanju Lu,[1] and Shenlin Huang[1,]*

[1]National Key Laboratory for the Development and Utilization of Forest Food Resources, Jiangsu Co-Innovation Center of Efficient Processing and Utilization of Forest Resources, Nanjing Forestry University, Nanjing 210037, China

*email: zhengy@njfu.edu.cn; shuang@njfu.edu.cn

Table of Contents

1. General Information S1
2. Optimization Studies S2
3. Preparation of the Starting Materials S4
4. General Procedure for the Synthesis of Product S10
5. Mechanistic Studies S11
6. Products Characterization S15
7. Further Transformations S26
8. X-ray Crystal Data S31
9. References S33
10. NMR Spectra S34

1. General Information

Unless otherwise stated, all glassware was oven-dried. All solvents were distilled from appropriate drying agents prior to use. All reagents were used as received from commercial suppliers unless otherwise indicated. Reactions were monitored using UV light as the visualizing agent. Flash column chromatography was performed using silica gel 60 (200-300 mesh). HRMS data were recorded on Agilent Technologies using EI or ESI mode. All ^{1}H NMR and ^{13}C NMR spectra were recorded on Bruker DRX600 and AMX-400 instruments. Chemical shifts were given in parts per million (ppm, δ), referenced to the solvent peak of $CDCl_3$, defined at δ = 7.26 (^{1}H NMR), defined at δ = 77.16 (^{13}C NMR); or DMSO-d_6, defined at 2.50 (^{1}H NMR), defined at δ = 39.52 (^{13}C NMR). Coupling constants were quoted in Hz (*J*). ^{1}H NMR spectroscopy splitting patterns were designated as singlet (s), doublet (d), triplet (t), and quartet (q). Splitting patterns that could not be interpreted or easily visualized were designated as multiplet (m) or broad (br). The 40 W blue LEDs were purchased from taobao.com. The manufacturer of the lamp is Zhongshan Langlong Lighting Appliance Co., Ltd. The model of it is PAR 30 and the power of it is 40 W.

2. Optimization Studies

Table S1 Optimization of reaction conditions.[a]

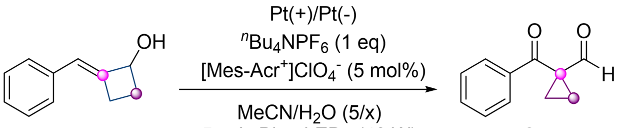

1a 2a

Entry	PC	Electrode	Electrolyte	Solvent	Current	x	Yield (%)[b]
1	[Mes-Acr$^+$]ClO_4^-	Pt(+)/Pt(-)	$^nBu_4NPF_6$	MeCN	5 mA	1.2	59 (72)[c]
2	4CzIPN	Pt(+)/Pt(-)	$^nBu_4NPF_6$	MeCN	5 mA	1.2	48
3	Ir(dF(CF$_3$)ppy)$_2$(dtbbpy)]PF$_6$	Pt(+)/Pt(-)	$^nBu_4NPF_6$	MeCN	5 mA	1.2	52
4	Eosin Y	Pt(+)/Pt(-)	$^nBu_4NPF_6$	MeCN	5 mA	1.2	31
5	PC1	Pt(+)/Pt(-)	$^nBu_4NPF_6$	MeCN	5 mA	1.2	30
6	PC2	Pt(+)/Pt(-)	$^nBu_4NPF_6$	MeCN	5 mA	1.2	46
7	PC3	Pt(+)/Pt(-)	$^nBu_4NPF_6$	MeCN	5 mA	1.2	35
8	[Mes-Acr$^+$]ClO_4^-	C(+)/Pt(-)	$^nBu_4NPF_6$	MeCN	5 mA	1.2	<5
9	[Mes-Acr$^+$]ClO_4^-	Pt(+)/C(-)	$^nBu_4NPF_6$	MeCN	5 mA	1.2	35
10	[Mes-Acr$^+$]ClO_4^-	Pt(+)/Sn(-)	$^nBu_4NPF_6$	MeCN	5 mA	1.2	32
11	[Mes-Acr$^+$]ClO_4^-	Pt(+)/Pt(-)	Et_4NPF_6	MeCN	5 mA	1.2	42
12	[Mes-Acr$^+$]ClO_4^-	Pt(+)/Pt(-)	$^nBu_4NBF_4$	MeCN	5 mA	1.2	50
13	[Mes-Acr$^+$]ClO_4^-	Pt(+)/Pt(-)	Me_4NBr	MeCN	5 mA	1.2	33
14	[Mes-Acr$^+$]ClO_4^-	Pt(+)/Pt(-)	$^nBu_4NPF_6$	CH_2Cl_2	5 mA	1.2	N.R.
15	[Mes-Acr$^+$]ClO_4^-	Pt(+)/Pt(-)	$^nBu_4NPF_6$	THF	5 mA	1.2	N.R.
16	[Mes-Acr$^+$]ClO_4^-	Pt(+)/Pt(-)	$^nBu_4NPF_6$	DMF	5 mA	1.2	N.R.
17	[Mes-Acr$^+$]ClO_4^-	Pt(+)/Pt(-)	$^nBu_4NPF_6$	MeCN	1 mA	1.2	33
18	[Mes-Acr$^+$]ClO_4^-	Pt(+)/Pt(-)	$^nBu_4NPF_6$	MeCN	3 mA	1.2	54
19	[Mes-Acr$^+$]ClO_4^-	Pt(+)/Pt(-)	$^nBu_4NPF_6$	MeCN	8 mA	1.2	37
20	[Mes-Acr$^+$]ClO_4^-	Pt(+)/Pt(-)	$^nBu_4NPF_6$	MeCN	10 mA	1.2	5
21	[Mes-Acr$^+$]ClO_4^-	Pt(+)/Pt(-)	$^nBu_4NPF_6$	MeCN	5 mA	0.1	28
22	[Mes-Acr$^+$]ClO_4^-	Pt(+)/Pt(-)	$^nBu_4NPF_6$	MeCN	5 mA	0.3	22
23	[Mes-Acr$^+$]ClO_4^-	Pt(+)/Pt(-)	$^nBu_4NPF_6$	MeCN	5 mA	0.8	34
24	[Mes-Acr$^+$]ClO_4^-	Pt(+)/Pt(-)	$^nBu_4NPF_6$	MeCN	5 mA	1	34

25	[Mes-Acr$^+$]ClO_4^-	Pt(+)/Pt(-)	^{n}Bu_4NPF_6	MeCN	5 mA	2	49
26[d]	[Mes-Acr$^+$]ClO_4^-	Pt(+)/Pt(-)	^{n}Bu_4NPF_6	MeCN	5 mA	1.2	31
27[e]	[Mes-Acr$^+$]ClO_4^-	Pt(+)/Pt(-)	^{n}Bu_4NPF_6	MeCN	-	1.2	12

[a]Reaction conditions: **1** (0.2 mmol), electrolyte (0.2 mmol, 1 equiv), PC (5 mol%), solvent (0.04 M), Blue LEDs (λ_{max} = 465 nm)， room temperature for 10 h. [b]Yield was determined by GC using 1-nitronaphthalene as internal standard. [c]Isolated yield. [d]No light. [e]No electricity.

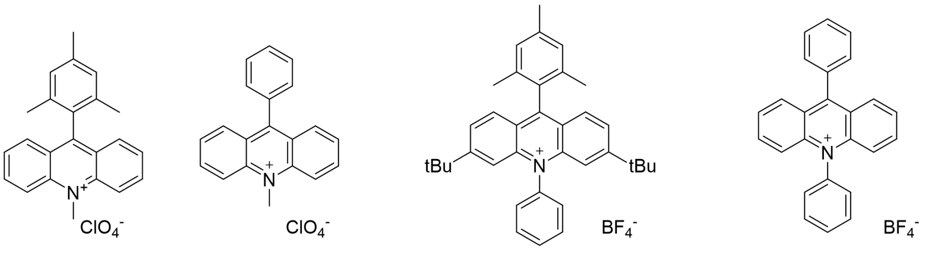

[Mes-Acr$^+$]ClO_4- PC1 PC2 PC3

3. Preparation of the Starting Materials

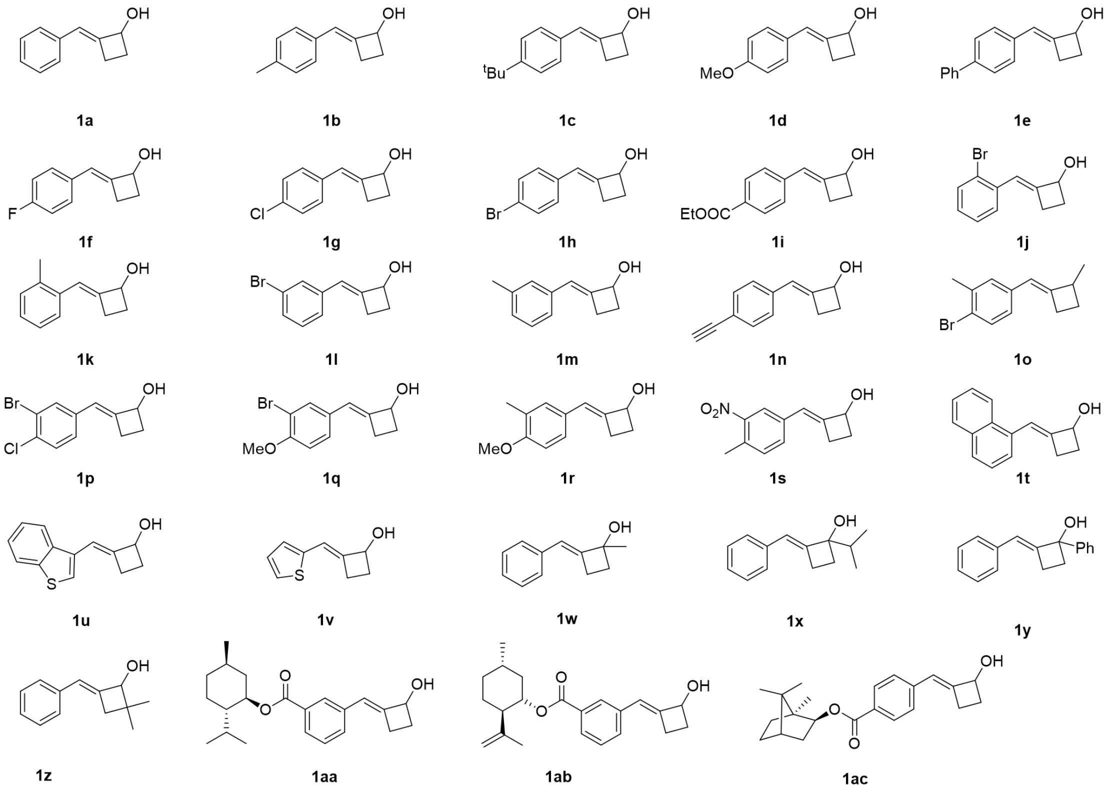

1a-1m, **1o-1q, 1t, 1u, 1w-1z** are known compounds and were synthesized according to the literature.[1,2] The preparation of new compounds and their characterization data are provided as follows.

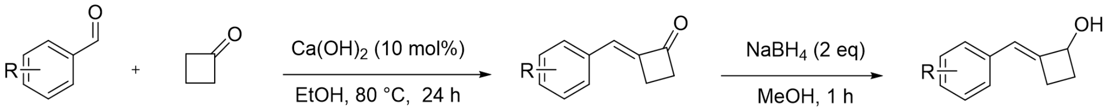

Procedure A:

To a 100 mL round-bottomed flask were added $Ca(OH)_2$ (0.1 mmol, 10 mol%), aldehydes (1.0 mmol, 1.0 equiv), cyclobutanone (3.0 mmol, 3.0 equiv), and anhydrous EtOH (5 mL) sequentially. The reaction was then heated at reflux in an oil bath under a N_2 atmosphere for 24 h. Then, the solvent was evaporated in vacuo, and the mixture was purified by flash chromatography to afford the corresponding α,β-unsaturated cyclobutanone. To the stirring solution of α,β-unsaturated cyclobutanone in 5 mL of MeOH, $NaBH_4$ (2.0 mmol, 2.0 equiv) was added at 0 °C. After a reaction time of 1 h, the solvent was evaporated in vacuo, and the mixture was purified by flash chromatography to get the corresponding products.

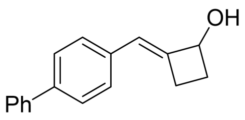

(*E*)-2-([1,1'-Biphenyl]-4-ylmethylene)cyclobutan-1-ol

^{1}H NMR (600 MHz, $CDCl_3$) δ 7.63 – 7.55 (m, 4H), 7.46 – 7.41 (m, 2H), 7.34 (ddd, *J* = 8.5, 4.1, 1.6 Hz, 3H), 6.47 (dd, *J* = 4.6, 2.3 Hz, 1H), 4.88 (t, *J* = 7.4 Hz, 1H), 2.89 – 2.80 (m, 1H), 2.71 (dtd, *J* = 11.7, 9.0, 2.6 Hz, 1H), 2.59 – 2.49 (m, 1H), 1.99 (tdd, *J* = 10.5, 9.1, 7.1 Hz, 2H).

These data are in accordance with the literature.[1]

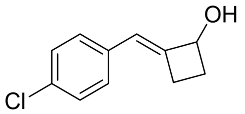

(*E*)-2-(4-Chlorobenzylidene)cyclobutan-1-ol

^{1}H NMR (600 MHz, $CDCl_3$) δ 7.28 (d, *J* = 8.5 Hz, 2H), 7.18 (d, *J* = 8.5 Hz, 2H), 6.38 (d, *J* = 2.3 Hz, 1H), 4.84 (s, 1H), 4.84 (s, 1H), 2.81 – 2.70 (m, 1H), 2.65 (dtd, *J* = 11.6, 9.0, 2.6 Hz, 1H), 2.56 – 2.48 (m, 1H), 2.03 – 1.90 (m, 2H).

These data are in accordance with the literature.[1]

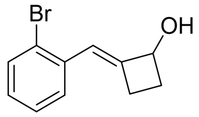

(*E*)-2-(2-bromobenzylidene)cyclobutan-1-ol

^{1}H NMR (600 MHz, $CDCl_3$) δ 7.57 (dd, *J* = 8.0, 1.1 Hz, 1H), 7.31 – 7.29 (m, 1H), 7.26 (dd, *J* = 4.8, 3.2 Hz, 1H), 7.06 (d, *J* = 1.3 Hz, 1H), 6.75 (d, *J* = 2.3 Hz, 1H), 4.85 (t, *J* = 7.7 Hz, 1H), 2.73 – 2.58 (m, 2H), 2.55 – 2.46 (m, 1H), 1.96 (dddd, *J* = 17.5, 10.2, 9.3, 7.3 Hz, 2H).

These data are in accordance with the literature.[1]

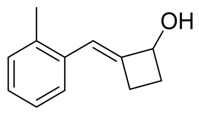

(*E*)-2-(2-Methylbenzylidene)cyclobutan-1-ol

^{1}**H NMR** (600 MHz, $CDCl_3$) δ 7.24 (d, J = 7.4 Hz, 1H), 7.14 (dt, J = 20.5, 7.4 Hz, 3H), 6.60 (s, 1H), 4.86 (d, J = 7.1 Hz, 1H), 2.72 – 2.58 (m, 2H), 2.54 – 2.46 (m, 1H), 2.35 (s, 3H), 1.96 – 1.89 (m, 1H).

These data are in accordance with the literature.[1]

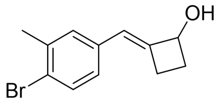

(*E*)-2-(4-Bromo-3-methylbenzylidene)cyclobutan-1-ol

^{1}**H NMR** (600 MHz, $CDCl_3$) δ 7.56 (d, J = 8.2 Hz, 1H), 7.36 – 7.33 (m, 1H), 7.20 (dd, J = 8.4, 2.2 Hz, 1H), 6.95 (t, J = 2.8 Hz, 1H), 3.15 (dd, J = 9.1, 6.5 Hz, 2H), 2.97 (dd, J = 8.3, 2.8 Hz, 2H), 2.42 (s, 3H).

These data are in accordance with the literature.[2]

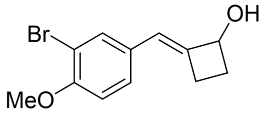

(*E*)-2-(3-Bromo-4-methoxybenzylidene)cyclobutan-1-ol

^{1}**H NMR** (600 MHz, $CDCl_3$) δ 7.45 (d, J = 2.0 Hz, 1H), 7.16 (dd, J = 8.5, 2.0 Hz, 1H), 6.88 – 6.83 (m, 1H), 6.30 (dd, J = 4.5, 2.2 Hz, 1H), 4.83 (t, J = 7.4 Hz, 1H), 3.89 (s, 3H), 2.81 – 2.70 (m, 1H), 2.64 (dtd, J = 11.6, 9.0, 2.6 Hz, 1H), 2.56 – 2.46 (m, 1H), 1.98 – 1.93 (m, 1H).

^{13}**C NMR** (151 MHz, $CDCl_3$) δ 154.6, 147.1, 132.5, 131.1, 128.0, 118.4, 111.8 (d, J = 15.7 Hz), 72.2, 56.4, 32.0, 25.0, 1.1.

HRMS (ESI**)** m/z $(M+H)^+$ calcd 269.0172, found 269.0167.

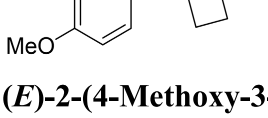

(*E*)-2-(4-Methoxy-3-methylbenzylidene)cyclobutan-1-ol

^{1}**H NMR** (600 MHz, $CDCl_3$) δ 7.09 – 7.04 (m, 2H), 6.79 (d, J = 8.3 Hz, 1H), 6.34 (d, J = 2.2 Hz, 1H), 4.82 (t, J = 7.3 Hz, 1H), 3.83 (s, 3H), 2.77 (s, 1H), 2.66 (d, J = 2.5 Hz, 1H), 2.50 (d, J = 8.5 Hz, 1H), 2.21 (s, 3H), 1.98 – 1.91 (m, 1H).

^{13}C NMR (151 MHz, $CDCl_3$) δ 156.8, 145.2, 130.33, 129.31, 126.6, 126.4, 119.8, 110.0, 72.4, 55.4, 32.1, 25.1, 16.4.

HRMS (ESI**)** m/z $(M+H-H_2O)^+$ calcd 187.1117, found 187.1114.

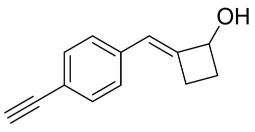

(*E*)-2-(4-Ethynylbenzylidene)cyclobutan-1-ol

^{1}H NMR (600 MHz, $CDCl_3$) δ 7.44 (d, *J* = 8.3 Hz, 2H), 7.20 (d, *J* = 8.2 Hz, 2H), 6.41 (d, *J* = 2.3 Hz, 1H), 4.85 (s, 1H), 3.09 (s, 1H), 2.83 – 2.73 (m, 1H), 2.68 (ddd, *J* = 9.4, 8.0, 2.6 Hz, 1H), 2.57 – 2.48 (m, 1H), 2.02 – 1.92 (m, 2H).

^{13}C NMR (151 MHz, $CDCl_3$) δ 149.6, 137.4, 132.3, 127.7, 120.2, 119.6, 83.9, 77.6, 72.3, 31.9, 25.3.

HRMS (ESI**)** m/z $(M)^+$ calcd 185.0961, found 185.0959.

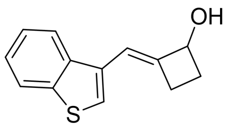

(*E*)-2-(Benzo[b]thiophen-3-ylmethylene)cyclobutan-1-ol

^{1}H NMR (600 MHz, $CDCl_3$) δ 7.88 – 7.84 (m, 2H), 7.43 – 7.34 (m, 2H), 7.22 (s, 1H), 6.78 (dd, *J* = 4.6, 2.2 Hz, 1H), 4.91 (s, 1H), 2.79 – 2.72 (m, 1H), 2.64 (dtd, *J* = 11.6, 8.9, 2.7 Hz, 1H), 2.59 – 2.53 (m, 1H), 1.99 (tdd, *J* = 10.6, 8.9, 7.0 Hz, 1H).

^{13}C NMR (151 MHz, $CDCl_3$) δ 149.2, 139.8, 138.4, 132.0, 124.6, 124.2, 122.8, 122.2, 121.8, 111.6, 72.1, 32.0, 25.3.

HRMS (EI) m/z $(M)^+$ calcd 216.0603, found 216.0600.

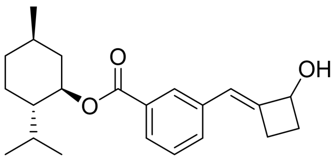

(1R,2S,5R)-2-Isopropyl-5-methylcyclohexyl-3-((*E*)-(2-hydroxycyclobutylidene)methyl)benzoate

^{1}H NMR (600 MHz, $CDCl_3$) δ 7.93 (d, *J* = 1.2 Hz, 1H), 7.87 (d, *J* = 7.6 Hz, 1H), 7.42 (d, *J* = 6.7 Hz, 1H), 7.38 (dd, *J* = 9.6, 5.7 Hz, 1H), 6.48 – 6.46 (m, 1H), 4.94 – 4.84 (m, 2H), 2.84 – 2.64 (m, 2H), 2.52 (dtd, *J* = 11.7, 8.4, 3.4 Hz, 1H), 2.13 (dd, *J* = 8.3, 3.8 Hz, 1H), 2.03 – 1.91 (m, 3H), 1.77 – 1.69 (m, 2H), 1.55 (tt, *J* = 6.0, 3.1 Hz, 2H), 1.18 – 1.04 (m, 2H), 0.92 (dd, *J* = 6.6, 5.5 Hz, 7H), 0.79 (d, *J* = 6.9 Hz, 3H).
^{13}C NMR (151 MHz, $CDCl_3$) δ 166.2, 149.3, 137.1, 131.9, 131.1, 128.9, 128.5, 127.7, 119.3, 75.0, 72.2, 47.4, 41.0, 34.4, 31.9, 31.5, 26.6, 25.2, 23.7, 22.1, 20.9, 16.6.
HRMS (EI) m/z $(M)^+$ calcd 343.2268, found 343.2264.

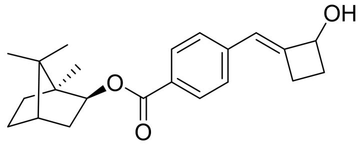

(1S,2S,4S)-1,7,7-Trimethylbicyclo[2.2.1]heptan-2-yl-4-((*E*)-(2-hydroxycyclobutylidene)methyl)benzoate

^{1}H NMR (600 MHz, $CDCl_3$) δ 7.94 (d, J = 8.0 Hz, 2H), 7.27 (dd, J = 8.1, 2.0 Hz, 2H), 6.45 (s, 1H), 5.01 (td, J = 10.9, 4.4 Hz, 1H), 4.85 (s, 1H), 4.79 (s, 1H), 4.71 (d, J = 1.2 Hz, 1H), 2.77 (ddd, J = 15.6, 10.0, 3.4 Hz, 1H), 2.72 – 2.63 (m, 1H), 2.55 – 2.48 (m, 1H), 2.30 (dt, J = 14.4, 5.5 Hz, 1H), 2.15 (t, J = 11.6 Hz, 1H), 2.02 – 1.94 (m, 1H), 1.75 (ddd, J = 22.3, 12.7, 8.3 Hz, 3H), 1.69 (s, 3H), 1.51 – 1.41 (m, 3H), 0.95 (d, J = 6.6 Hz, 4H).
^{13}C NMR (151 MHz, $CDCl_3$) δ 166.0, 150.9, 146.2, 141.2, 129.8, 128.8, 127.5, 119.4, 112.0, 74.4, 72.1, 51.0, 40.6, 34.3, 31.7, 31.5, 30.5, 25.4, 22.1, 19.6, 15.4.
HRMS (EI) m/z $(M)^+$ calcd 341.2111, found 341.2112.

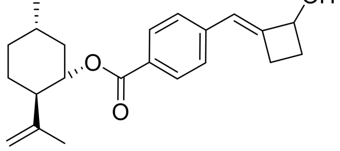

(1S,2R,5S)-5-Methyl-2-(prop-1-en-2-yl)cyclohexyl-4-((*E*)-(2-hydroxycyclobutylidene)methyl)benzoate

^{1}H NMR (600 MHz, $CDCl_3$) δ 7.99 (d, *J* = 8.3 Hz, 2H), 7.29 (d, *J* = 8.3 Hz, 2H), 6.46 (d, *J* = 2.2 Hz, 1H), 5.12 – 5.07 (m, 1H), 4.88 – 4.82 (m, 1H), 2.81 – 2.74 (m, 1H), 2.72 – 2.64 (m, 1H), 2.54 – 2.42 (m, 3H), 2.16 – 2.09 (m, 1H), 2.03 – 1.95 (m, 1H), 1.84 – 1.75 (m, 1H), 1.72 (t, *J* = 4.5 Hz, 1H), 1.43 – 1.36 (m, 1H), 1.30 (ddd, *J* = 16.4, 9.0, 4.0 Hz, 1H), 1.13 – 1.09 (m, 1H), 0.95 (s, 3H), 0.90 (d, *J* = 2.3 Hz, 6H).
^{13}C NMR (151 MHz, $CDCl_3$) δ 166.8, 151.1, 141.4, 129.7, 128.7, 127.6, 119.4, 80.6, 77.3, 77.1, 76.9, 72.1, 49.1, 47.9, 45.0, 37.0, 31.6, 28.1, 27.4, 25.4, 19.8, 19.0, 13.7.
HRMS (EI) m/z $(M)^+$ calcd 341.2111, found 341.2104.

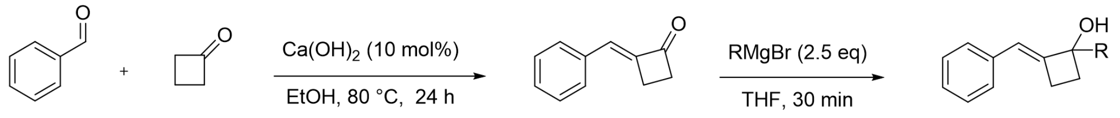

Procedure B:

Following General Procedure A to afford the alkylidenecyclobutanone. Then a vial was charged with alkylidenecyclobutanone (316.4 mg, 2 mmol,1 equiv) and evacuated under high vacuum and backfilled with N_2. THF (10 mL) was next added to dissolve alkylidenecyclobutanone. Then, Grignard Reagent (1.7 mL, 5 mmol, 2.5 equiv, 3 M in THF) was added, and the solution was stirred at room temperature for 30 min. The resulting mixture was then quenched by a saturated aqueous solution of NH_4Cl (20 mL). After extraction with diethyl ether, the organic layer was washed with brine, dried over anhydrous Na_2SO_4, filtered, and concentrated. The residue was purified by column chromatography over silica gel to afford substrates.

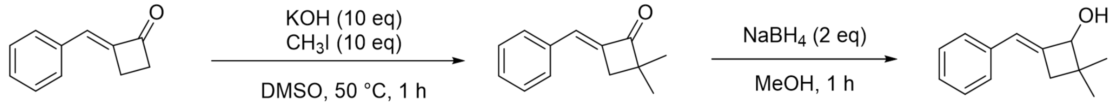

Procedure C:

To a 100 mL flask was added KOH (20 mmol, 10 equiv) and DMSO (4 mL). To this was added CH_3I (20 mmol, 10 equiv) and (*E*)-2-benzylidenecyclobutan-1-one (2 mmol, 1 equiv) in DMSO (4 mL) through an addition funnel. The suspension was stirred at 50 °C for 1 h. The resulting mixture was washed with water and then

extracted with ethyl acetate. Combined organic dried over Na_2SO_4. The residue was purified by column chromatography over silica gel to afford substrates.

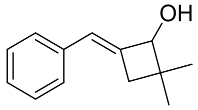

(*E*)-4-Benzylidene-2,2-dimethylcyclobutan-1-ol

^{1}H NMR (600 MHz, $CDCl_3$) δ 7.32 (t, *J* = 7.5 Hz, 2H), 7.27 – 7.23 (m, 2H), 7.20 (t, *J* = 7.3 Hz, 1H), 6.48 (s, 1H), 4.43 (s, 1H), 2.46 (d, *J* = 17.6 Hz, 2H), 1.27 (s, 3H), 1.05 (s, 3H).

^{13}C NMR (151 MHz, $CDCl_3$) δ 145.1, 137.0, 128.5, 127.8, 126.7, 121.0, 79.2, 40.8, 40.1, 27.3, 20.6.

HRMS (ESI**)** m/z $(M+H-H_2O)^+$ calcd 171.1168, found 171.1166.

4. General Procedure for the Synthesis of Product

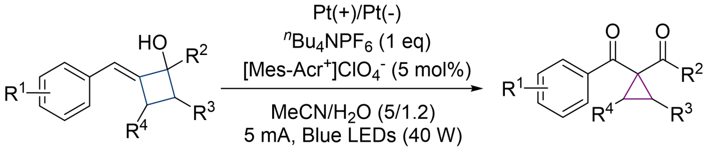

Procedure D:

To a 10 mL Schlenk tube equipped with a magnetic stir bar was charged with the substrate (0.2 mmol, 1 equiv), 9-mesityl-10-methyl acridinium perchlorate (0.01 mmol, 5 mol%, 4.1 mg), and ^{n}Bu$_4$NPF$_6$ (0.2 mmol, 1 equiv, 77.4 mg). The Schlenk tube was equipped with Pt plates (10 mm × 10 mm × 0.1 mm) as the anode and the cathode. CH_3CN (5 mL) and H_2O (1.2 mL) were then added. The Schlenk tube was placed on a magnetic stirrer, irradiated with a 40 W blue light, and electrolyzed at a constant current of 5 mA for 10 h. The residue was chromatographed through silica gel eluting with ethyl acetate/petroleum ether to give the product.

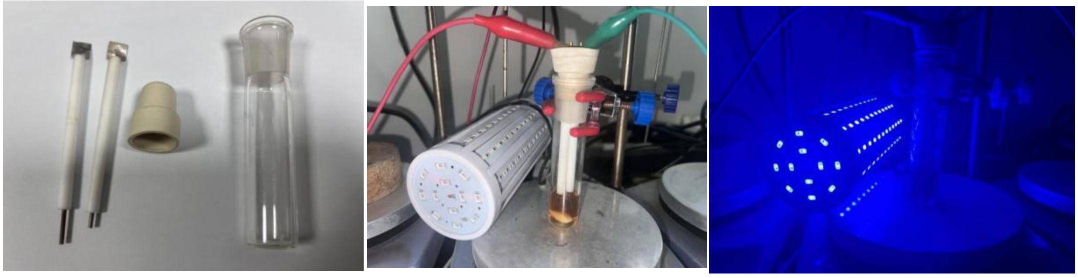

Figure S1 Reaction setup.

5. Mechanistic Studies

5.1 Radical scavenging experiments.

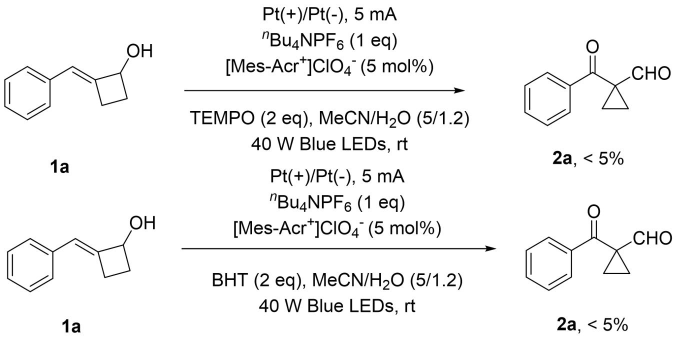

To a 10 mL Schlenk tube equipped with a magnetic stir bar was charged with the substrate (0.2 mmol, 1 equiv), 9-mesityl-10-methyl acridinium perchlorate (0.01 mmol, 5 mol%, 4.1 mg), TEMPO (0.4 mmol, 2 equiv) or BHT (0.4 mmol, 2 equiv), and $^{n}Bu_4NPF_6$ (0.2 mmol, 1 equiv, 77.4 mg). The Schlenk tube was equipped with Pt plates (10 mm × 10 mm × 0.1 mm) as the anode and the cathode. CH_3CN (5 mL) and H_2O (1.2 mL) were then added. The Schlenk tube was placed on a magnetic stirrer, irradiated with a 40 W blue light, and electrolyzed at a constant current of 5 mA. Both of the radical scavengers exhibited inhibitory effects on this reaction, and only a trace of product was observed.

5.2 Cyclic voltammetry studies.

Cyclic voltametric experiments were conducted on CHI600E in a three-electrode cell (beakertype cell) at room temperature. The cyclic voltammograms were recorded in an electrolyte solution of $^{n}Bu_4NPF_6$ (0.04 M) in CH_3CN (5 mL) using a glassy carbon

working electrode, a platinum wire counter electrode, and an Ag/AgCl reference electrode. For the CV experiments, the initial potential was 0.0 V, the switching potential was +3.0 V, and the scan rate was 100 mV/s.

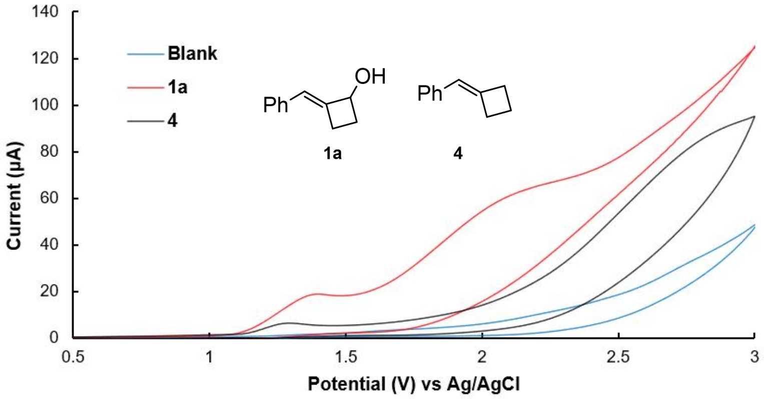

Figure S2 CVs of blank (0.04 M ${}^{n}Bu_4NPF_6$ in CH_3CN, blue line), the mixture of 0.04 M **1a** with 0.04 M ${}^{n}Bu_4NPF_6$ in CH_3CN (red line), the mixture of 0.04 M **4** with 0.04 M ${}^{n}Bu_4NPF_6$ in CH_3CN (black line) at 100 mV/s were scanned from 0 V to 3 V using glassy carbon as the working electrode.

5.3 Stern-Volmer quenching experiments

Steady-state emission spectra were obtained at room temperature with a scan range of 385 nm to 710 nm. Emission slit width was maintained at 5.0 nm. The photocatalyst, quencher, and MeCN solution were added to vials. The [Mes-Acr$^+$]ClO_4^- solution was excited at 365 nm, and the emission intensity at 510 nm was recorded. In a typical experiment, the solution of [Mes-Acr$^+$]ClO_4^- (3 mM) was added to the appropriate amount of quencher in a screw-top 1.0 cm quartz cuvette.

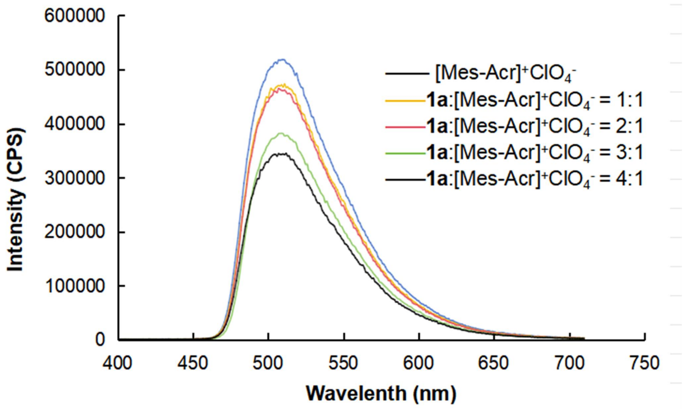

Figure S3 Fluorescence quenching experiment.

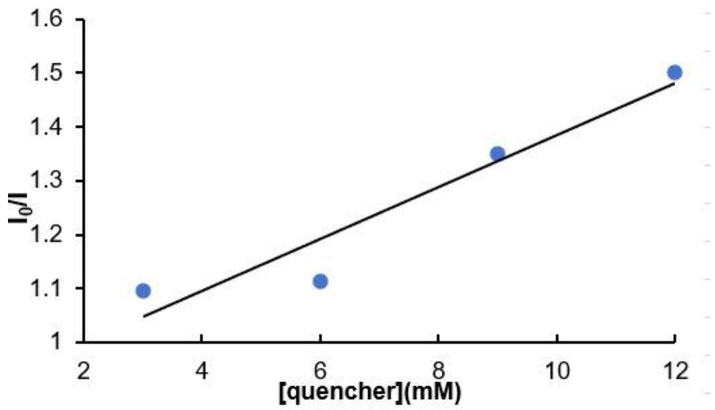

Figure S4 Stern-Volmer quenching analysis.

5.4 Labelling experiment

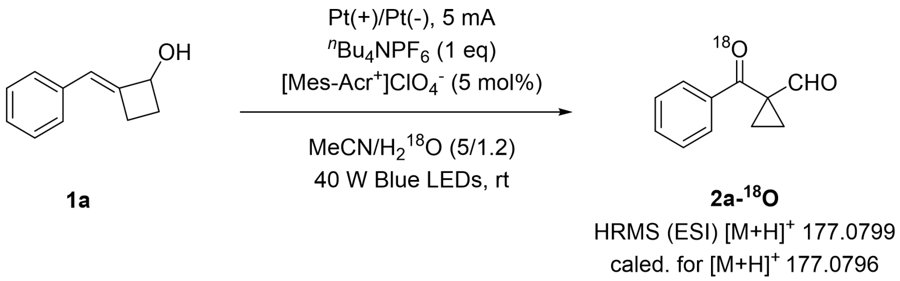

To a 10 mL Schlenk tube equipped with a magnetic stir bar was charged with **1a** (0.2 mmol, 1 equiv), 9-mesityl-10-methyl acridinium perchlorate (0.01 mmol, 5 mol%), and $^{n}Bu_4NPF_6$ (0.2 mmol, 1 equiv). The Schlenk tube was equipped with Pt plates (10 mm × 10 mm × 0.1 mm) as the anode and the cathode. CH_3CN (5 mL) and $H_2{}^{18}O$ (1.2 mL) were then added. The Schlenk tube was placed on a magnetic stirrer, irradiated with a 40 W blue light, and electrolyzed at a constant current of 5 mA for 10 h. Finally, using HRMS analysis detected the presence of **2a-^{18}O** in the reaction mixture.

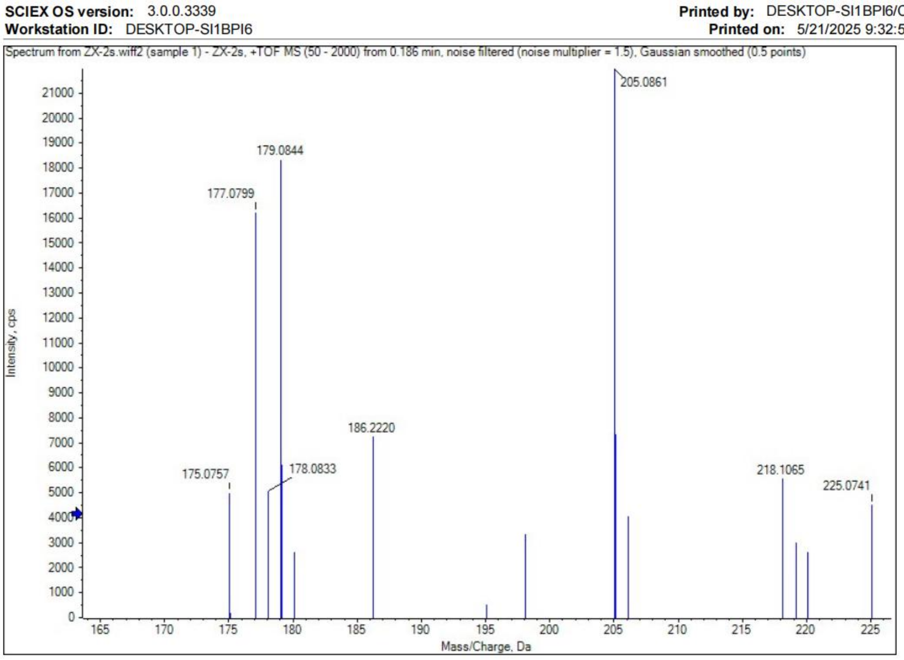

Figure S5 HRMS analysis of the reaction mixture.

5.5 TMS-Protected experiment

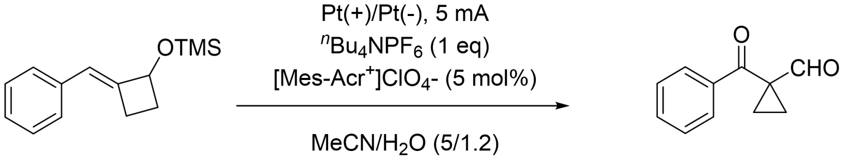

3 **2a**, 45%

To a 10 mL Schlenk tube equipped with a magnetic stir bar was charged **3** (0.2 mmol, 1 equiv), 9-mesityl-10-methyl acridinium perchlorate (0.01 mmol, 5 mol%), and $^{n}Bu_4NPF_6$ (0.2 mmol, 1 equiv). The Schlenk tube was equipped with Pt plates (10 mm × 10 mm × 0.1 mm) as the anode and the cathode. CH_3CN (5 mL) and H_2O (1.2 mL) were then added. The Schlenk tube was placed on a magnetic stirrer, irradiated with a 40 W blue light, and electrolyzed at a constant current of 5 mA for 10 h. The residue was chromatographed through silica gel eluting with ethyl acetate/petroleum ether to give the **2a** product. This indicates that the reaction involves the oxidation of the alkenes first.

5.6 The isolation of intermediated 5

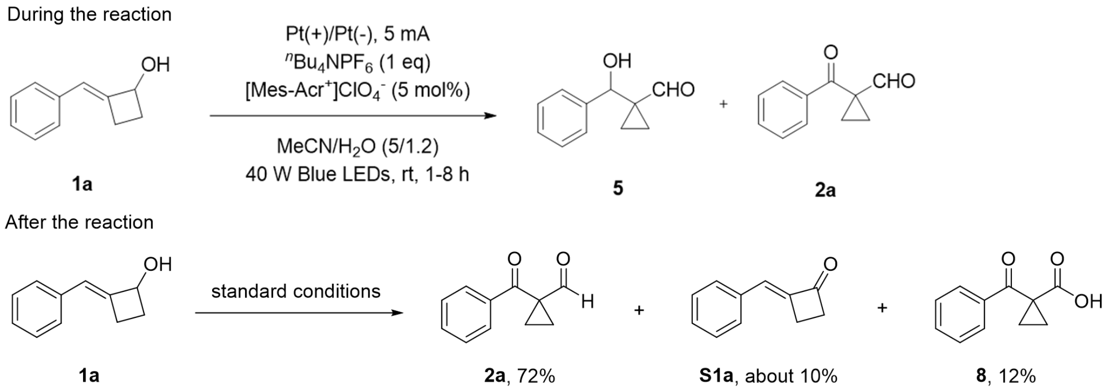

The formation of **5** was observed using TLC during the reaction process. It disappears after the reaction is completed. Its structure was determined by NMR and HMRS. After the reaction, we also observed the formation of **S1a** and **8** as main byporducts.

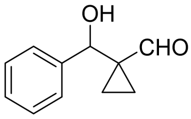

^{1}H NMR (400 MHz, CDCl$_3$) δ 8.89 (s, 1H), 7.36 – 7.25 (m, 5H), 5.23 (d, *J* = 2.4 Hz, 1H), 3.06 (d, *J* = 3.4 Hz, 1H), 1.23 – 1.10 (m, 2H), 0.95 – 0.85 (m, 2H).

^{13}C NMR (101 MHz, CDCl$_3$) δ 202.4, 140.1, 128.4, 128.0, 126.9, 72.2, 37.8, 12.3, 9.9.

HRMS (EI**)** m/z (M+H-H$_2$O)$^+$ calcd 159.0804, found 159.0809.

6. Products Characterization

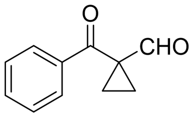

1-Benzoylcyclopropane-1-carbaldehyde (2a). 25.1 mg; Yield = 72%; White solid. Mp 60-62 °C.

^{1}H NMR (600 MHz, CDCl$_3$) δ 9.77 (s, 1H), 7.81 (d, *J* = 7.5 Hz, 2H), 7.60 (t, *J* = 7.4 Hz, 1H), 7.50 (t, *J* = 7.7 Hz, 2H), 1.82 (dd, *J* = 7.5, 3.7 Hz, 2H), 1.75 (dd, *J* = 7.5, 3.7 Hz, 2H);

^{13}C NMR (151 MHz, CDCl$_3$) δ 197.9, 197.2, 137.0, 133.4, 129.0, 77.3, 77.1, 76.9, 41.3, 20.2.

HRMS (EI**)** m/z (M)$^+$ calcd 174.0675, found 174.0666.

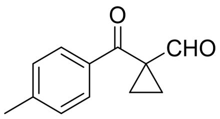

1-(4-Methylbenzoyl)cyclopropane-1-carbaldehyde (2b). 18.6 mg; Yield = 48%; White solid. Mp 75-76 °C.

^{1}H NMR (600 MHz, $CDCl_3$) δ 9.76 (s, 1H), 7.71 (d, *J* = 8.1 Hz, 2H), 7.29 (d, *J* = 8.0 Hz, 2H), 2.43 (s, 3H), 1.79 (t, *J* = 3.6 Hz, 2H), 1.72 (t, *J* = 3.6 Hz, 2H).

^{13}C NMR (151 MHz, $CDCl_3$) δ 198.1, 196.6, 144.4, 134.4, 129.7, 129.2, 41.2, 21.8, 19.9.

HRMS (ESI**)** m/z $(M+H)^+$ calcd 189.0910, found 189.0915.

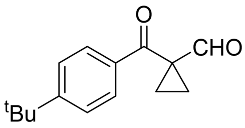

1-(4-(Tert-butyl)benzoyl)cyclopropane-1-carbaldehyde(2c). 23.4 mg; Yield = 51%; White solid. Mp 96-97 °C.

^{1}H NMR (600 MHz, $CDCl_3$) δ 9.79 (s, 1H), 7.83 – 7.69 (m, 2H), 7.57 – 7.43 (m, 2H), 1.80 – 1.77 (m, 2H), 1.74 – 1.71 (m, 2H), 1.35 (s, 9H).

^{13}C NMR (151 MHz, $CDCl_3$) δ 198.0, 196.4, 157.1, 134.1, 128.9, 125.8, 77.2, 77.0, 76.8, 41.1, 35.2, 31.0, 19.8.

HRMS (EI**)** m/z $(M)^+$ calcd 230.1301, found 230.1298.

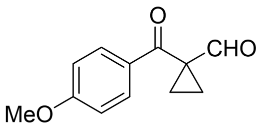

1-(4-Methoxybenzoyl)cyclopropane-1-carbaldehyde (2d). 18.3 mg; Yield = 45%; White solid. Mp 71-72 °C.

^{1}H NMR (600 MHz, $CDCl_3$) δ 9.73 (s, 1H), 7.82 (d, *J* = 8.7 Hz, 2H), 6.97 (d, *J* = 8.7 Hz, 2H), 3.88 (s, 3H), 1.77 (d, *J* = 3.8 Hz, 2H), 1.70 (dd, *J* = 7.5, 3.8 Hz, 2H).

^{13}C NMR (151 MHz, $CDCl_3$) δ 198.2, 195.0, 163.8, 131.5, 129.7, 114.2, 55.7, 41.0, 19.3.

HRMS (EI**)** m/z (M)$^+$ calcd 204.0781, found 204.0777.

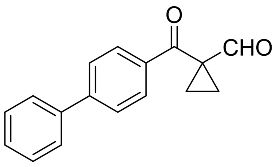

1-([1,1'-Biphenyl]-4-carbonyl)cyclopropane-1-carbaldehyde (2e). 24.5 mg; Yield = 49%; White solid. Mp135-137 °C.

^{1}H NMR (600 MHz, $CDCl_3$) δ 9.79 (s, 1H), 7.90 (d, *J* = 8.3 Hz, 2H), 7.72 (d, *J* = 8.3 Hz, 2H), 7.63 (d, *J* = 7.3 Hz, 2H), 7.48 (s, 2H), 7.42 (t, *J* = 7.4 Hz, 1H), 1.84 (d, *J* = 3.8 Hz, 2H), 1.77 (d, *J* = 3.8 Hz, 2H).

^{13}C NMR (151 MHz, $CDCl_3$) δ 197.9, 196.5, 146.2, 139.8, 135.6, 129.7, 129.1, 128.5, 127.6, 127.4, 41.3, 19.9.

HRMS (EI**)** m/z (M)$^+$ calcd 251.1067, found 251.1065.

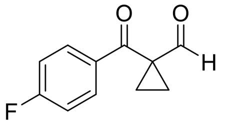

1-(4-Fluorobenzoyl)cyclopropane-1-carbaldehyde (2f). 19.9 mg; Yield = 52%; Colorless oil.

^{1}H NMR (400 MHz, $CDCl_3$) δ 9.65 (s, 1H), 7.88 – 7.83 (m, 2H), 7.21 – 7.14 (m, 2H), 1.81 (t, *J* = 3.4 Hz, 2H), 1.74 (t, *J* = 3.4 Hz, 2H).

^{13}C NMR (151 MHz, $CDCl_3$) δ 198.3, 195.2, 137.9, 134.2, 132.6, 119.3, 43.1, 19.8.

^{19}F NMR (565 MHz, $CDCl_3$) δ -104.2.

HRMS (ESI) m/z (M+H)$^+$ calcd 193.0659, found 193.0661.

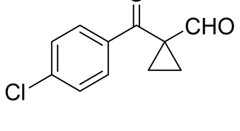

1-(4-Chlorobenzoyl)cyclopropane-1-carbaldehyde (2g). 25.0 mg; Yield = 60%; White solid. Mp 76-78 °C.

^{1}**H NMR** (600 MHz, CDCl3) δ 9.63 (s, 1H), 7.75 (d, *J* = 8.6 Hz, 2H), 7.47 (d, *J* = 8.6 Hz, 2H), 1.81 (t, *J* = 3.6 Hz, 2H), 1.75 – 1.72 (m, 2H).

^{13}**C NMR** (151 MHz, $CDCl_3$) δ 197.3, 195.7, 139.9, 135.2, 130.4, 129.3, 41.2, 19.4.

HRMS (EI**)** m/z $(M)^+$ calcd 208.0286, found 208.0289.

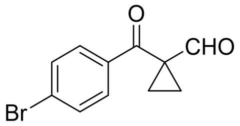

1-(4-Bromobenzoyl)cyclopropane-1-carbaldehyde (2h). 31.3 mg; Yield = 62%; White solid. Mp 88-90 °C.

^{1}**H NMR** (600 MHz, $CDCl_3$) δ 9.63 (s, 1H), 7.69 – 7.66 (m, 2H), 7.65 – 7.63 (m, 2H), 1.81 (t, *J* = 3.6 Hz, 2H), 1.75 (t, *J* = 3.5 Hz, 2H).

^{13}**C NMR** (151 MHz, $CDCl_3$) δ 197.3, 195.9, 135.6, 132.3, 130.5, 128.6, 41.2, 19.5.

HRMS (ESI) m/z $(M+H)^+$ calcd 252.9859, found 252.9851.

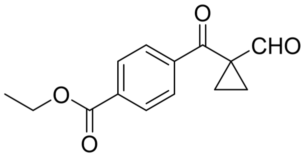

Ethyl 4-(1-formylcyclopropane-1-carbonyl)benzoate (2i). 27.5 mg; Yield = 56%; White solid. Mp 84-86 °C.

^{1}**H NMR** (600 MHz, $CDCl_3$) δ 9.67 (s, 1H), 8.15 (d, *J* = 8.4 Hz, 2H), 7.84 (d, *J* = 8.4 Hz, 2H), 4.41 (q, *J* = 7.1 Hz, 2H), 1.86 (dd, *J* = 6.1, 2.3 Hz, 2H), 1.78 (dd, *J* = 6.1, 2.3 Hz, 2H), 1.41 (t, *J* = 7.1 Hz, 3H).

^{13}**C NMR** (151 MHz, $CDCl_3$) δ 197.3, 197.0, 165.7, 140.4, 134.5, 130.1, 128.7, 61.7, 41.5, 20.2, 14.4.

HRMS (ESI) m/z $(M+H)^+$ calcd 247.0965, found 247.0962.

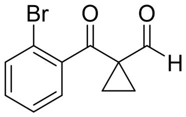

1-(2-Bromobenzoyl)cyclopropane-1-carbaldehyde (2g). 22.7 mg; Yield = 45%; Colorless oil.

^{1}H NMR (600 MHz, $CDCl_3$) δ 9.96 (s, 1H), 7.63 (d, J = 8.0 Hz, 1H), 7.44 (t, J = 7.2 Hz, 1H), 7.36 (td, J = 7.8, 1.7 Hz, 1H), 7.32 (dd, J = 7.5, 1.6 Hz, 1H), 1.97 – 1.94 (m, 2H), 1.91 – 1.88 (m, 2H).

^{13}C NMR (151 MHz, $CDCl_3$) δ 201.0, 198.1, 133.4, 132.0, 128.8, 128.0, 118.8, 42.1, 27.0.

HRMS (EI**)** m/z $(M)^+$ calcd 251.9780, found 251.9783.

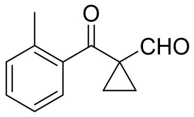

1-(2-Methylbenzoyl)cyclopropane-1-carbaldehyde (2k). 12.8 mg; Yield = 34%; White solid. Mp 91-93 °C.

^{1}H NMR (600 MHz, $CDCl_3$) δ 9.94 (s, 1H), 7.39 (ddd, J = 11.4, 9.0, 4.4 Hz, 2H), 7.28 (dd, J = 14.1, 6.3 Hz, 2H), 2.41 (s, 3H), 1.89 – 1.86 (m, 2H), 1.82 – 1.79 (m, 2H).

^{13}C NMR (151 MHz, $CDCl_3$) δ 202.2, 198.5, 138.2, 136.4, 131.7, 131.1, 127.7, 126.1, 42.5, 24.1, 20.1.

HRMS (EI**)** m/z $(M)^+$ calcd 188.0832, found 188.0830.

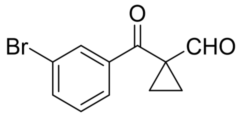

1-(3-Bromobenzoyl)cyclopropane-1-carbaldehyde (2l). 24.8 mg; Yield = 49%; White solid. Mp 73-75 °C.

^{1}H NMR (600 MHz, $CDCl_3$) δ 9.65 (s, 1H), 7.93 (s, 1H), 7.71 (t, J = 8.3 Hz, 2H), 7.37 (t, J = 7.9 Hz, 1H), 1.83 (dd, J = 7.7, 4.0 Hz, 2H), 1.76 (dd, J = 7.7, 4.0 Hz, 2H).

^{13}C NMR (151 MHz, $CDCl_3$) δ 197.2, 195.8, 136.2, 131.8, 130.5, 127.5, 123.3, 41.3, 19.8.

HRMS (EI**)** m/z $(M)^+$ calcd 251.9780, found 251.9783.

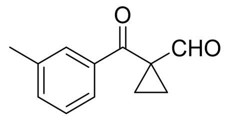

1-(3-Methylbenzoyl)cyclopropane-1-carbaldehyde (2m). 15 mg; Yield = 40%; Colorless oil.

^{1}H NMR (600 MHz, $CDCl_3$) δ 9.80 (s, 1H), 7.62 – 7.57 (m, 2H), 7.42 – 7.36 (m, 2H), 2.42 (s, 3H), 1.81 (d, J = 3.9 Hz, 2H), 1.74 (d, J = 3.9 Hz, 2H).

^{13}C NMR (151 MHz, $CDCl_3$) δ 198.0, 197.5, 139.0, 137.2, 134.1, 129.4, 128.8, 126.2, 41.3, 21.4, 20.4.

HRMS (EI**)** m/z $(M)^+$ calcd 188.0832, found 188.0825.

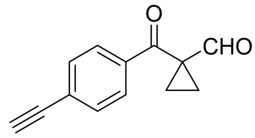

1-(4-Ethynylbenzoyl)cyclopropane-1-carbaldehyde (2n). 16.6 mg; Yield = 42%; Colorless oil.

^{1}H NMR (600 MHz, $CDCl_3$) δ 9.68 (s, 1H), 7.76 (d, J = 8.4 Hz, 2H), 7.60 (d, J = 8.4 Hz, 2H), 3.27 (s, 1H), 1.82 (t, J = 3.5 Hz, 2H), 1.75 (dd, J = 6.1, 2.4 Hz, 2H).

^{13}C NMR (151 MHz, $CDCl_3$) δ 197.5, 196.3, 136.7, 132.6, 128.9, 127.3, 82.6, 80.8, 41.3, 19.8.

HRMS (ESI) m/z $(M+H)^+$ calcd 199.0754, found 199.0750.

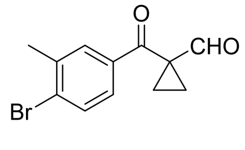

1-(4-Bromo-3-methylbenzoyl)cyclopropane-1-carbaldehyde (2o). 29.8 mg; Yield = 56%; White solid. Mp 75-76 °C.

^{1}H NMR (600 MHz, $CDCl_3$) δ 9.67 (s, 1H), 7.65 (s, 2H), 7.45 (d, J = 7.9 Hz, 1H), 2.46 (s, 3H), 1.80 (s, 2H), 1.74 (s, 2H).

^{13}C NMR (151 MHz, $CDCl_3$) δ 197.5, 196.2, 139.1, 136.0, 133.0, 130.9, 127.7, 41.2, 23.1, 19.7.

HRMS (EI**)** m/z (M)$^+$ calcd 265.9937, found 265.9932.

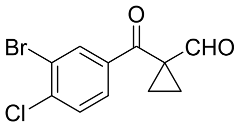

1-(3-Bromo-4-chlorobenzoyl)cyclopropane-1-carbaldehyde (2p). 31 mg; Yield = 54%; White solid. Mp 79-81 °C.

^{1}H NMR (600 MHz, $CDCl_3$) δ 9.53 (s, 1H), 8.05 (s, 1H), 7.66 (d, *J* = 8.3 Hz, 1H), 7.57 (d, *J* = 8.3 Hz, 1H), 1.82 (d, *J* = 3.2 Hz, 2H), 1.75 (d, *J* = 3.5 Hz, 2H).

^{13}C NMR (151 MHz, $CDCl_3$) δ 196.8, 194.5, 140.0, 136.5, 134.1, 130.8, 128.6, 123.5, 41.2, 19.0.

HRMS (ESI) m/z (M+H)$^+$ calcd 286.9469, found 286.9467.

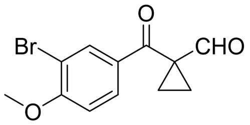

1-(3-Bromo-4-methoxybenzoyl)cyclopropane-1-carbaldehyde (2q). 27.1 mg; Yield = 48%; Colorless oil.

^{1}H NMR (600 MHz, $CDCl_3$) δ 9.62 (s, 1H), 8.05 (d, *J* = 1.6 Hz, 1H), 7.77 (dd, *J* = 8.6, 1.5 Hz, 1H), 6.95 (d, *J* = 8.6 Hz, 1H), 3.97 (s, 3H), 1.77 (t, *J* = 3.4 Hz, 2H), 1.71 (t, *J* = 3.3 Hz, 2H).

^{13}C NMR (151 MHz, $CDCl_3$) δ 197.5, 193.9, 159.9, 134.4, 130.6, 130.4, 112.5, 111.4, 56.7, 41.0, 18.9.

HRMS (ESI) m/z (M+H)$^+$ calcd 282.9964, found 282.9962.

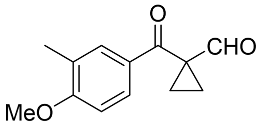

1-(4-Methoxy-3-methylbenzoyl)cyclopropane-1-carbaldehyde (2r). 22.2 mg; Yield = 51%; White solid. Mp 70-72 °C.

^{1}H NMR (600 MHz, $CDCl_3$) δ 9.76 (s, 1H), 7.68 (dd, *J* = 8.5, 2.2 Hz, 1H), 7.66 – 7.63 (m, 1H), 6.88 (s, 1H), 3.90 (s, 3H), 2.25 (s, 3H), 1.78 – 1.75 (m, 2H), 1.70 (s, 2H).

^{13}C NMR (151 MHz, $CDCl_3$) δ 198.4, 195.3, 162.1, 131.6, 129.5, 129.2, 127.5, 109.6, 55.7, 41.0, 19.5, 16.4.

HRMS (ESI) m/z $(M+H)^+$ calcd 219.1016, found 219.1011.

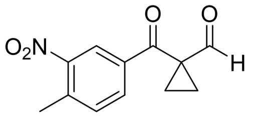

1-(4-Methyl-3-nitrobenzoyl)cyclopropane-1-carbaldehyde (2s). 18.6 mg; Yield = 42%; Colorless oil.

^{1}H NMR (600 MHz, $CDCl_3$) δ 9.44 (s, 1H), 8.38 (d, *J* = 1.7 Hz, 1H), 7.92 (dd, *J* = 7.9, 1.8 Hz, 1H), 7.48 (d, *J* = 7.9 Hz, 1H), 2.68 (s, 3H), 1.86 (dd, *J* = 7.8, 4.3 Hz, 2H), 1.77 (dd, *J* = 7.8, 4.3 Hz, 2H).

HRMS (EI) m/z $(M)^+$ calcd 234.0761, found 234.0764.

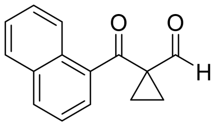

1-(1-Naphthoyl)cyclopropane-1-carbaldehyde(2t). 17.9 mg; Yield = 40%; Yellow solid. Mp 130-131 °C.

^{1}H NMR (600 MHz, $CDCl_3$) δ 9.92 (s, 1H), 8.16 (d, *J* = 8.1 Hz, 1H), 8.01 (d, *J* = 8.1 Hz, 1H), 7.93 (d, *J* = 7.3 Hz, 1H), 7.65 (dd, *J* = 7.0, 0.8 Hz, 1H), 7.60 – 7.56 (m, 2H), 7.55 – 7.52 (m, 1H), 2.01 (d, *J* = 5.3 Hz, 2H), 1.90 (d, *J* = 5.3 Hz, 2H).

^{13}C NMR (151 MHz, $CDCl_3$) δ 201.7, 198.5, 136.0, 133.9, 132.2, 130.0, 128.8, 128.1, 127.0 (d, *J* = 2.4 Hz), 125.0, 124.8, 43.2, 24.9.

HRMS (EI) m/z $(M)^+$ calcd 224.0832, found 224.0827.

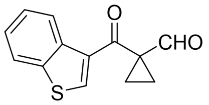

1-(Benzo[b]thiophene-3-carbonyl)cyclopropane-1-carbaldehyde(2u). 24.8 mg; Yield = 61%; White solid. Mp 80-81 °C.

^{1}H NMR (600 MHz, $CDCl_3$) δ 9.87 (s, 1H), 8.54 (d, *J* = 8.1 Hz, 1H), 8.13 (s, 1H), 7.90 (d, *J* = 8.0 Hz, 1H), 7.52 (t, *J* = 7.6 Hz, 1H), 7.46 (t, *J* = 7.5 Hz, 1H), 1.87 (dd, *J* = 7.7, 3.7 Hz, 2H), 1.76 (dd, *J* = 7.7, 3.7 Hz, 2H).

^{13}C NMR (151 MHz, $CDCl_3$) δ 197.9, 190.9, 140.1, 137.8, 136.6, 135.0, 126.1, 126.0, 125.1, 122.6, 42.6, 19.8.

HRMS (EI) m/z $(M)^+$ calcd 231.0474, found 231.0472.

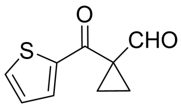

1-(Thiophene-2-carbonyl)cyclopropane-1-carbaldehyde (2v). 16.5 mg; Yield = 46%; White solid. Mp 76-78 °C.

^{1}H NMR (600 MHz, $CDCl_3$) δ 9.85 (s, 1H), 7.71 (dd, *J* = 4.9, 0.8 Hz, 1H), 7.62 (dd, *J* = 3.8, 0.8 Hz, 1H), 7.20 – 7.13 (m, 1H), 1.78 (dd, *J* = 7.8, 4.1 Hz, 2H), 1.69 (dd, *J* = 7.8, 4.1 Hz, 2H).

^{13}C NMR (151 MHz, $CDCl_3$) δ 197.4, 188.1, 143.1, 134.6, 133.6, 128.4, 41.8, 19.0.

HRMS (EI**)** m/z $(M)^+$ calcd 180.0240, found 180.0234.

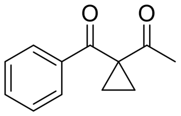

1-(1-Benzoylcyclopropyl)ethan-1-one (2w). 19.1 mg; Yield = 51%; White solid. Mp 68-70 °C.

^{1}H NMR (600 MHz, $CDCl_3$) δ 7.94 – 7.90 (m, 2H), 7.58 (t, *J* = 7.4 Hz, 1H), 7.47 (t, *J* = 7.8 Hz, 2H), 2.05 (s, 3H), 1.59 (dd, *J* = 7.3, 4.0 Hz, 2H), 1.50 (dd, *J* = 7.3, 4.0 Hz, 2H).

^{13}C NMR (151 MHz, $CDCl_3$) δ 203.9, 196.5, 137.0, 133.6, 129.0, 42.0, 29.2, 17.2.

HRMS (EI**)** m/z $(M)^+$ calcd 188.0832, found 188.0826.

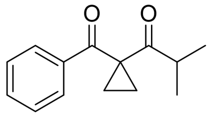

1-(1-Benzoylcyclopropyl)-2-methylpropan-1-one (2x). 25 mg; Yield = 58%; Colorless oil.

^{1}H NMR (600 MHz, $CDCl_3$) δ 7.91 – 7.87 (m, 2H), 7.57 (s, 1H), 7.46 (t, *J* = 7.7 Hz, 2H), 2.57 (dt, *J* = 13.6, 6.8 Hz, 1H), 1.55 (dt, *J* = 6.4, 3.0 Hz, 2H), 1.51 – 1.48 (m, 2H), 0.95 (d, *J* = 6.8 Hz, 6H). **^{13}C NMR** (151 MHz, $CDCl_3$) δ 210.7, 196.6, 137.1, 133.5, 129.0, 128.8, 40.9, 40.6, 19.1, 16.8.

HRMS (ESI**)** m/z $(M+H)^+$ calcd 217.1223, found 217.1217.

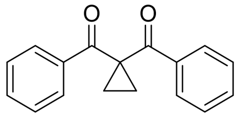

Cyclopropane-1,1-diylbis(phenylmethanone)(2y). 22.5 mg; Yield = 45%; White solid. Mp 73-74 °C.

^{1}H NMR (600 MHz, $CDCl_3$) δ 7.73 (d, *J* = 7.8 Hz, 4H), 7.35 (s, 2H), 7.25 (t, *J* = 7.3 Hz, 4H), 1.76 (s, 4H).

^{13}C NMR (151 MHz, $CDCl_3$) δ 197.6, 137.7, 132.9, 128.6 (d, *J* = 12.8 Hz), 40.8, 16.7.

HRMS (EI**)** m/z $(M)^+$ calcd 250.0988, found 250.0991

.

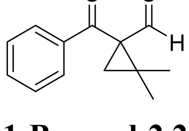

1-Benzoyl-2,2-dimethylcyclopropane-1-carbaldehyde (2z). 23.4 mg; Yield = 43%; Colorless oil.

^{1}H NMR (400 MHz, $CDCl_3$) δ 9.69 (s, 1H), 7.40 – 7.37 (m, 2H), 7.29 (m, 3H), 2.48 (d, *J* = 1.2 Hz, 2H), 1.27 (s, 6H).

^{13}C NMR (151 MHz, $CDCl_3$) δ 201.7, 192.1, 135.0, 133.8, 124.0, 121.8, 45.3, 42.2, 18.1, 12.3.

HRMS (ESI**)** m/z $(M+H)^+$ calcd 127.1223, found 127.1217.

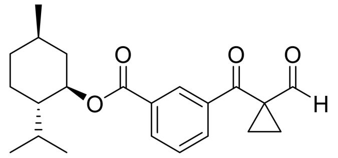

(1S,2S,5R)-2-Isopropyl-5-methylcyclohexyl-3-(1-formylcyclopropane-1-carbonyl) benzoate (2aa). Yield = 38%; Colorless oil.

^{1}H NMR (600 MHz, $CDCl_3$) δ 9.65 (s, 1H), 8.44 (s, 1H), 8.26 (d, *J* = 7.8 Hz, 1H), 7.97 (d, *J* = 7.7 Hz, 1H), 7.58 (t, *J* = 7.7 Hz, 1H), 4.95 (td, *J* = 10.9, 4.4 Hz, 1H), 2.12 (d, *J* = 11.9 Hz, 1H), 1.93 (dtd, *J* = 14.0, 7.0, 2.8 Hz, 1H), 1.85 (t, *J* = 6.0 Hz, 2H), 1.79 – 1.76 (m, 2H), 1.16 – 1.09 (m, 2H), 0.93 (t, *J* = 7.4 Hz, 8H), 0.80 (d, *J* = 6.9 Hz, 3H).

^{13}C NMR (151 MHz, $CDCl_3$) δ 197.3, 196.5, 165.1, 137.31, 134.09, 132.8, 131.8, 130.0, 129.1, 75.7, 47.3, 41.3, 41.0, 34.4, 31.6, 26.7, 23.8, 22.1, 20.8, 19.7, 16.7.

HRMS (ESI**)** m/z $(M+H)^+$ calcd 357.2060, found 357.2055.

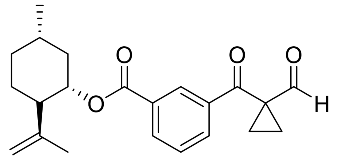

(1S,2S,5S)-5-Methyl-2-(prop-1-en-2-yl)cyclohexyl-3-(1-formylcyclopropane-1-car bonyl)benzoate (2ab). 29.7 mg**;** Yield = 42%, Colorless oil.

^{1}H NMR (600 MHz, $CDCl_3$) δ 9.67 (s, 1H), 8.12 – 8.07 (m, 2H), 7.84 – 7.79 (m, 2H), 5.04 (td, *J* = 10.9, 4.4 Hz, 1H), 4.78 (s, 1H), 4.74 – 4.70 (m, 1H), 2.36 – 2.25 (m, 1H), 2.20 – 2.12 (m, 1H), 1.88 – 1.83 (m, 2H), 1.78 (t, *J* = 3.4 Hz, 2H), 1.68 (s, 3H), 0.97 (d, *J* = 6.5 Hz, 3H).

^{13}C NMR (151 MHz, $CDCl_3$) δ 197.3, 146.1, 140.2, 130.1, 128.7, 112.2, 77.3, 77.0 (d, *J* = 27.6 Hz), 76.9, 75.1, 51.0, 41.5, 40.5, 34.2, 31.5, 30.5, 22.1, 20.1 (d, *J* = 5.4 Hz), 19.5.

HRMS (ESI**)** m/z $(M+H)^+$ calcd 355.1904, found 355.1902.

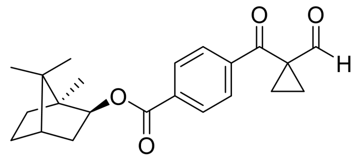

(1S,2S,4S)-1,7,7-Trimethylbicyclo[2.2.1]heptan-2-yl-4-(1-formylcyclopropane-1-carbonyl)benzoate (2ac). 29.7 mg; Yield = 42%; White solid. Mp 212-214 °C.

^{1}H NMR (600 MHz, $CDCl_3$) δ 9.66 , 8.16 (d, J = 8.4 Hz), 7.85 (d, J = 8.4 Hz), 5.16 – 5.12 (m), 2.48 (tdd, J = 16.2, 7.7, 3.6 Hz), 2.15 – 2.06 (m), 1.86 (t, J = 3.6 Hz), 1.83 – 1.80 (m), 1.78 (t, J = 3.6 Hz), 1.47 – 1.38 (m), 1.34 – 1.27 (m), 1.12 (ddd, J = 13.7, 7.5, 3.4 Hz), 0.97 (d, J = 2.1 Hz), 0.92 (d, J = 4.6 Hz).

^{13}C NMR (151 MHz, $CDCl_3$) δ 197.3, 196.9, 165.8, 131.3, 130.0, 128.8, 121.6, 81.4, 49.2 (d, J = 6.9 Hz), 48.0, 45.1, 41.5, 37.0, 28.2, 27.5, 23.5, 20.0 (d, J = 2.0 Hz), 19.8, 19.0, 13.7.

HRMS (ESI**)** m/z $(M+H)^+$ calcd 355.1904, found 355.1901.

7. Further Transformations

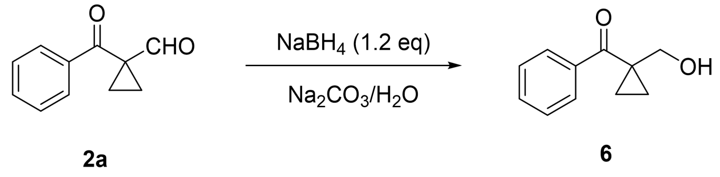

To the solution of compound **2a** (0.1 mmol, 1 equiv) in an 1:1 mixture of Na_2CO_3/H_2O, $NaBH_4$ (0.12 mmol, 1.2 equiv) was added at 0 °C. The reaction was stirred at room temperature for 1 hour. Then the solvent was directly removed under reduced pressure, and the crude mixture was further purified by flash chromatography to afford the product.

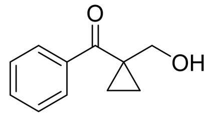

(1-(Hydroxymethyl)cyclopropyl)(phenyl)methanone (6). 10.4 mg, Yield = 60%, colorless oil.

^{1}H NMR (400 MHz, $CDCl_3$) δ 7.36 – 7.23 (m, 5H), 3.65 (s, 2H), 3.10 (s, 1H), 1.67 – 1.62 (m, 2H), 1.61 – 1.55 (m, 2H).

These data are in accordance with the literature[4].

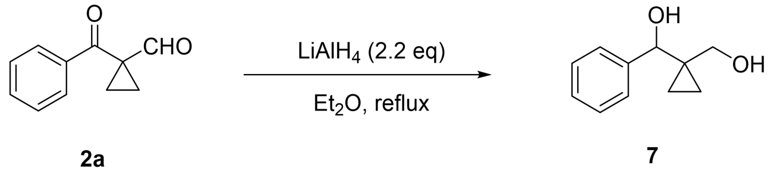

Under a N_2 atmosphere, compound **2a** (0.1 mmol, 1 equiv) was dissolved in ether (4 mL), and then $LiAlH_4$ (0.22 mmol, 2.2 equiv) was added. The mixture was refluxed for overnight. The TLC monitoring indicates that the reaction is complete when the raw materials are exhausted. After this time the solvent was removed in vacuo and the mixture was purified by flash chromatography.

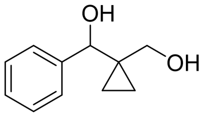

(1-(Hydroxymethyl)cyclopropyl)(phenyl)methanol (7). 13.8 mg, Yield = 78%, colorless oil.

^{1}H NMR (400 MHz, $CDCl_3$) δ 7.36 – 7.23 (m, 5H), 4.43 (s, 1H), 3.70 (d, *J* = 11.3 Hz, 1H), 3.10 (d, *J* = 11.4 Hz, 1H), 0.67 – 0.54 (m, 3H), 0.42 – 0.36 (m, 1H).

^{13}C NMR (151 MHz, $CDCl_3$)144.1, 130.2, 129.4, 128.3, 81.5, 79.5, 29.3, 11.8, 9.9.

HRMS (ESI) m/z $(M+Na)^+$ calcd 201.0886, found 201.0885.

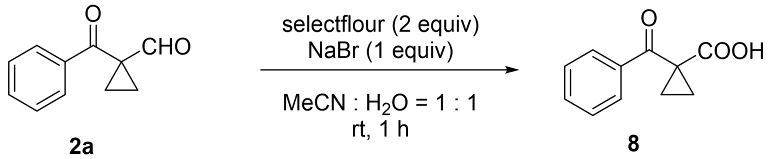

To the solution of compound **2a** (0.1 mmol, 1 equiv) in a 1:1 mixture of CH_3CN/H_2O, selectflour (0.2 mmol, 2 equiv) and NaBr (0.1 mmol, 1 equiv) were added. The reaction was stirred at room temperature for 1 hour. Then the solvent was directly removed under reduced pressure, and the crude mixture was further purified by flash chromatography.

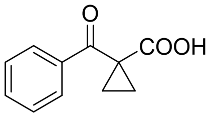

1-Benzoylcyclopropane-1-carboxylic acid (8). 8.6 mg, Yield = 45%, white solid.

^{1}H NMR (600 MHz, $CDCl_3$) δ 7.90 (d, *J* = 7.4 Hz, 2H), 7.56 (s, 1H), 7.45 (s, 2H), 1.68 (dd, *J* = 7.6, 4.1 Hz, 2H), 1.59 (dd, *J* = 7.6, 4.1 Hz, 2H).

These data are in accordance with the literature[3].

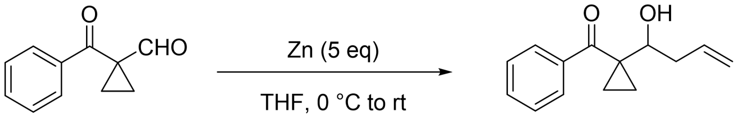

2a **9**

Compound **2a** (0.1 mmol, 1 equiv) was dissolved in THF (1 mL). Activated Zn (0.5 mmol, 5 equiv) and allyl bromide (0.15 mmol, 1.5 equiv) were added at 0 °C and the mixtures was stirred for 10 min. To this mixture saturated NH_4Cl solution (3 mL) was added dropwise at 0 °C and the solution was further stirred for 3 h at ambient temperature. The reaction mixture was extracted with EtOAc (2 x10 mL). The organic layer was separated, dried over anhydrous Na_2SO_4 and concentrated in vacuo. The residue was purified by column chromatography.

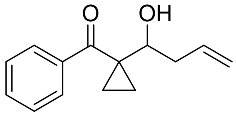

(1-(1-hydroxybut-3-en-1-yl)cyclopropyl)(phenyl)methanone (9). 10.3 mg, Yield = 48%, colorless oil.

^{1}H NMR (600 MHz, $CDCl_3$) δ 7.79 – 7.75 (m, 2H), 7.53 – 7.49 (m, 1H), 7.42 (t, *J* = 7.6 Hz, 2H), 5.83 – 5.71 (m, 1H), 5.10 (s, 1H), 5.09 – 5.06 (m, 1H), 3.88 (dd, *J* = 8.7, 4.2 Hz, 1H), 2.39 – 2.32 (m, 1H), 2.22 (ddd, *J* = 14.3, 8.6, 7.7 Hz, 1H), 1.15 (dtdd, *J* = 19.1, 9.4, 6.5, 3.9 Hz, 3H), 1.06 (ddd, *J* = 10.1, 7.2, 4.1 Hz, 1H).

^{13}C NMR (151 MHz, $CDCl_3$) δ 203.8, 137.4, 134.6, 132.1, 128.4, 118.4, 77.3, 77.1, 76.9, 72.3, 40.4, 35.0, 11.3, 9.2.

HRMS (ESI) m/z $(M+H-H_2O)^+$ calcd 199.1117, found 199.1114.

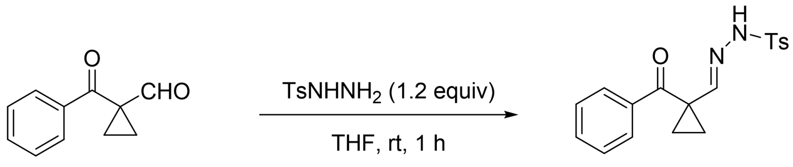

2a **10**

At room temperature, $TsNHNH_2$ (0.12 mmol, 1.2 equiv) was gradually added to the stirring solution of compound **2a** (0.1 mmol, 1 equiv) in THF (2 mL). The reaction was completed within 1 h. After that, the solvent was directly removed under reduced pressure, and the crude mixture was purified by flash chromatography.

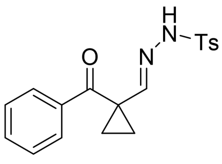

(*E*)-*N'*-((1-Benzoylcyclopropyl)methylene)-4-methylbenzenesulfonohydrazide (10). 25.3 mg, Yield = 74%, white solid. Mp 140-142 °C.

^{1}H NMR (600 MHz, $CDCl_3$) δ 7.80 (s, 1H), 7.67 (d, *J* = 8.3 Hz, 2H), 7.61 (dd, *J* = 8.2, 1.2 Hz, 2H), 7.49 (s, 1H), 7.47 (dd, *J* = 4.9, 3.7 Hz, 1H), 7.33 (d, *J* = 7.7 Hz, 2H), 7.28 (d, *J* = 8.1 Hz, 2H), 2.44 (s, 3H), 1.60 (d, *J* = 3.7 Hz, 2H), 1.43 (d, *J* = 3.7 Hz, 2H).

^{13}C NMR (151 MHz, $CDCl_3$) δ 198.7, 151.8, 144.3, 137.1, 135.1, 132.7, 129.7, 128.9, 128.6, 128.0, 32.7, 21.7, 19.3.

HRMS (ESI) m/z $(M+H)^+$ calcd 343.1111, found 343.1109.

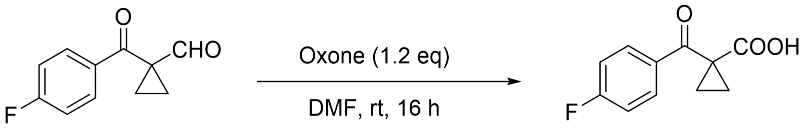

2f **11**

Compound **2f** (0.4 mmol, 1 equiv) was dissolved in DMF and Oxone (0.48 mmol, 1.2 equiv) was added in batches at room temperature for 16 h. After this time the solvent was removed in vacuo and the mixture was purified by flash chromatography.

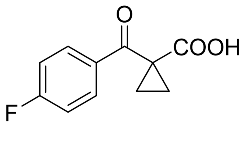

1-(4-Fluorobenzoyl)cyclopropane-1-carboxylic acid (11). 62.4 mg, Yield = 75%, white solid.

^{1}H NMR (400 MHz, $CDCl_3$): 7.92–7.95 (m, 2H), 7.10–7.14 (m, 2H), 1.66–1.69 (m, 2H), 1.55–1.58 (m, 2H).

These data are in accordance with the literature[3].

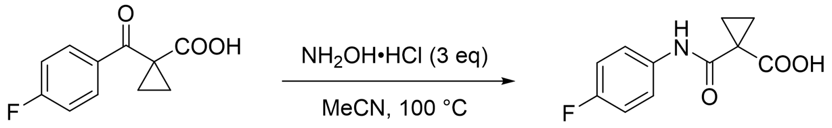

11 **12**

To the solution of compound **11** (0.2 mmol, 1 equiv) in MeCN, $NH_2OH \cdot HCl$ (0.6 mmol, 3 equiv) was added. The mixture was refluxed for overnight. After this time the solvent was removed in vacuo and the mixture was purified by flash chromatography.

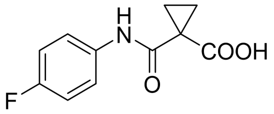

1-((4-Fluorophenyl)carbamoyl)cyclopropane-1-carboxylic acid (12). 33.0 mg, Yield = 74%, white solid.

^{1}H NMR (400 MHz, d_6-DMSO) δ 10.58 (s, 1H), 7.65 – 7.58 (m, 2H), 7.13 (t, J = 8.5 Hz, 2H), 1.41 (s, 4H).

These data are in accordance with the literature[6]

8. X-ray Crystal Data

The single crystal of compound **8** was cultivated in 4-mL glass vial by slow evaporation technique. Compound **8** (20 mg) was dissolved in the mixing solvent of ethyl acetate/petroleum ether (0.2 mL/2 mL) at ambient temperature. Ethyl acetate and petroleum ether spontaneously evaporates in open air at 25 °C. After several days, the crystal of **8** emerged and was collected for x-ray diffraction analysis. A suitable crystal was selected and then tested on a SuperNova, Dual, Mo at zero, AtlasS2 diffractometer. The crystal was kept at 100.0(2) K during data collection.

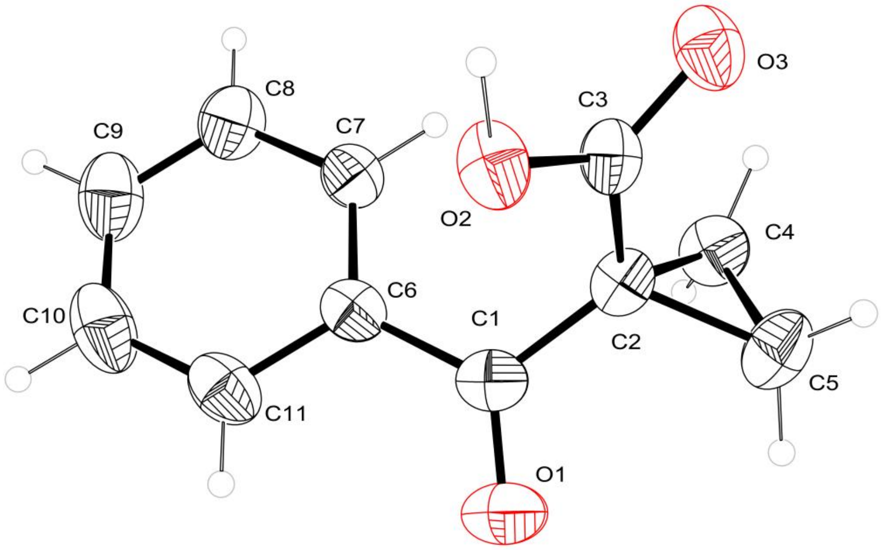

Figure S5 X-ray crystal graph.

CCDC 2455791

X-ray structure and CCDC number of **8**

Crystal data and structure refinement for **8**

Identification code	**8**
Empirical formula	$C_{11}H_{10}O_3$
Formula weight	190.19
Temperature/K	298.41(10)
Crystal system	monoclinic
Space group	$P2_1/n$
a/Å	12.2361(2)
b/Å	5.83641(11)
c/Å	13.4714(3)
α/°	90
β/°	96.6635(17)
γ/°	90

Volume/Å^3	955.55(3)
Z	4
ρ_{calc}g/cm^3	1.322
μ/mm^{-1}	0.798
F(000)	400.0
Crystal size/mm^3	0.13 × 0.12 × 0.1
Radiation	Cu Kα (λ = 1.54184)
2Θ range for data collection/°	9.246 to 145.788
Index ranges	$-13 \leq h \leq 15$, $-6 \leq k \leq 5$, $-16 \leq l \leq 16$
Reflections collected	5904
Independent reflections	1834 [R_{int} = 0.0126, R_{sigma} = 0.0130]
Data/restraints/parameters	1834/0/132
Goodness-of-fit on F^2	1.062
Final R indexes [I>=2σ (I)]	R_1 = 0.0350, wR_2 = 0.1009
Final R indexes [all data]	R_1 = 0.0388, wR_2 = 0.1047
Largest diff. peak/hole / e Å^{-3}	0.14/-0.13

9. References

1. Y. Nie, J, Z. Chen and W. B. Zhang. *Tetrahedron*, **2019**, *75*, 130563.
2. T. He, C. Liang, P. Jiang, H. Liang, S. Liao and S. Huang. *Org. Lett*., **2024**, *26*, 5577–5581.
3. G.-Q. Chen, X.-Y. Tang and M. Shi. *Chem. Commun.*, **2012**, *48*, 2340–2342.
4. K. G. Taylor. V. N. Nichols. R. Isaac and G. S. Poindexter. *J. Org. Chem*., **1974**, *39*, 1761–1763.
5. Q. Zhao, D. P. Curran, M. Malacriaa, L. Fensterbank, J.-P. Goddard and E. Lacôte. *Synlett.*, **2012**, *3*, 433–437.
6. H. Qiang, W. Gu, D. Huang, W. Shi, Q. Qiu, Y. Dai, W. Huang and H. Qian. *Bioorg. Med. Chem*., **2016**, *24*, 3353–3358.

10. NMR spectra

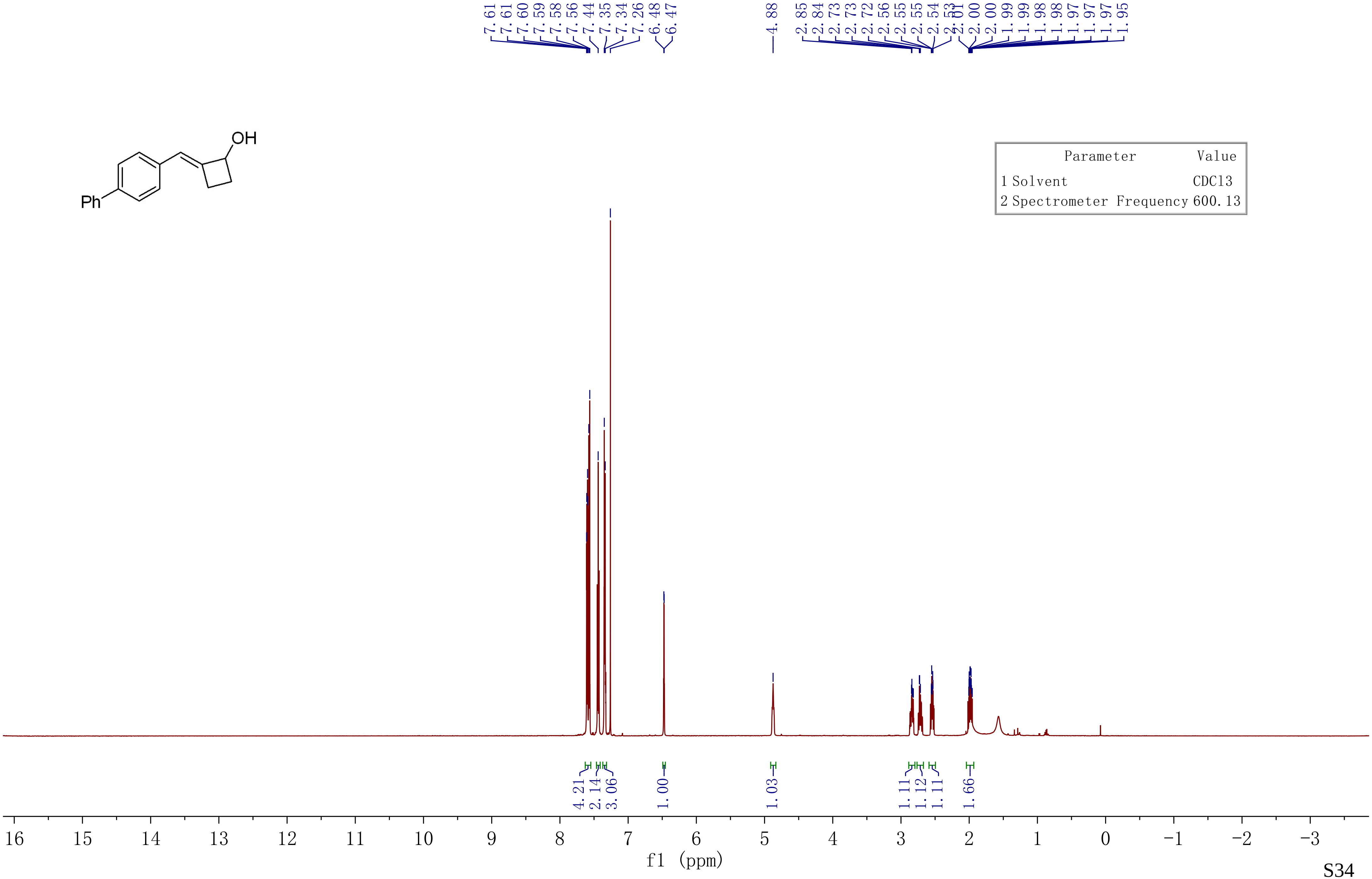

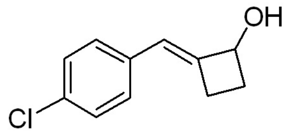

Parameter	Value
1 Solvent	CDCl3
2 Spectrometer Frequency	600.13

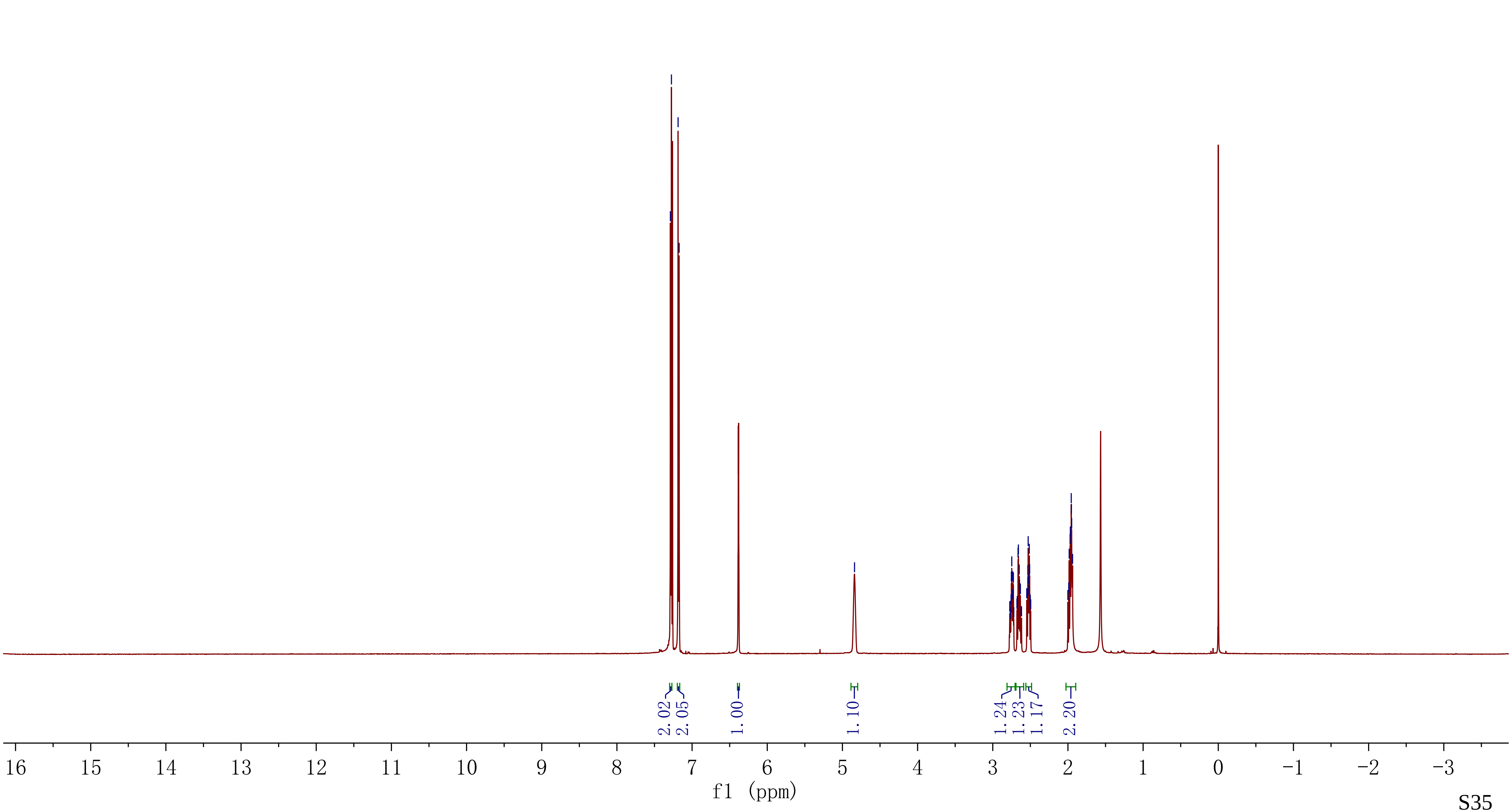

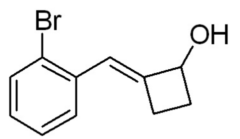

	Parameter	Value
1	Solvent	CDCl3
2	Spectrometer Frequency	600.13

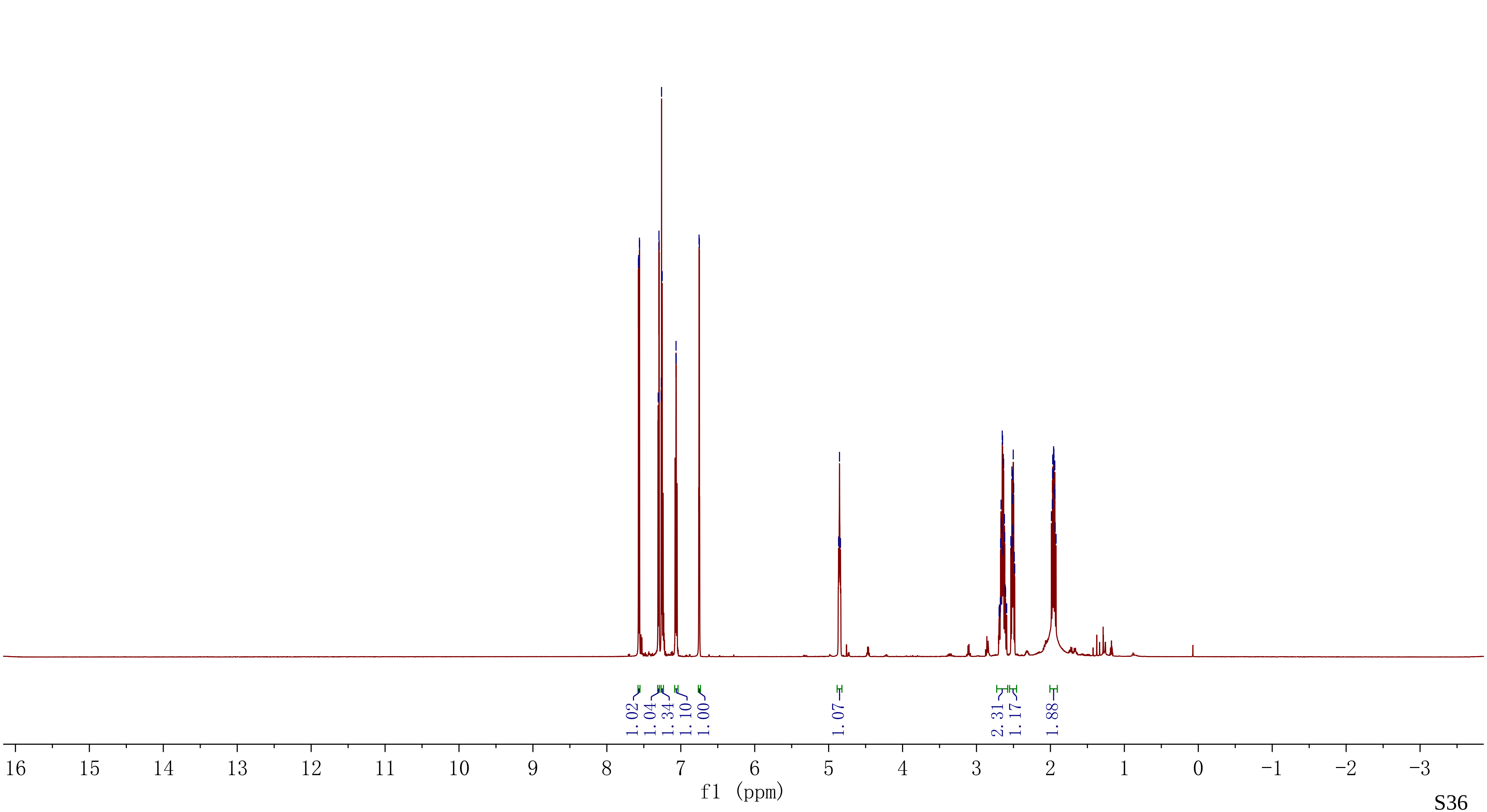

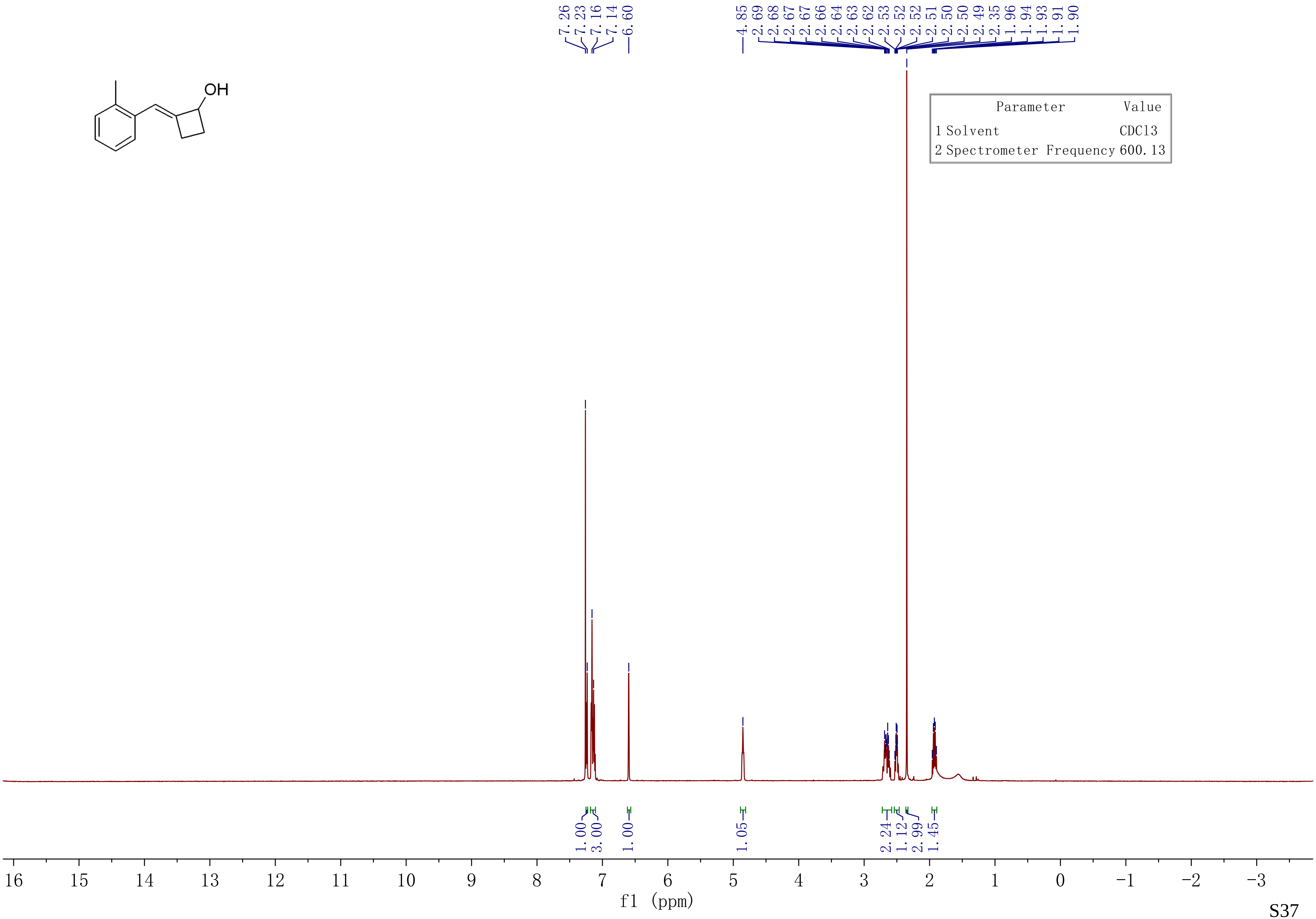
OH
7.26
7.23
7.16
7.14
6.60
4.85
2.69
2.68
2.67
2.67
2.66
2.64
2.63
2.62
2.53
2.52
2.52
2.51
2.50
2.50
2.49
2.35
1.96
1.94
1.93
1.91
1.90
Parameter Value
1 Solvent CDCl3
2 Spectrometer Frequency 600.13
1.00
3.00
1.00
1.05
2.24
1.12
2.99
1.45
16 15 14 13 12 11 10 9 8 7 6 5 4 3 2 1 0 −1 −2 −3
f1 (ppm)

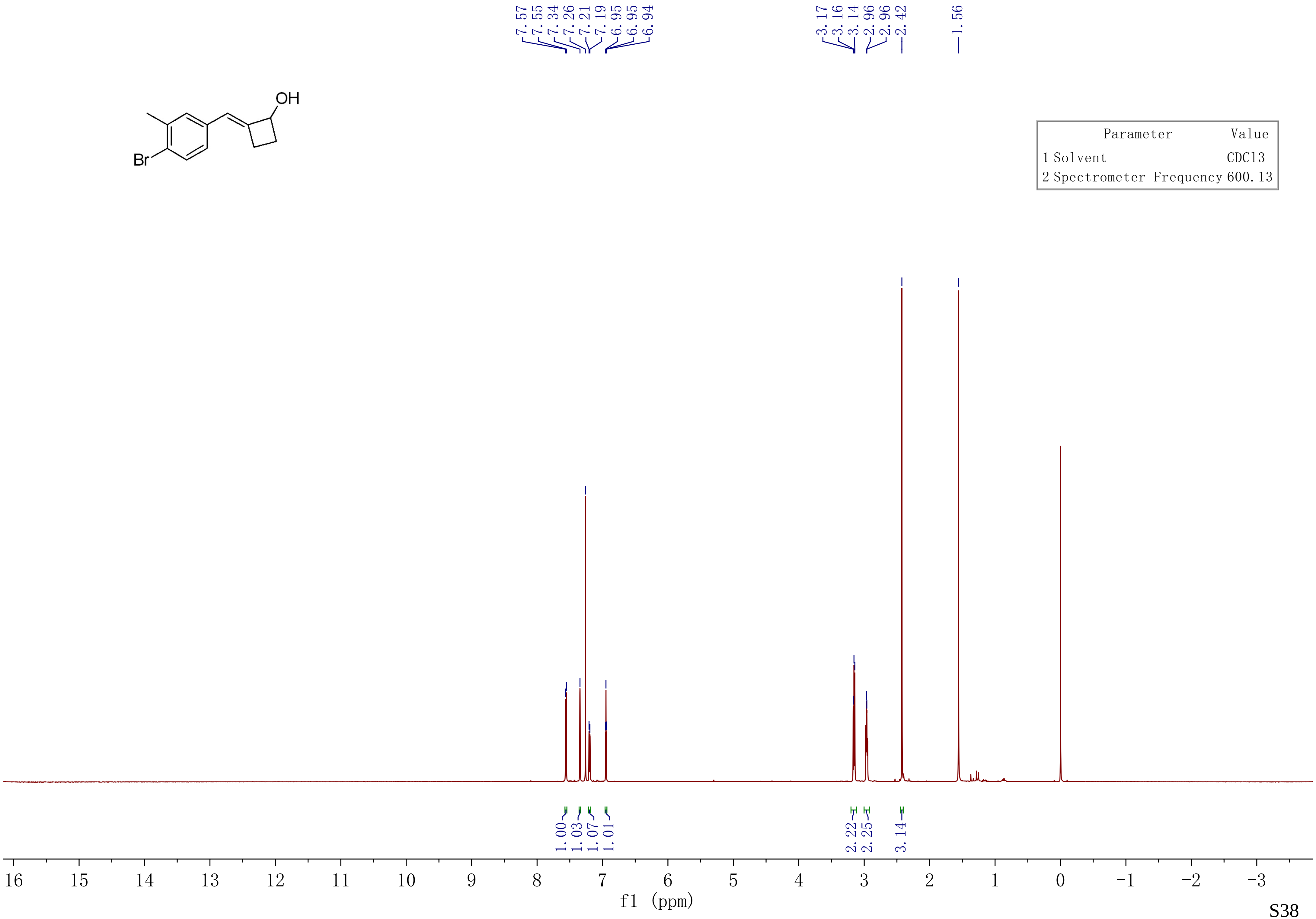
7.57
7.55
7.34
7.26
7.21
7.19
6.95
6.95
6.94
3.17
3.16
3.14
2.96
2.96
2.42
1.56
OH
Br
Parameter Value
1 Solvent CDCl3
2 Spectrometer Frequency 600.13
1.00
1.03
1.07
1.01
2.22
2.25
3.14
16 15 14 13 12 11 10 9 8 7 6 5 4 3 2 1 0 −1 −2 −3
f1 (ppm)

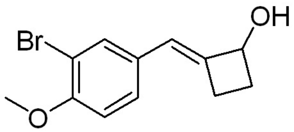

Parameter	Value
1 Solvent	CDCl3
2 Spectrometer Frequency	600.13

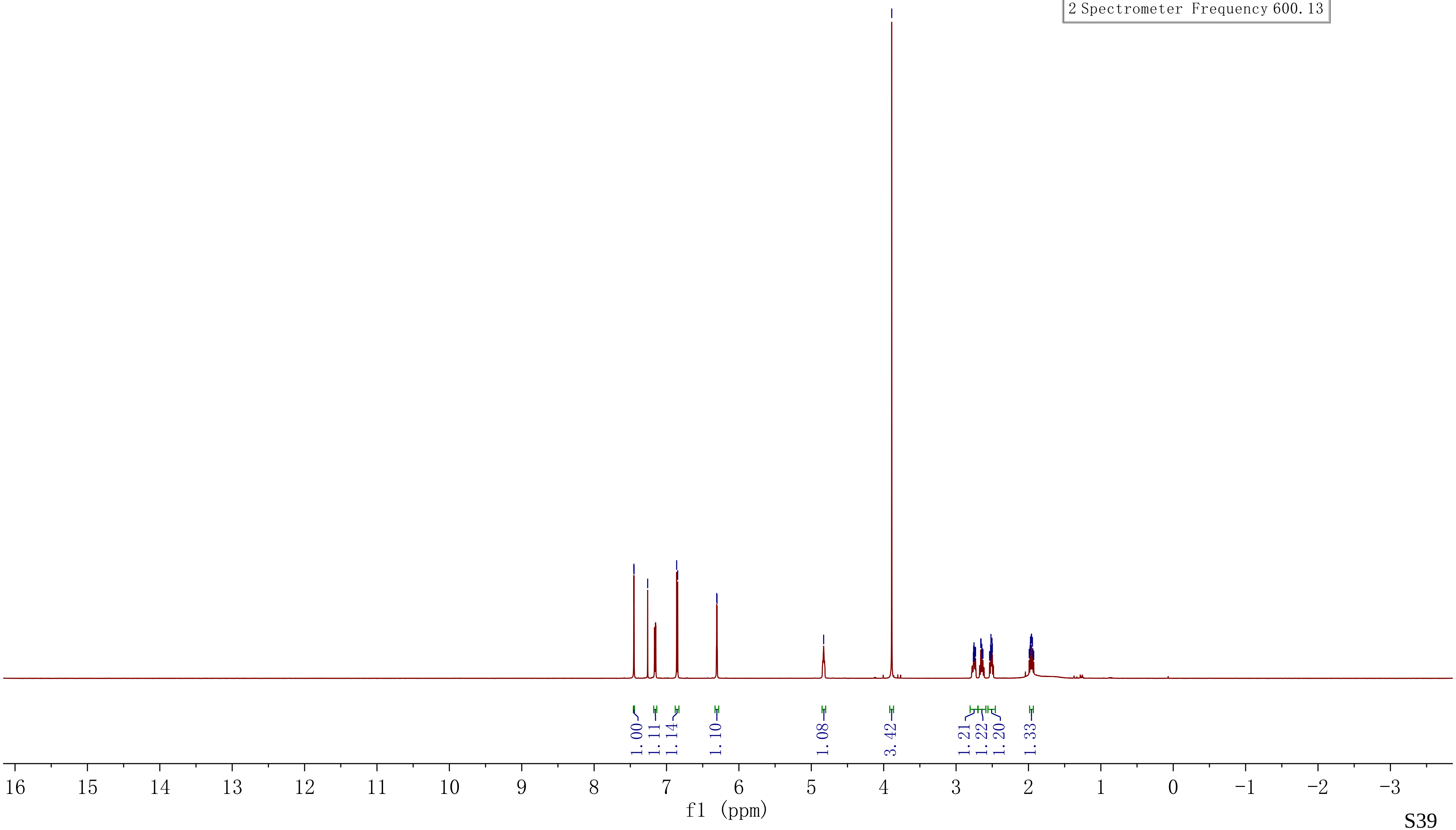

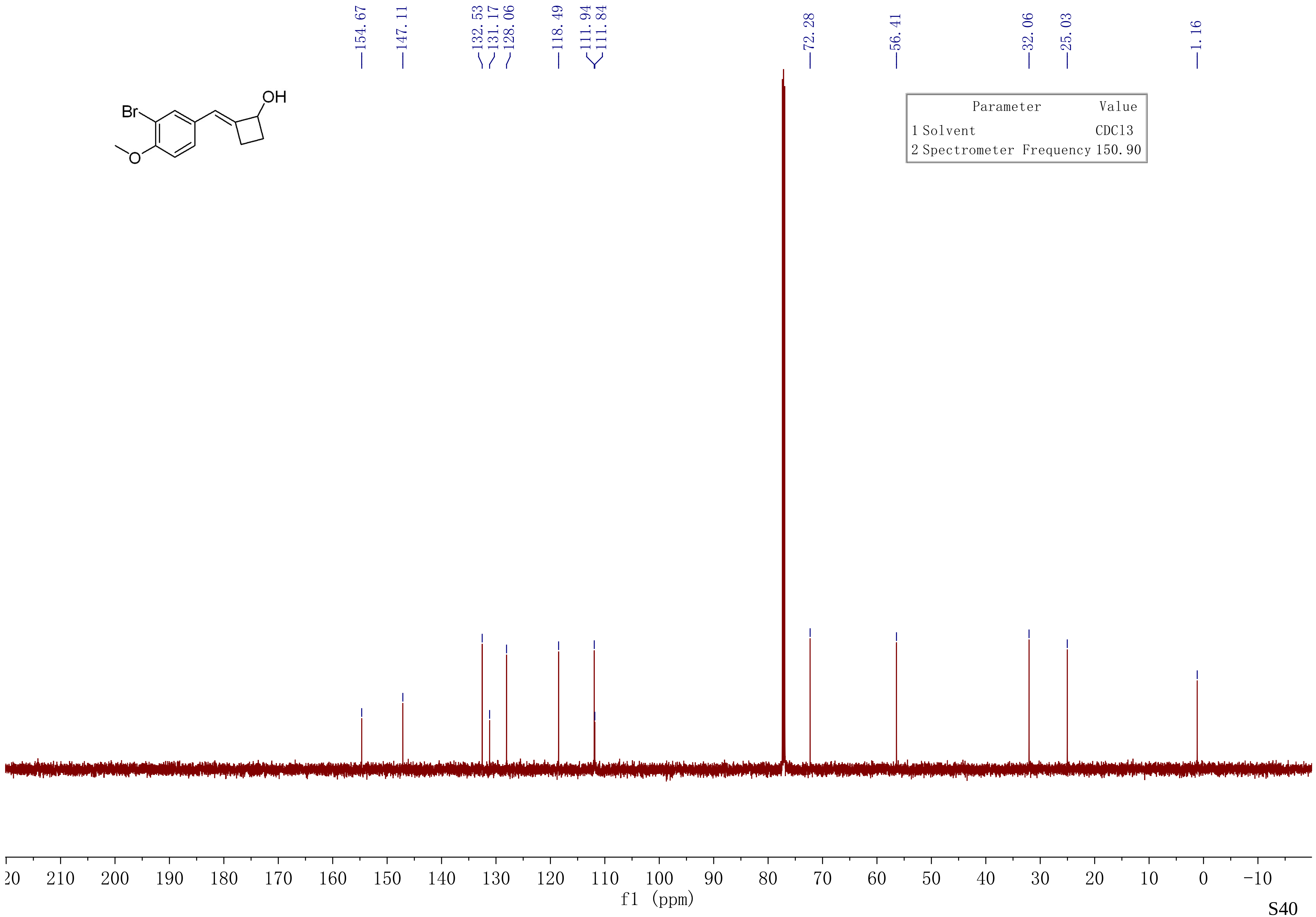
154.67
147.11
132.53
131.17
128.06
118.49
111.94
111.84
72.28
56.41
32.06
25.03
1.16
Br
OH
O
Parameter Value
1 Solvent CDCl3
2 Spectrometer Frequency 150.90
f1 (ppm)

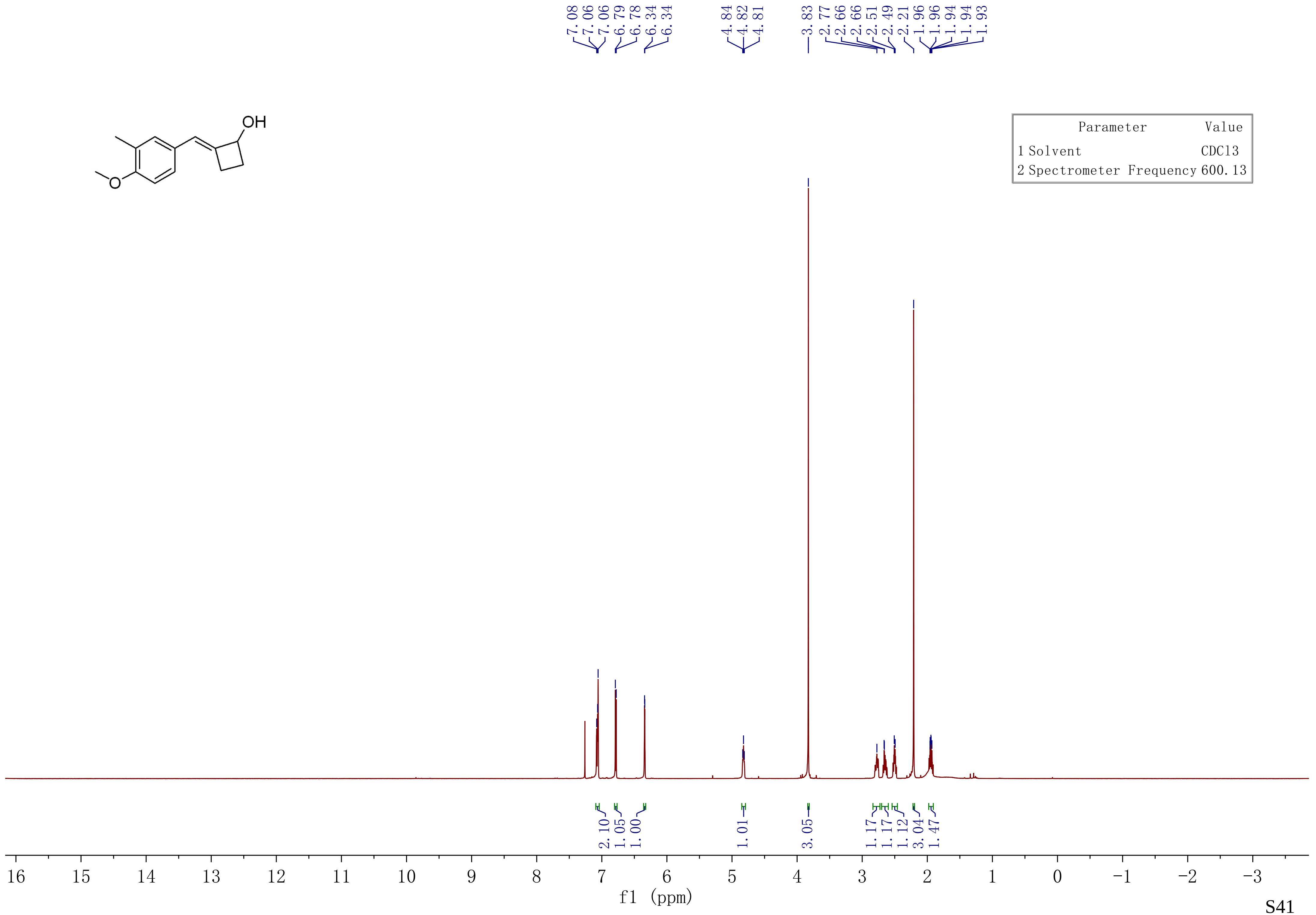
OH
O
7.08
7.06
7.06
6.79
6.78
6.34
6.34
4.84
4.82
4.81
3.83
2.77
2.66
2.66
2.51
2.49
2.21
1.96
1.96
1.94
1.94
1.93
Parameter Value
1 Solvent CDCl3
2 Spectrometer Frequency 600.13
2.10
1.05
1.00
1.01
3.05
1.17
1.17
1.12
3.04
1.47
16 15 14 13 12 11 10 9 8 7 6 5 4 3 2 1 0 -1 -2 -3
f1 (ppm)

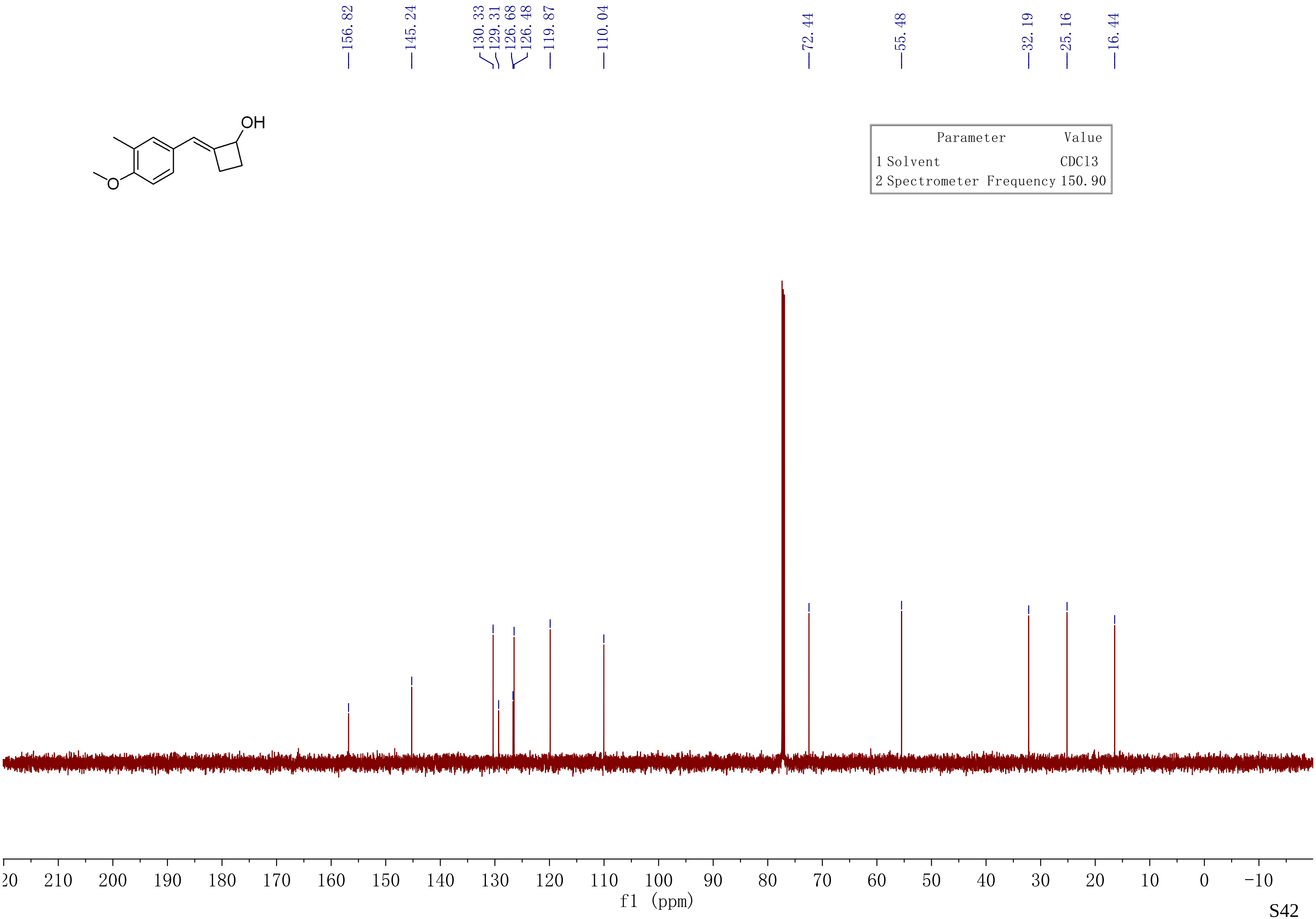
156.82
145.24
130.33
129.31
126.68
126.48
119.87
110.04
72.44
55.48
32.19
25.16
16.44
OH
O
Parameter Value
1 Solvent CDCl3
2 Spectrometer Frequency 150.90
220 210 200 190 180 170 160 150 140 130 120 110 100 90 80 70 60 50 40 30 20 10 0 −10
f1 (ppm)

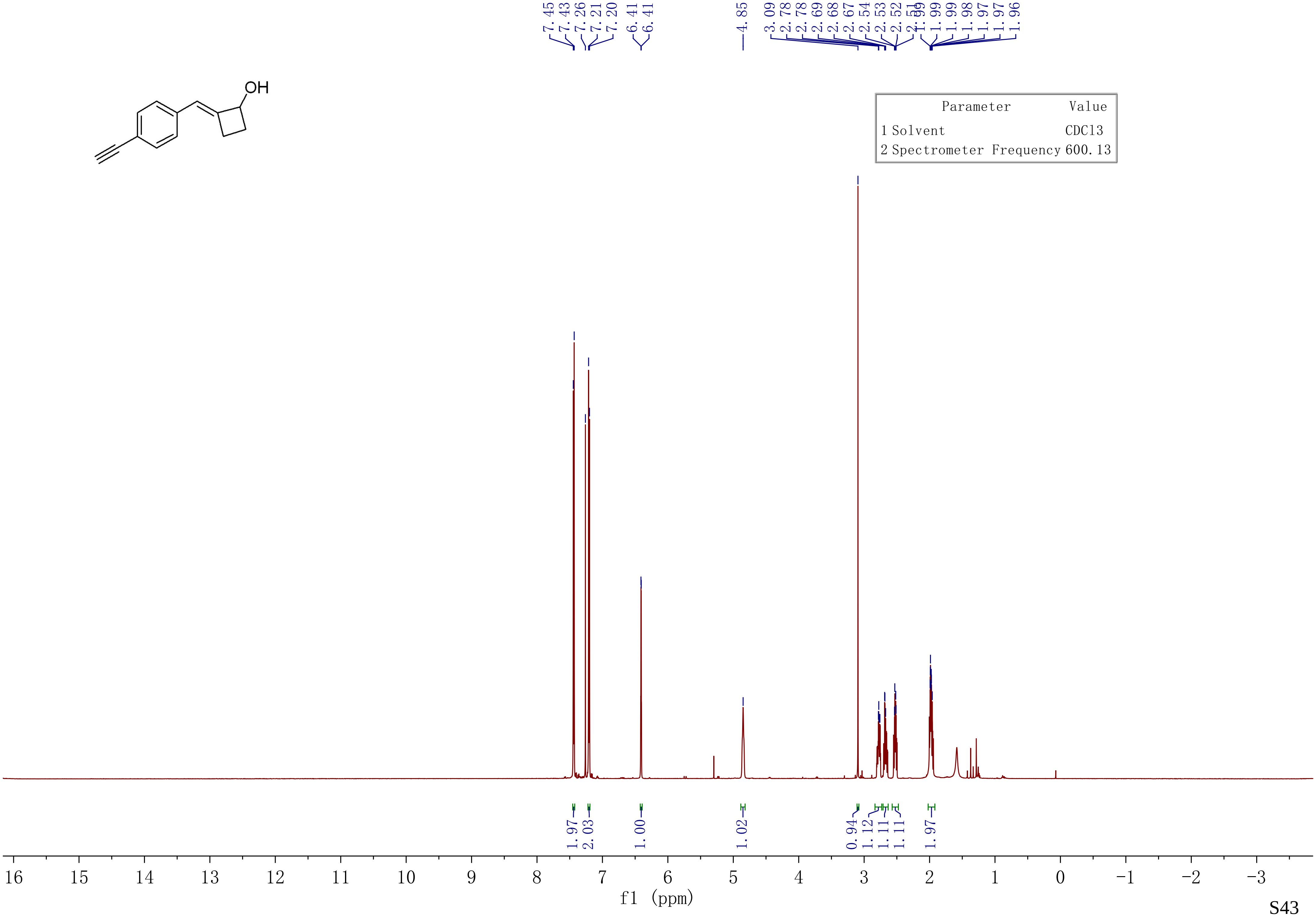
OH
7.45
7.43
7.26
7.21
7.20
6.41
6.41
4.85
3.09
2.78
2.78
2.69
2.68
2.67
2.54
2.53
2.52
1.99
1.99
1.98
1.97
1.97
1.96
Parameter Value
1 Solvent CDCl3
2 Spectrometer Frequency 600.13
1.97
2.03
1.00
1.02
0.94
1.12
1.11
1.11
1.97
16 15 14 13 12 11 10 9 8 7 6 5 4 3 2 1 0 −1 −2 −3
f1 (ppm)

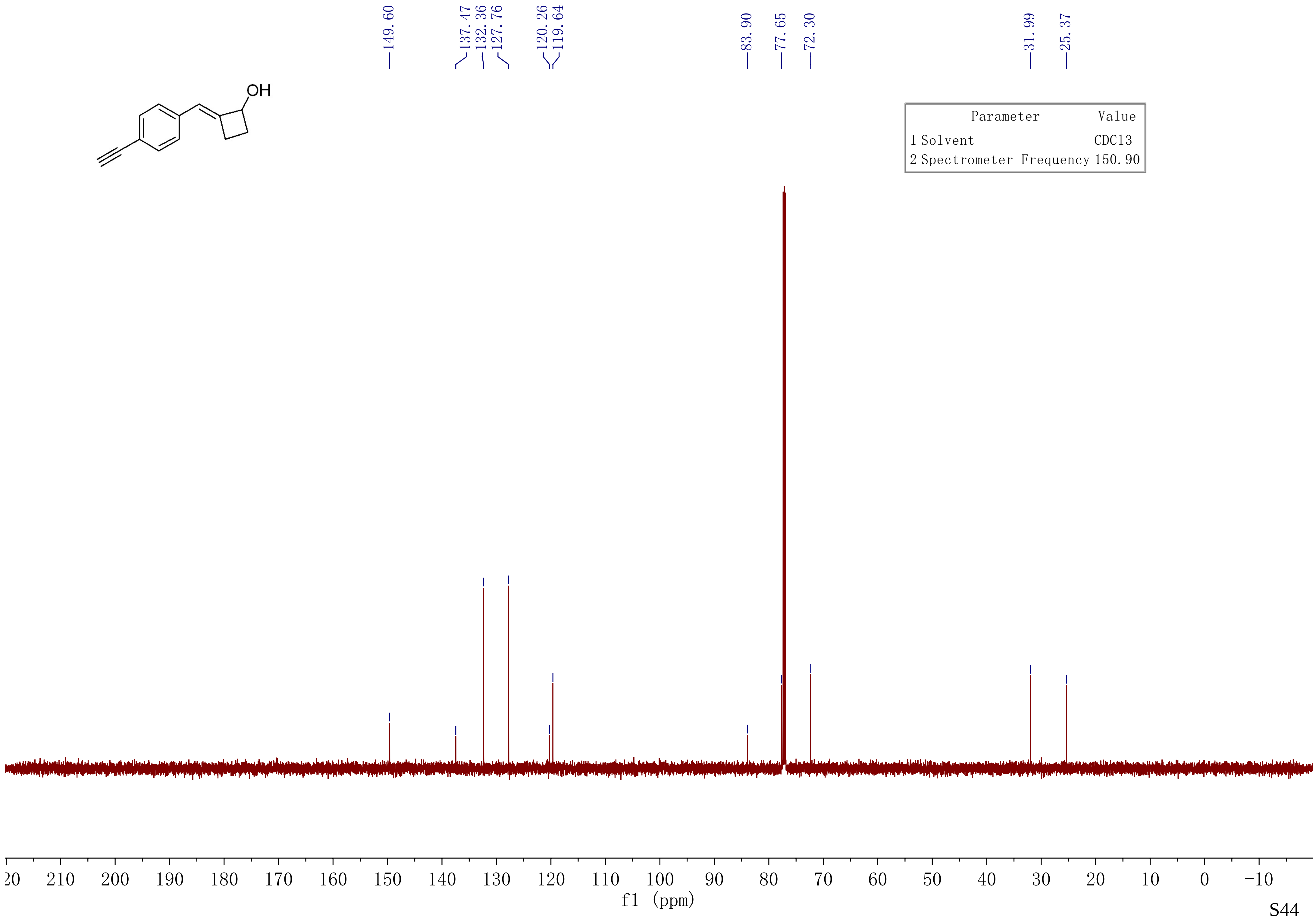

149.60
137.47
132.36
127.76
120.26
119.64
83.90
77.65
72.30
31.99
25.37
OH
Parameter Value
1 Solvent CDCl3
2 Spectrometer Frequency 150.90
20 210 200 190 180 170 160 150 140 130 120 110 100 90 80 70 60 50 40 30 20 10 0 −10
f1 (ppm)

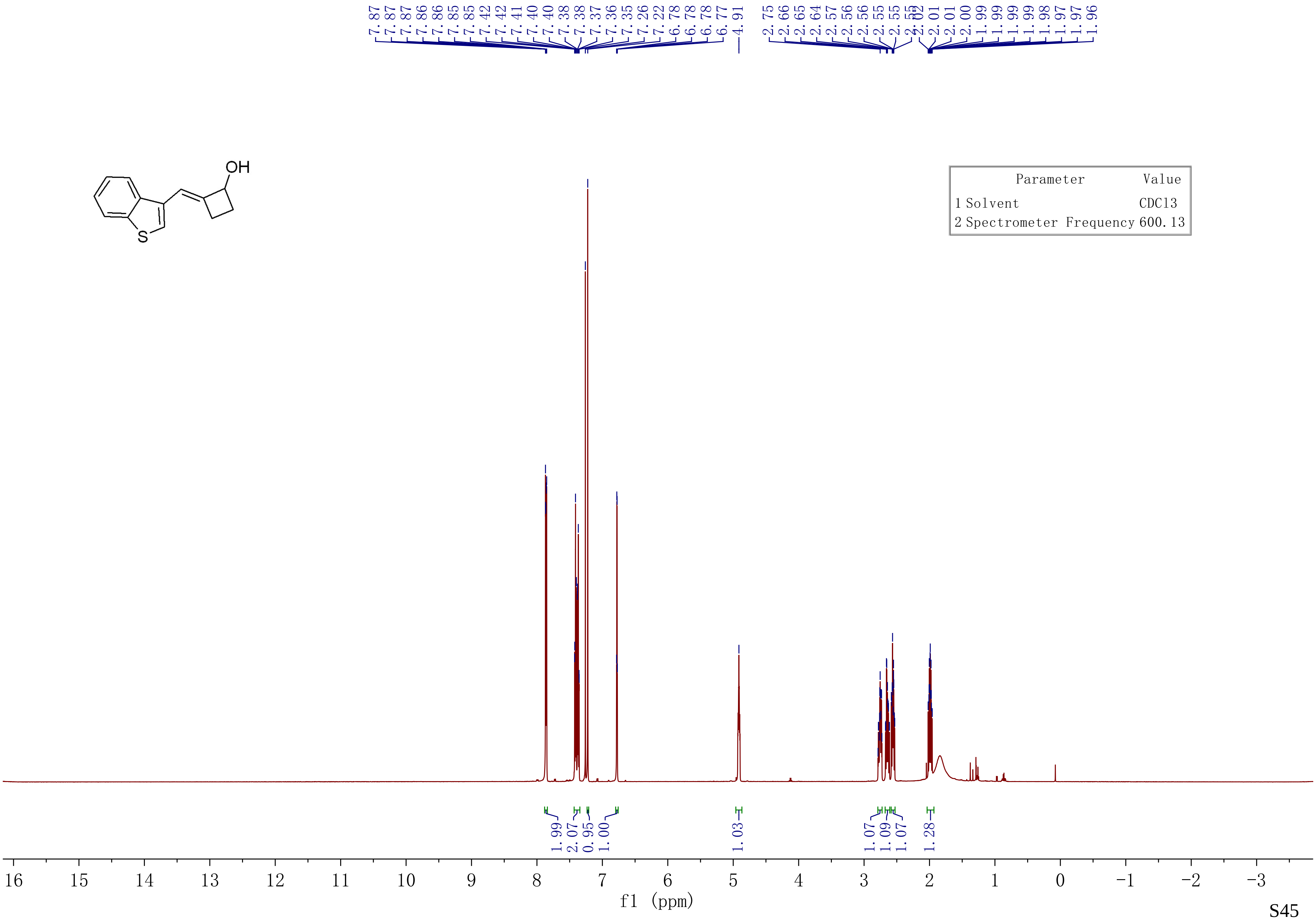

OH
S
7.87
7.87
7.87
7.86
7.86
7.85
7.85
7.42
7.42
7.41
7.40
7.40
7.38
7.38
7.37
7.36
7.35
7.26
7.22
6.78
6.78
6.78
6.77
4.91
2.75
2.66
2.65
2.64
2.57
2.56
2.56
2.55
2.55
2.02
2.01
2.01
2.00
1.99
1.99
1.99
1.99
1.98
1.97
1.97
1.96
Parameter Value
1 Solvent CDCl3
2 Spectrometer Frequency 600.13
1.99
2.07
0.95
1.00
1.03
1.07
1.09
1.07
1.28
f1 (ppm)

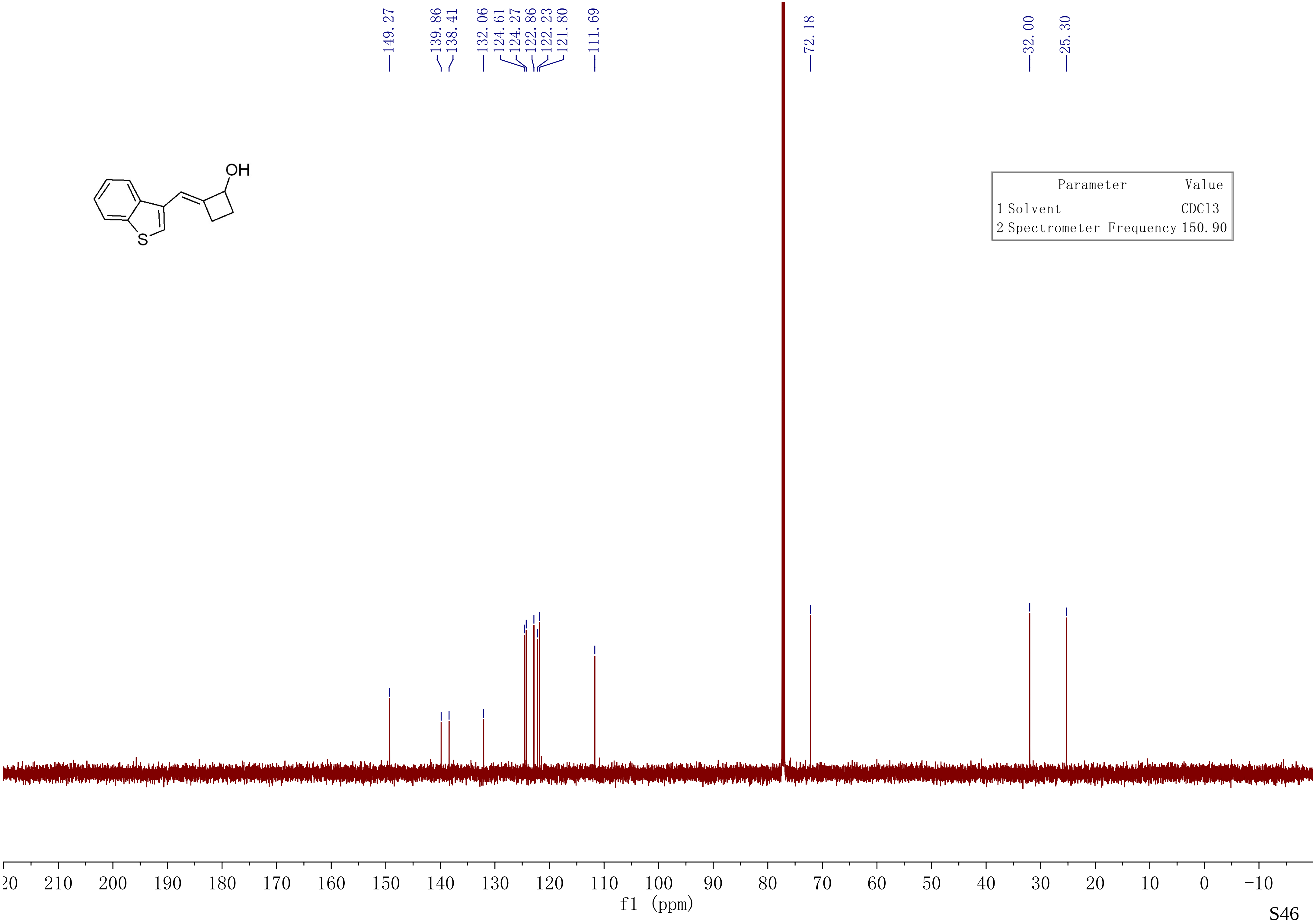
149.27
139.86
138.41
132.06
124.61
124.27
122.86
122.23
121.80
111.69
72.18
32.00
25.30
OH
S
Parameter Value
1 Solvent CDCl3
2 Spectrometer Frequency 150.90
20 210 200 190 180 170 160 150 140 130 120 110 100 90 80 70 60 50 40 30 20 10 0 −10
f1 (ppm)

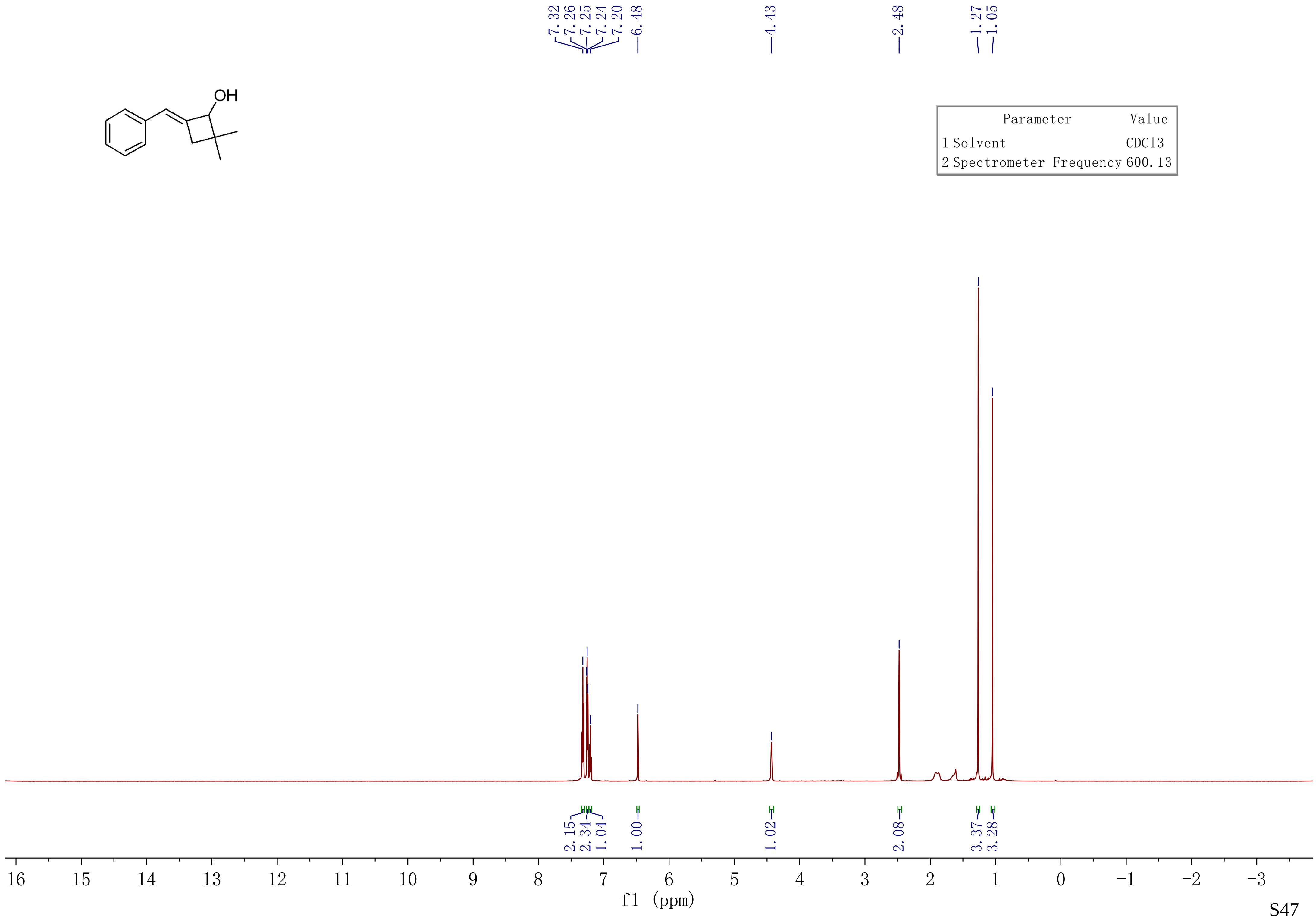
7.32
7.26
7.25
7.24
7.20
6.48
4.43
2.48
1.27
1.05
OH
Parameter Value
1 Solvent CDCl3
2 Spectrometer Frequency 600.13
2.15
2.34
1.04
1.00
1.02
2.08
3.37
3.28
16 15 14 13 12 11 10 9 8 7 6 5 4 3 2 1 0 −1 −2 −3
f1 (ppm)

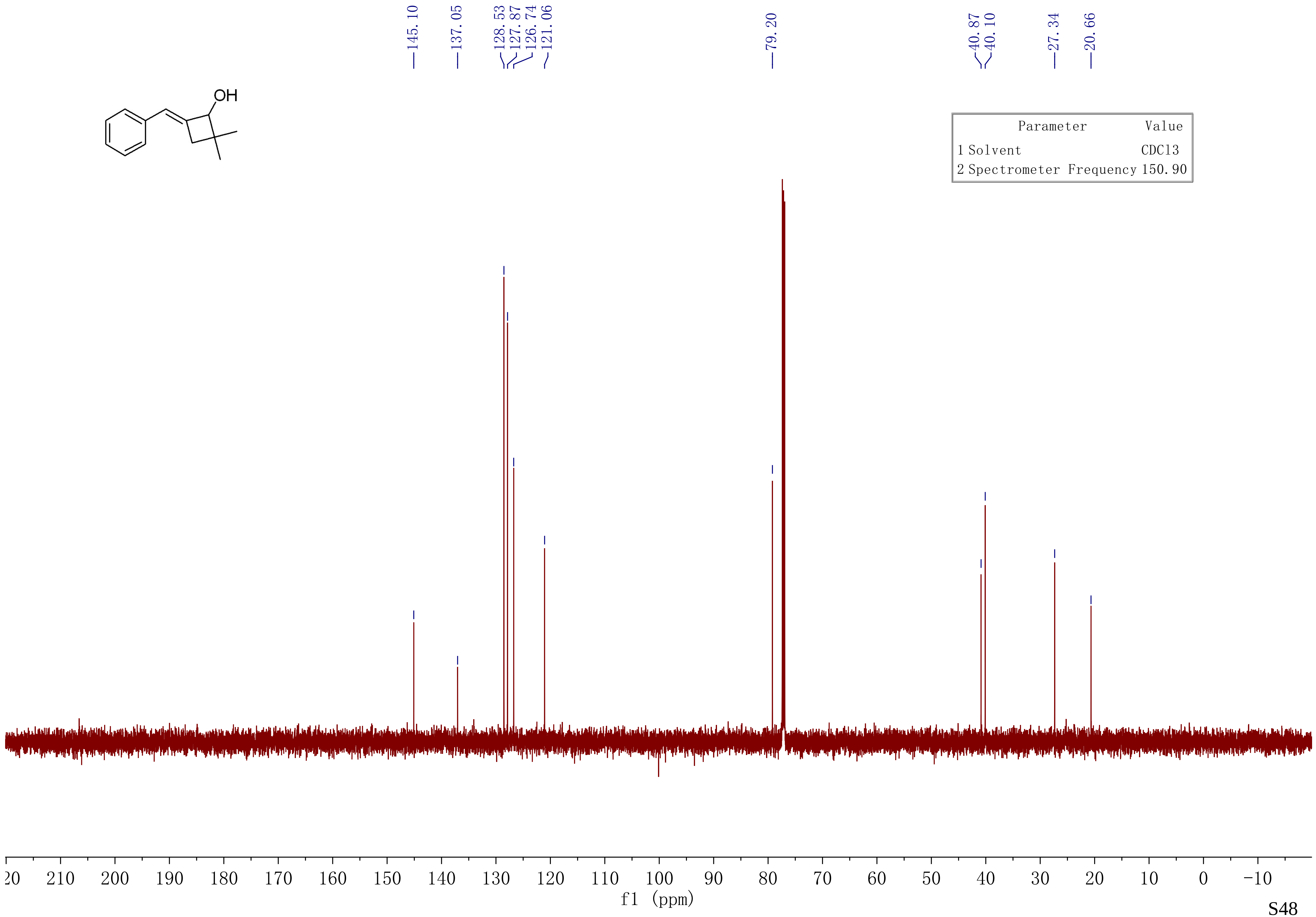

	Parameter	Value
1	Solvent	CDCl3
2	Spectrometer Frequency	150.90

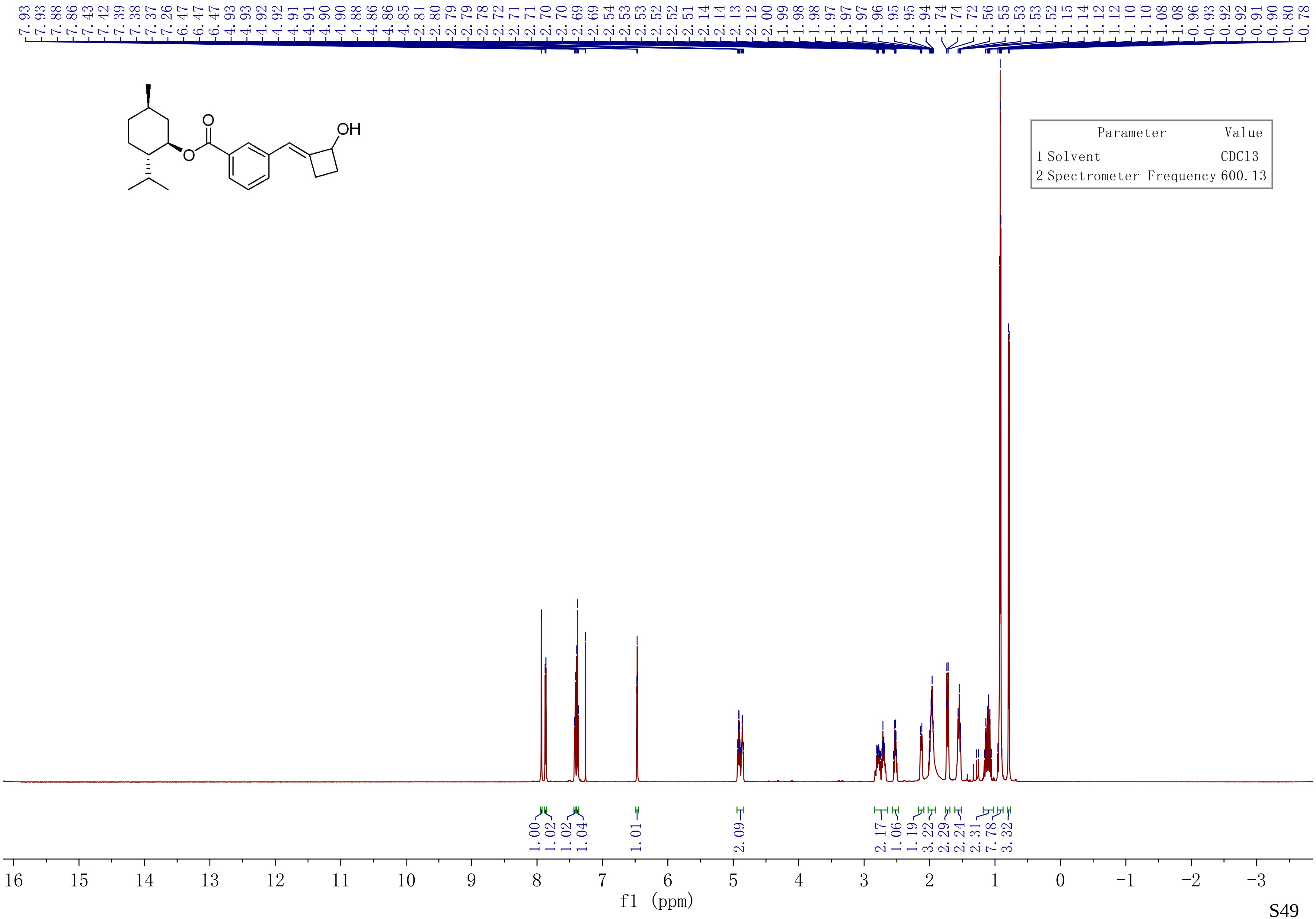
7.93
7.93
7.88
7.86
7.43
7.42
7.39
7.38
7.37
7.26
6.47
6.47
6.47
4.93
4.93
4.92
4.92
4.91
4.91
4.90
4.90
4.88
4.86
4.86
4.85
2.81
2.80
2.79
2.79
2.78
2.72
2.71
2.71
2.70
2.70
2.69
2.69
2.54
2.53
2.53
2.52
2.52
2.51
2.14
2.14
2.13
2.12
2.00
1.99
1.98
1.98
1.97
1.97
1.97
1.96
1.95
1.95
1.94
1.74
1.74
1.72
1.56
1.55
1.53
1.53
1.52
1.15
1.14
1.12
1.12
1.10
1.10
1.08
1.08
0.96
0.93
0.92
0.92
0.91
0.90
0.80
0.78
O
OH
O
Parameter
Value
1 Solvent
CDCl3
2 Spectrometer Frequency
600.13
1.00
1.02
1.02
1.04
1.01
2.09
2.17
1.06
1.19
3.22
2.29
2.24
2.31
7.78
3.32
16
15
14
13
12
11
10
9
8
7
6
5
4
3
2
1
0
−1
−2
−3
f1 (ppm)

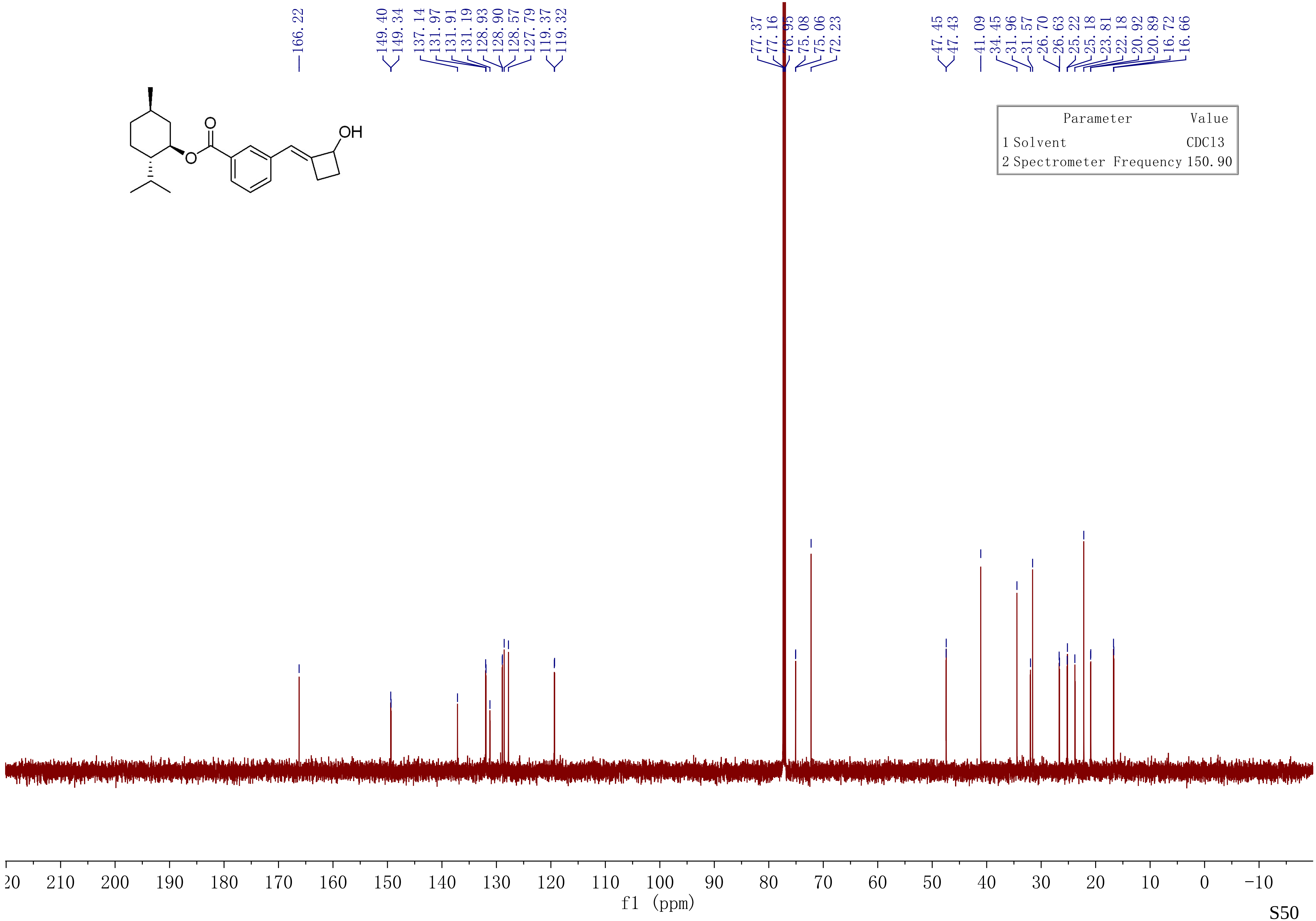
166.22
149.40
149.34
137.14
131.97
131.91
131.19
128.93
128.90
128.57
127.79
119.37
119.32
77.37
77.16
76.95
75.08
75.06
72.23
47.45
47.43
41.09
34.45
31.96
31.57
26.70
26.63
25.22
25.18
23.81
22.18
20.92
20.89
16.72
16.66
OH
O
O
Parameter Value
1 Solvent CDCl3
2 Spectrometer Frequency 150.90
f1 (ppm)

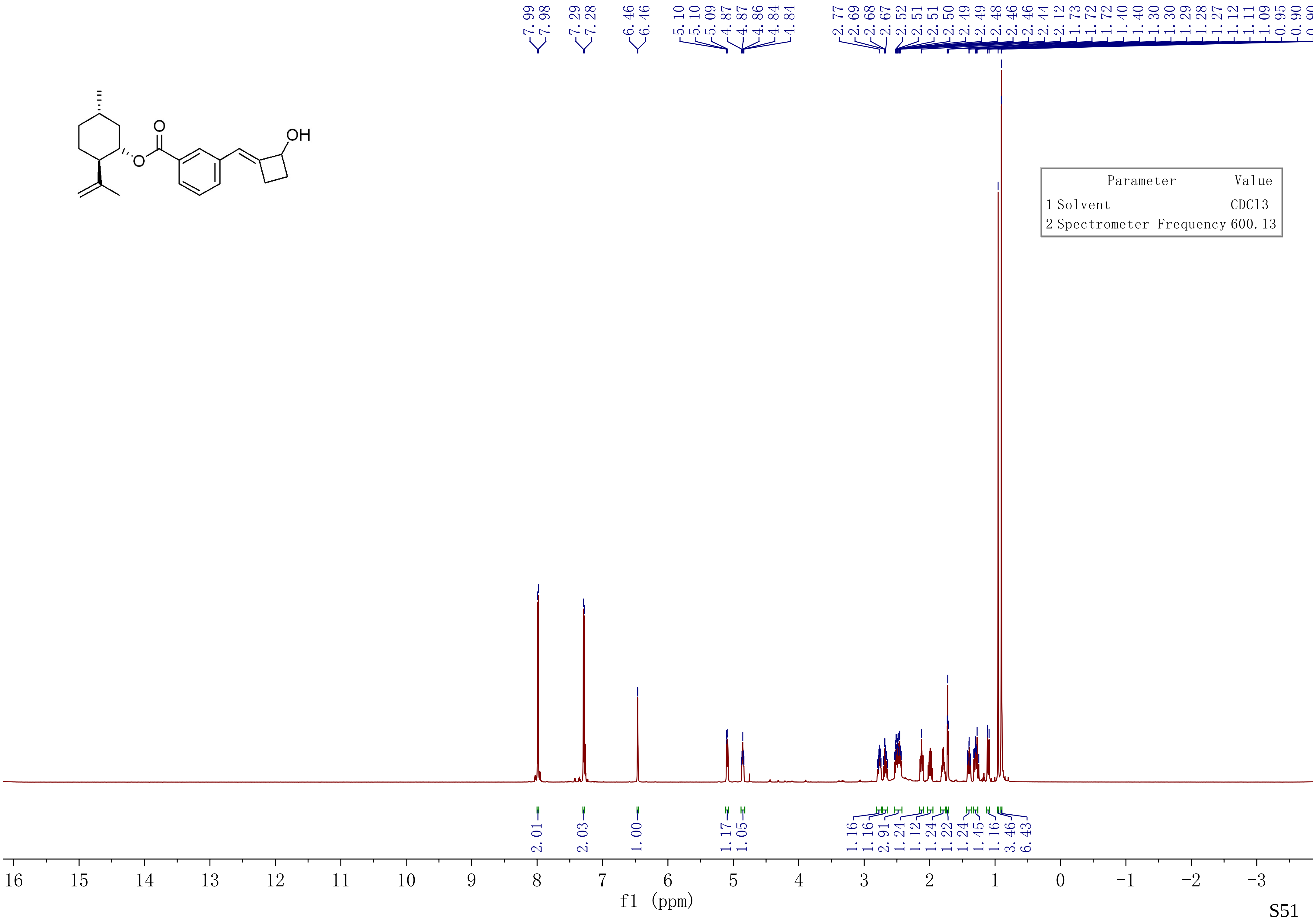
O
OH
O
7.99
7.98
7.29
7.28
6.46
6.46
5.10
5.10
5.09
4.87
4.87
4.86
4.84
4.84
2.77
2.69
2.68
2.67
2.52
2.51
2.51
2.50
2.49
2.49
2.48
2.46
2.46
2.44
2.12
1.73
1.72
1.72
1.40
1.40
1.30
1.30
1.29
1.28
1.27
1.12
1.11
1.09
0.95
0.90
Parameter Value
1 Solvent CDCl3
2 Spectrometer Frequency 600.13
2.01
2.03
1.00
1.17
1.05
1.16
1.16
2.91
1.24
1.12
1.24
1.22
1.24
1.45
1.16
3.46
6.43
16 15 14 13 12 11 10 9 8 7 6 5 4 3 2 1 0 −1 −2 −3
f1 (ppm)

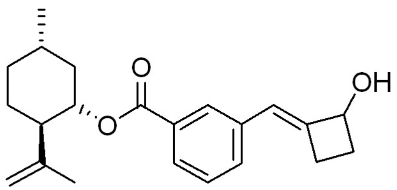

Parameter	Value
1 Solvent	CDCl3
2 Spectrometer Frequency	150.90

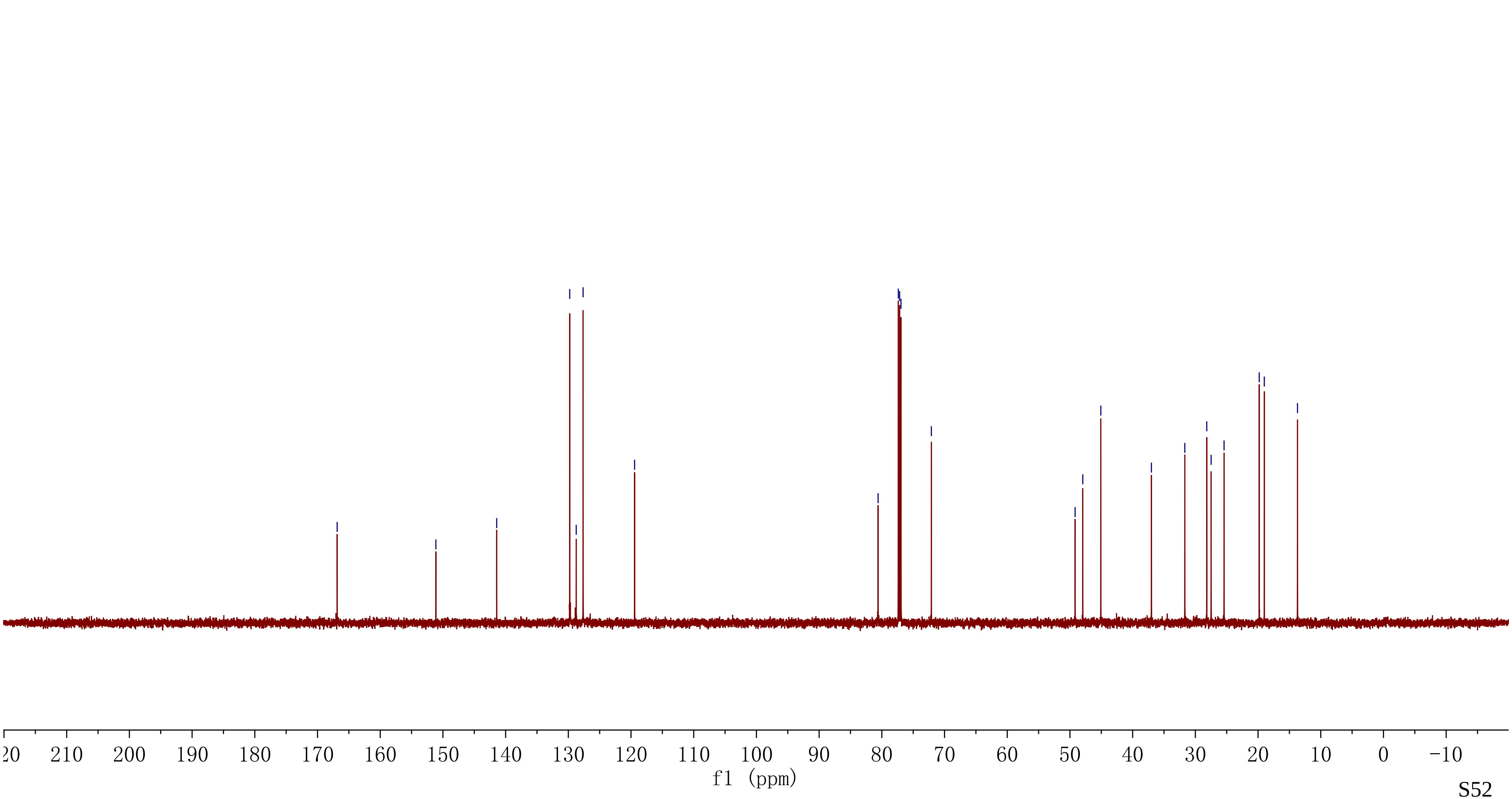

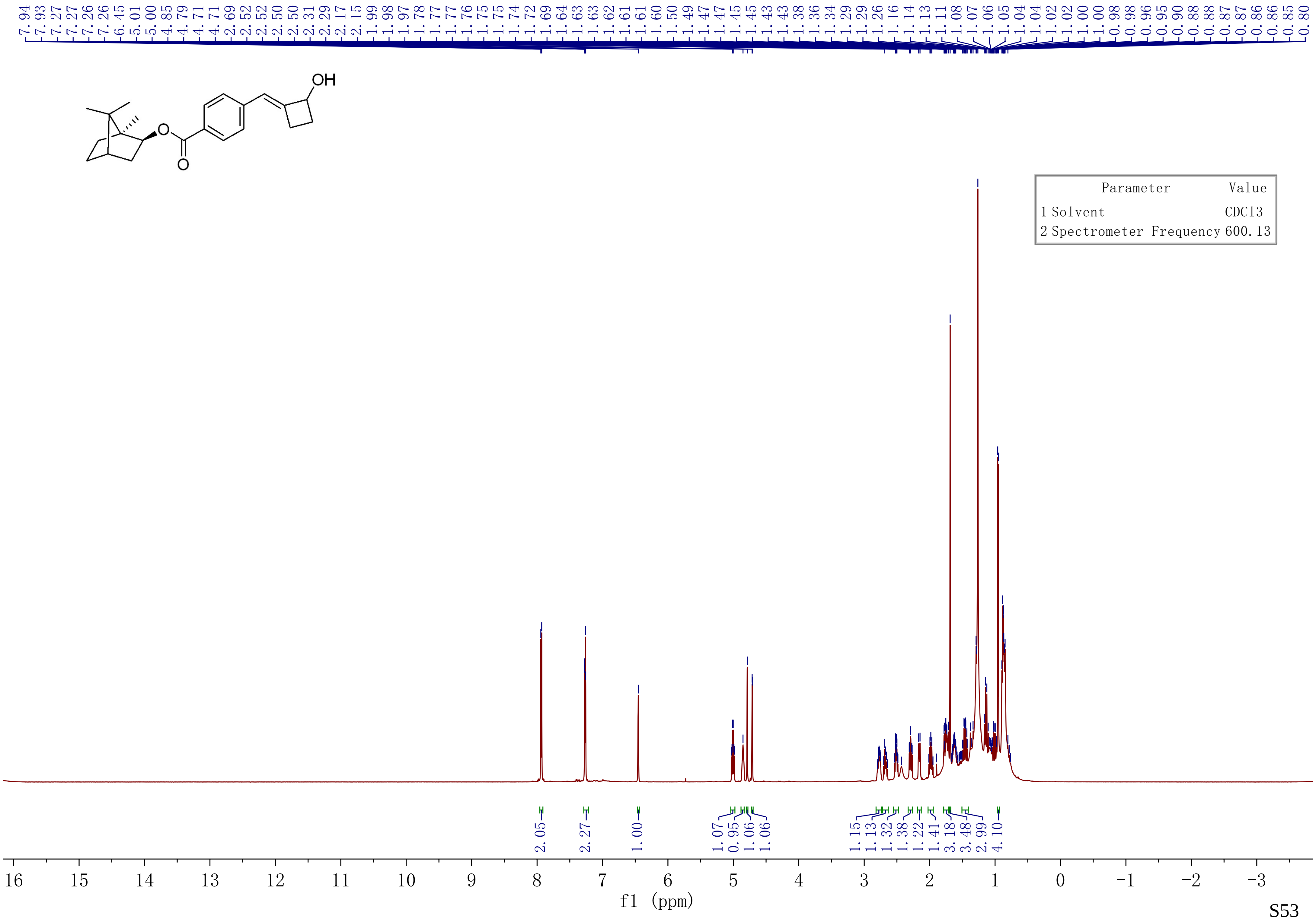

7.94
7.93
7.27
7.27
7.26
7.26
6.45
5.01
5.00
4.85
4.79
4.71
4.71
2.69
2.52
2.52
2.50
2.50
2.31
2.29
2.17
2.15
1.99
1.98
1.97
1.78
1.77
1.77
1.76
1.75
1.75
1.74
1.72
1.69
1.64
1.63
1.63
1.62
1.61
1.61
1.60
1.50
1.49
1.47
1.47
1.45
1.45
1.43
1.43
1.38
1.36
1.34
1.29
1.29
1.26
1.16
1.14
1.13
1.11
1.08
1.07
1.06
1.05
1.04
1.04
1.02
1.02
1.00
1.00
0.98
0.98
0.96
0.95
0.90
0.88
0.87
0.87
0.86
0.86
0.85
0.80
Parameter
Value
1 Solvent
CDCl3
2 Spectrometer Frequency
600.13
2.05
2.27
1.00
1.07
0.95
1.06
1.06
1.15
1.13
1.32
1.38
1.22
1.41
3.18
3.48
2.99
4.10
16
15
14
13
12
11
10
9
8
7
6
5
4
3
2
1
0
−1
−2
−3
f1 (ppm)

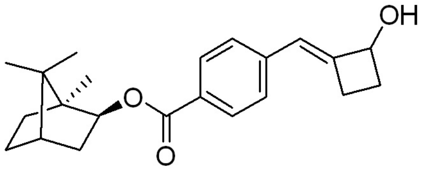

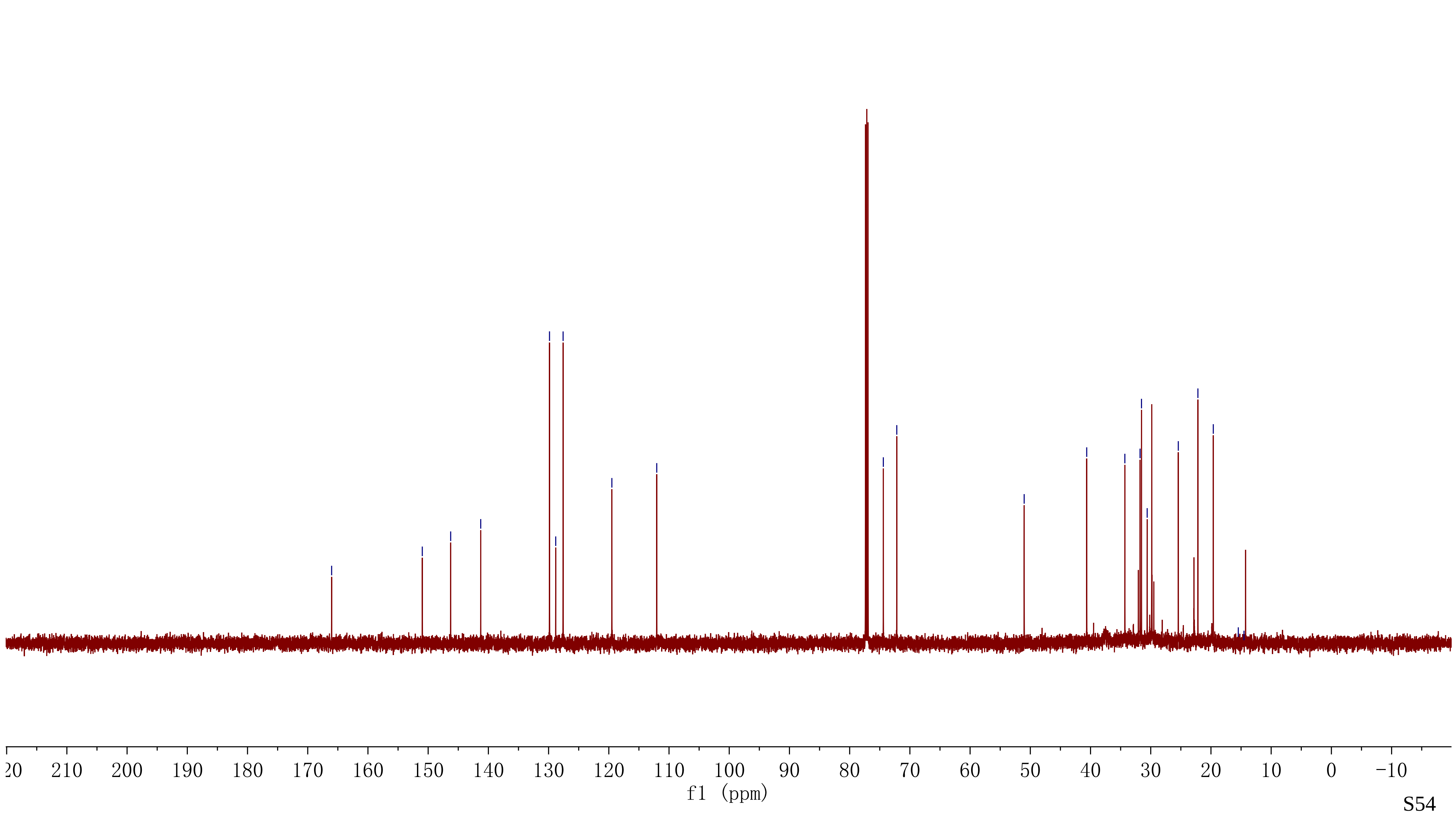

	Parameter	Value
1	Solvent	CDCl3
2	Spectrometer Frequency	150.90

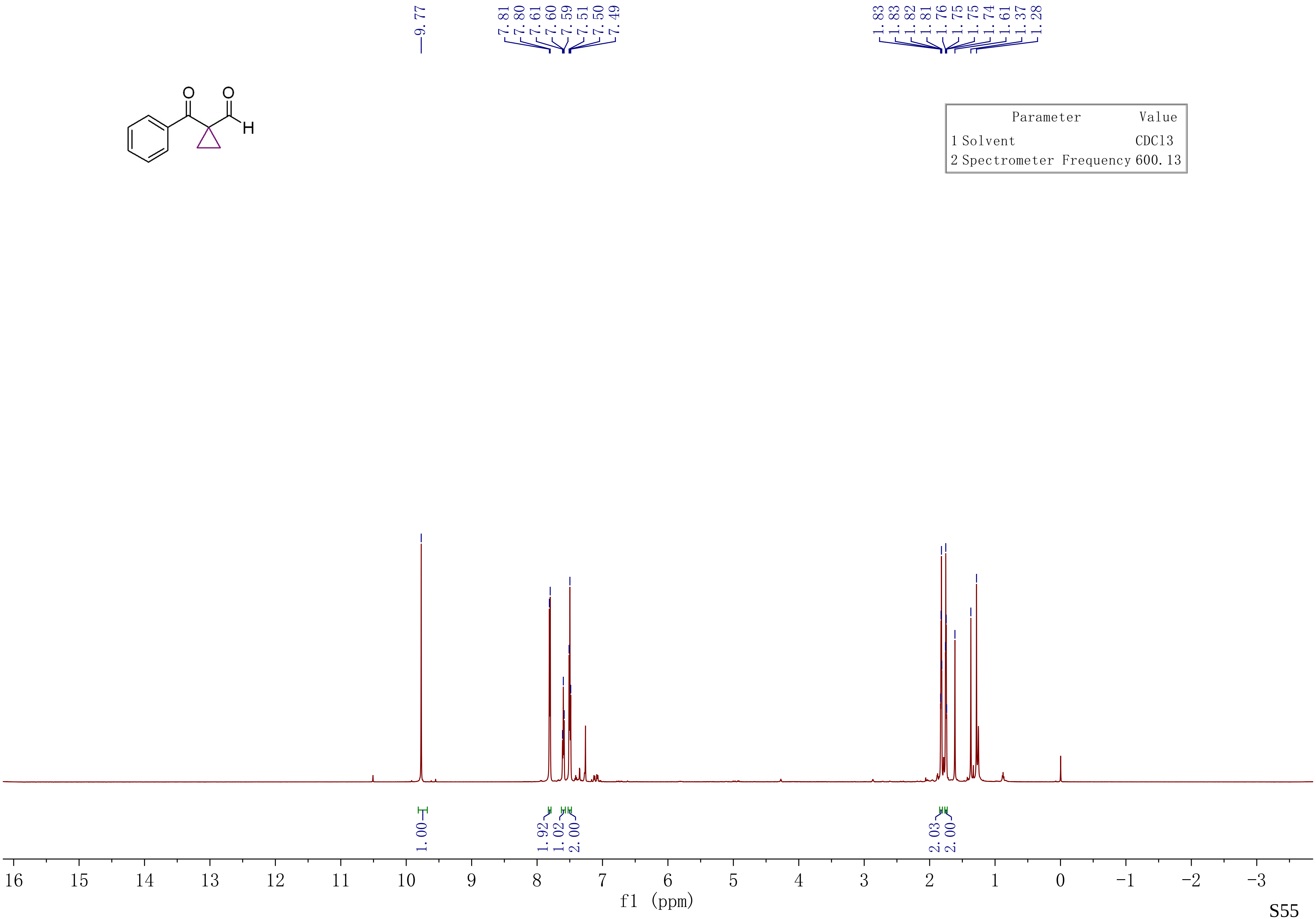
9.77
7.81 7.80 7.61 7.60 7.59 7.51 7.50 7.49
1.83 1.83 1.82 1.81 1.76 1.75 1.75 1.74 1.61 1.37 1.28
O O H
Parameter Value
1 Solvent CDCl3
2 Spectrometer Frequency 600.13
1.00 1.92 1.02 2.00 2.03 2.00
16 15 14 13 12 11 10 9 8 7 6 5 4 3 2 1 0 −1 −2 −3
f1 (ppm)

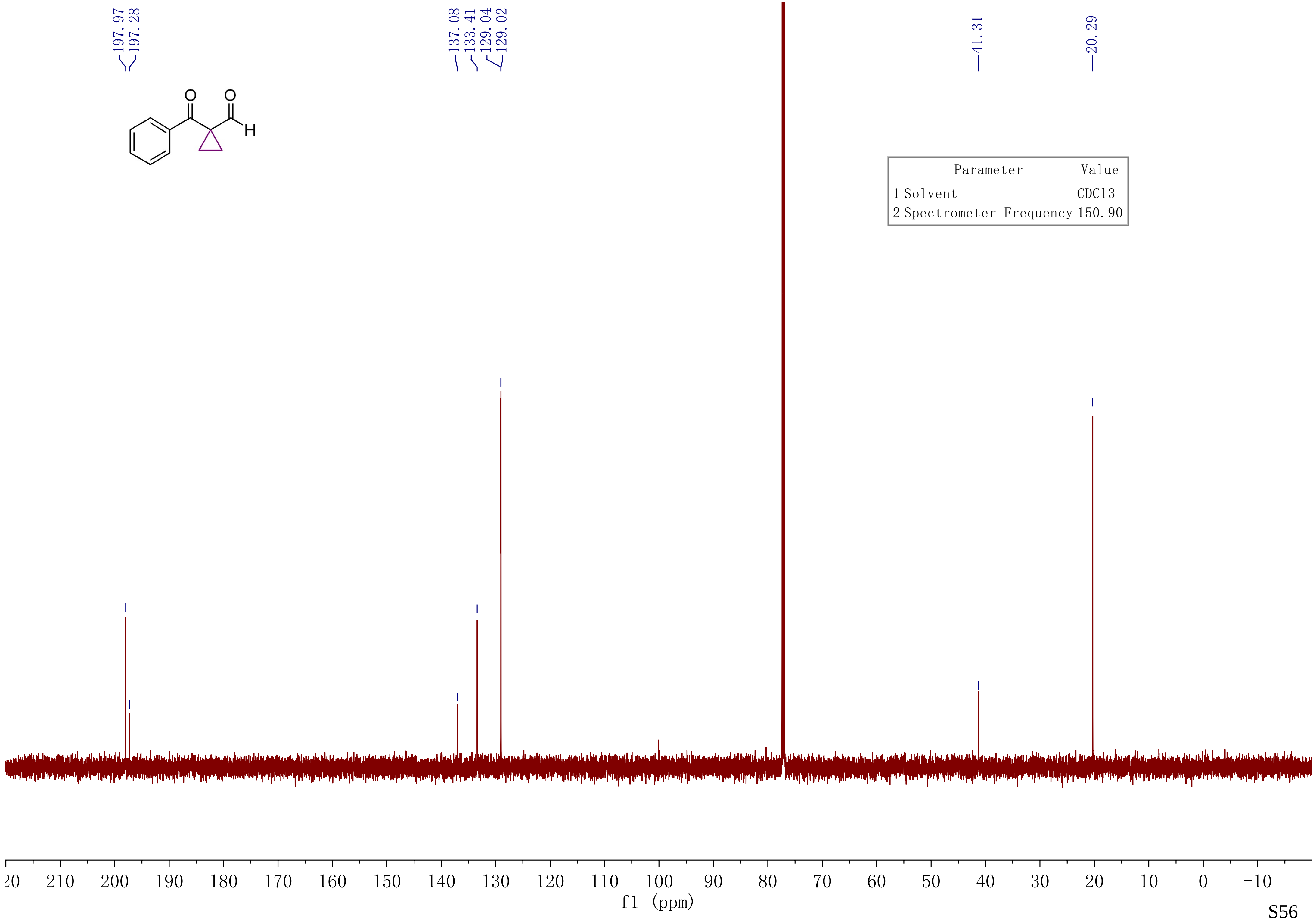
197.97
197.28
137.08
133.41
129.04
129.02
41.31
20.29
Parameter Value
1 Solvent CDCl3
2 Spectrometer Frequency 150.90
f1 (ppm)

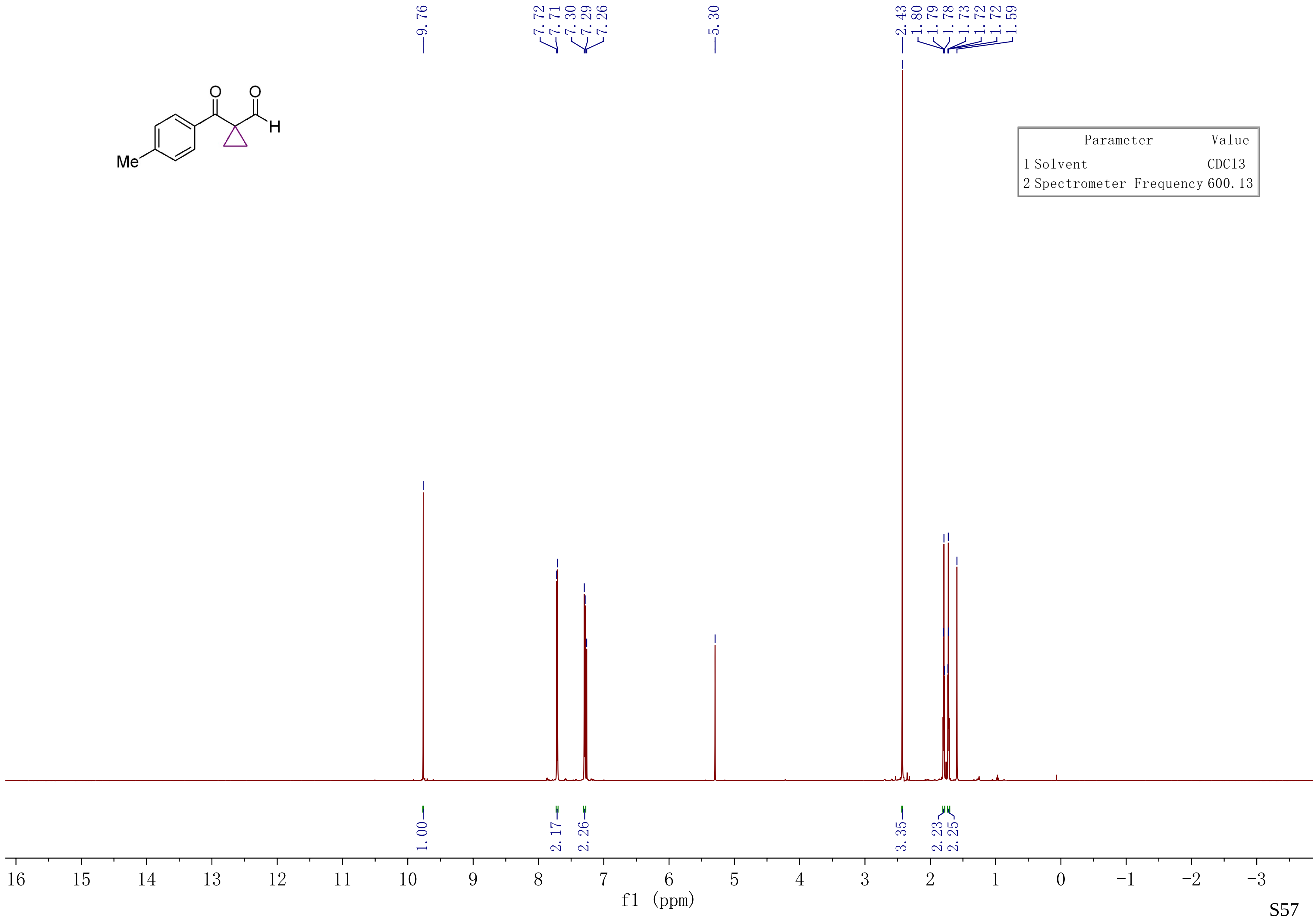

Parameter	Value
1 Solvent	CDCl3
2 Spectrometer Frequency	600.13

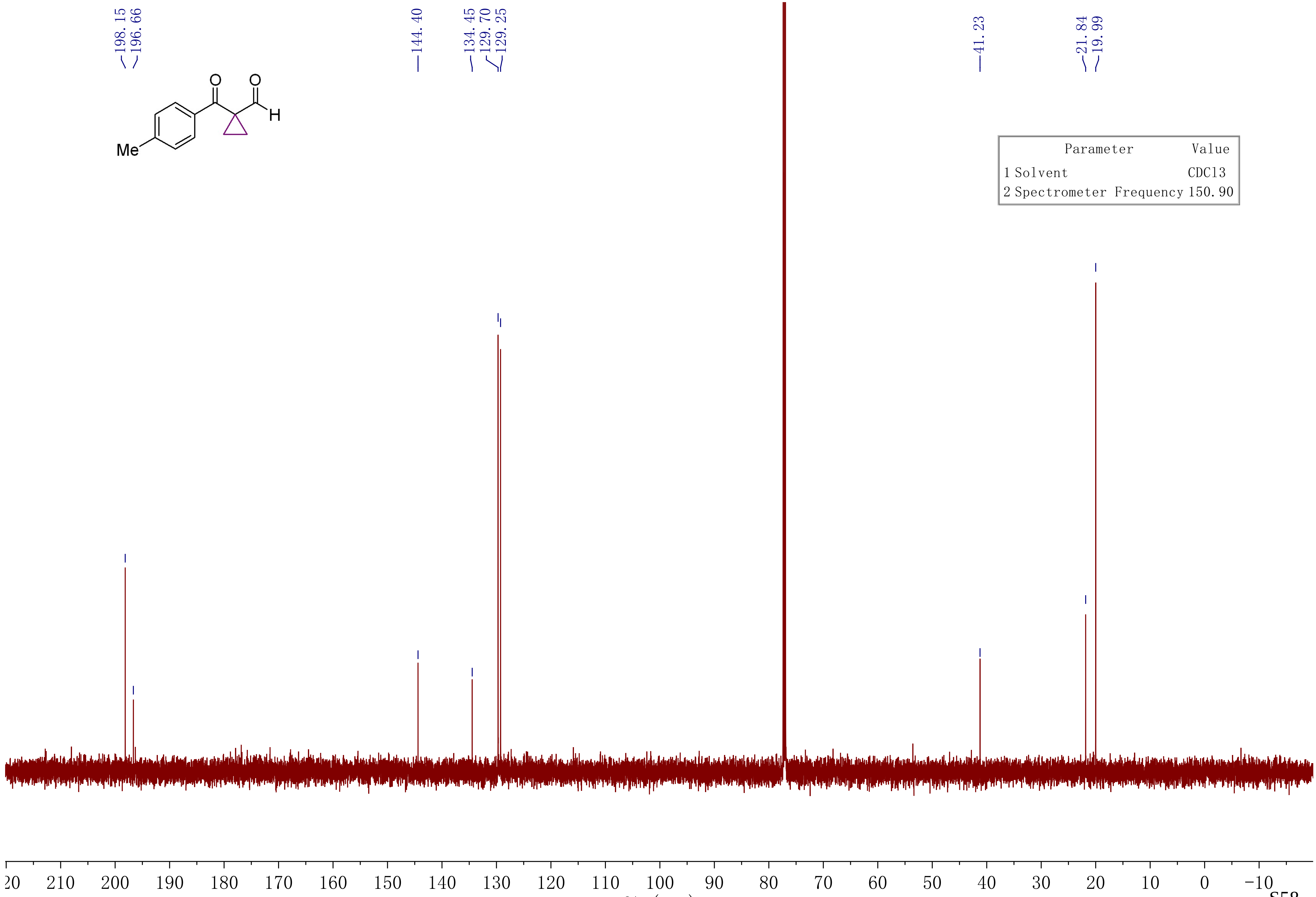

198.15
196.66
144.40
134.45
129.70
129.25
41.23
21.84
19.99
Me
H
Parameter Value
1 Solvent CDCl3
2 Spectrometer Frequency 150.90
220 210 200 190 180 170 160 150 140 130 120 110 100 90 80 70 60 50 40 30 20 10 0 −10

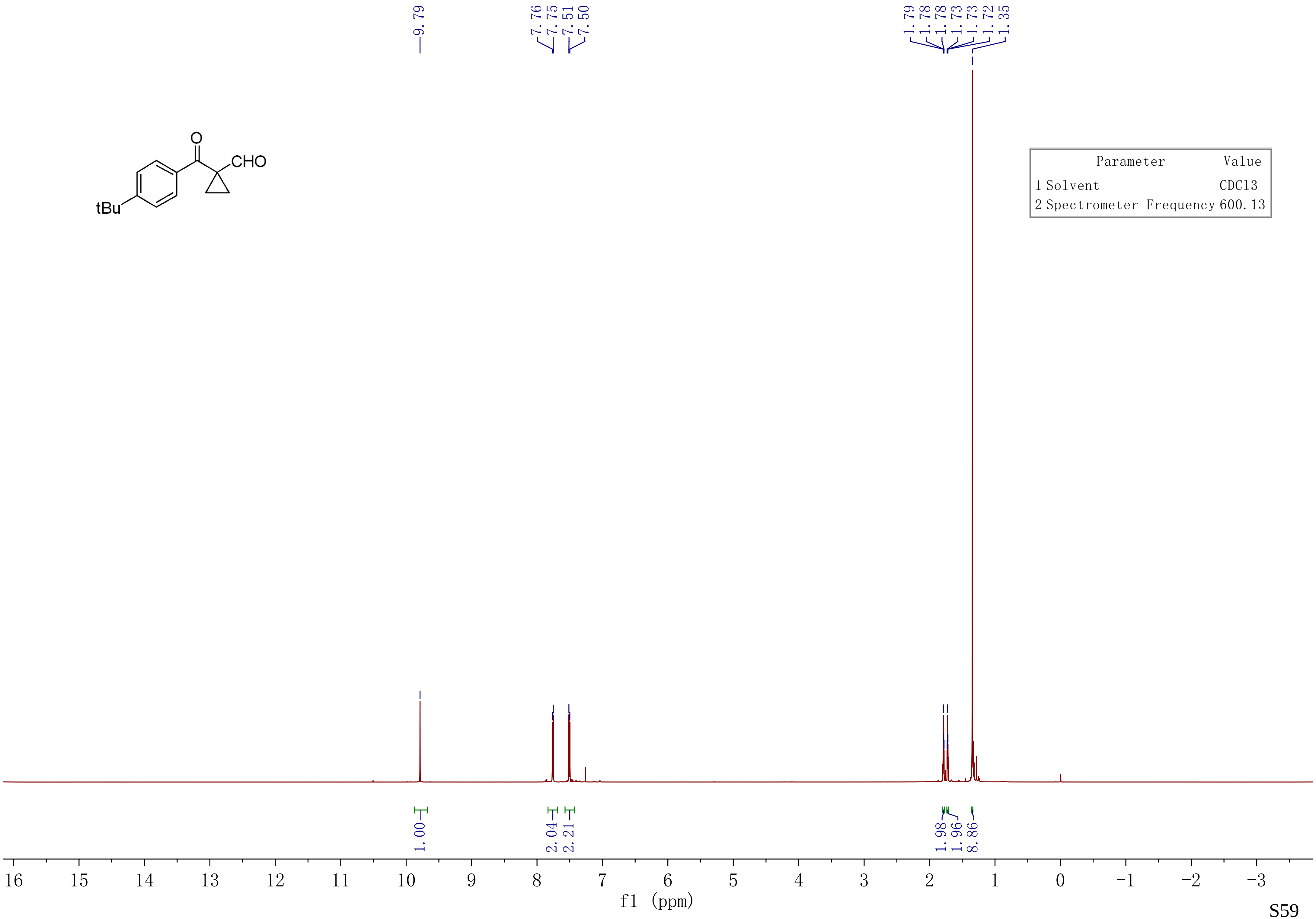
CHO
O
tBu
9.79
7.76
7.75
7.51
7.50
1.79
1.78
1.78
1.73
1.73
1.73
1.72
1.35
Parameter Value
1 Solvent CDCl3
2 Spectrometer Frequency 600.13
1.00
2.04
2.21
1.98
1.96
8.86
16 15 14 13 12 11 10 9 8 7 6 5 4 3 2 1 0 −1 −2 −3
f1 (ppm)

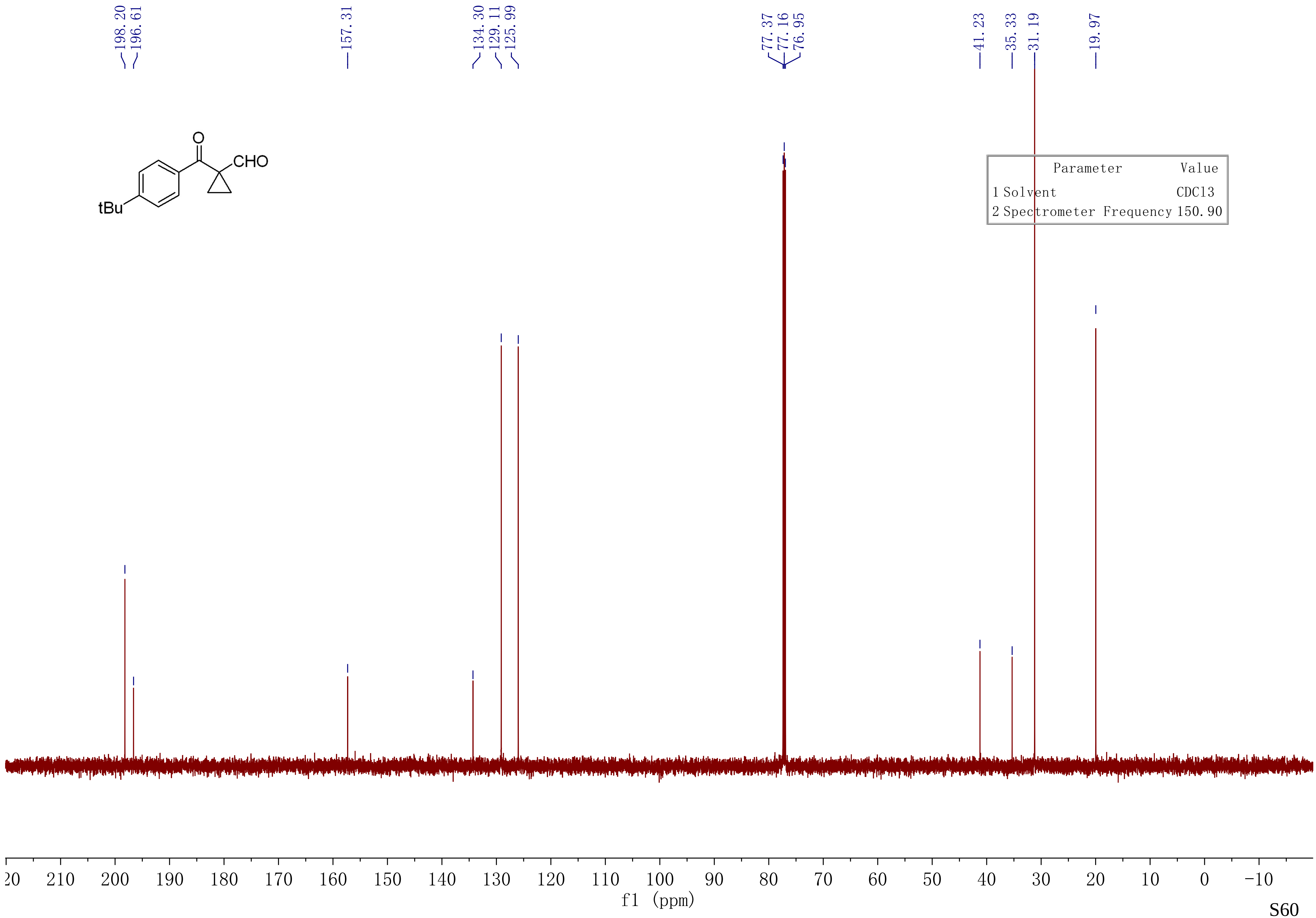
198.20
196.61
157.31
134.30
129.11
125.99
77.37
77.16
76.95
41.23
35.33
31.19
19.97
O
CHO
tBu
Parameter Value
1 Solvent CDCl3
2 Spectrometer Frequency 150.90
f1 (ppm)

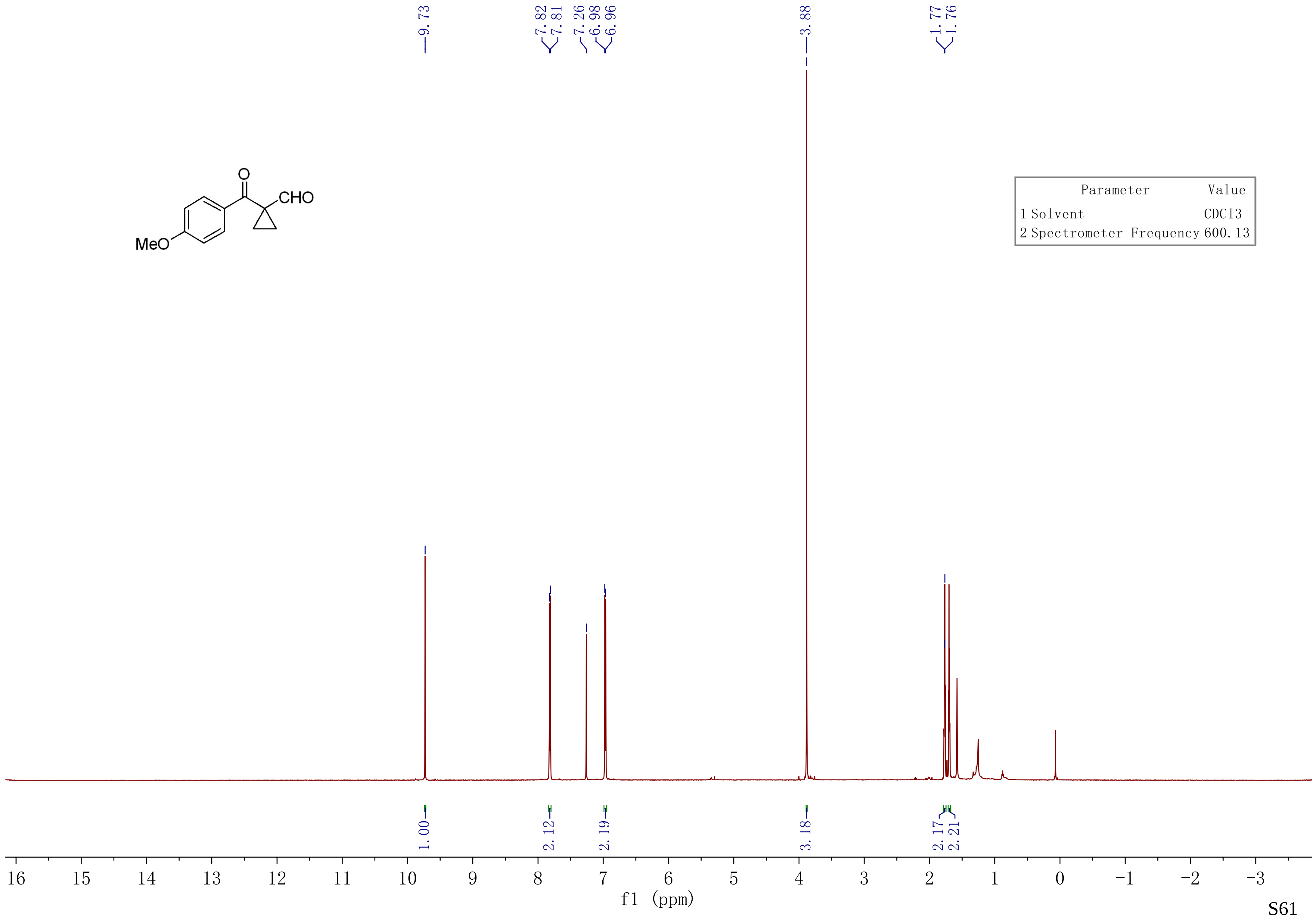

9.73
7.82
7.81
7.26
6.98
6.96
3.88
1.77
1.76
CHO
MeO
O
Parameter
Value
1 Solvent
CDCl3
2 Spectrometer Frequency
600.13
1.00
2.12
2.19
3.18
2.17
2.21
f1 (ppm)

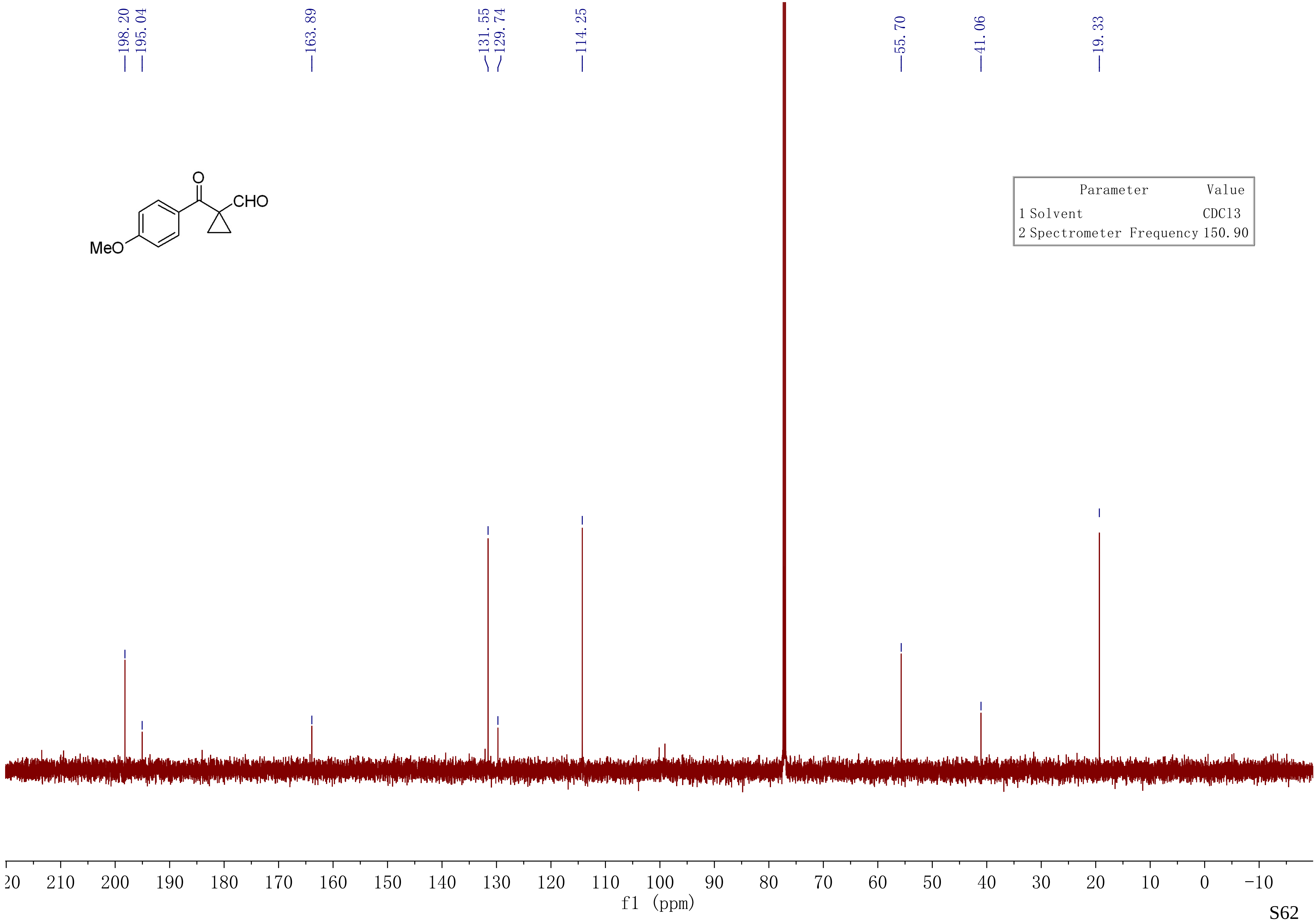
198.20
195.04
163.89
131.55
129.74
114.25
55.70
41.06
19.33
O
CHO
MeO
Parameter Value
1 Solvent CDCl3
2 Spectrometer Frequency 150.90
f1 (ppm)

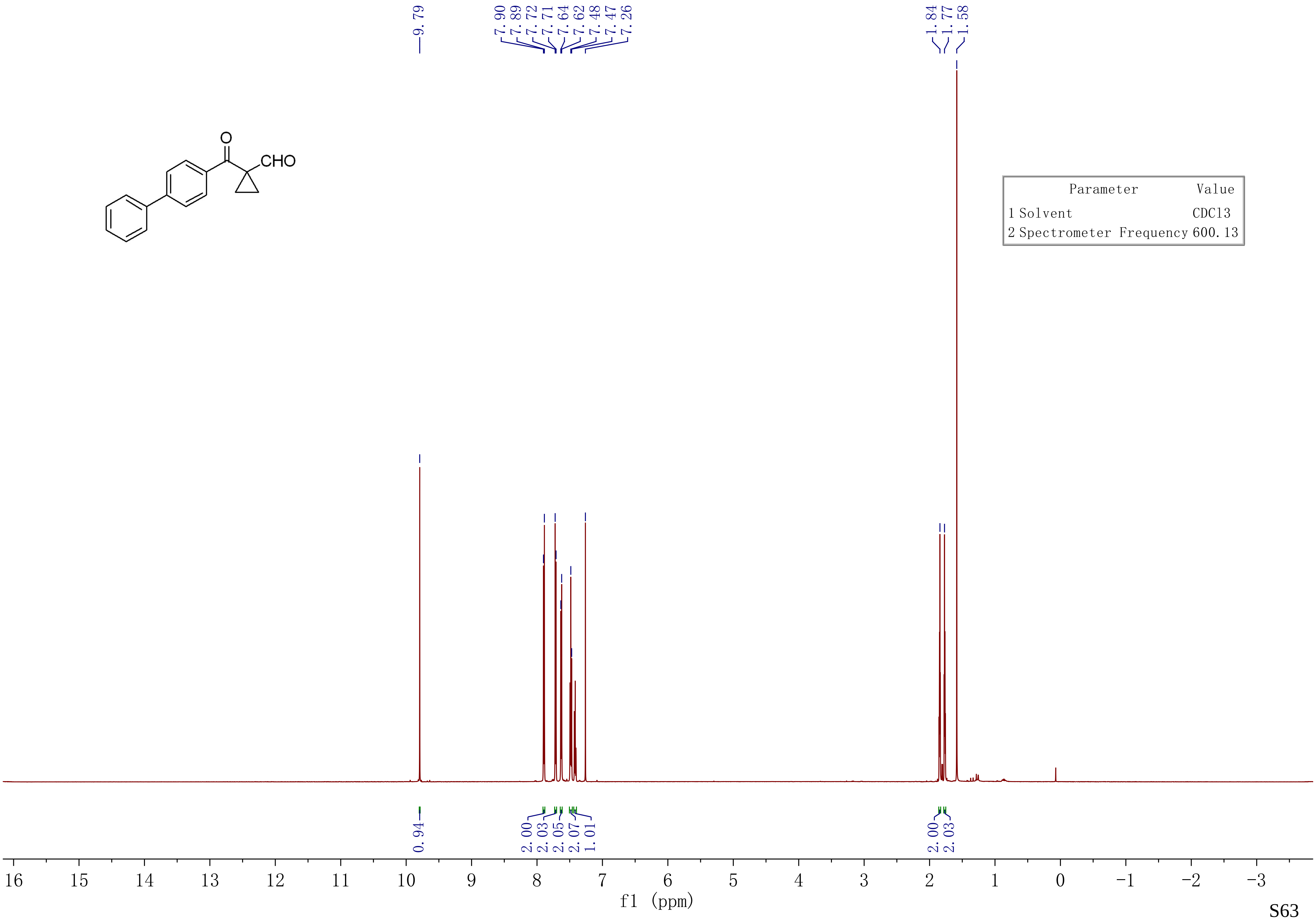
9.79
7.90 7.89 7.72 7.71 7.64 7.62 7.48 7.47 7.26
1.84 1.77 1.58
O
CHO
Parameter Value
1 Solvent CDCl3
2 Spectrometer Frequency 600.13
0.94
2.00 2.03 2.05 2.07 1.01
2.00 2.03
16 15 14 13 12 11 10 9 8 7 6 5 4 3 2 1 0 −1 −2 −3
f1 (ppm)

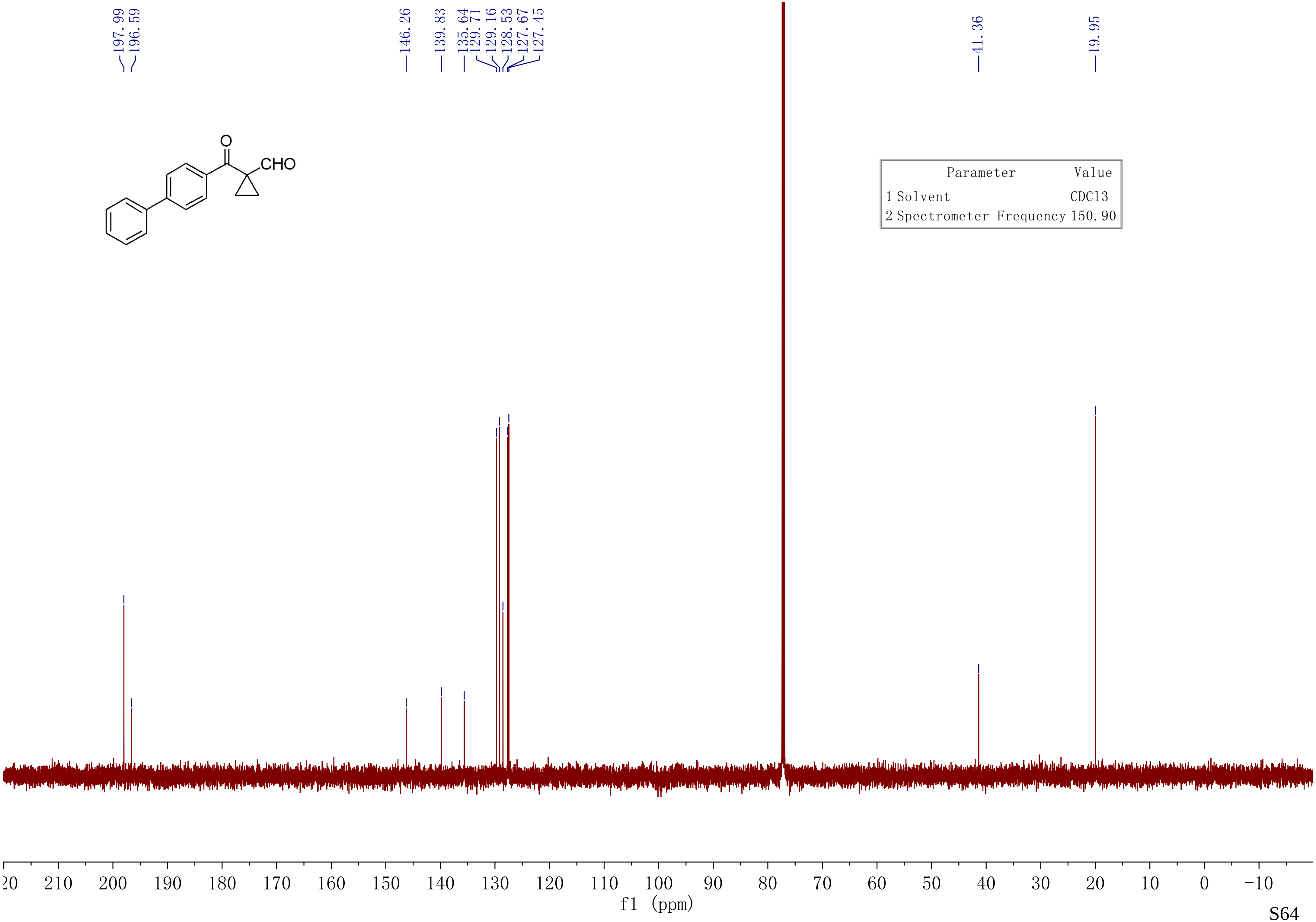
197.99
196.59
146.26
139.83
135.64
129.71
129.16
128.53
127.67
127.45
41.36
19.95
O
CHO
Parameter
Value
1 Solvent
CDCl3
2 Spectrometer Frequency
150.90
20
210
200
190
180
170
160
150
140
130
120
110
100
90
80
70
60
50
40
30
20
10
0
−10
f1 (ppm)

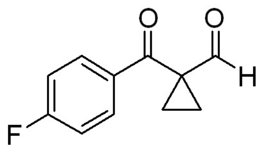

Parameter	Value
1 Solvent	CDCl3
2 Spectrometer Frequency	400.20

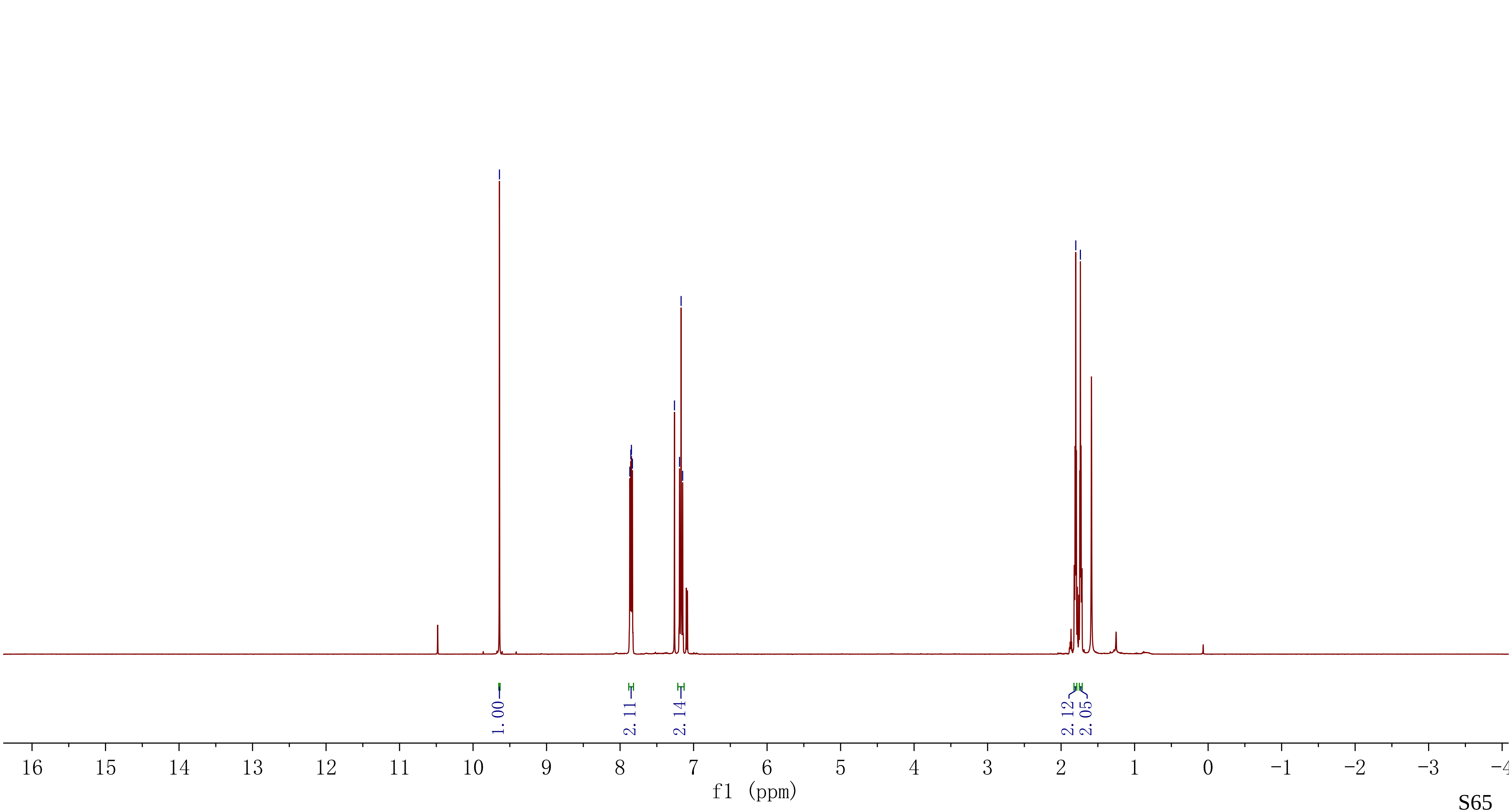

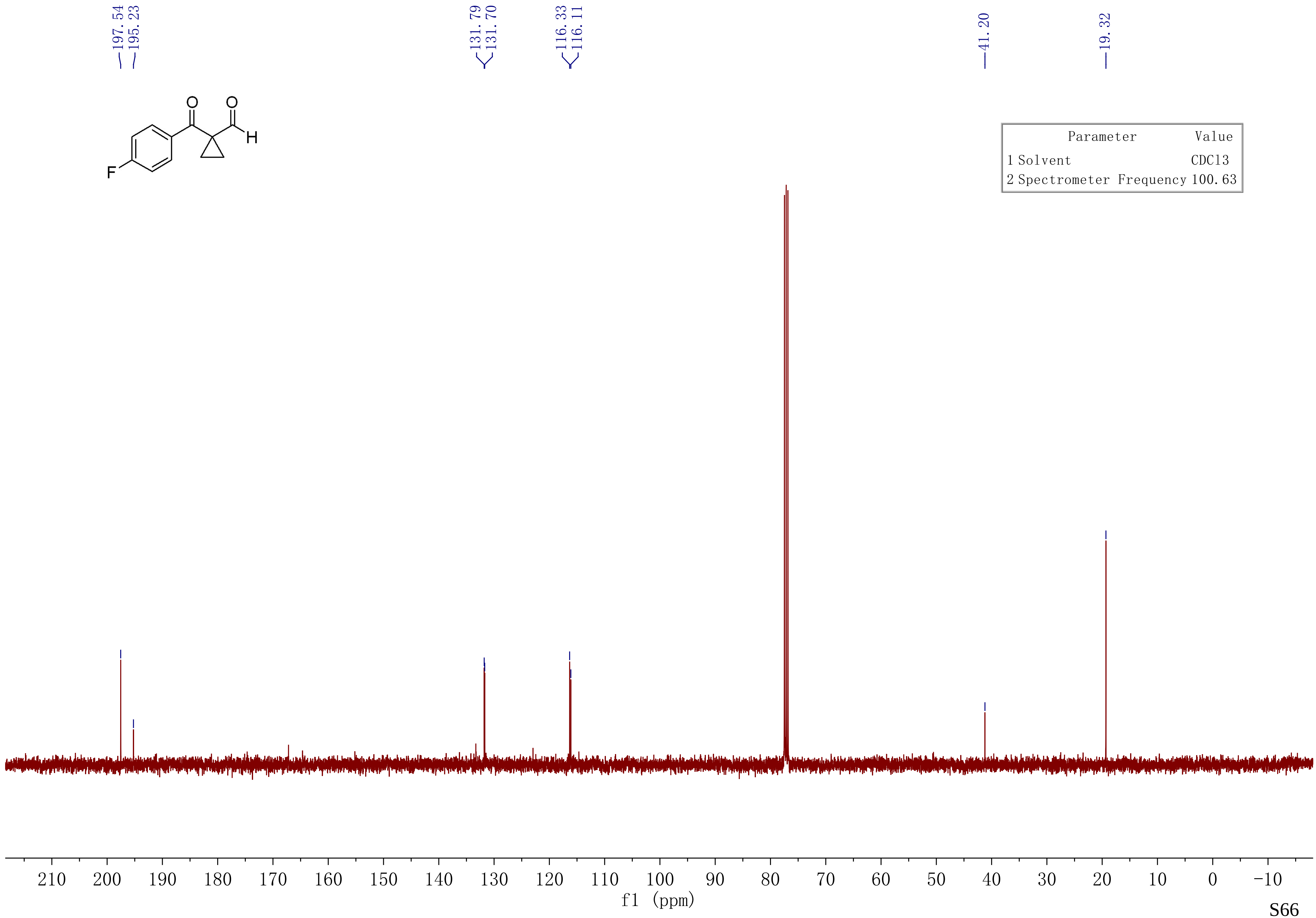
197.54
195.23
131.79
131.70
116.33
116.11
41.20
19.32
Parameter Value
1 Solvent CDCl3
2 Spectrometer Frequency 100.63
210 200 190 180 170 160 150 140 130 120 110 100 90 80 70 60 50 40 30 20 10 0 -10
f1 (ppm)

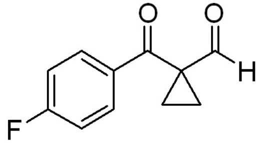

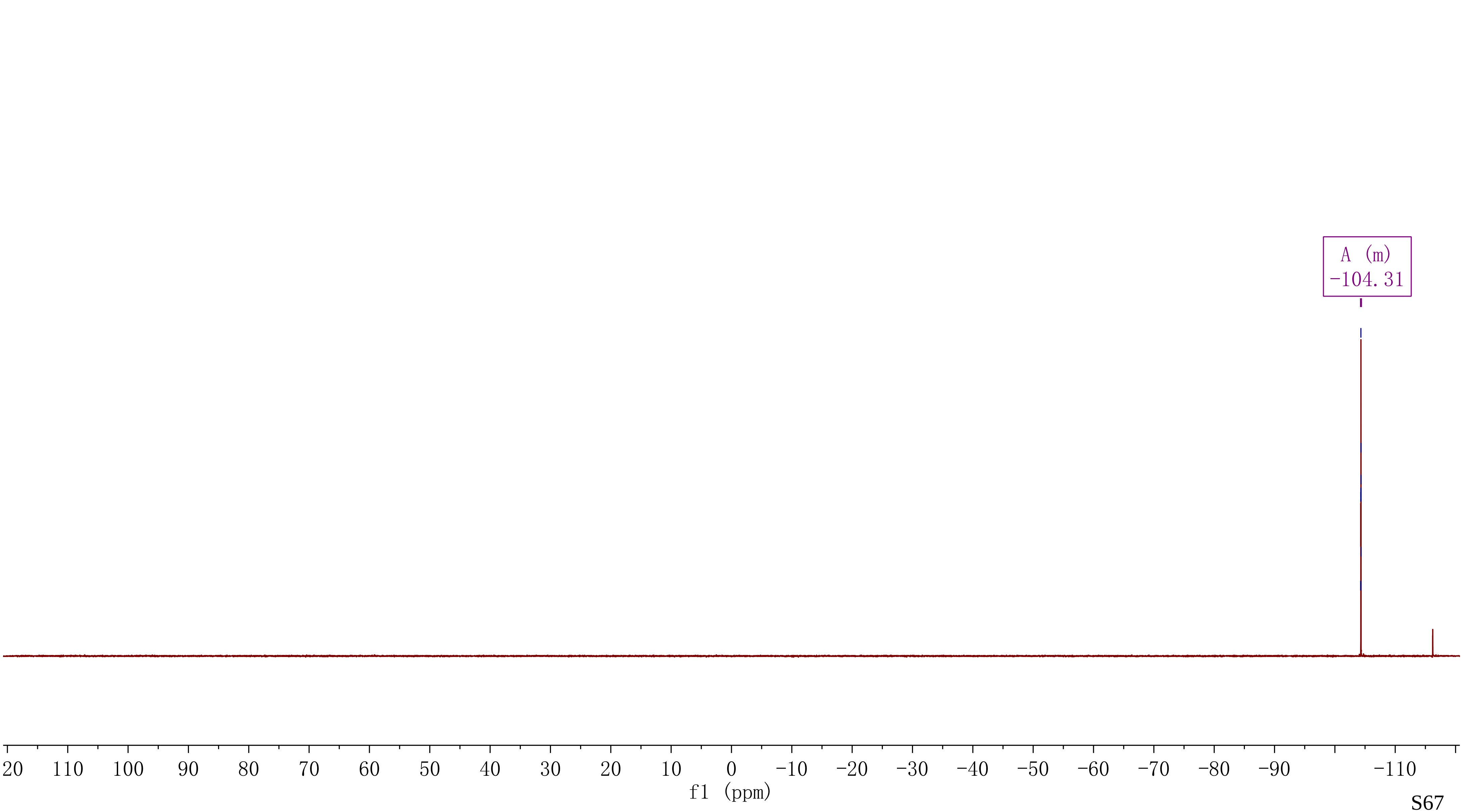

	Parameter	Value
1	Solvent	CDCl3
2	Spectrometer Frequency	376.56

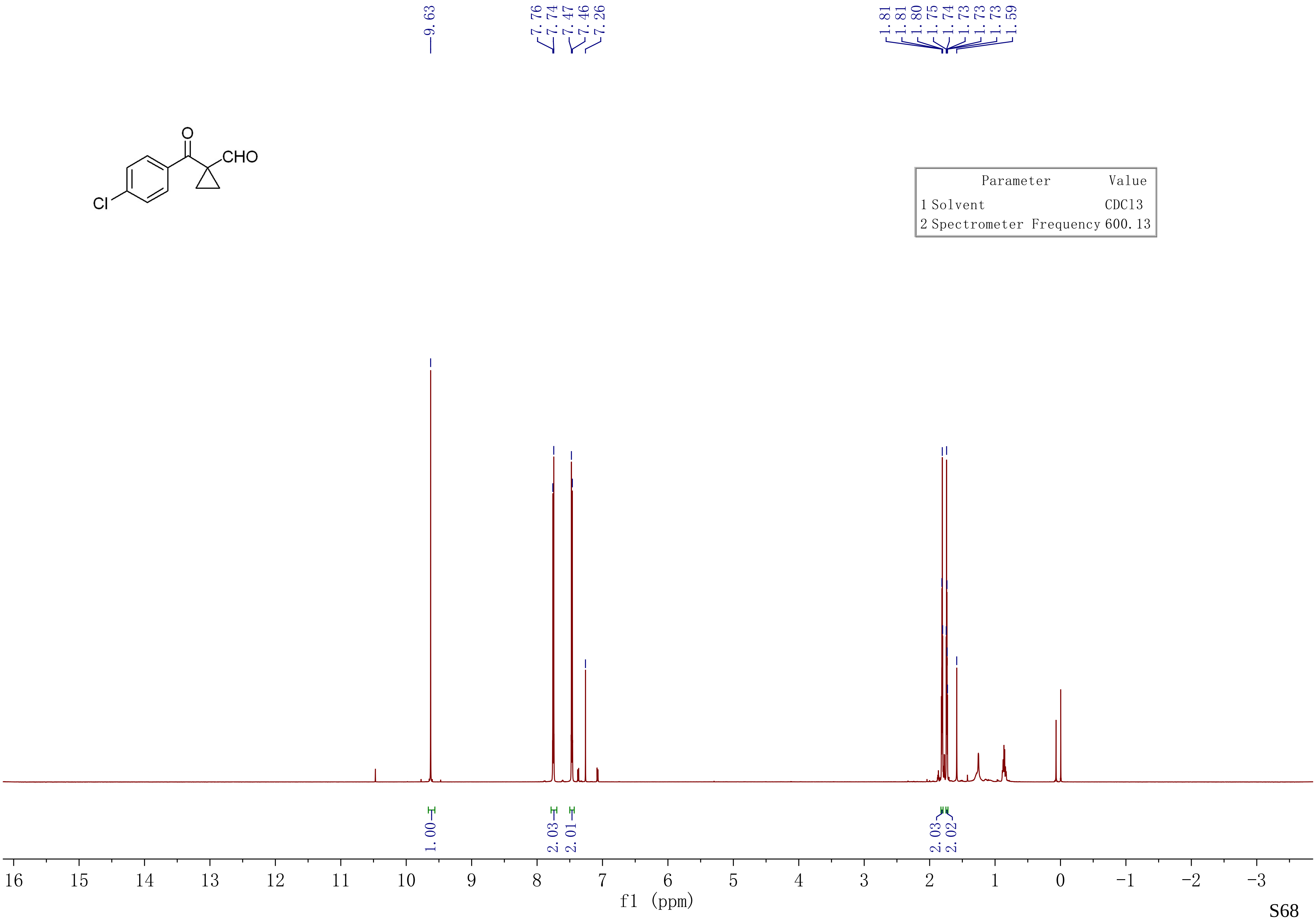

9.63
7.76
7.74
7.47
7.46
7.26
1.81
1.81
1.80
1.75
1.74
1.73
1.73
1.73
1.59
O
CHO
Cl
Parameter Value
1 Solvent CDCl3
2 Spectrometer Frequency 600.13
1.00
2.03
2.01
2.03
2.02
16 15 14 13 12 11 10 9 8 7 6 5 4 3 2 1 0 −1 −2 −3
f1 (ppm)

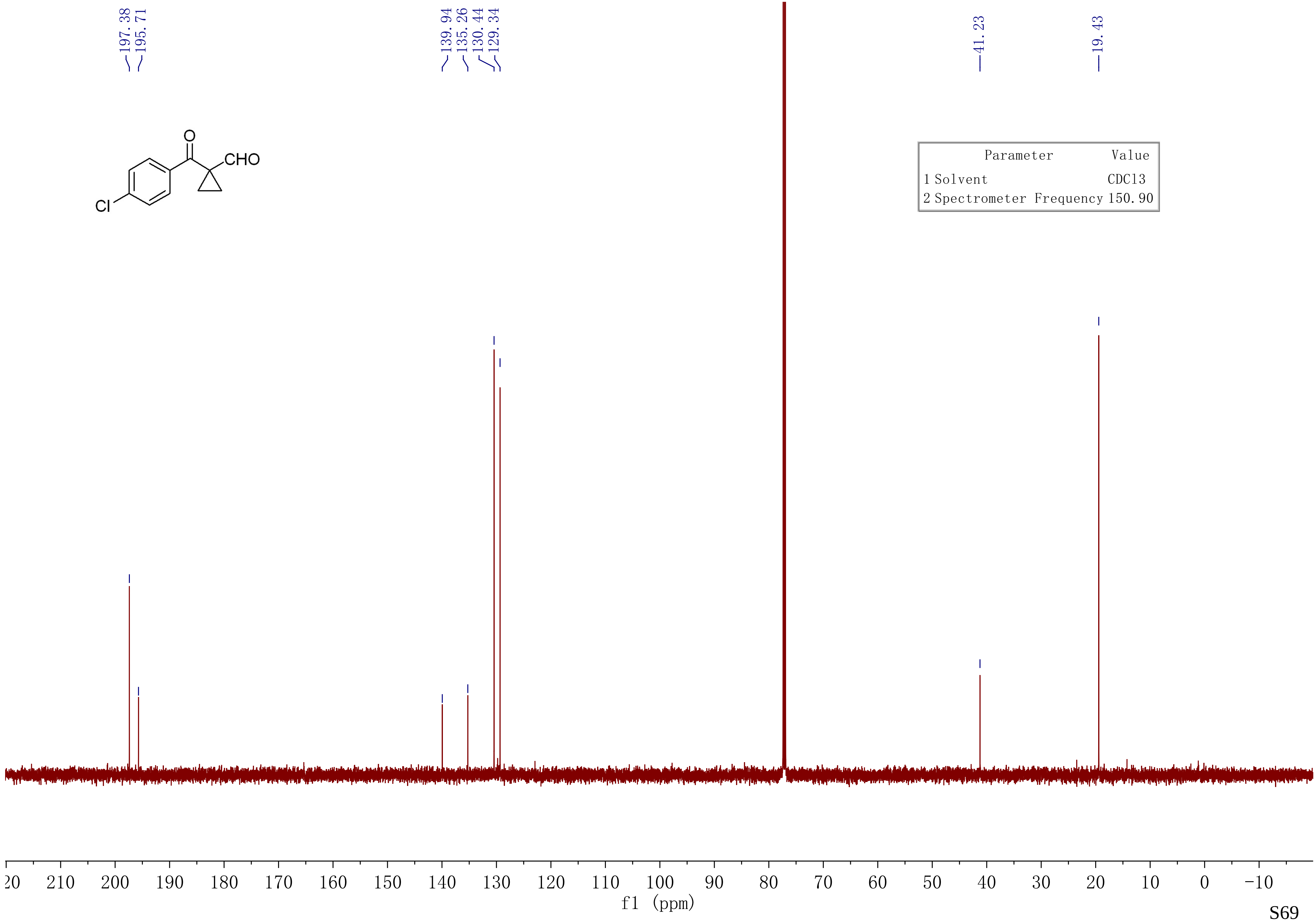
197.38
195.71
139.94
135.26
130.44
129.34
41.23
19.43
O
CHO
Cl
Parameter Value
1 Solvent CDCl3
2 Spectrometer Frequency 150.90
220 210 200 190 180 170 160 150 140 130 120 110 100 90 80 70 60 50 40 30 20 10 0 −10
f1 (ppm)

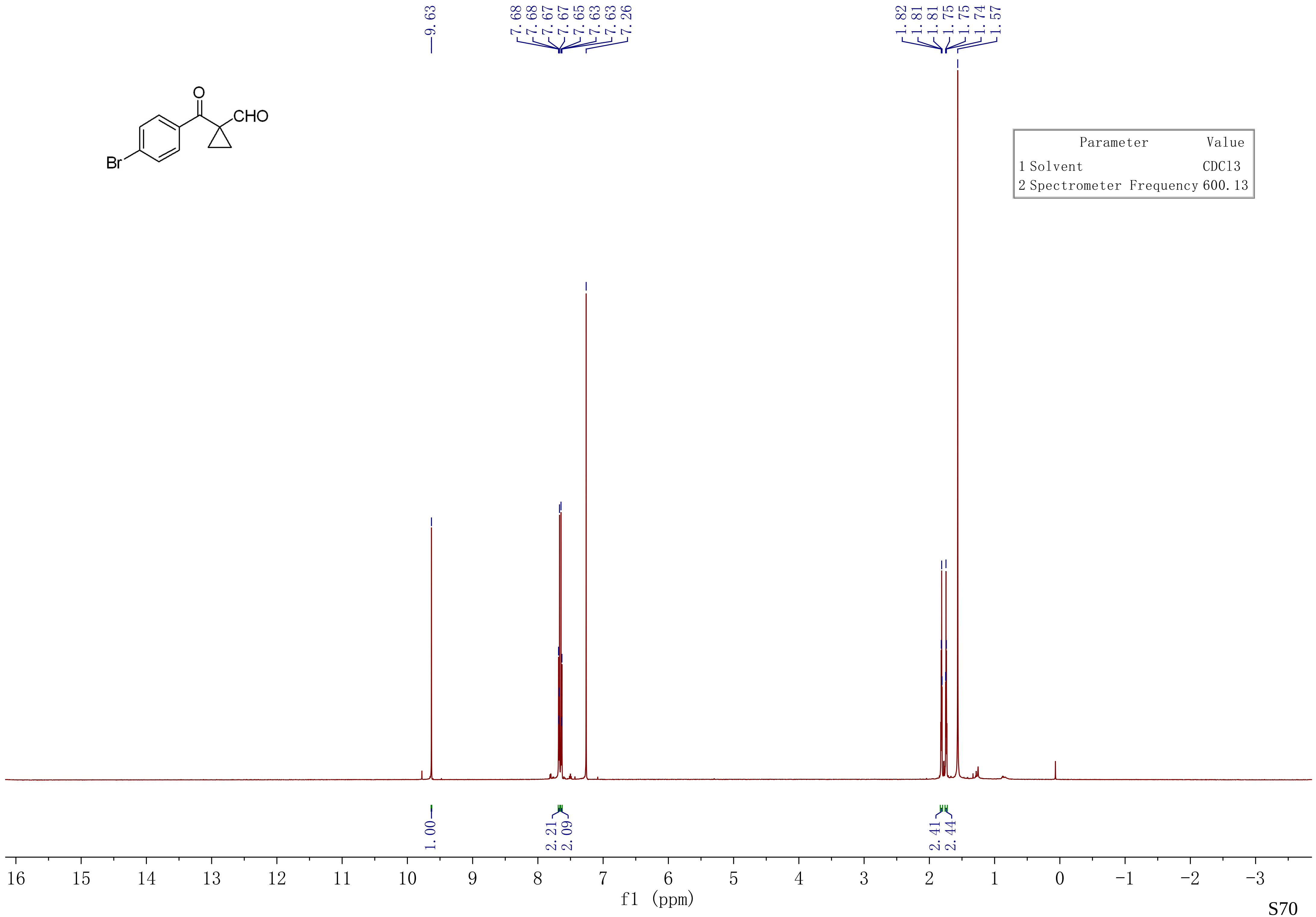
9.63
7.68 7.68 7.67 7.67 7.65 7.63 7.63 7.26
1.82 1.81 1.81 1.75 1.75 1.74 1.57
CHO
Br
O
Parameter Value
1 Solvent CDCl3
2 Spectrometer Frequency 600.13
1.00
2.21 2.09
2.41 2.44
16 15 14 13 12 11 10 9 8 7 6 5 4 3 2 1 0 −1 −2 −3
f1 (ppm)

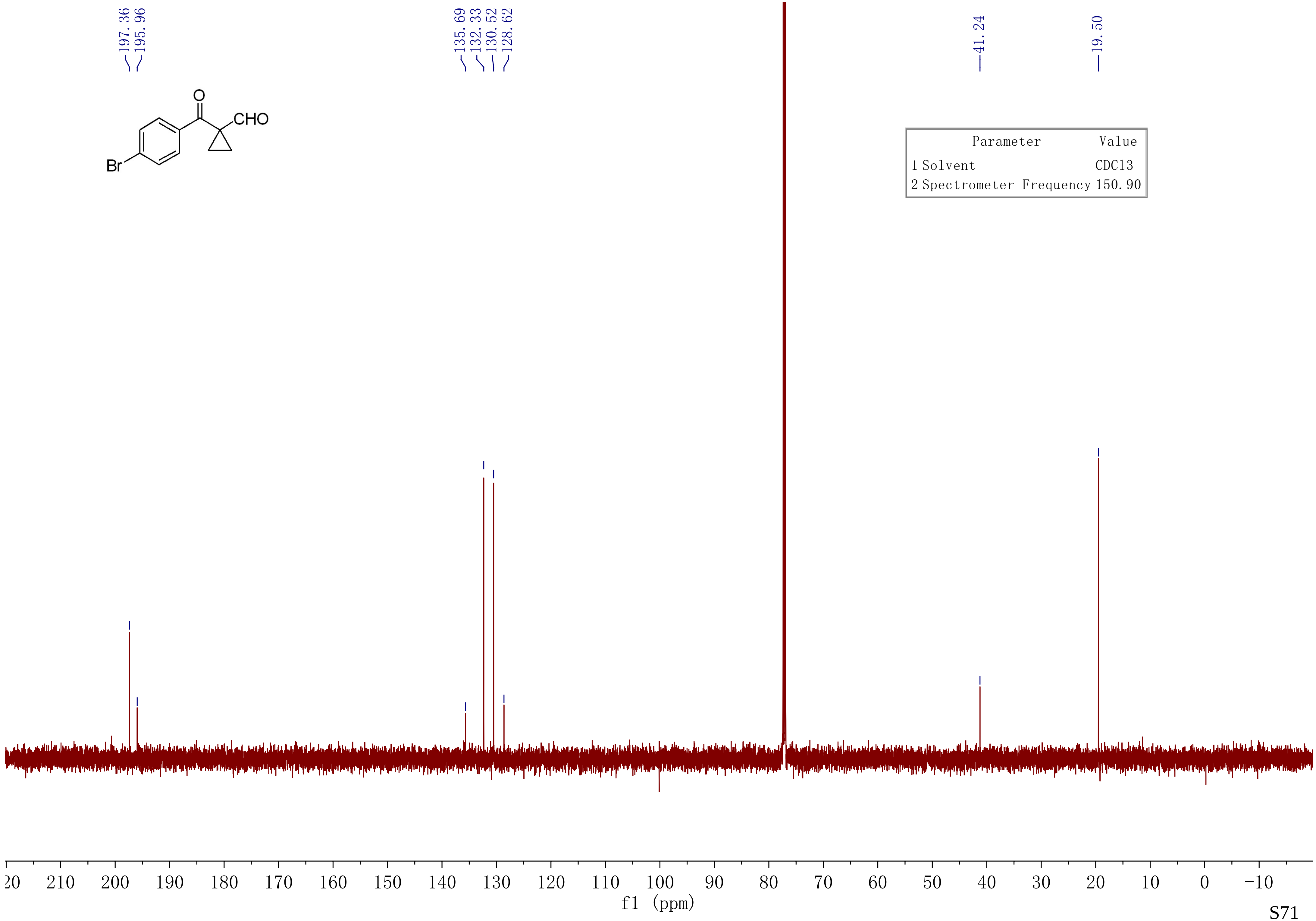

Parameter	Value
1 Solvent	CDCl3
2 Spectrometer Frequency	150.90

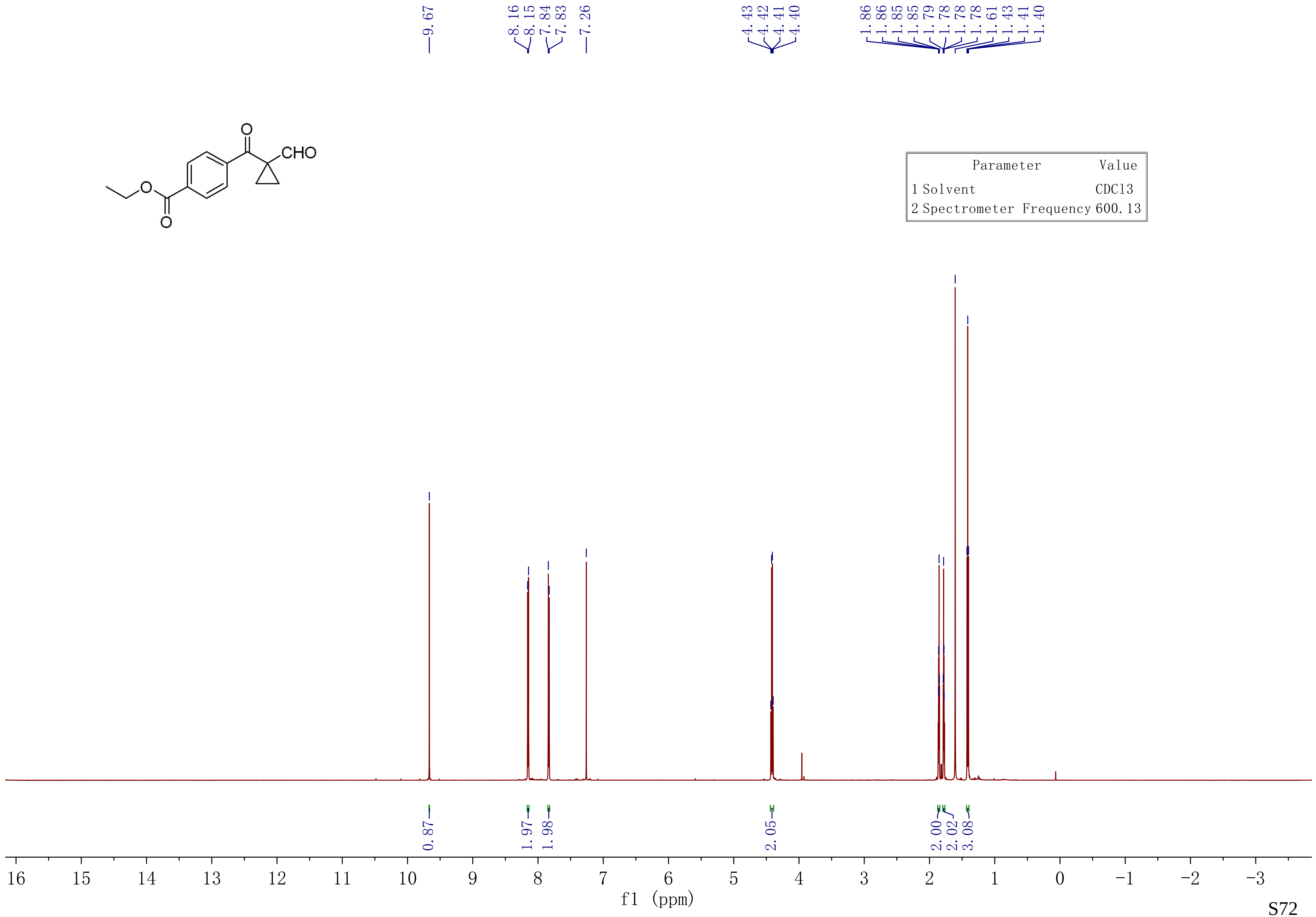
9.67
8.16
8.15
7.84
7.83
7.26
4.43
4.42
4.41
4.40
1.86
1.86
1.85
1.85
1.79
1.78
1.78
1.78
1.61
1.43
1.41
1.40
CHO
O
O
O
Parameter Value
1 Solvent CDCl3
2 Spectrometer Frequency 600.13
0.87
1.97
1.98
2.05
2.00
2.02
3.08
16 15 14 13 12 11 10 9 8 7 6 5 4 3 2 1 0 −1 −2 −3
f1 (ppm)

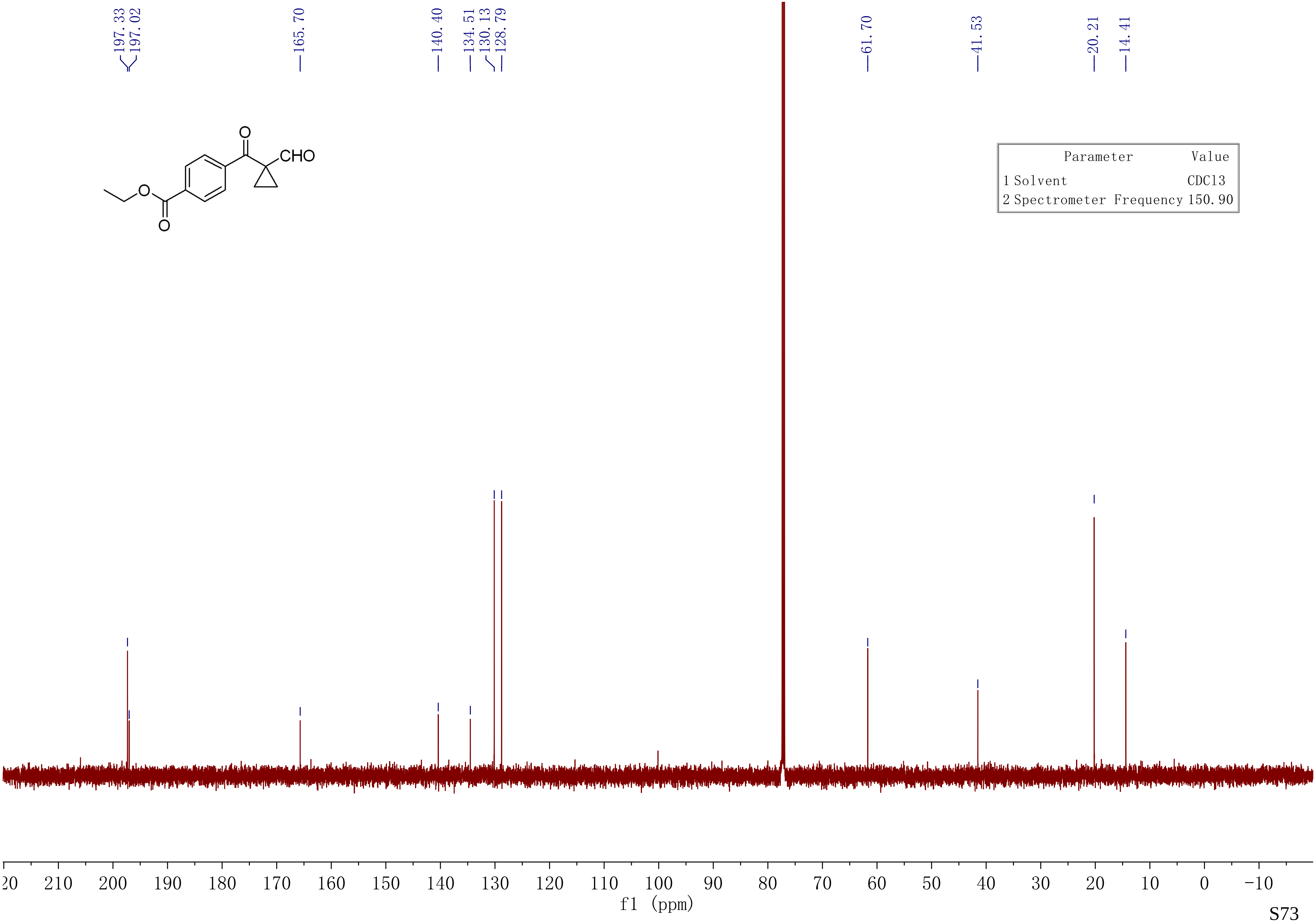
197.33
197.02
165.70
140.40
134.51
130.13
128.79
61.70
41.53
20.21
14.41
CHO
O
O
O
Parameter Value
1 Solvent CDCl3
2 Spectrometer Frequency 150.90
f1 (ppm)

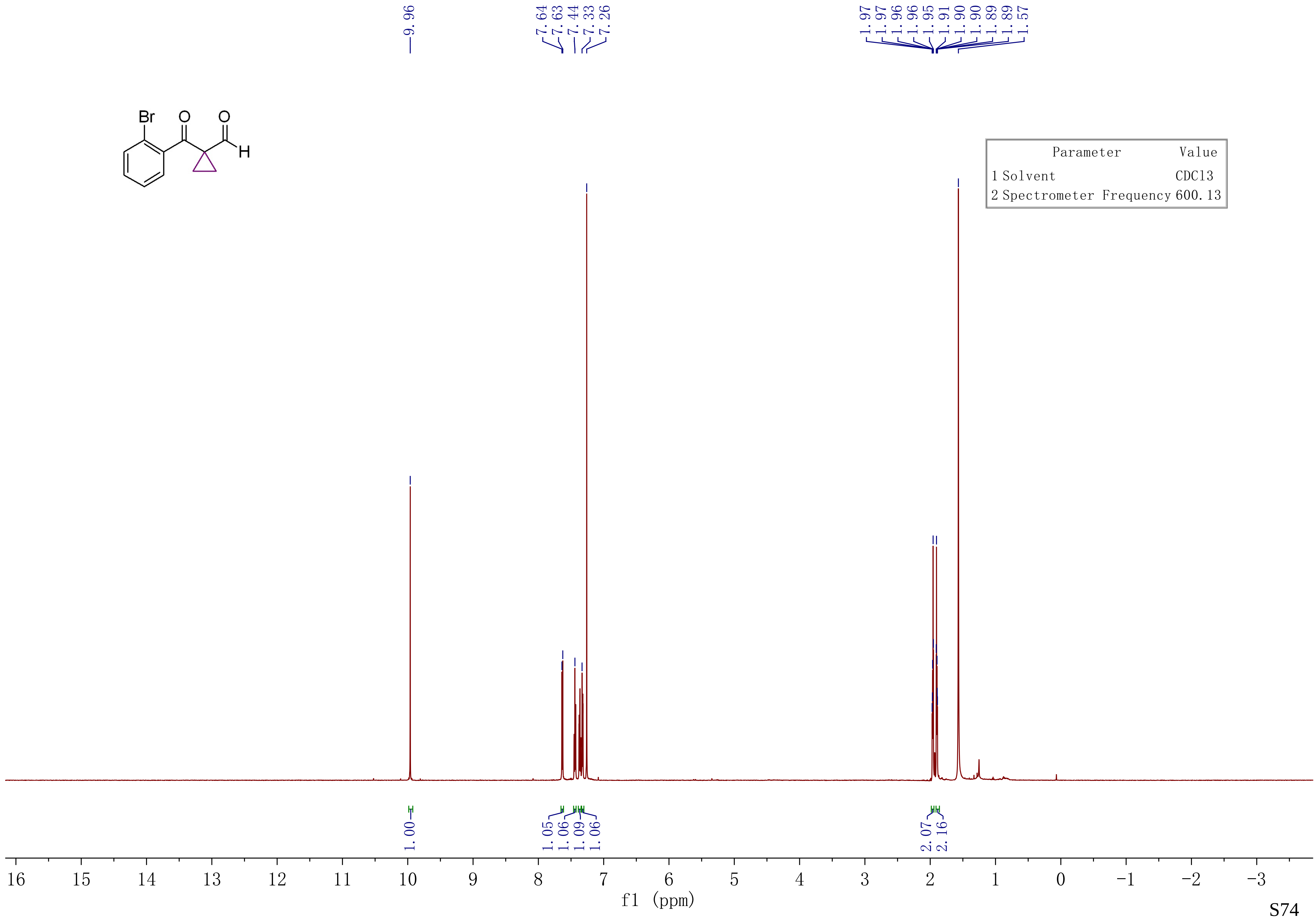
9.96
7.64
7.63
7.44
7.33
7.26
1.97
1.97
1.96
1.96
1.95
1.91
1.90
1.90
1.89
1.89
1.57
Br
O
O
H
Parameter Value
1 Solvent CDCl3
2 Spectrometer Frequency 600.13
1.00
1.05
1.06
1.09
1.06
2.07
2.16
16 15 14 13 12 11 10 9 8 7 6 5 4 3 2 1 0 −1 −2 −3
f1 (ppm)

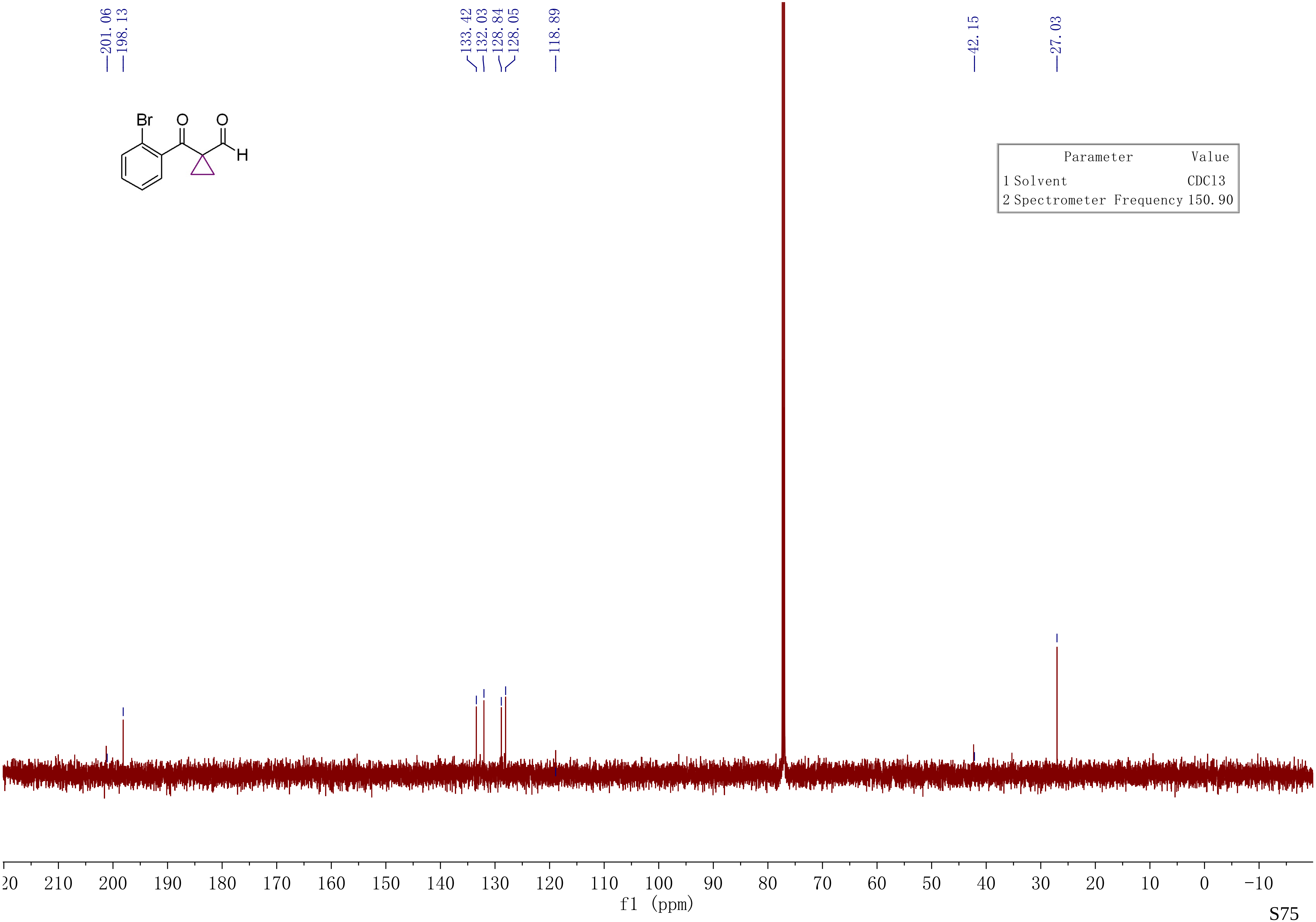

Parameter	Value
1 Solvent	CDCl3
2 Spectrometer Frequency	150.90

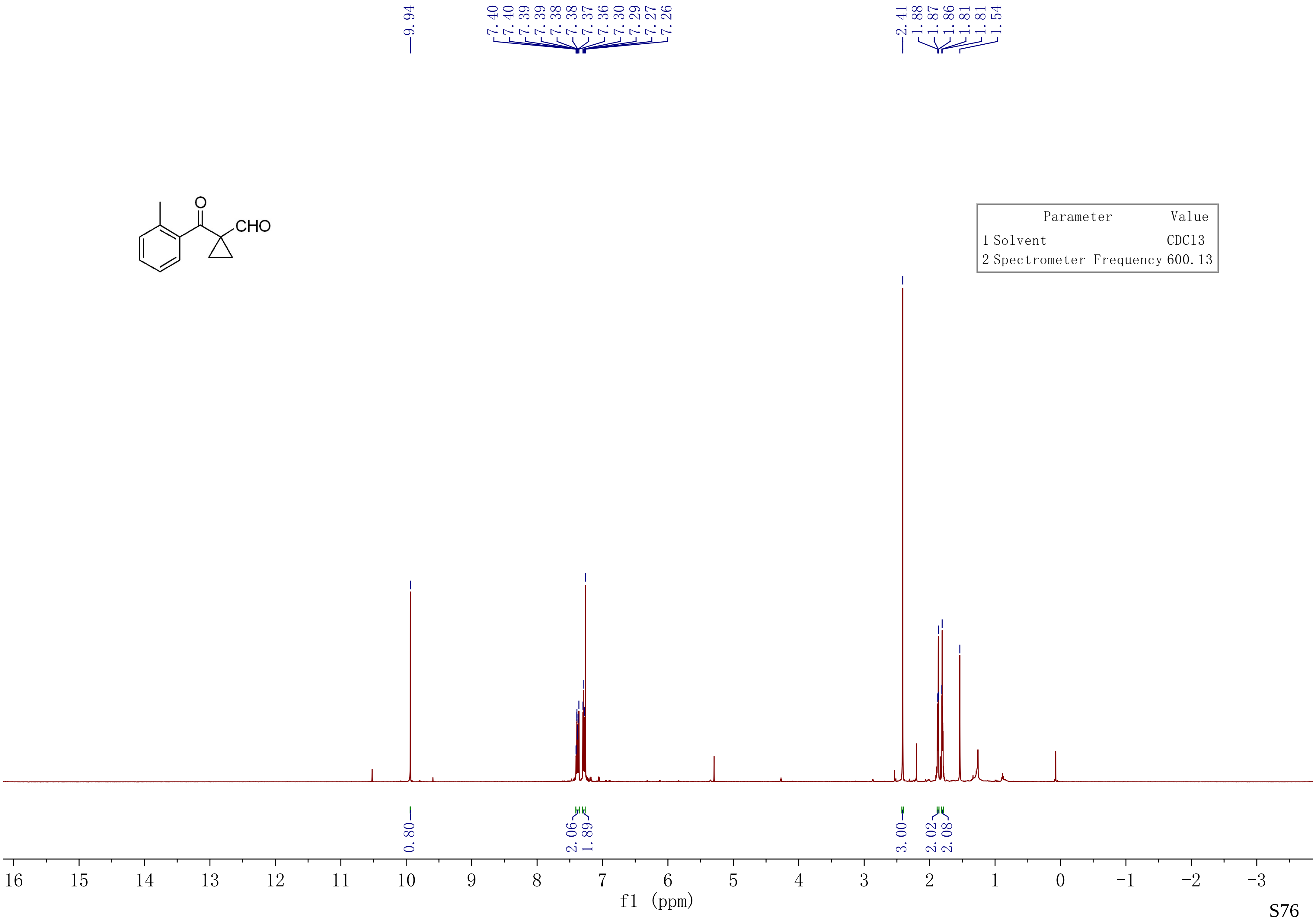
9.94
7.40
7.40
7.39
7.39
7.38
7.38
7.37
7.36
7.30
7.29
7.27
7.26
2.41
1.88
1.87
1.86
1.81
1.81
1.54
O
CHO
Parameter Value
1 Solvent CDCl3
2 Spectrometer Frequency 600.13
0.80
2.06
1.89
3.00
2.02
2.08
16 15 14 13 12 11 10 9 8 7 6 5 4 3 2 1 0 −1 −2 −3
f1 (ppm)

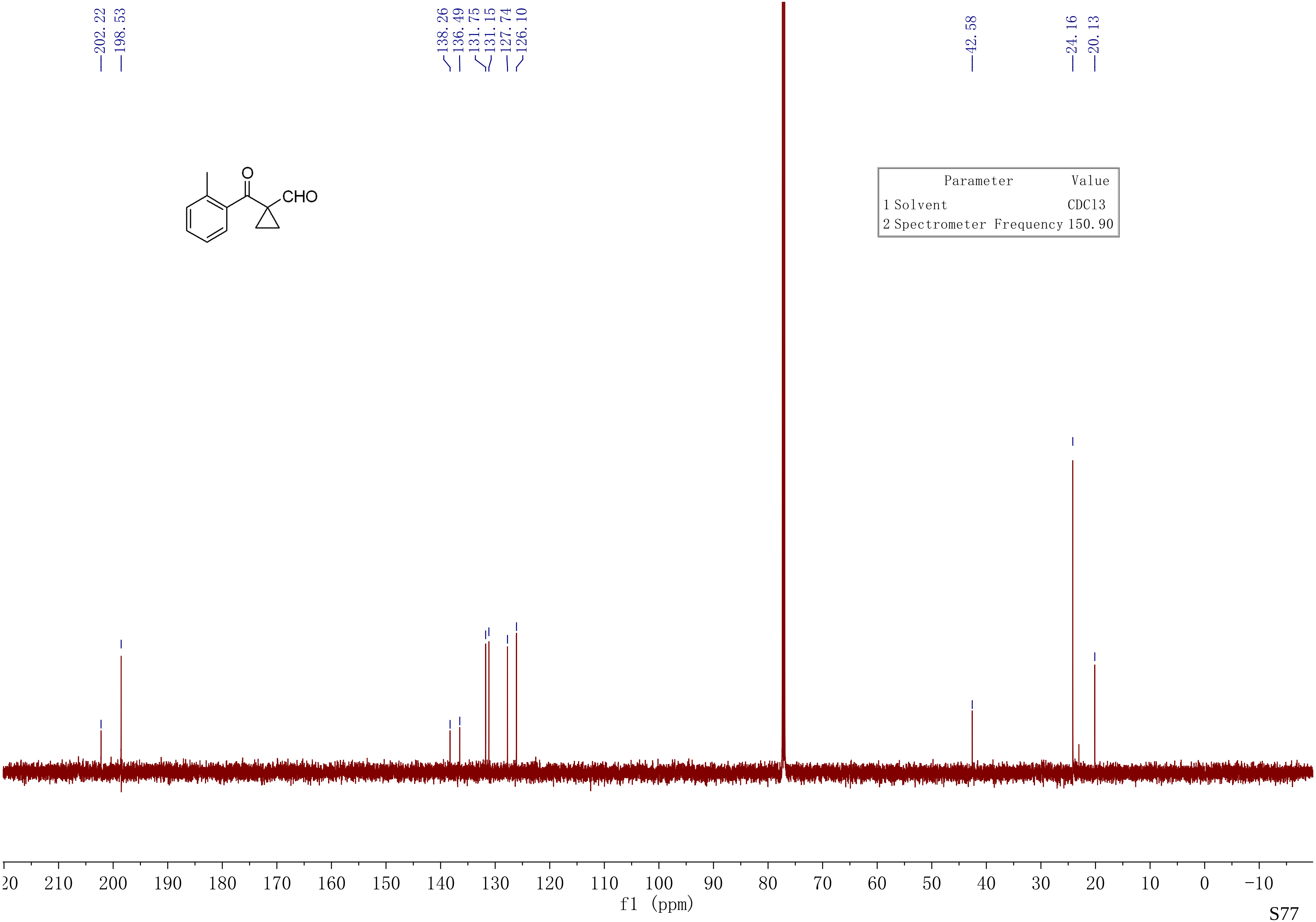
202.22
198.53
138.26
136.49
131.75
131.15
127.74
126.10
42.58
24.16
20.13
O
CHO
Parameter Value
1 Solvent CDCl3
2 Spectrometer Frequency 150.90
f1 (ppm)

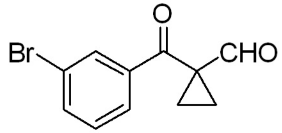

Parameter	Value
1 Solvent	CDCl3
2 Spectrometer Frequency	600.13

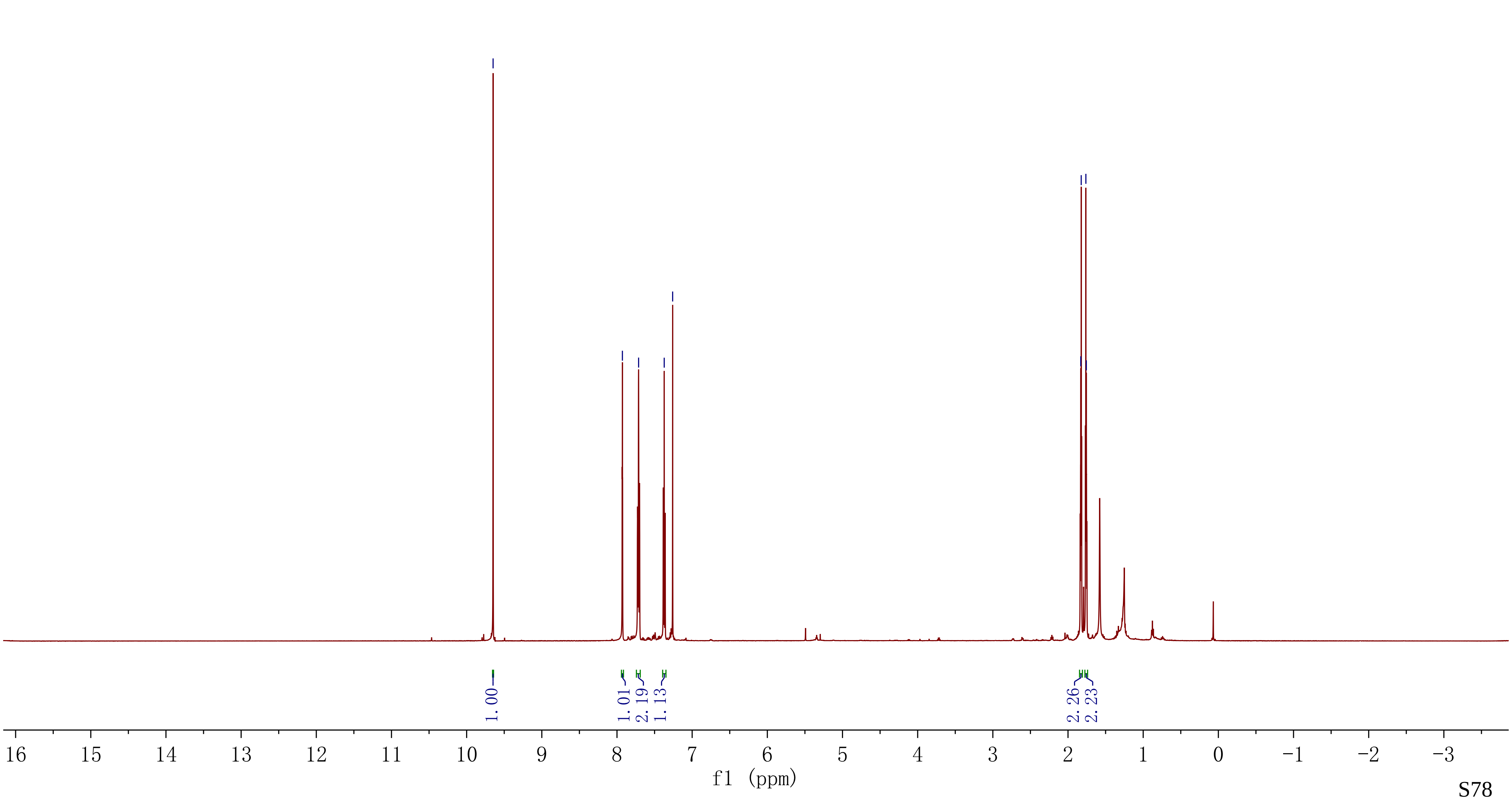

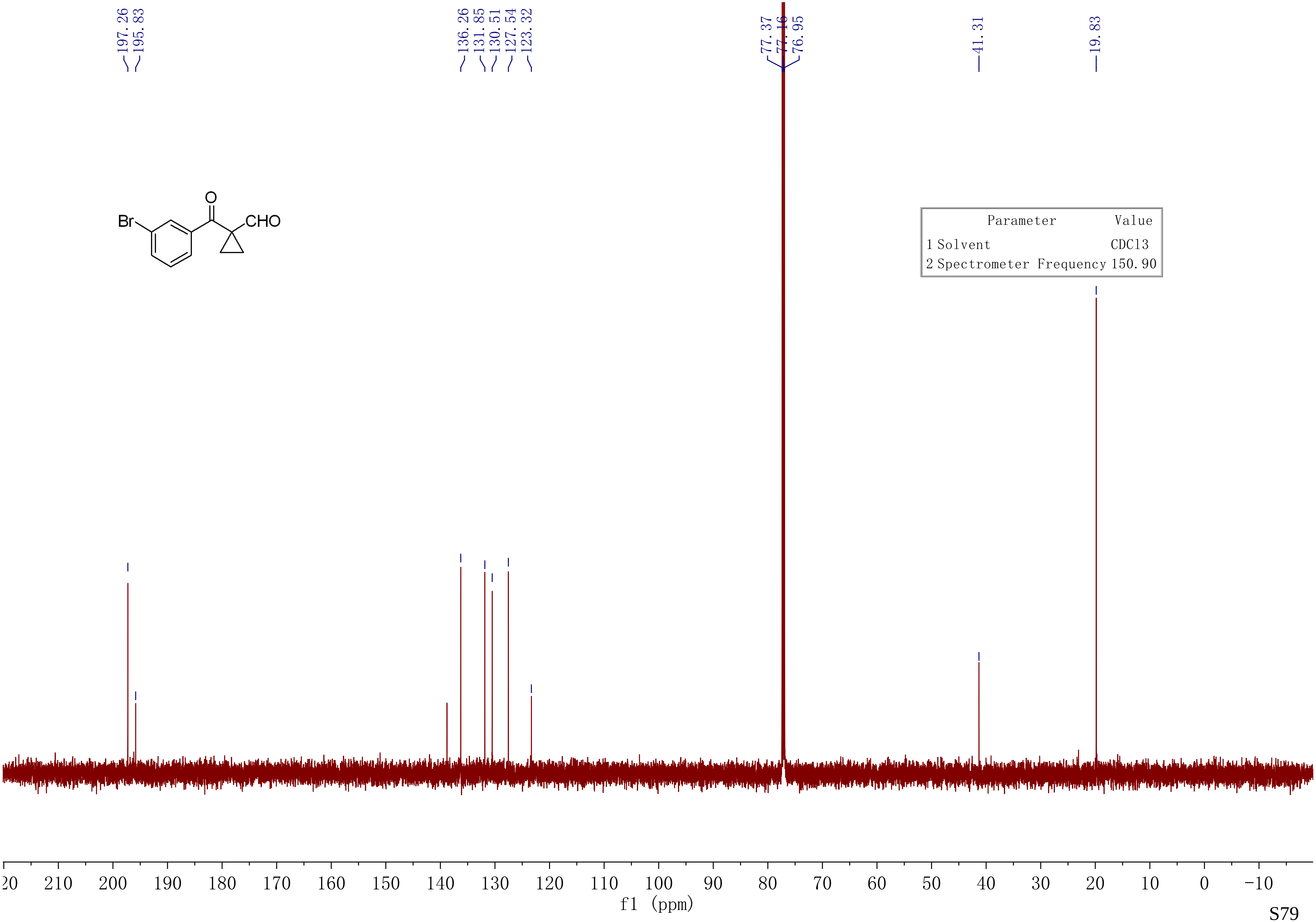

Parameter	Value
1 Solvent	CDCl3
2 Spectrometer Frequency	150.90

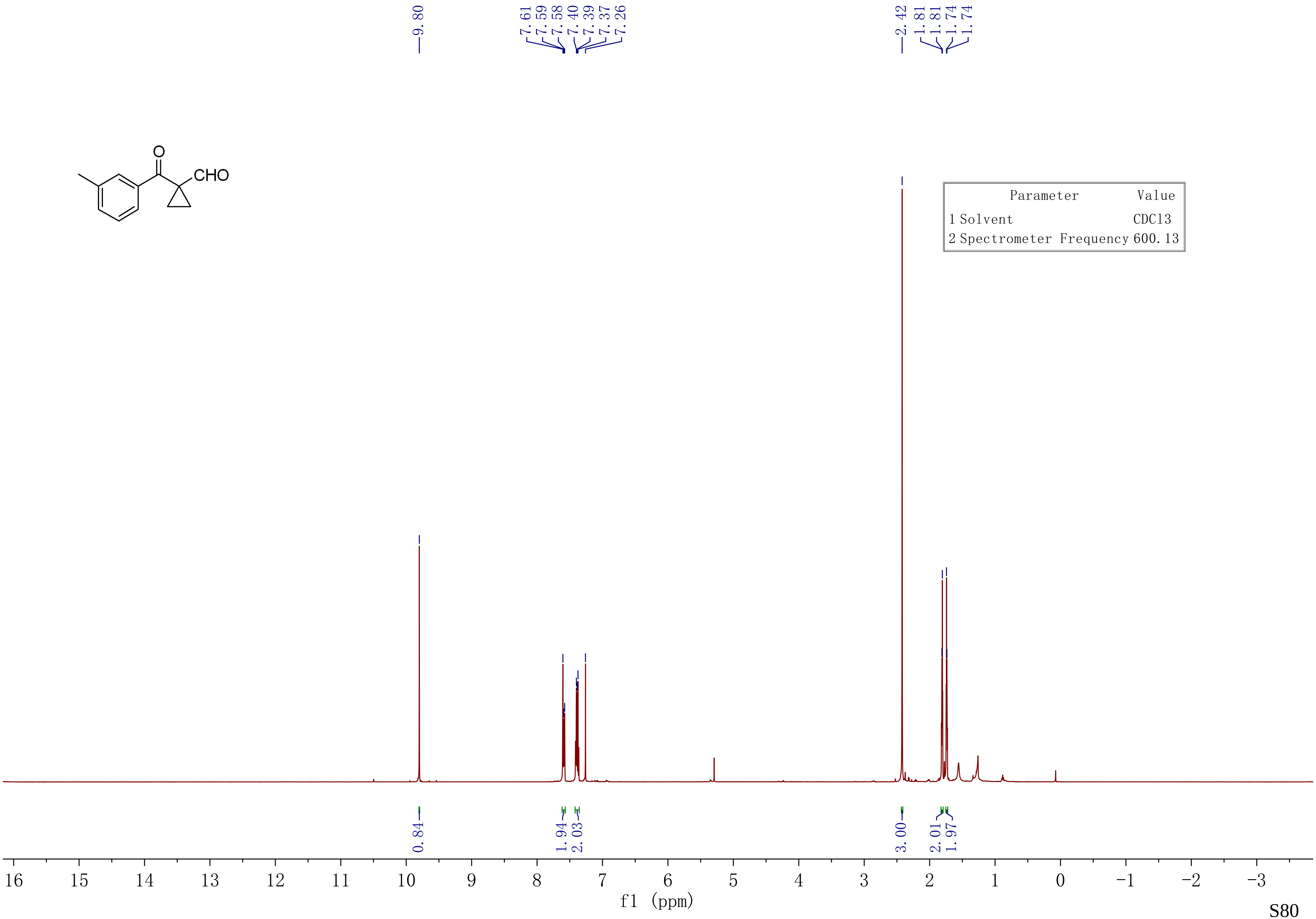
9.80
7.61
7.59
7.58
7.40
7.39
7.37
7.26
2.42
1.81
1.81
1.74
1.74
O
CHO
Parameter Value
1 Solvent CDCl3
2 Spectrometer Frequency 600.13
0.84
1.94
2.03
3.00
2.01
1.97
16 15 14 13 12 11 10 9 8 7 6 5 4 3 2 1 0 −1 −2 −3
f1 (ppm)

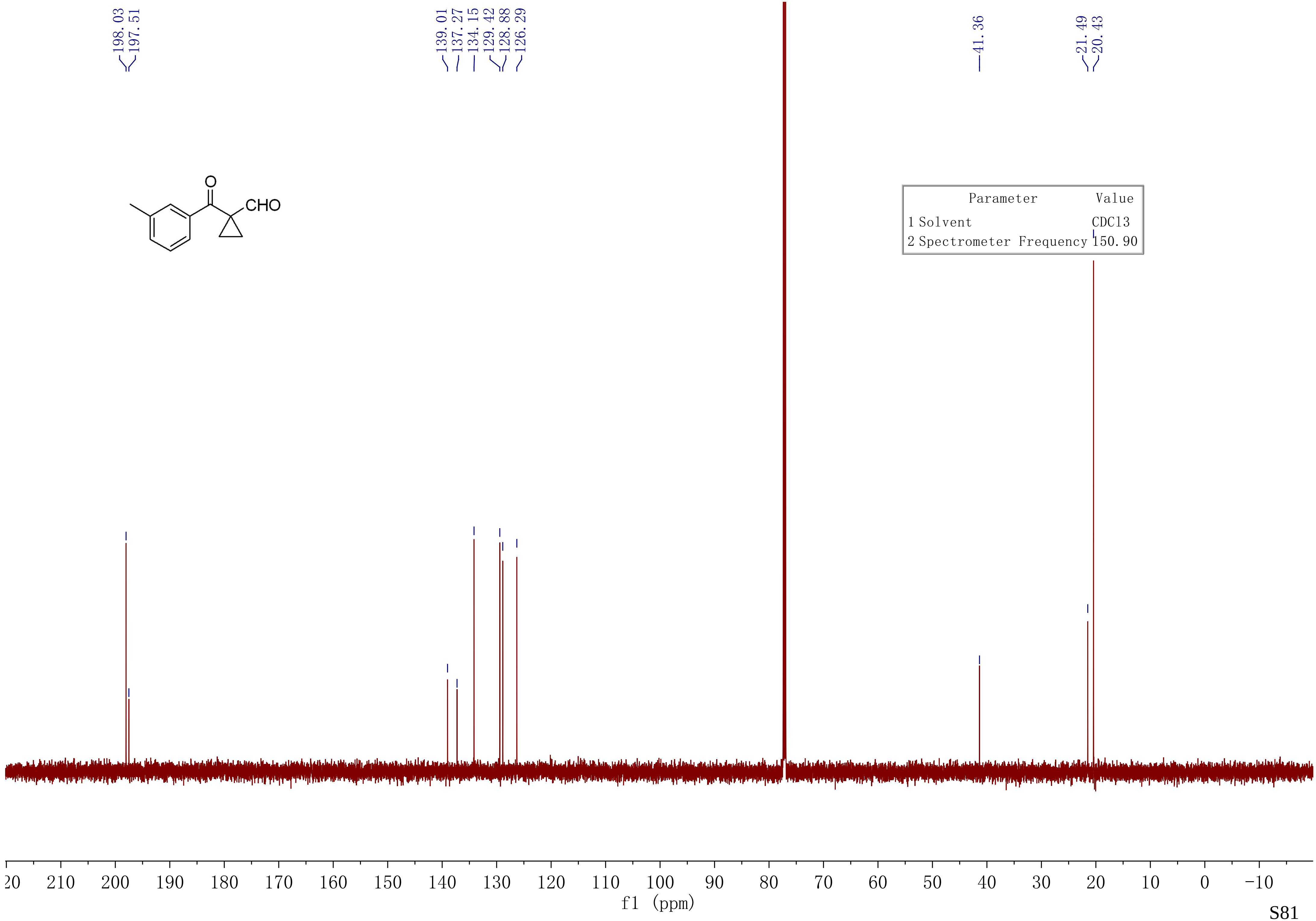
198.03
197.51
139.01
137.27
134.15
129.42
128.88
126.29
41.36
21.49
20.43
CHO
O
Parameter Value
1 Solvent CDCl3
2 Spectrometer Frequency 150.90
20 210 200 190 180 170 160 150 140 130 120 110 100 90 80 70 60 50 40 30 20 10 0 −10
f1 (ppm)

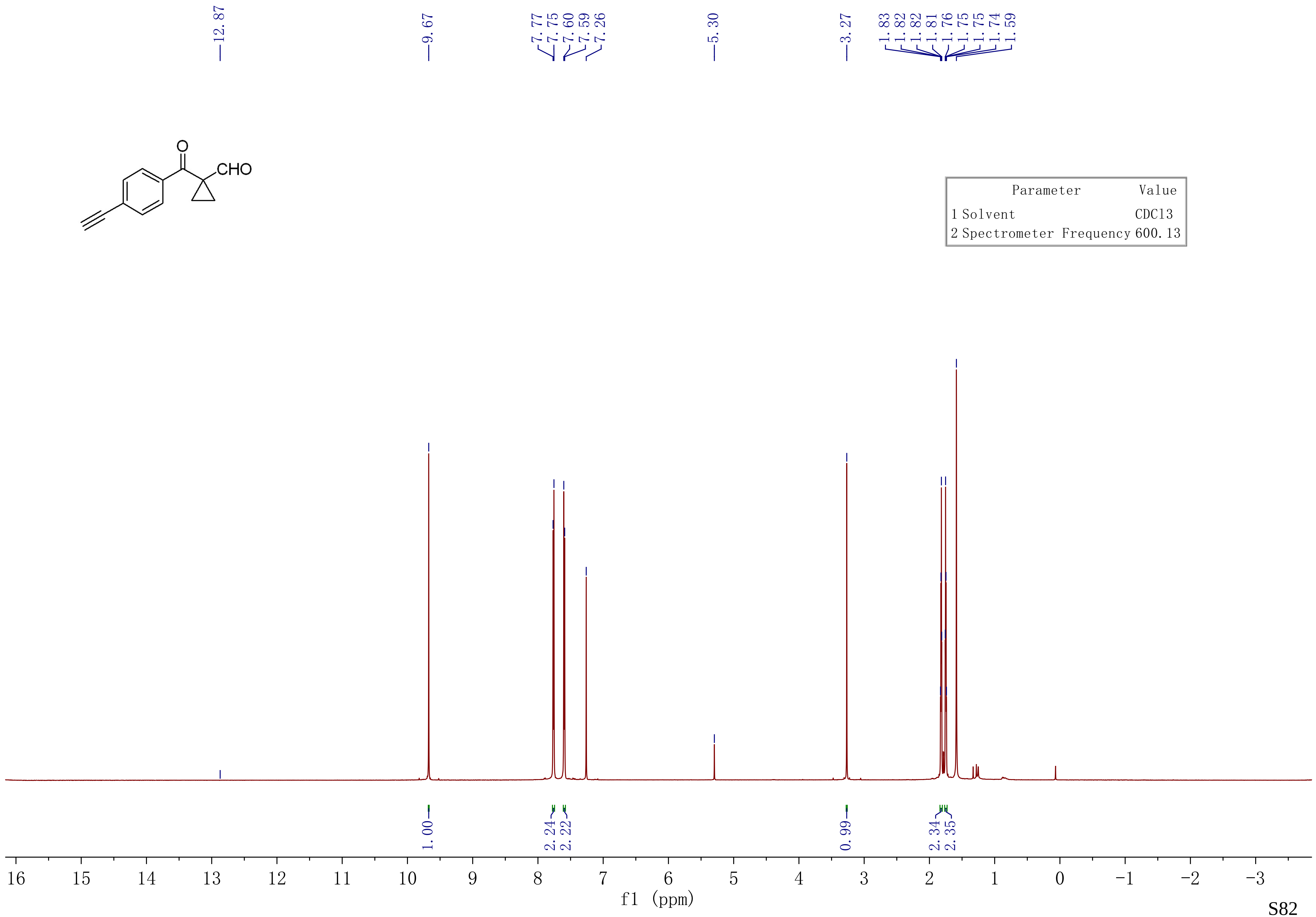
12.87
9.67
7.77
7.75
7.60
7.59
7.26
5.30
3.27
1.83
1.82
1.82
1.81
1.76
1.75
1.75
1.74
1.59
CHO
O
Parameter Value
1 Solvent CDCl3
2 Spectrometer Frequency 600.13
1.00
2.24
2.22
0.99
2.34
2.35
16 15 14 13 12 11 10 9 8 7 6 5 4 3 2 1 0 −1 −2 −3
f1 (ppm)

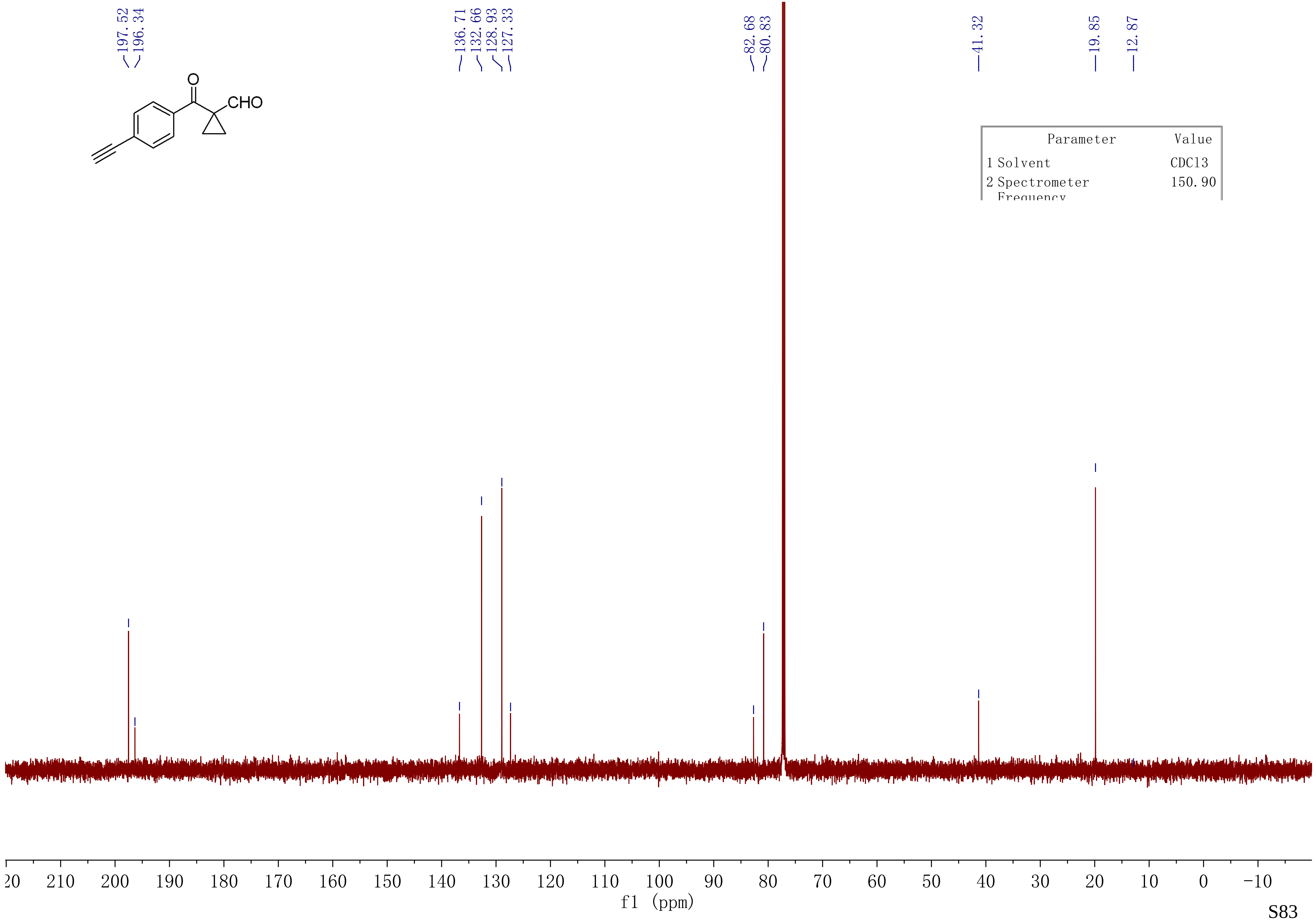
197.52
196.34
136.71
132.66
128.93
127.33
82.68
80.83
41.32
19.85
12.87
CHO
O
Parameter Value
1 Solvent CDCl3
2 Spectrometer Frequency 150.90
f1 (ppm)

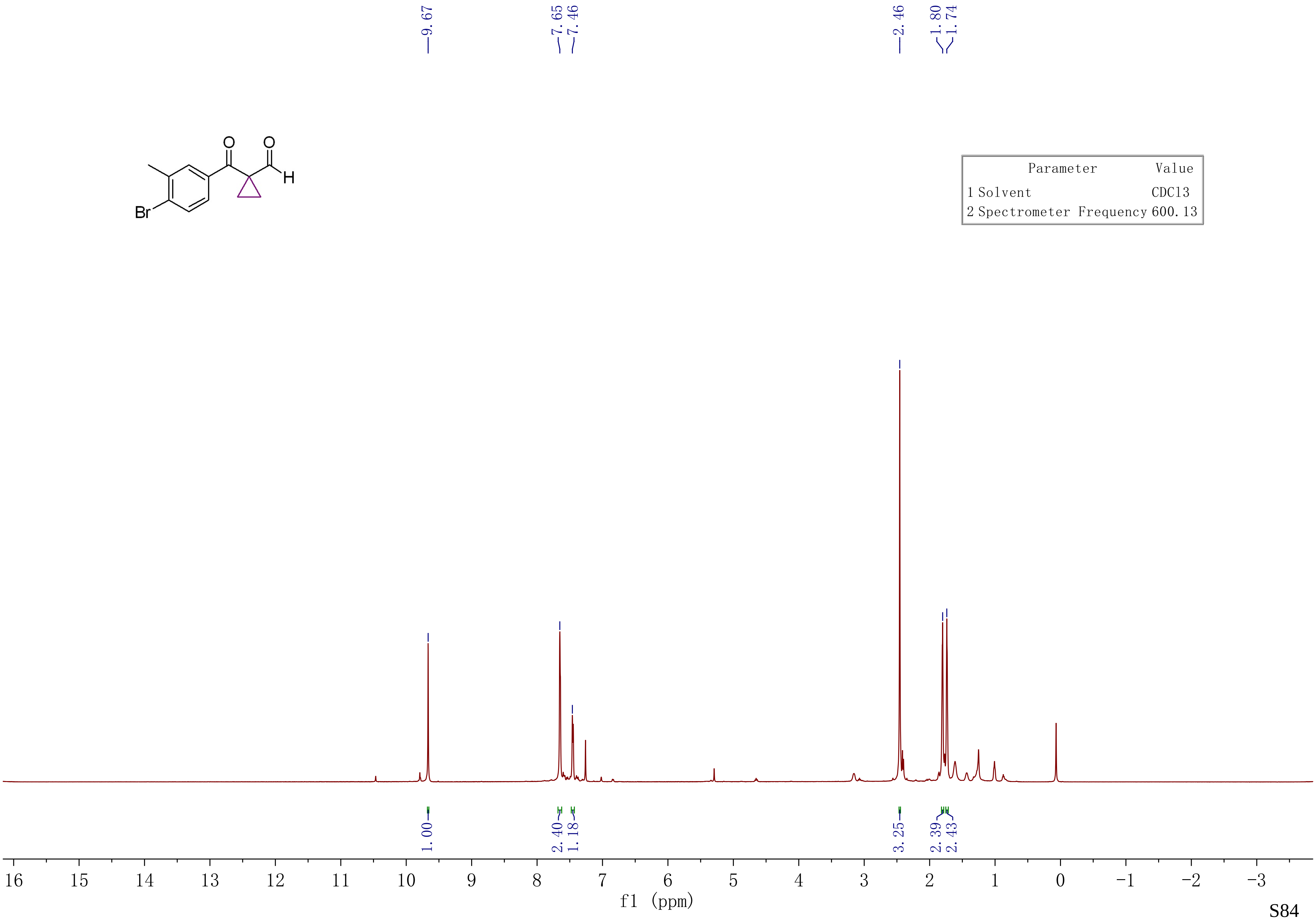

	Parameter	Value
1	Solvent	CDCl3
2	Spectrometer Frequency	600.13

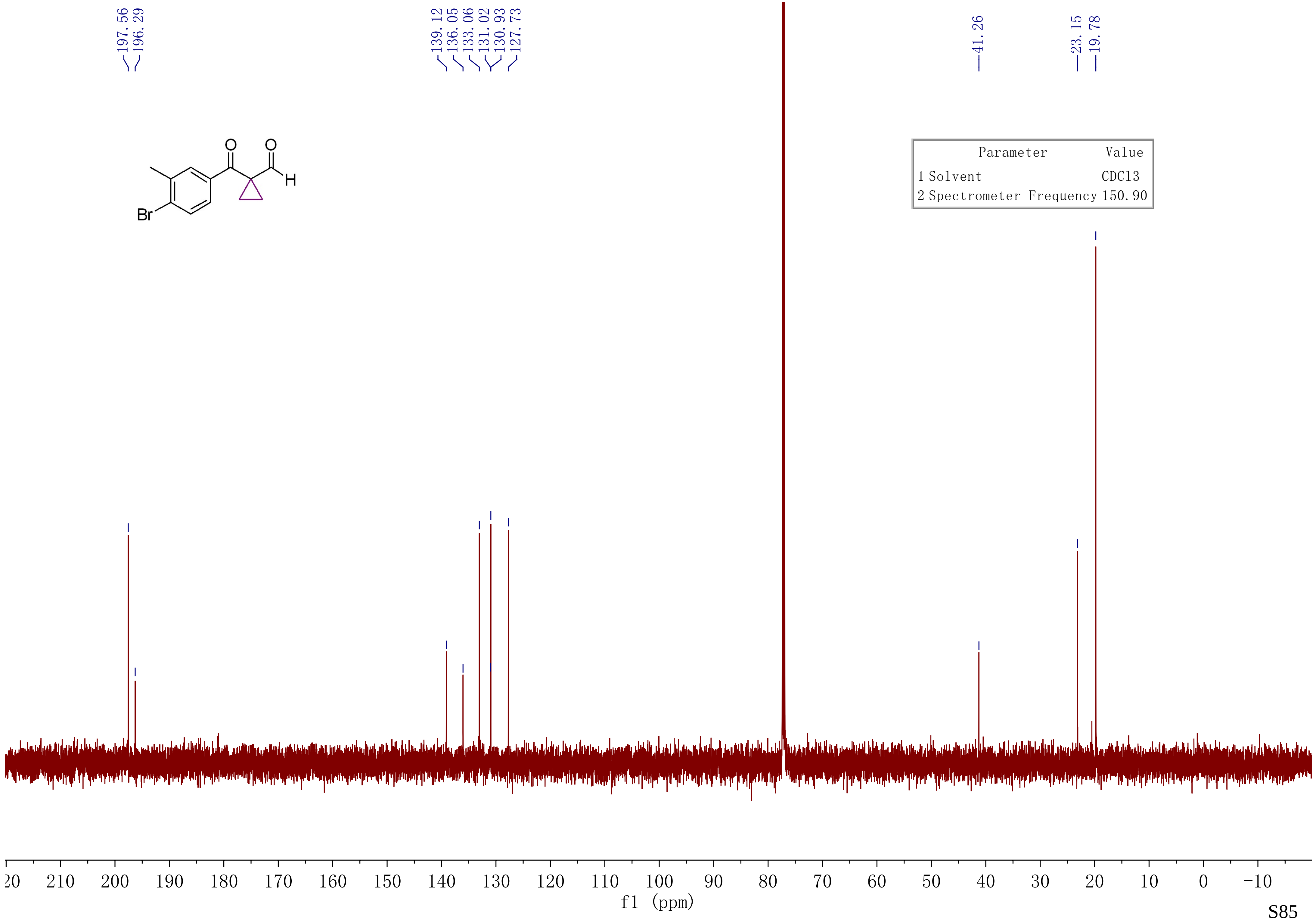
197.56
196.29
139.12
136.05
133.06
131.02
130.93
127.73
41.26
23.15
19.78
Br
O
O
H
Parameter Value
1 Solvent CDCl3
2 Spectrometer Frequency 150.90
f1 (ppm)

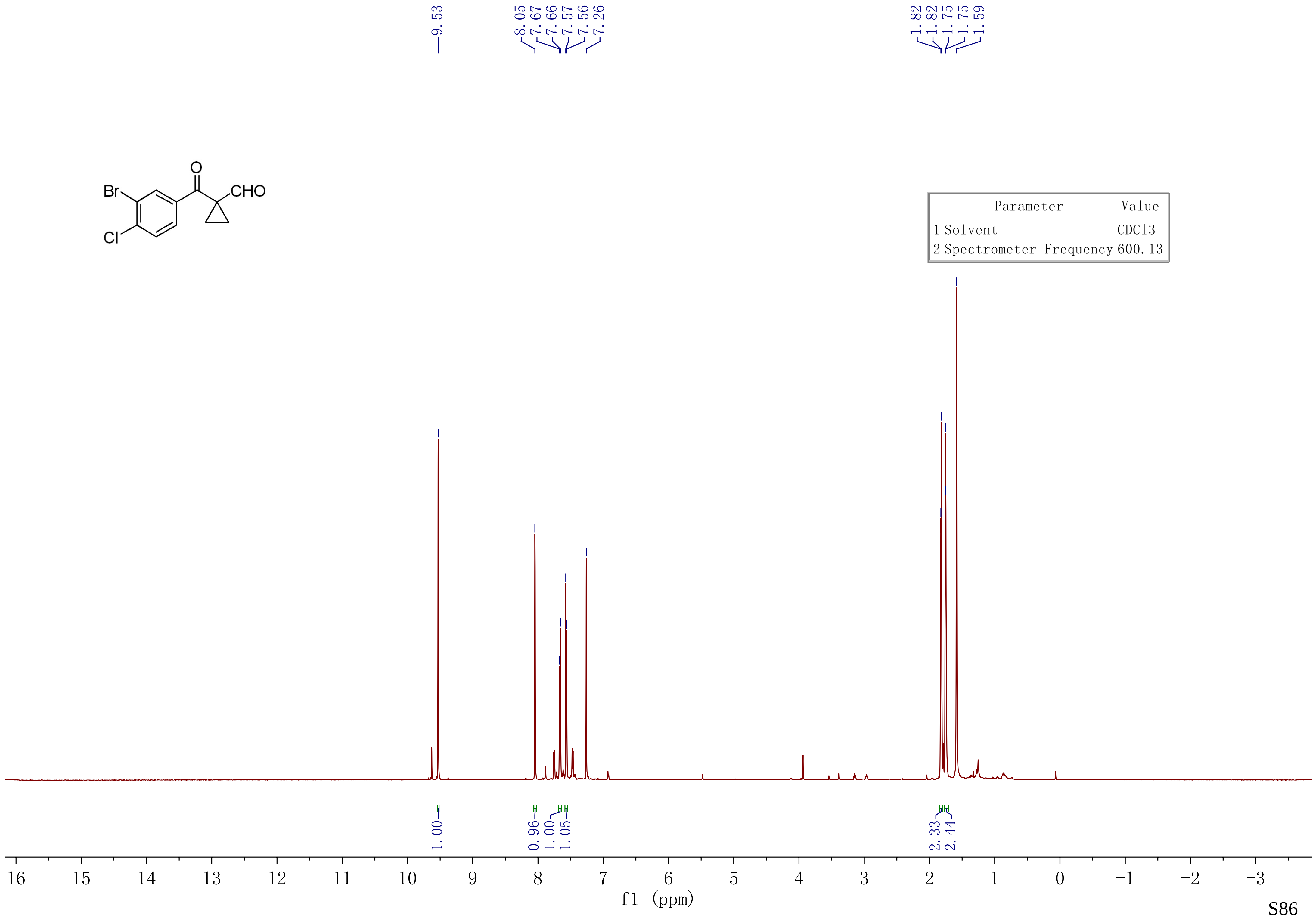
9.53
8.05
7.67
7.66
7.57
7.56
7.26
1.82
1.82
1.75
1.75
1.59
Br
Cl
O
CHO
Parameter Value
1 Solvent CDCl3
2 Spectrometer Frequency 600.13
1.00
0.96
1.00
1.05
2.33
2.44
16 15 14 13 12 11 10 9 8 7 6 5 4 3 2 1 0 −1 −2 −3
f1 (ppm)

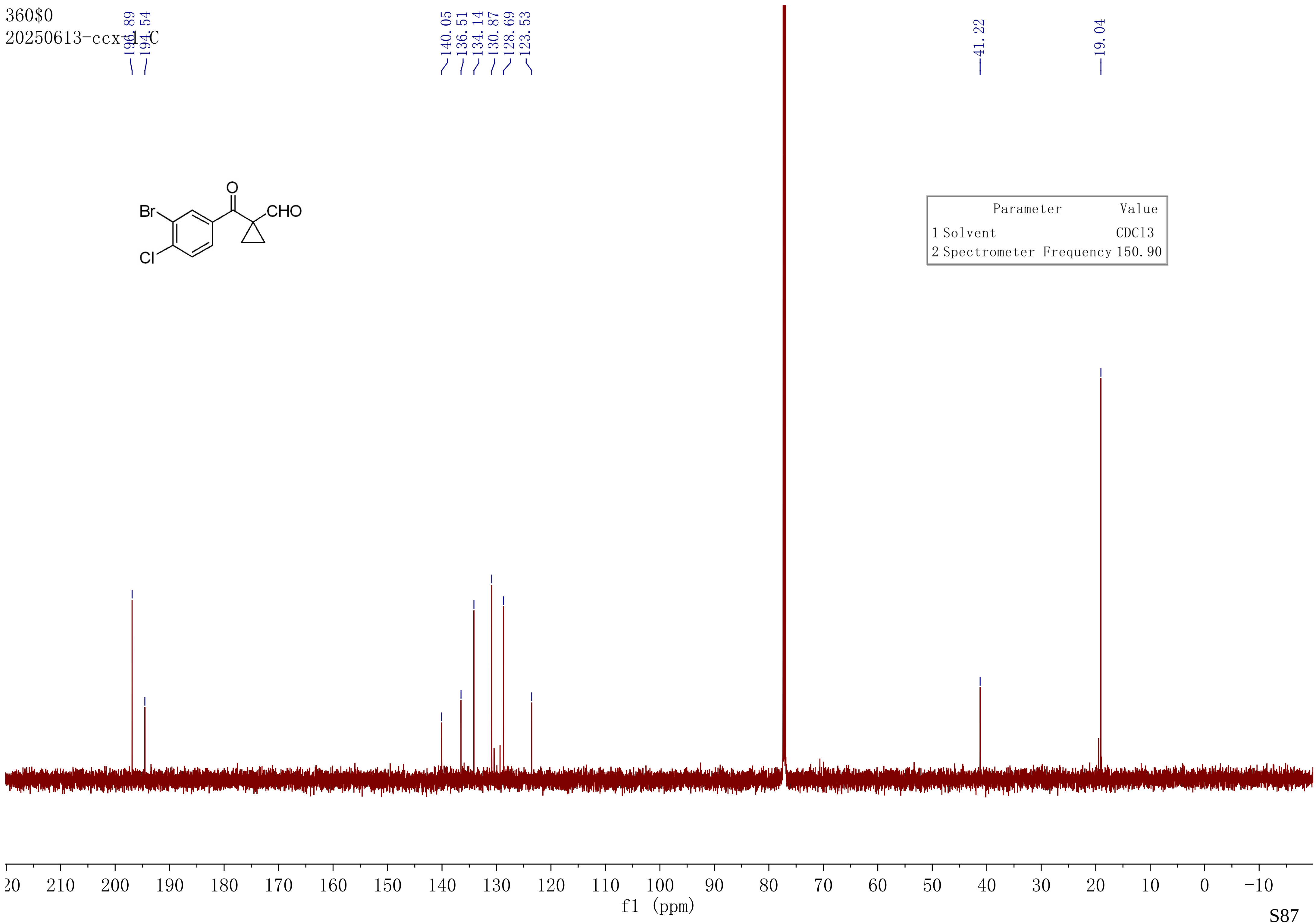
360$0
20250613-ccx-1-C
196.89
194.54
140.05
136.51
134.14
130.87
128.69
123.53
41.22
19.04
O
Br
CHO
Cl
Parameter Value
1 Solvent CDCl3
2 Spectrometer Frequency 150.90
20 210 200 190 180 170 160 150 140 130 120 110 100 90 80 70 60 50 40 30 20 10 0 -10
f1 (ppm)

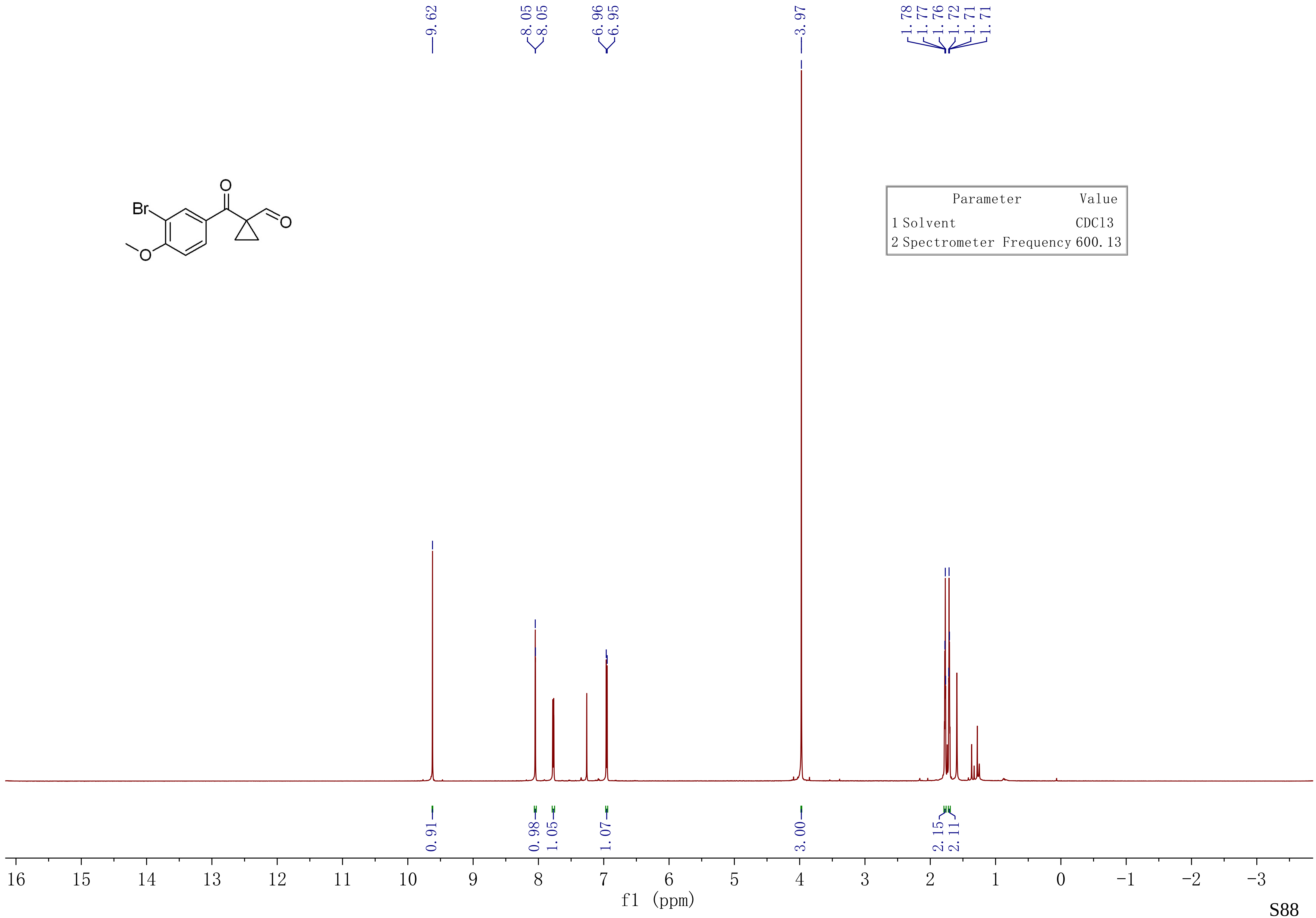
9.62
8.05
8.05
6.96
6.95
3.97
1.78
1.77
1.76
1.72
1.71
1.71
Br
O
O
O
Parameter | Value
1 Solvent | CDCl3
2 Spectrometer Frequency | 600.13
0.91
0.98
1.05
1.07
3.00
2.15
2.11
16 15 14 13 12 11 10 9 8 7 6 5 4 3 2 1 0 −1 −2 −3
f1 (ppm)

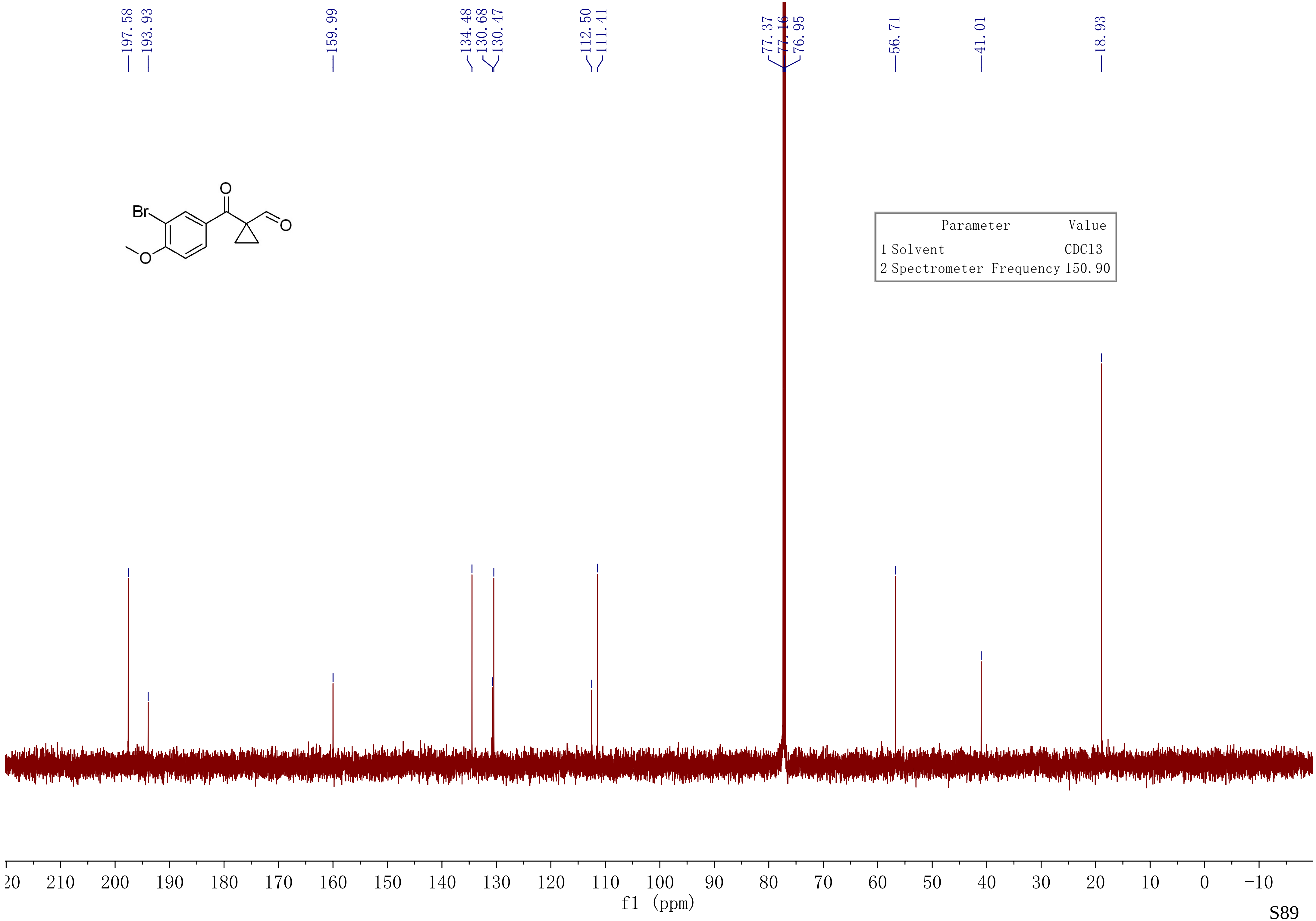
197.58
193.93
159.99
134.48
130.68
130.47
112.50
111.41
77.37
77.16
76.95
56.71
41.01
18.93
Br
O
O
O
Parameter Value
1 Solvent CDCl3
2 Spectrometer Frequency 150.90
220 210 200 190 180 170 160 150 140 130 120 110 100 90 80 70 60 50 40 30 20 10 0 −10
f1 (ppm)

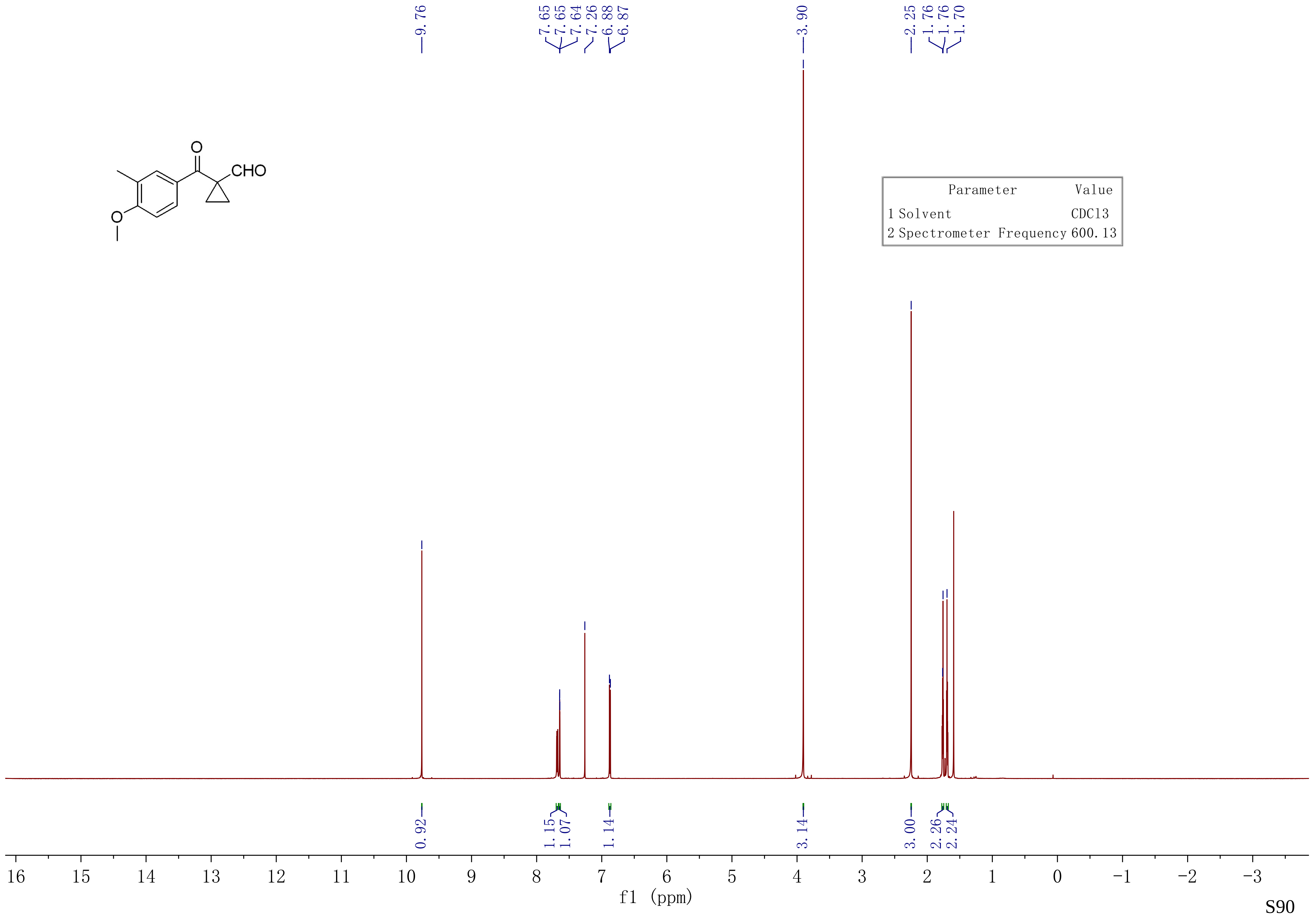
9.76
7.65
7.65
7.64
7.26
6.88
6.87
3.90
2.25
1.76
1.76
1.70
CHO
O
Parameter Value
1 Solvent CDCl3
2 Spectrometer Frequency 600.13
0.92
1.15
1.07
1.14
3.14
3.00
2.26
2.24
16 15 14 13 12 11 10 9 8 7 6 5 4 3 2 1 0 −1 −2 −3
f1 (ppm)

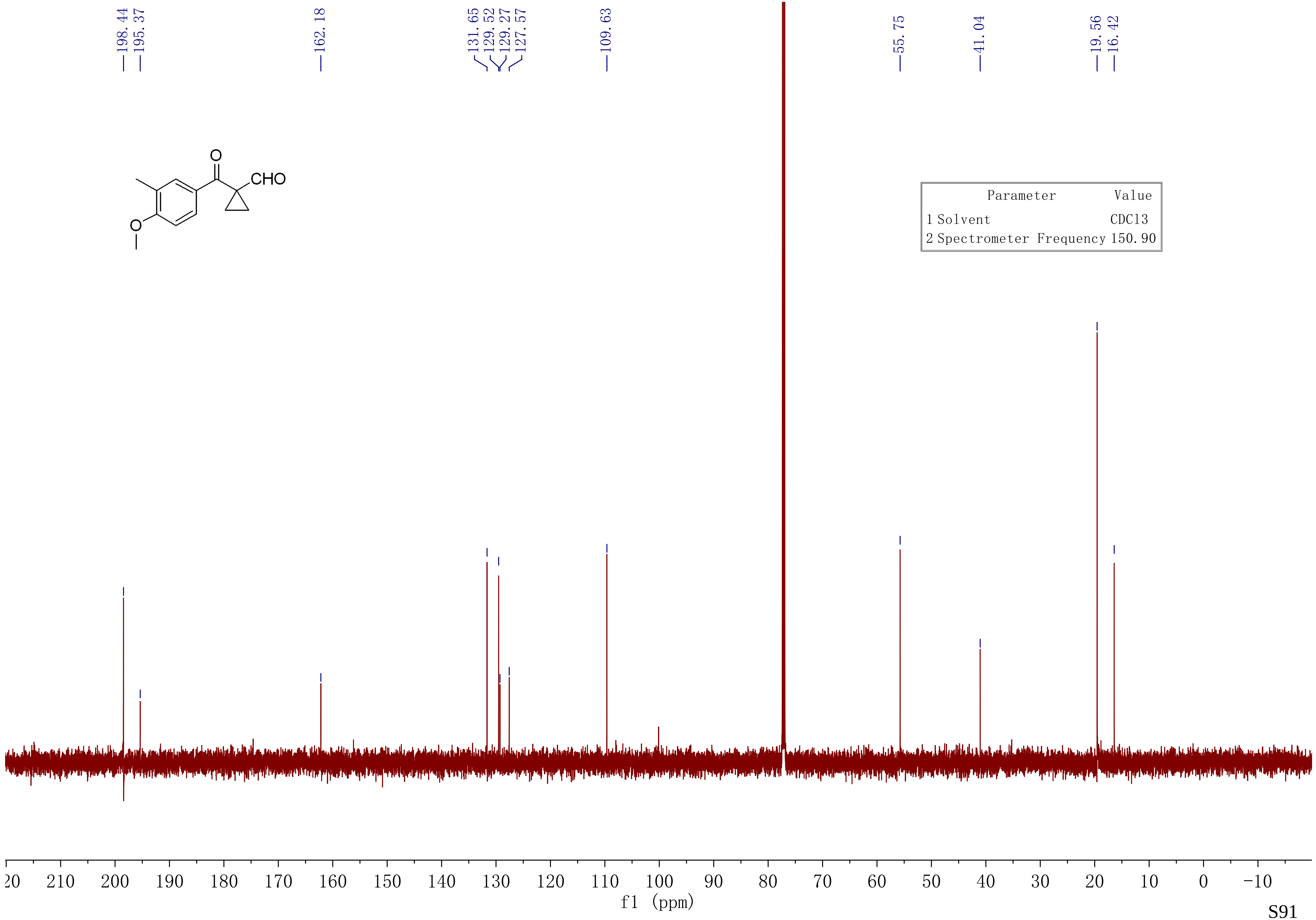

	Parameter	Value
1	Solvent	CDCl3
2	Spectrometer Frequency	150.90

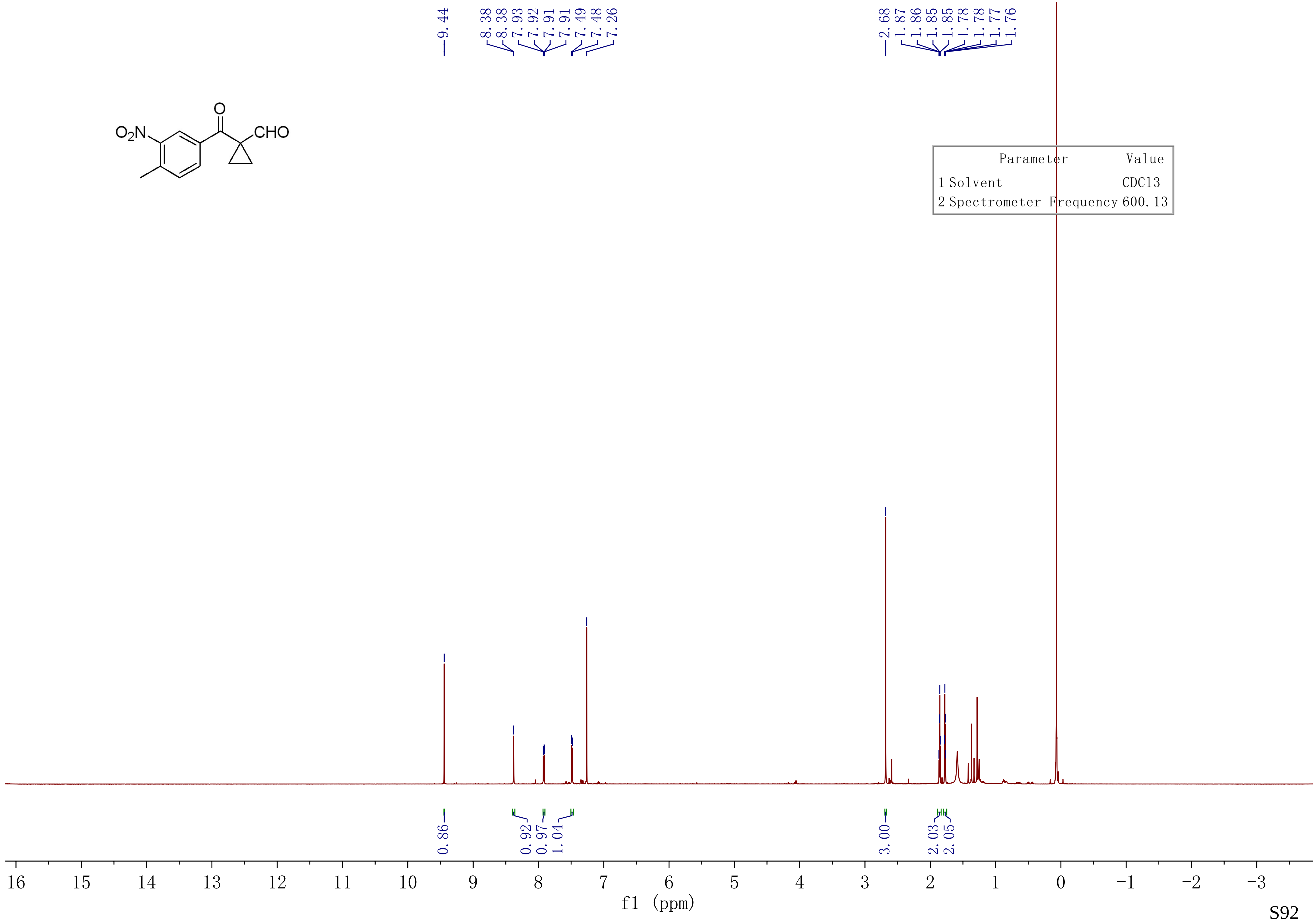

9.44
8.38
8.38
7.93
7.92
7.91
7.91
7.49
7.48
7.26
2.68
1.87
1.86
1.85
1.85
1.78
1.78
1.77
1.76
Parameter
Value
1 Solvent
CDCl3
2 Spectrometer Frequency
600.13
0.86
0.92
0.97
1.04
3.00
2.03
2.05
f1 (ppm)

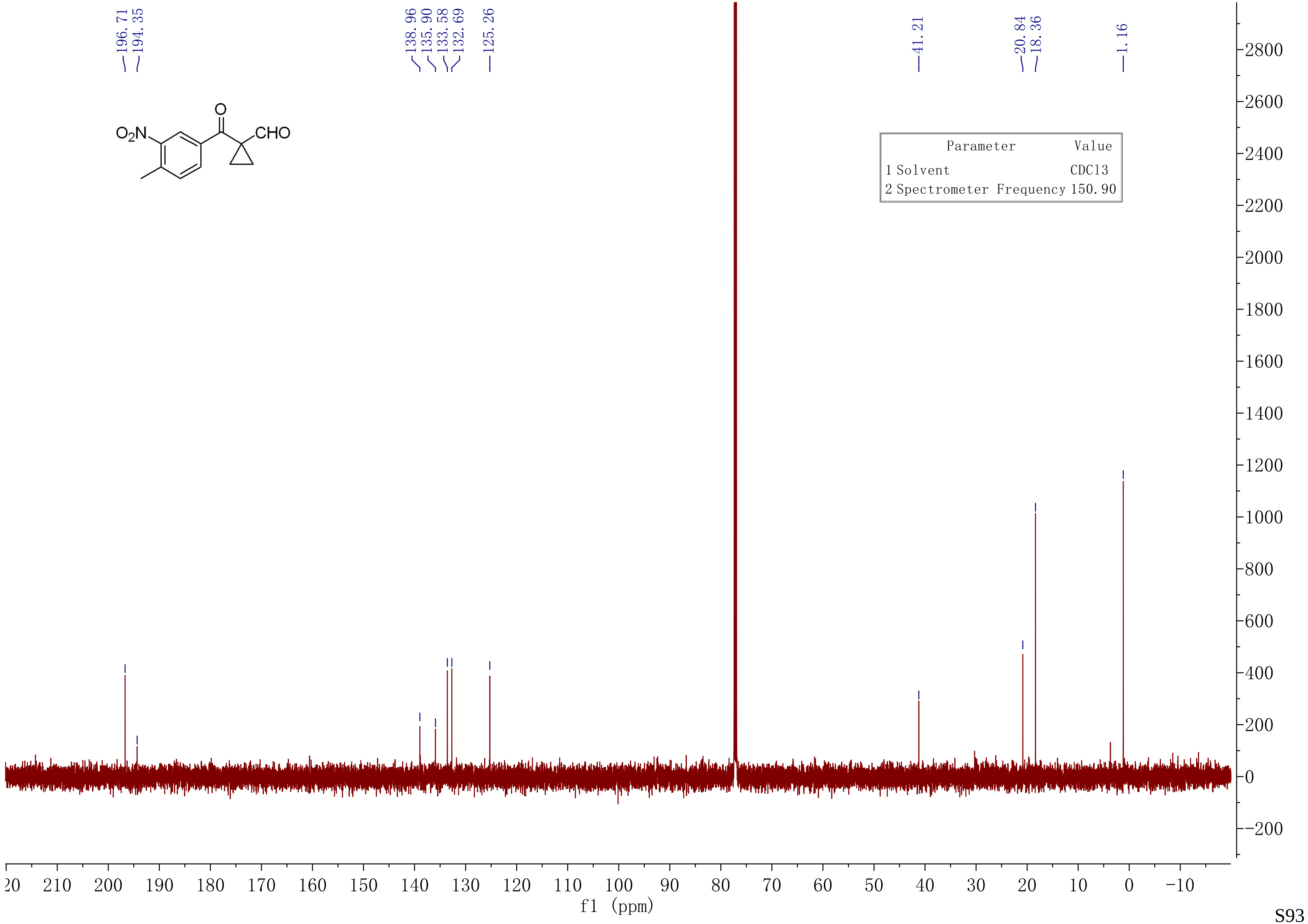
196.71
194.35
138.96
135.90
133.58
132.69
125.26
41.21
20.84
18.36
1.16
O
O_2N
CHO
Parameter Value
1 Solvent CDCl3
2 Spectrometer Frequency 150.90
f1 (ppm)

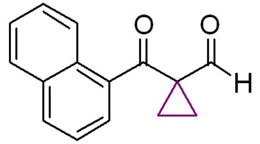

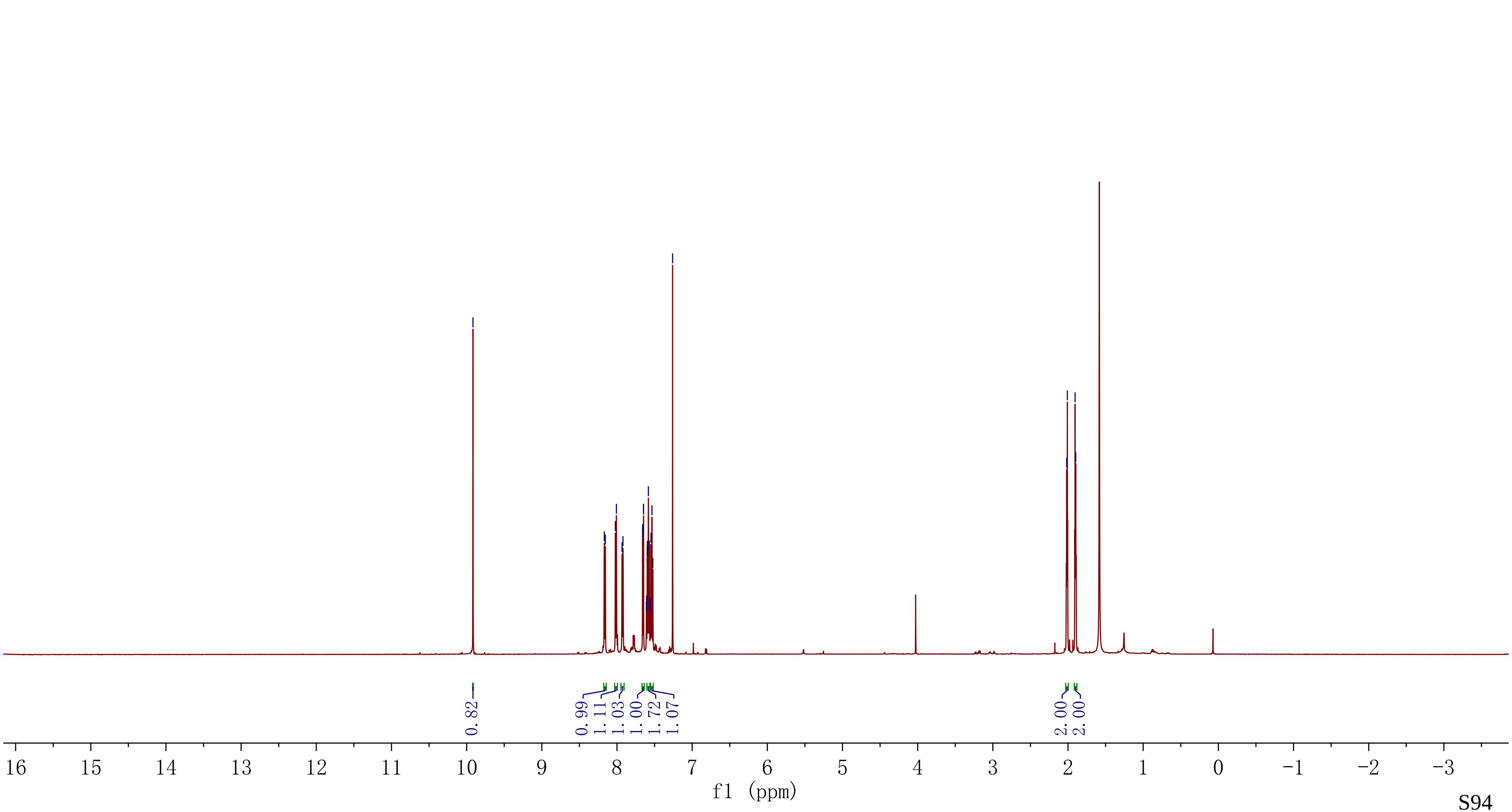

Parameter	Value
1 Solvent	CDCl3
2 Spectrometer Frequency	600.13

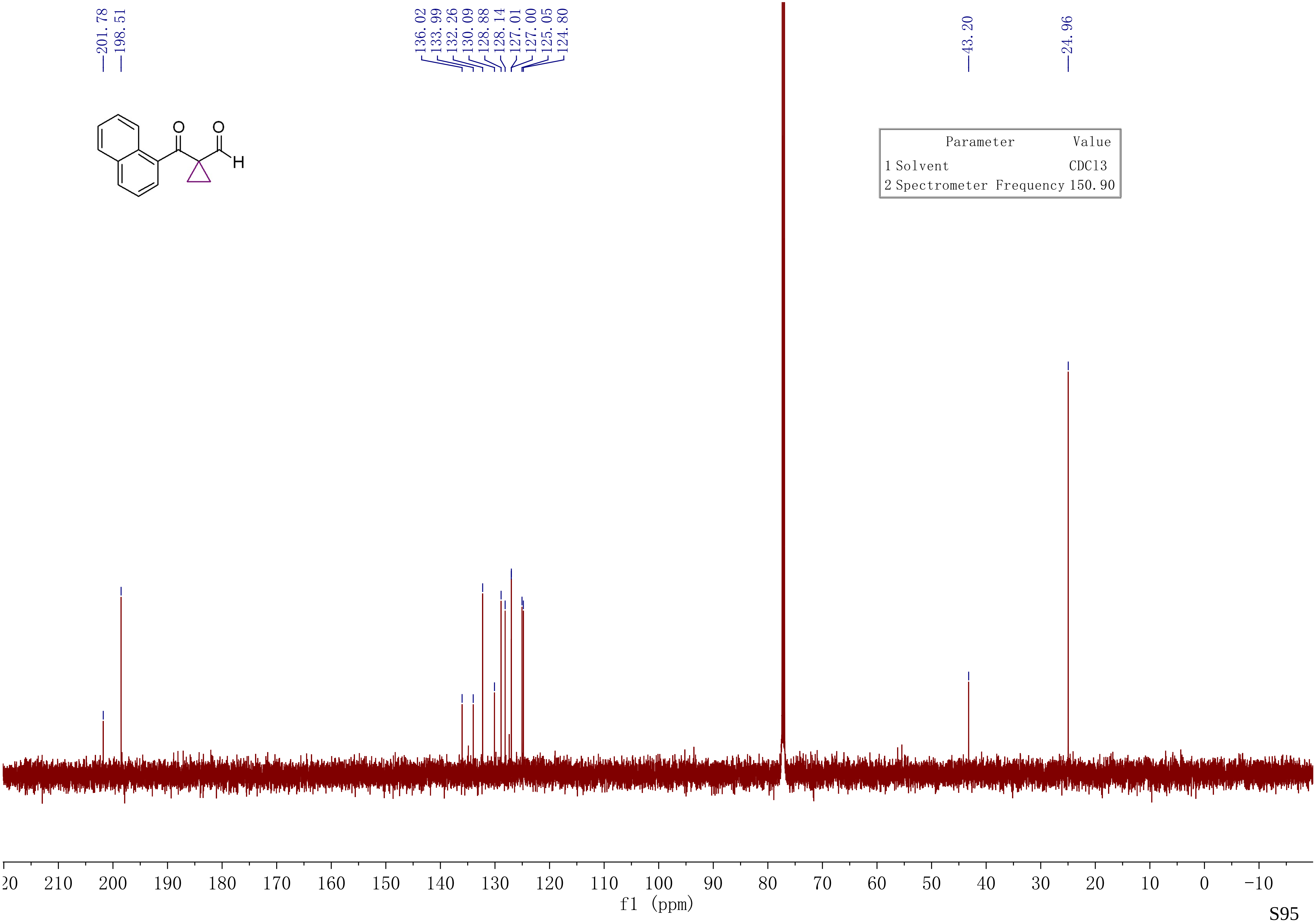
201.78
198.51
136.02
133.99
132.26
130.09
128.88
128.14
127.01
127.00
125.05
124.80
43.20
24.96
O
O
H
Parameter Value
1 Solvent CDCl3
2 Spectrometer Frequency 150.90
20 210 200 190 180 170 160 150 140 130 120 110 100 90 80 70 60 50 40 30 20 10 0 −10
f1 (ppm)

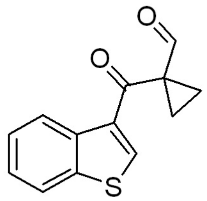

	Parameter	Value
1	Solvent	CDCl3
2	Spectrometer Frequency	600.13

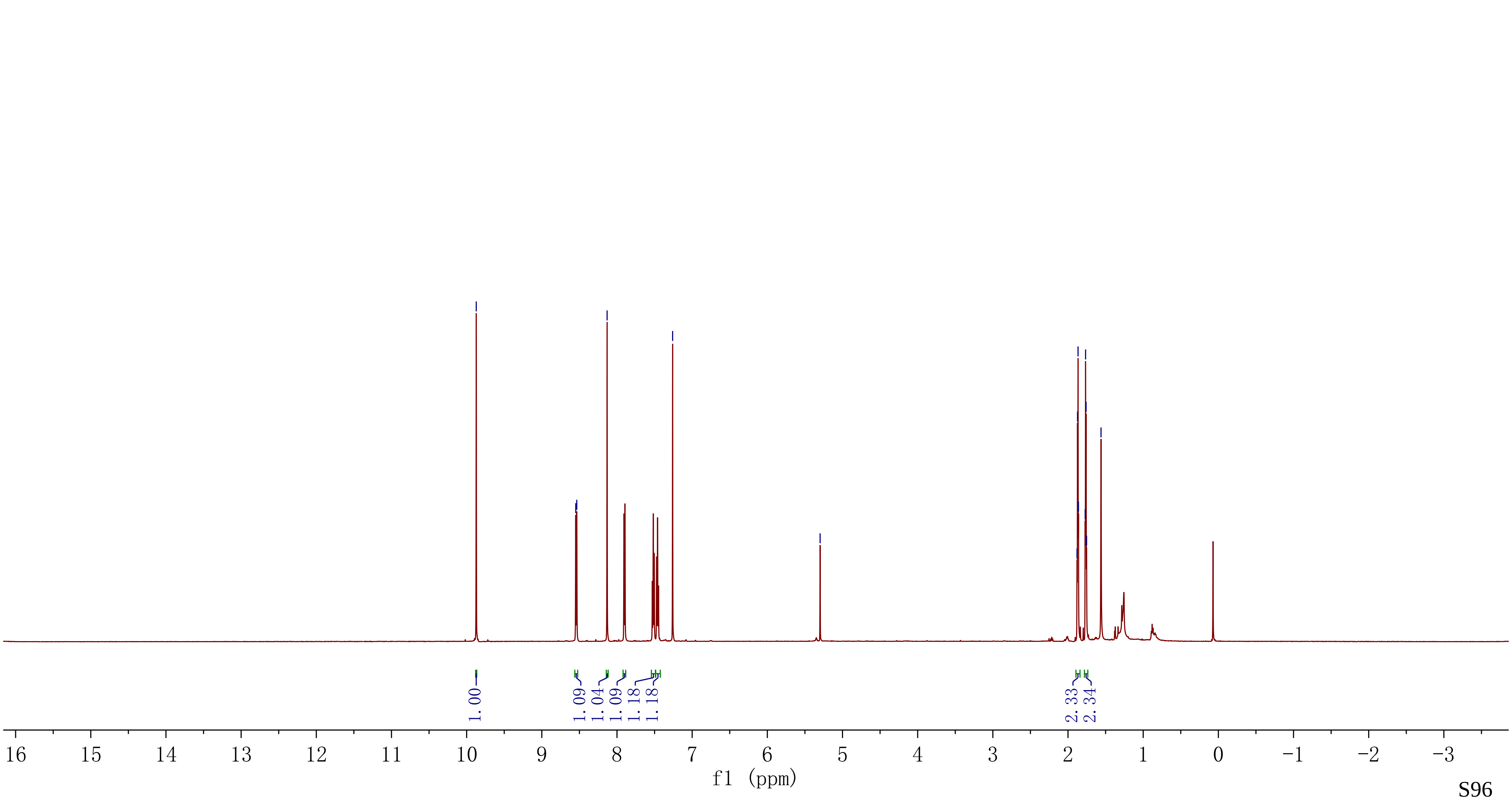

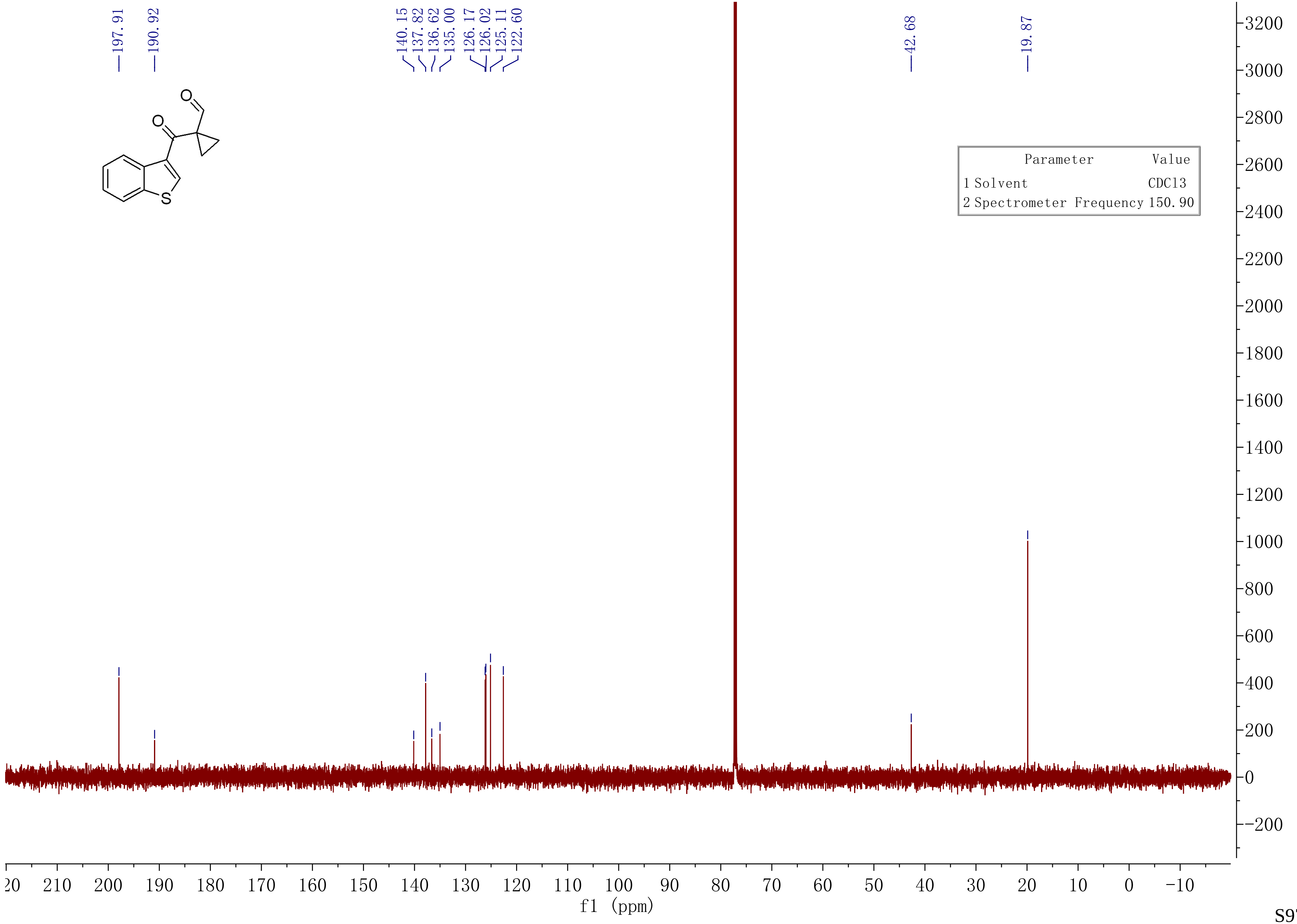

197.91
190.92
140.15
137.82
136.62
135.00
126.17
126.02
125.11
122.60
42.68
19.87
Parameter Value
1 Solvent CDCl3
2 Spectrometer Frequency 150.90
f1 (ppm)

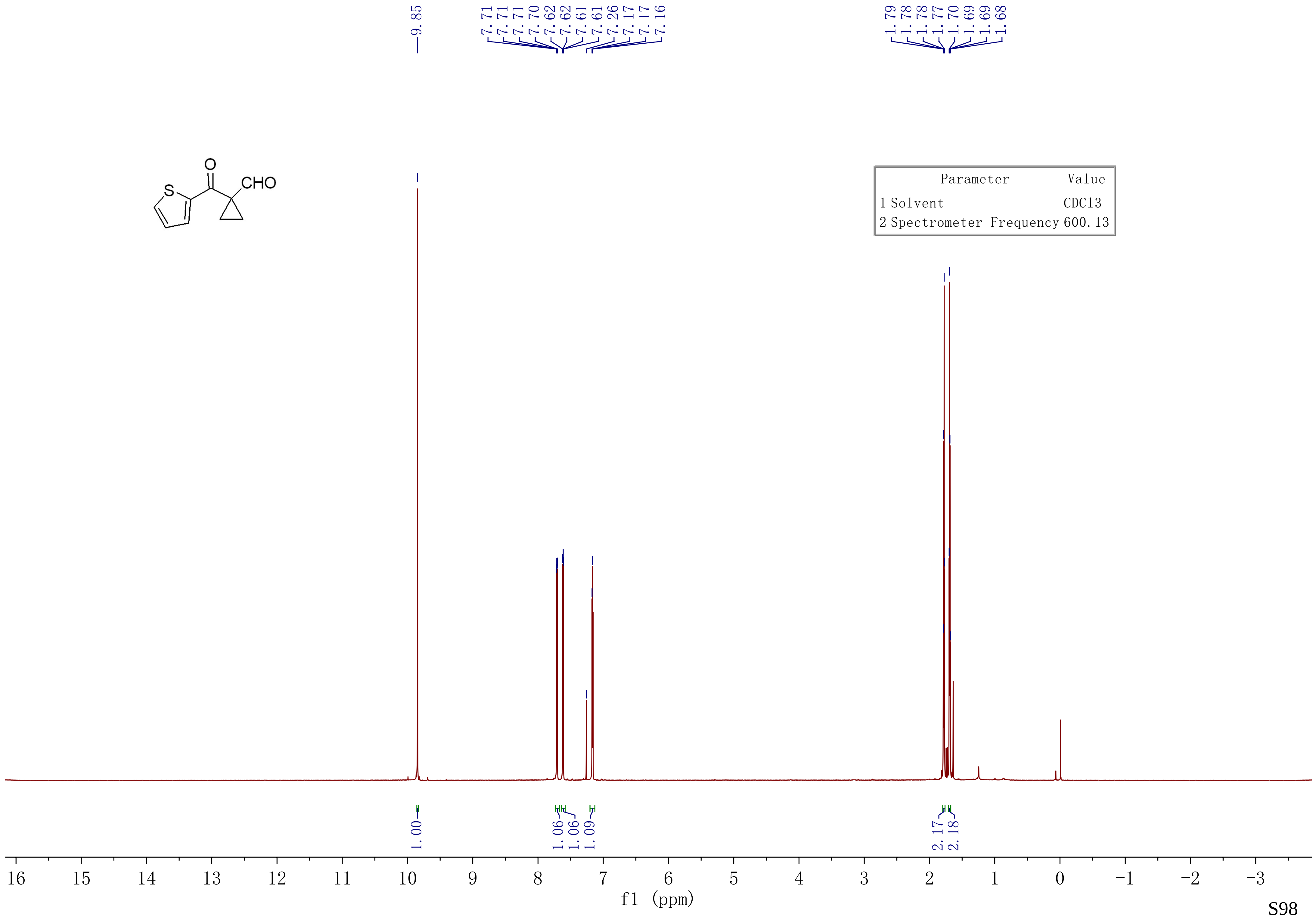
9.85
7.71
7.71
7.71
7.70
7.62
7.62
7.61
7.61
7.26
7.17
7.17
7.16
1.79
1.78
1.78
1.77
1.70
1.69
1.69
1.68
S
O
CHO
Parameter Value
1 Solvent CDCl3
2 Spectrometer Frequency 600.13
1.00
1.06
1.06
1.09
2.17
2.18
16 15 14 13 12 11 10 9 8 7 6 5 4 3 2 1 0 −1 −2 −3
f1 (ppm)

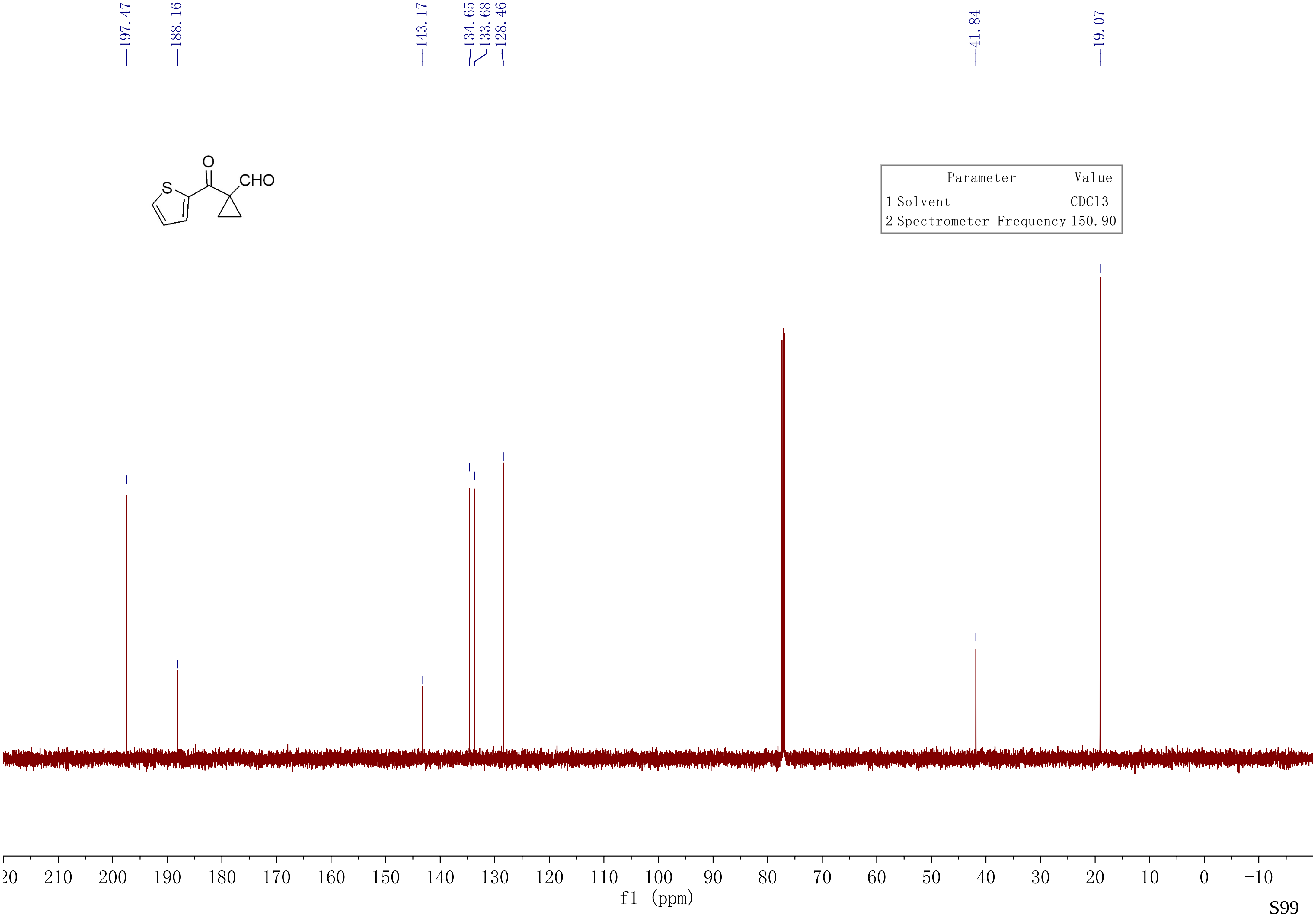

Parameter	Value
1 Solvent	CDCl3
2 Spectrometer Frequency	150.90

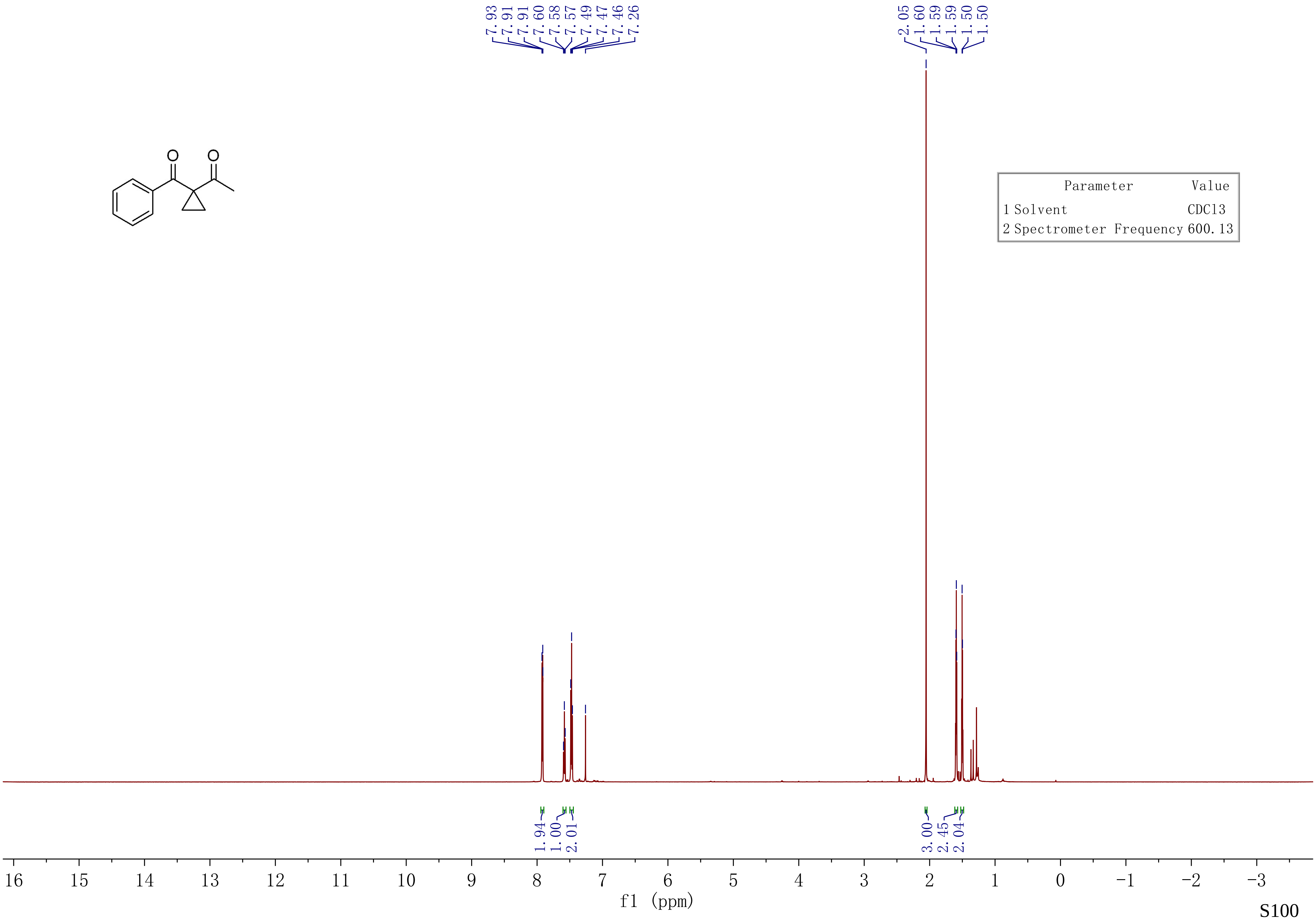
7.93
7.91
7.91
7.60
7.58
7.57
7.49
7.47
7.46
7.26
2.05
1.60
1.59
1.59
1.50
1.50
Parameter Value
1 Solvent CDCl3
2 Spectrometer Frequency 600.13
1.94
1.00
2.01
3.00
2.45
2.04
16 15 14 13 12 11 10 9 8 7 6 5 4 3 2 1 0 −1 −2 −3
f1 (ppm)

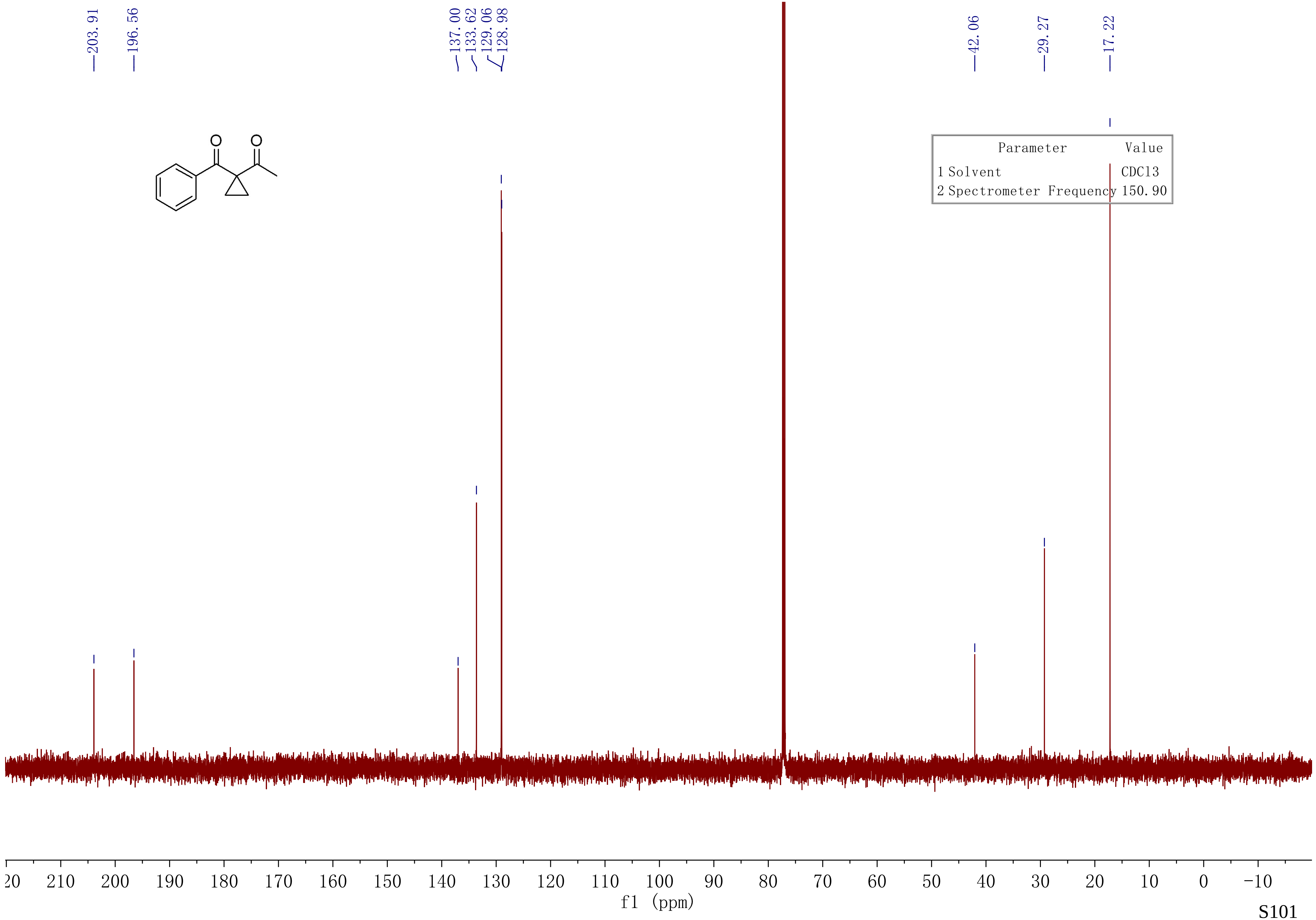
203.91
196.56
137.00
133.62
129.06
128.98
42.06
29.27
17.22
Parameter Value
1 Solvent CDCl3
2 Spectrometer Frequency 150.90
f1 (ppm)

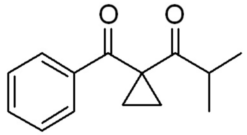

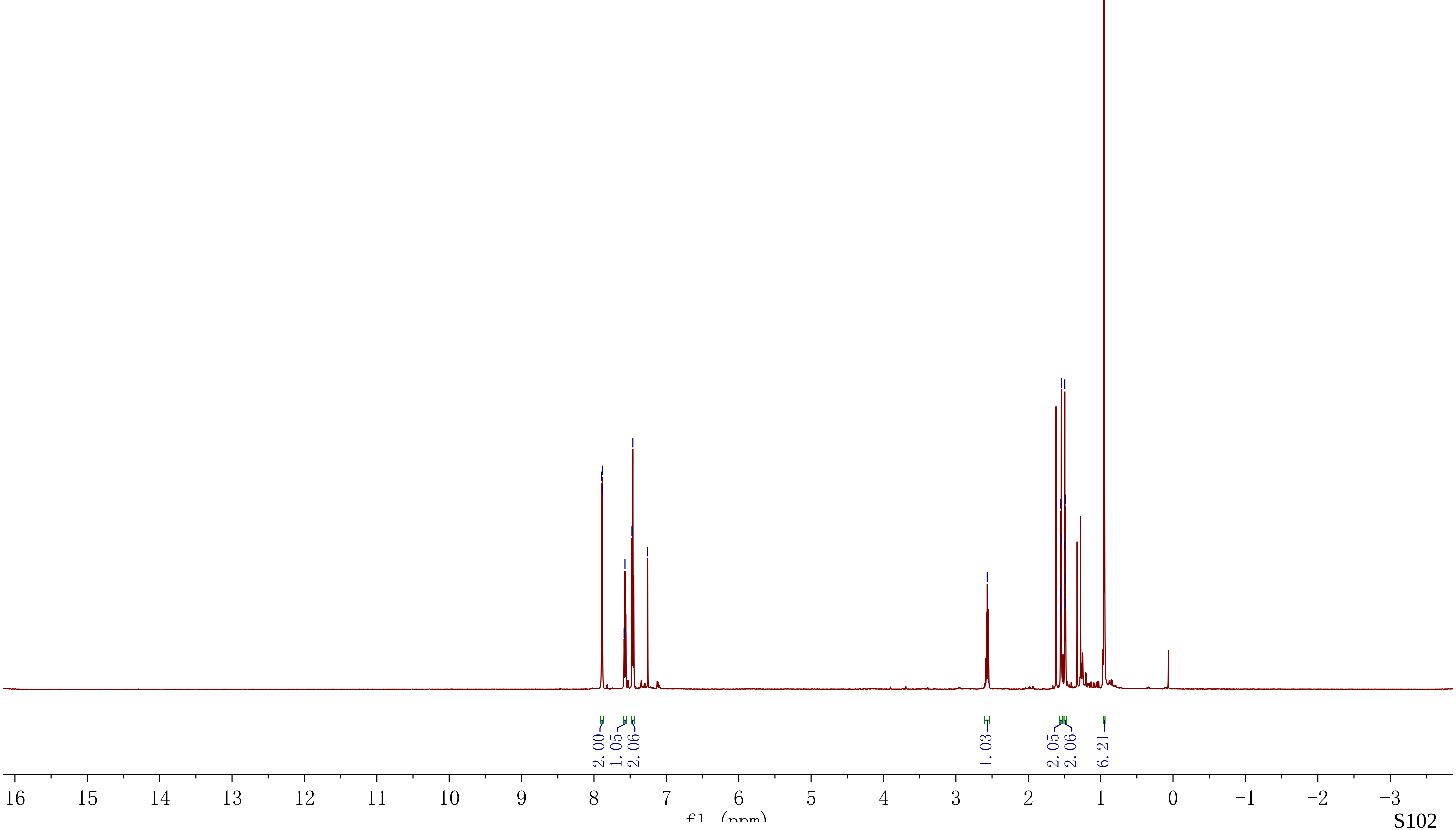

	Parameter	Value
1	Solvent	CDCl3
2	Spectrometer Frequency	600.13

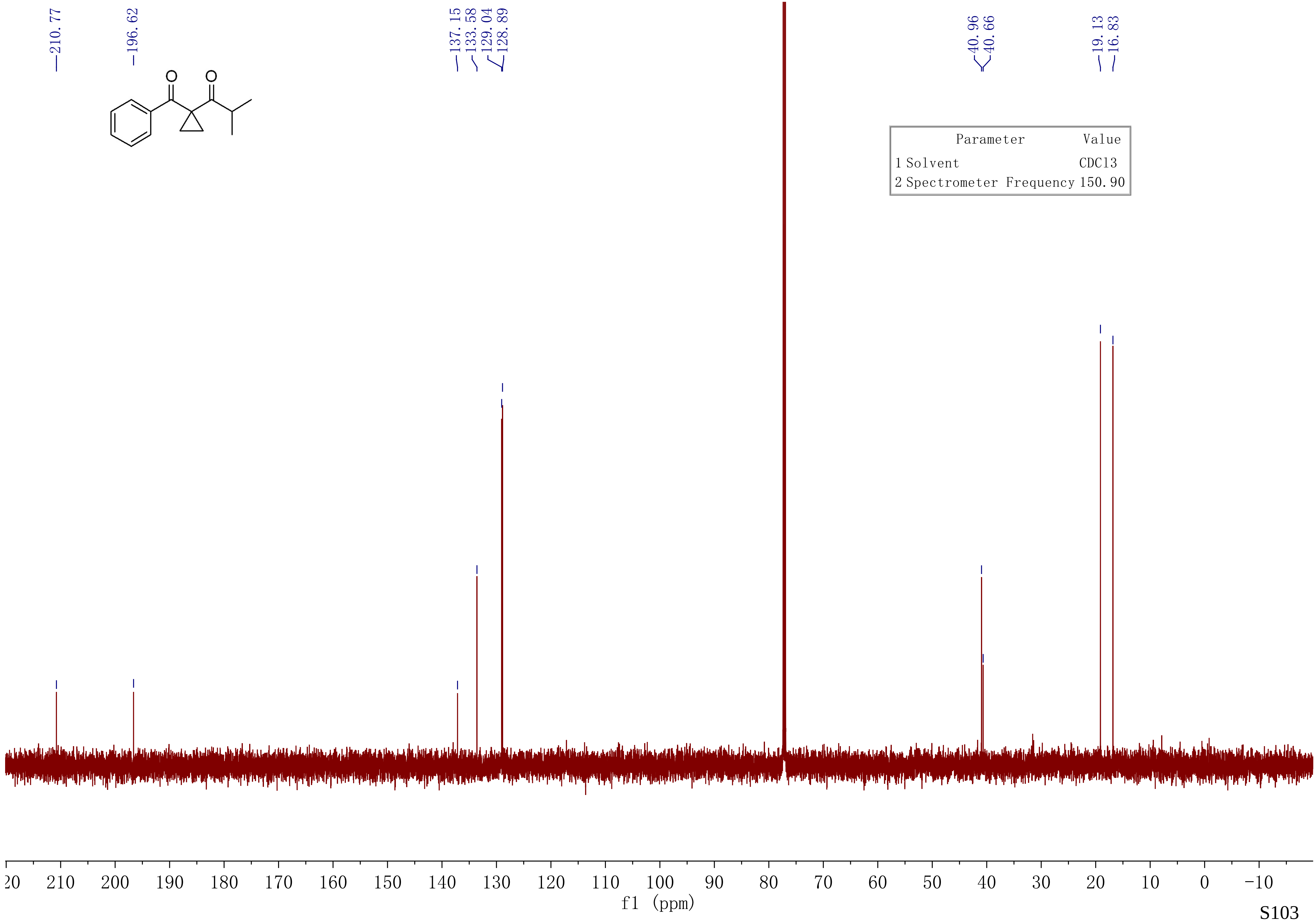

210.77
196.62
137.15
133.58
129.04
128.89
40.96
40.66
19.13
16.83
Parameter
Value
1 Solvent
CDCl3
2 Spectrometer Frequency
150.90
220
210
200
190
180
170
160
150
140
130
120
110
100
90
80
70
60
50
40
30
20
10
0
−10
f1 (ppm)

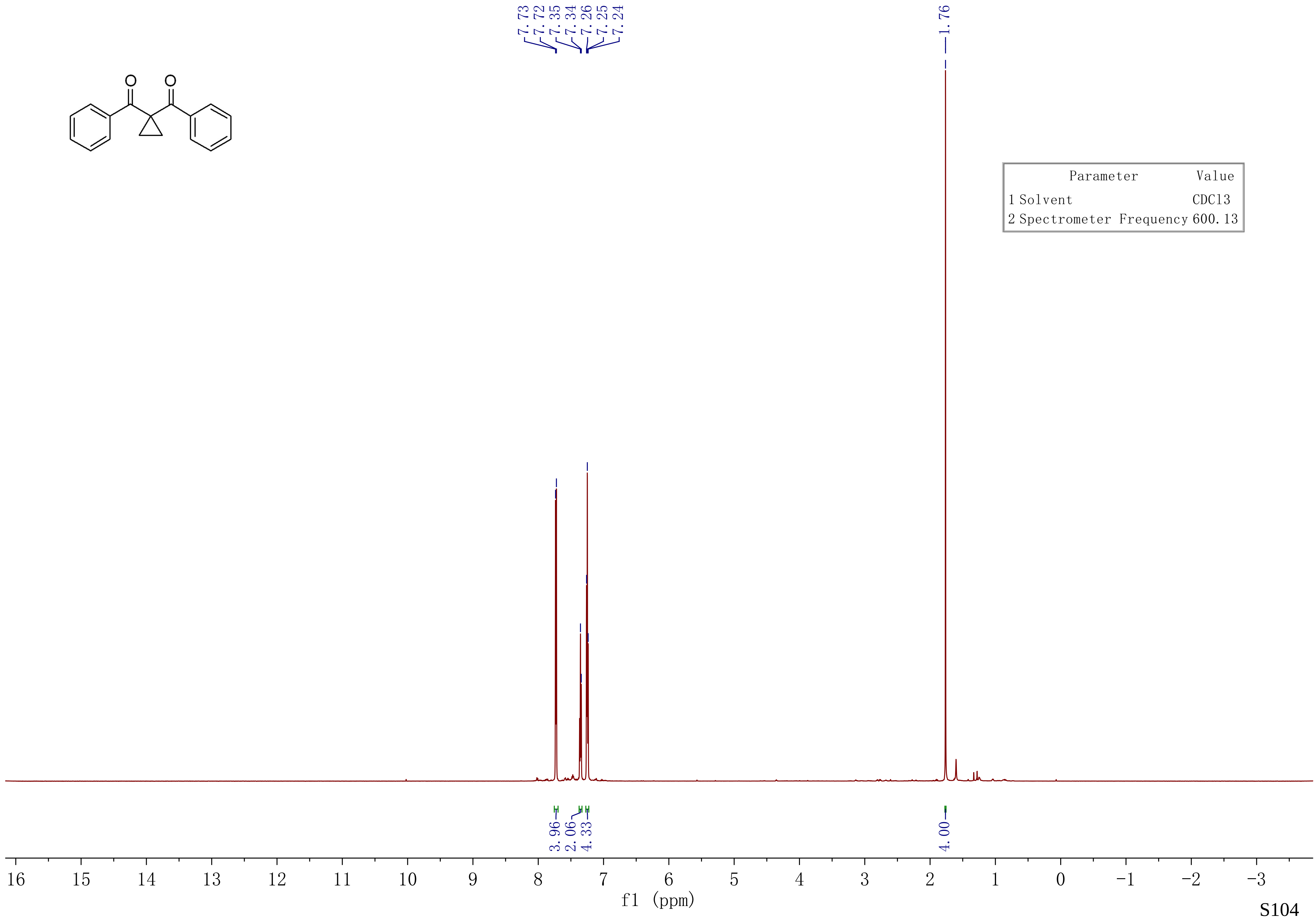

	Parameter	Value
1	Solvent	CDCl3
2	Spectrometer Frequency	600.13

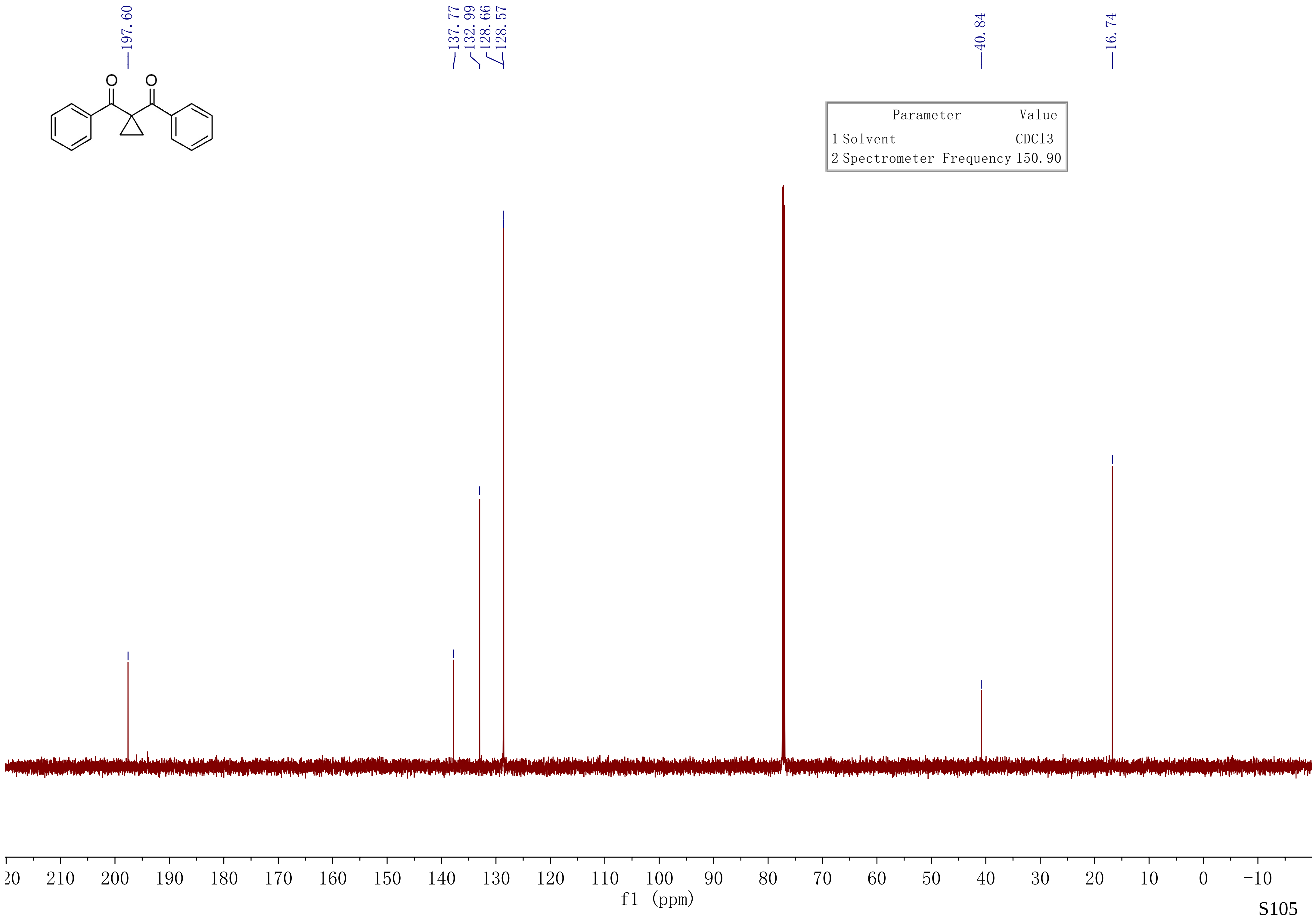
197.60
137.77
132.99
128.66
128.57
40.84
16.74
Parameter
Value
1 Solvent
CDCl3
2 Spectrometer Frequency
150.90
20
210
200
190
180
170
160
150
140
130
120
110
100
90
80
70
60
50
40
30
20
10
0
−10
f1 (ppm)

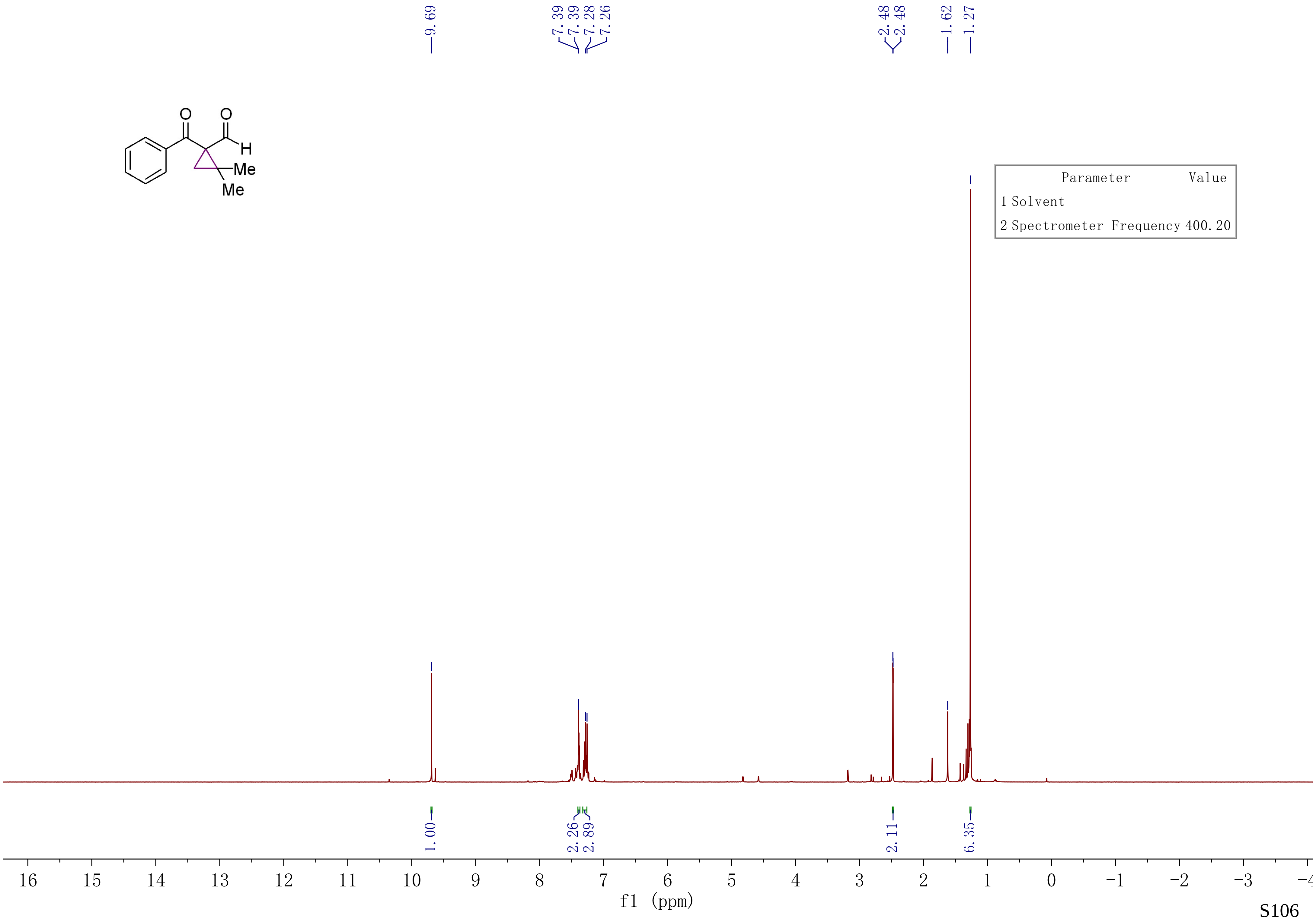
9.69
7.39
7.39
7.28
7.26
2.48
2.48
1.62
1.27
O
O
H
Me
Me
Parameter Value
1 Solvent
2 Spectrometer Frequency 400.20
1.00
2.26
2.89
2.11
6.35
16 15 14 13 12 11 10 9 8 7 6 5 4 3 2 1 0 −1 −2 −3 −4
f1 (ppm)

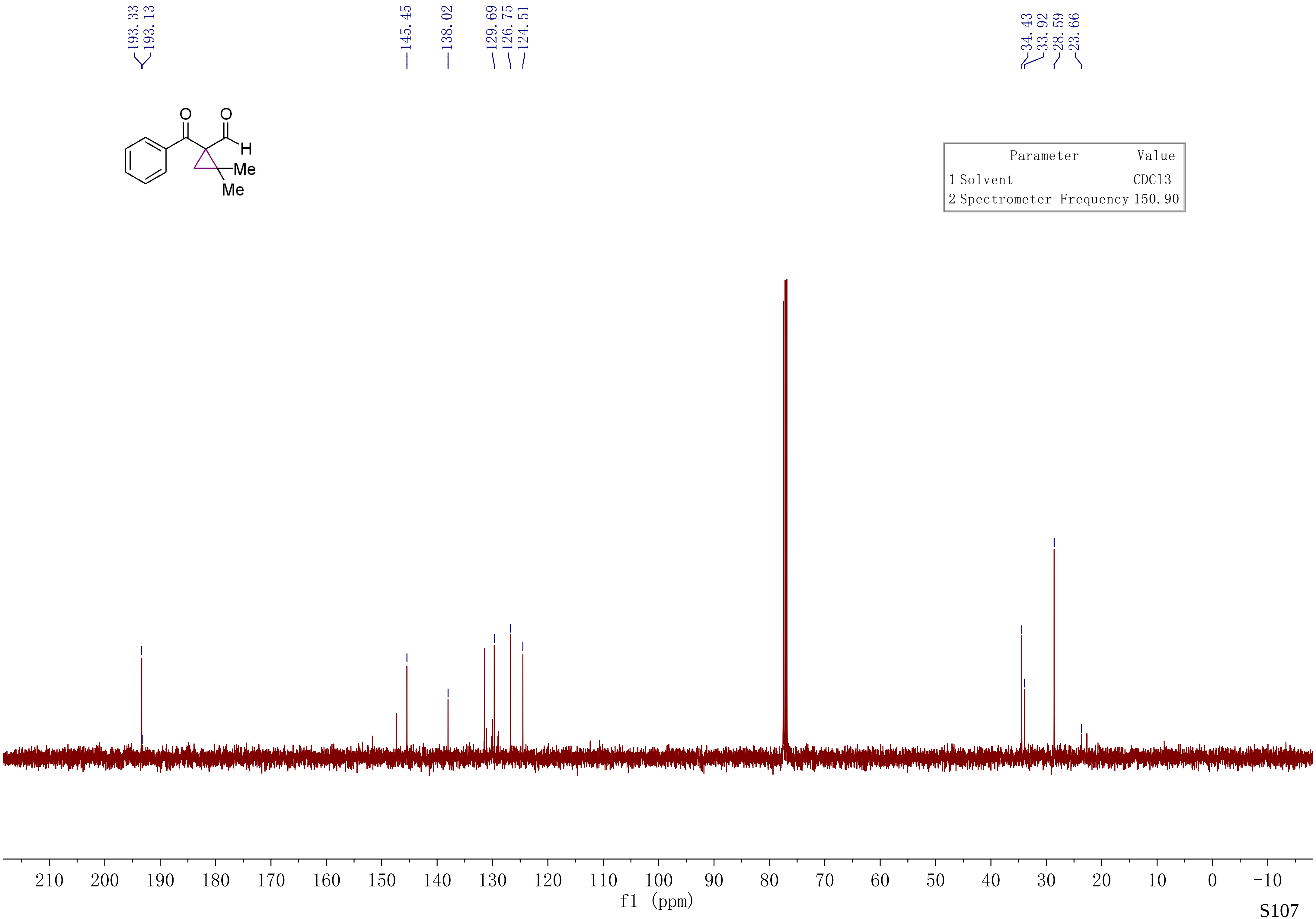

	Parameter	Value
1	Solvent	CDCl3
2	Spectrometer Frequency	150.90

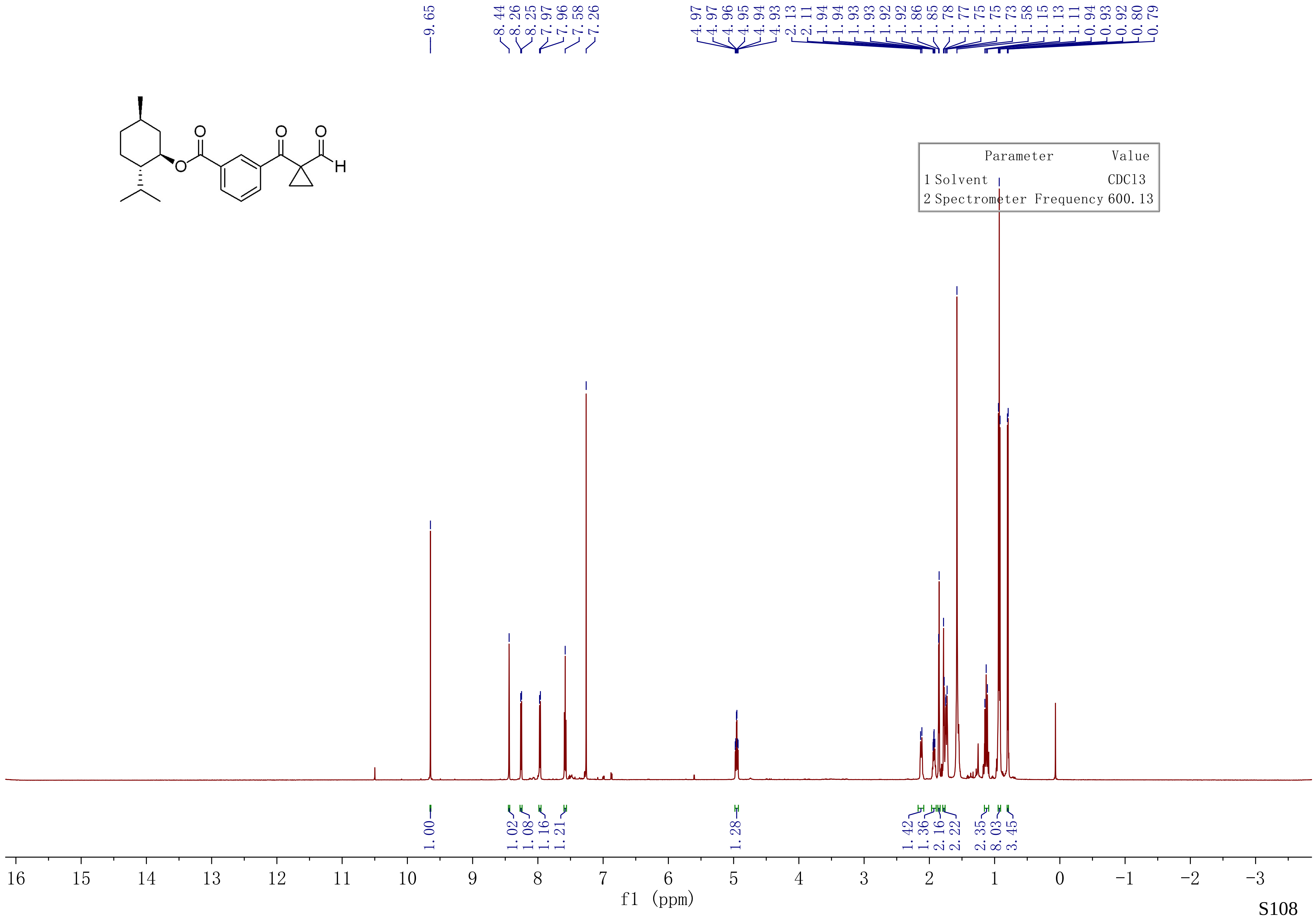
9.65
8.44 8.26 8.25 7.97 7.96 7.58 7.26
4.97 4.97 4.96 4.95 4.94 4.93 2.13 2.11 1.94 1.94 1.93 1.93 1.92 1.92 1.86 1.85 1.78 1.77 1.75 1.75 1.73 1.58 1.15 1.13 1.11 0.94 0.93 0.92 0.80 0.79
Parameter Value
1 Solvent CDCl3
2 Spectrometer Frequency 600.13
1.00
1.02 1.08 1.16 1.21
1.28
1.42 1.36 2.16 2.22 2.35 8.03 3.45
16 15 14 13 12 11 10 9 8 7 6 5 4 3 2 1 0 −1 −2 −3
f1 (ppm)

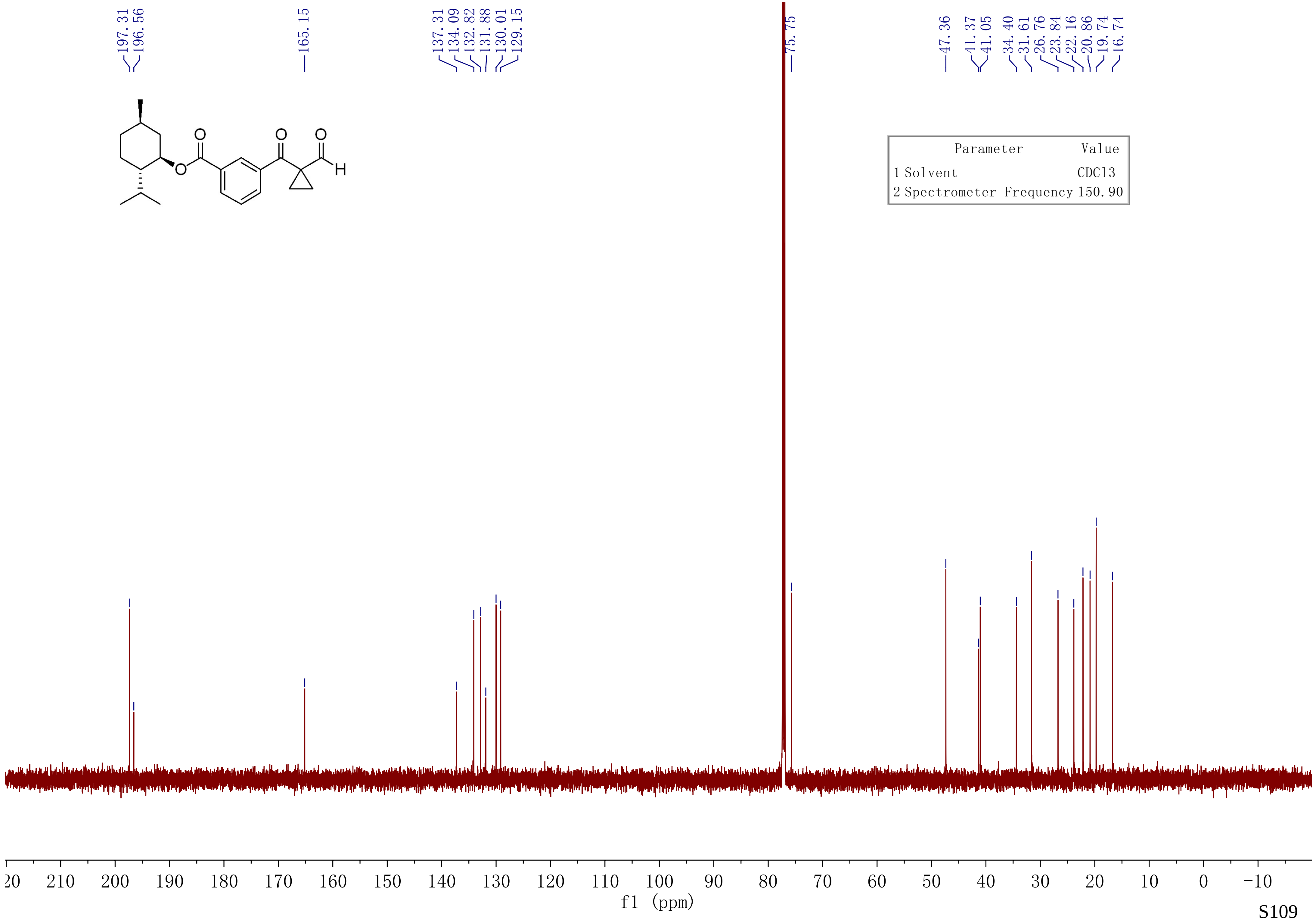
197.31
196.56
165.15
137.31
134.09
132.82
131.88
130.01
129.15
75.75
47.36
41.37
41.05
34.40
31.61
26.76
23.84
22.16
20.86
19.74
16.74
Parameter Value
1 Solvent CDCl3
2 Spectrometer Frequency 150.90
f1 (ppm)

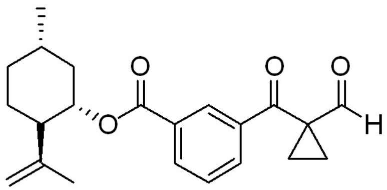

	Parameter	Value
1	Solvent	CDCl3
2	Spectrometer Frequency	600.13

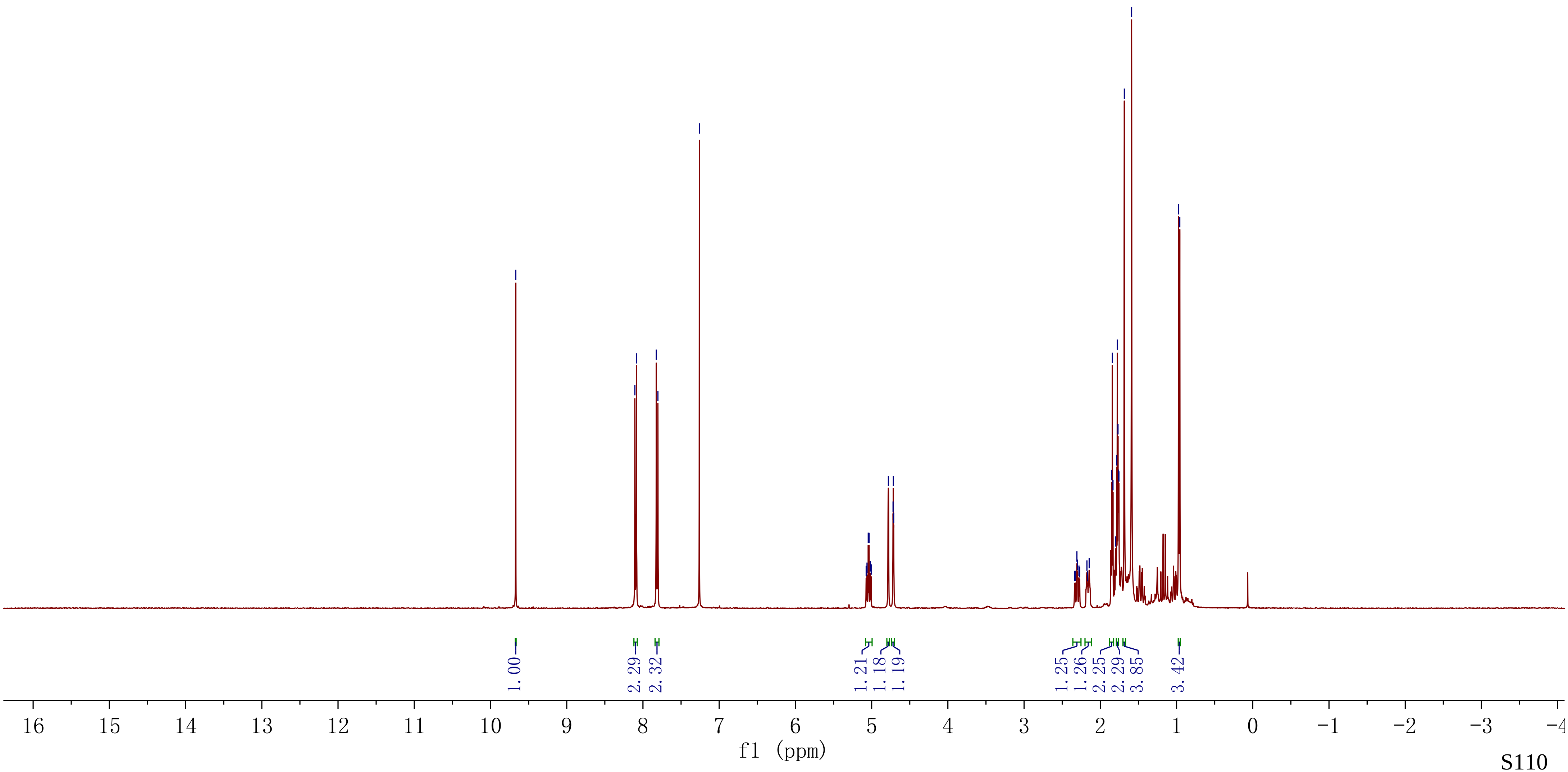

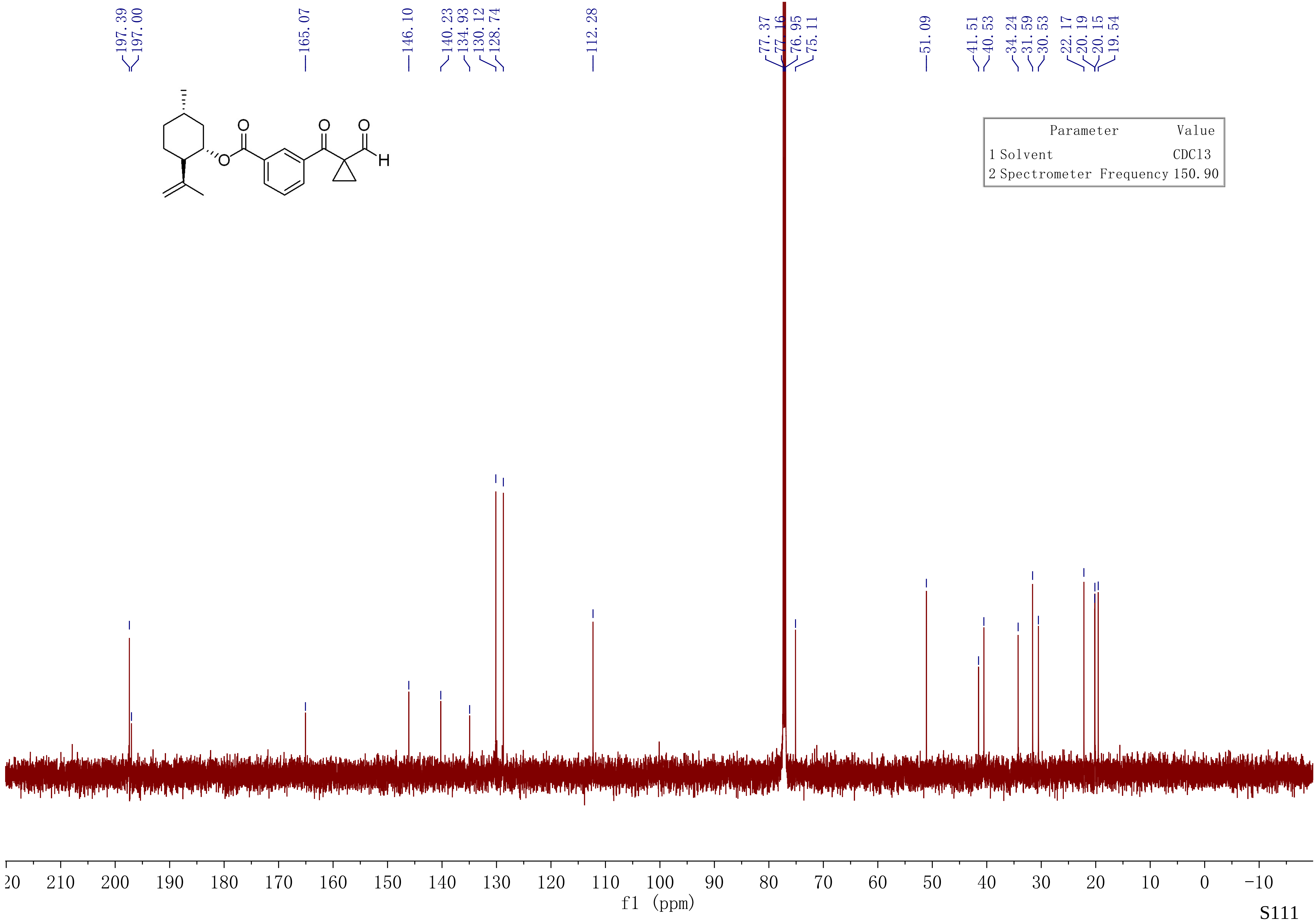

Parameter	Value
1 Solvent	CDCl3
2 Spectrometer Frequency	150.90

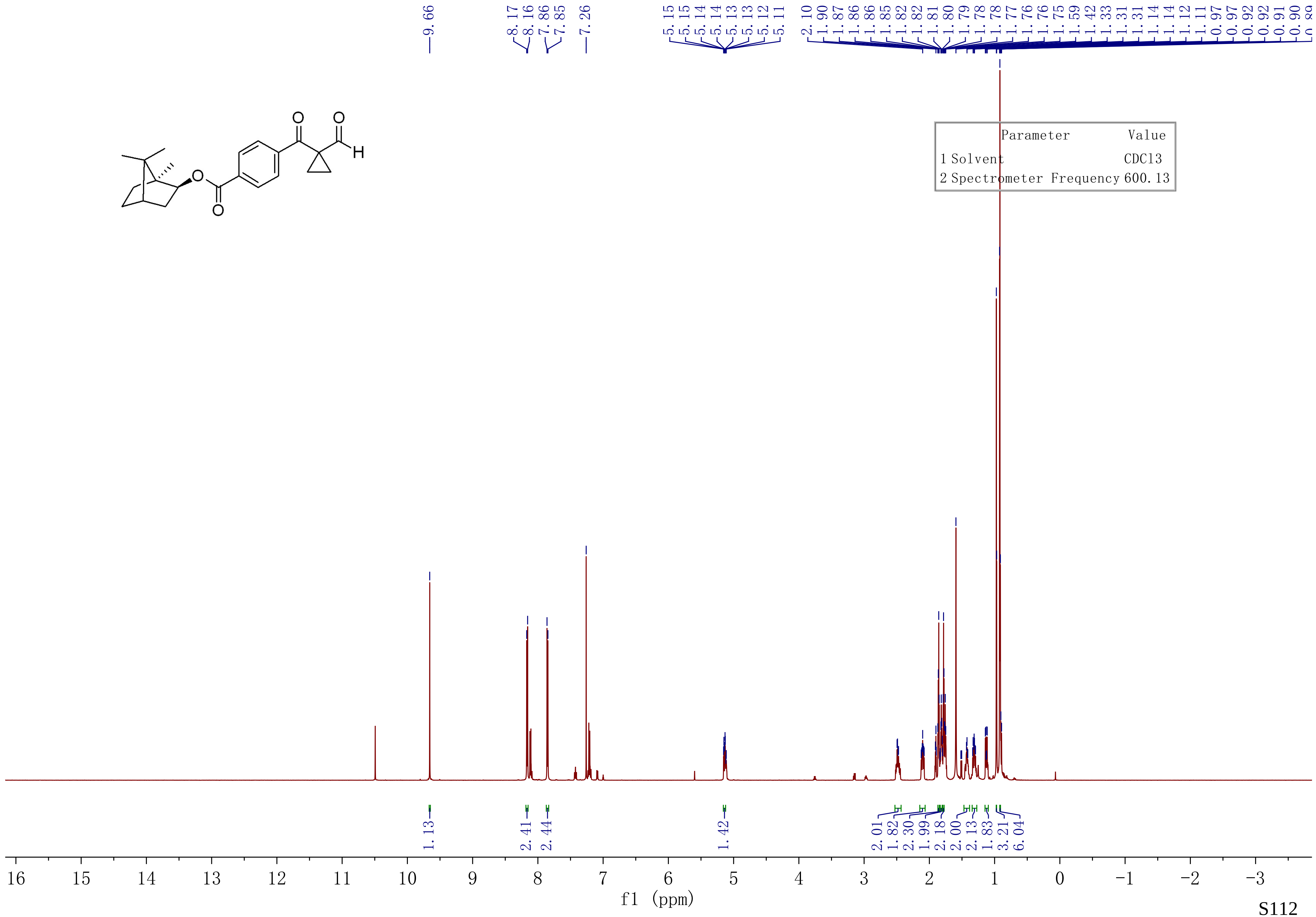
9.66
8.17
8.16
7.86
7.85
7.26
5.15
5.15
5.14
5.14
5.13
5.13
5.12
5.11
2.10
1.90
1.87
1.86
1.86
1.85
1.82
1.82
1.81
1.80
1.79
1.78
1.78
1.77
1.76
1.76
1.75
1.59
1.42
1.33
1.31
1.31
1.14
1.14
1.12
1.11
0.97
0.97
0.92
0.92
0.91
0.90
O
O
H
O
O
Parameter Value
1 Solvent CDCl3
2 Spectrometer Frequency 600.13
1.13
2.41
2.44
1.42
2.01
1.82
2.30
1.99
2.18
2.00
2.13
1.83
3.21
6.04
16 15 14 13 12 11 10 9 8 7 6 5 4 3 2 1 0 −1 −2 −3
f1 (ppm)

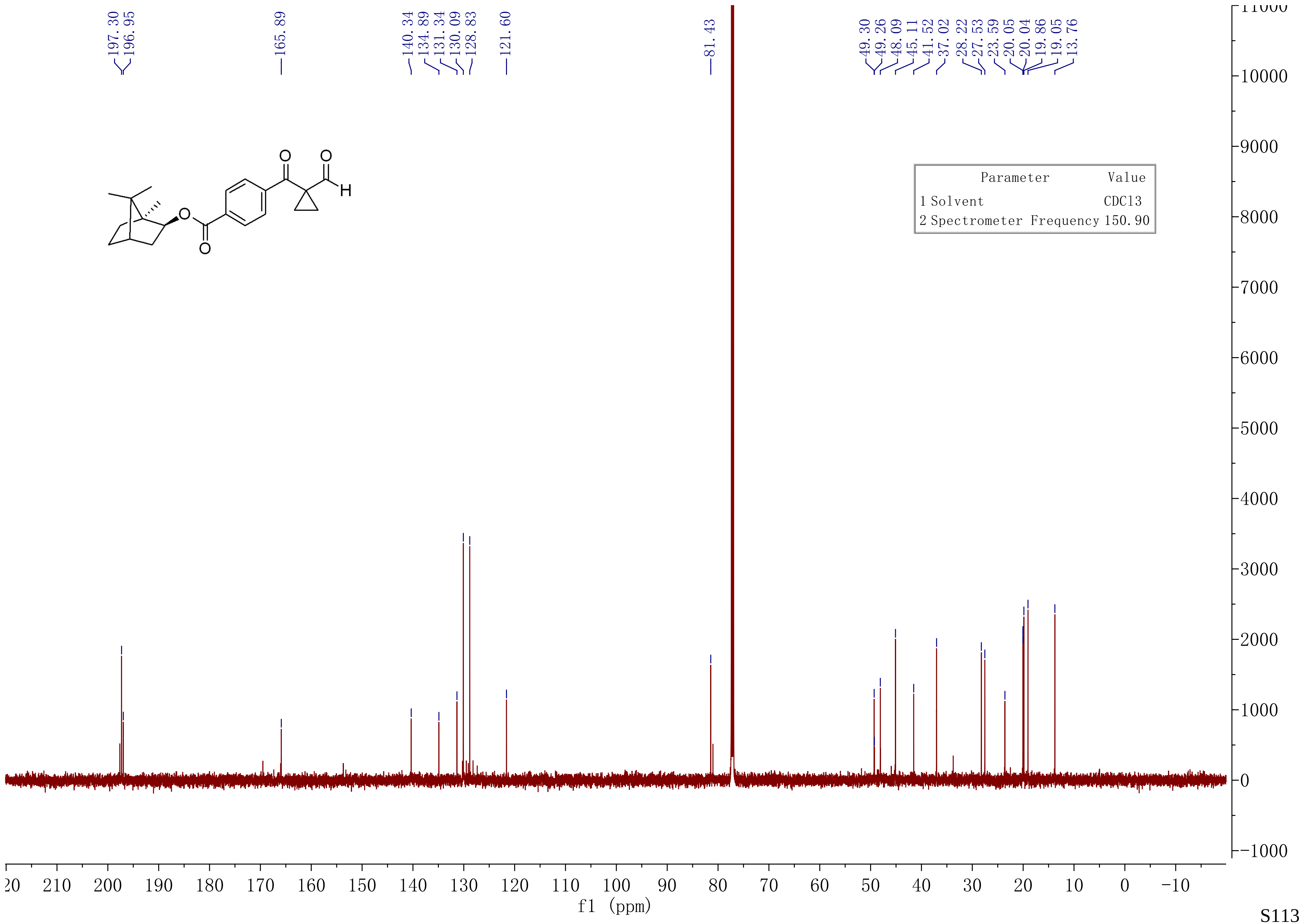

197.30
196.95
165.89
140.34
134.89
131.34
130.09
128.83
121.60
81.43
49.30
49.26
48.09
45.11
41.52
37.02
28.22
27.53
23.59
20.05
20.04
19.86
19.05
13.76
Parameter Value
1 Solvent CDCl3
2 Spectrometer Frequency 150.90
O
O
H
O
O
f1 (ppm)

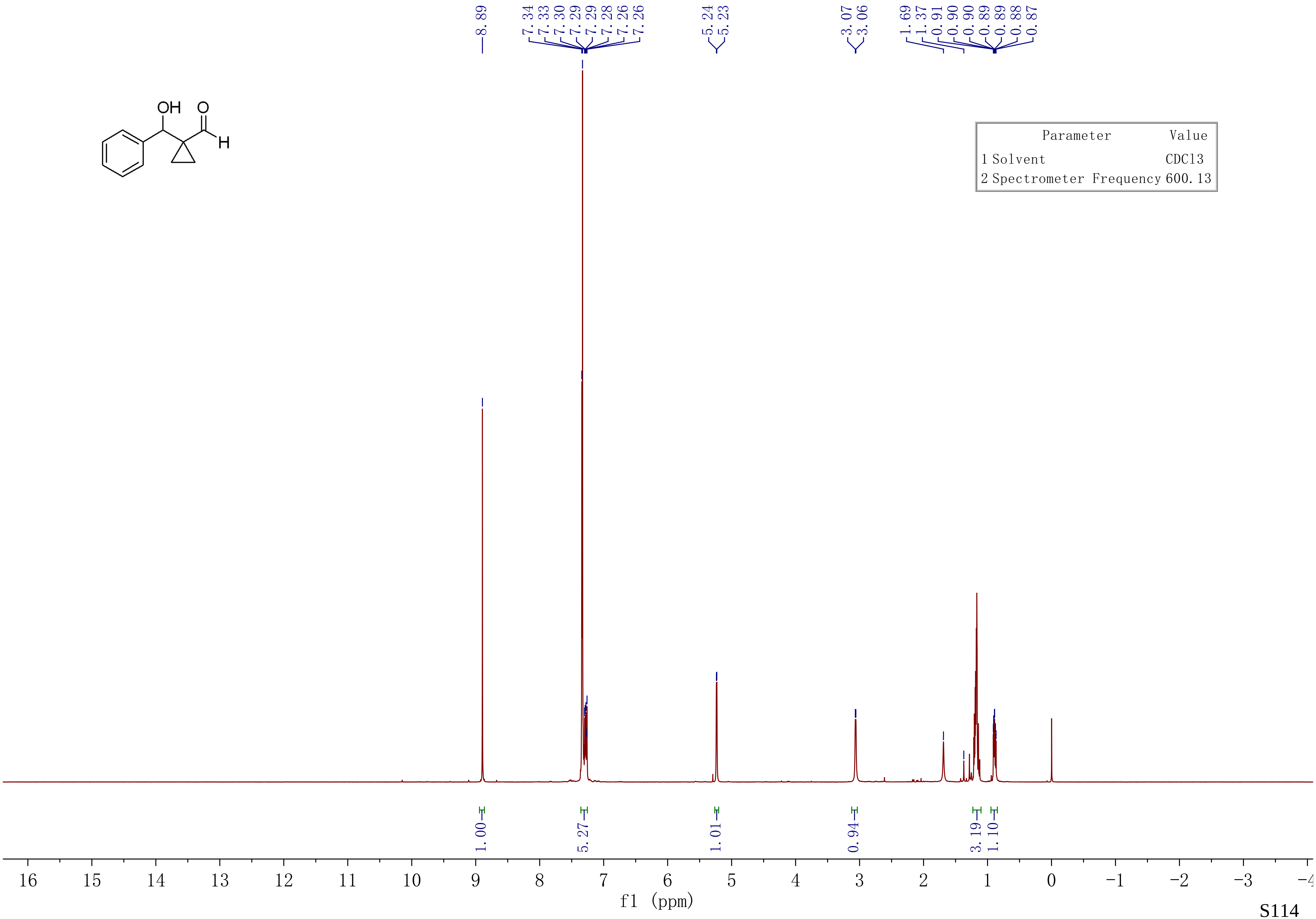

	Parameter	Value
1	Solvent	CDCl3
2	Spectrometer Frequency	600.13

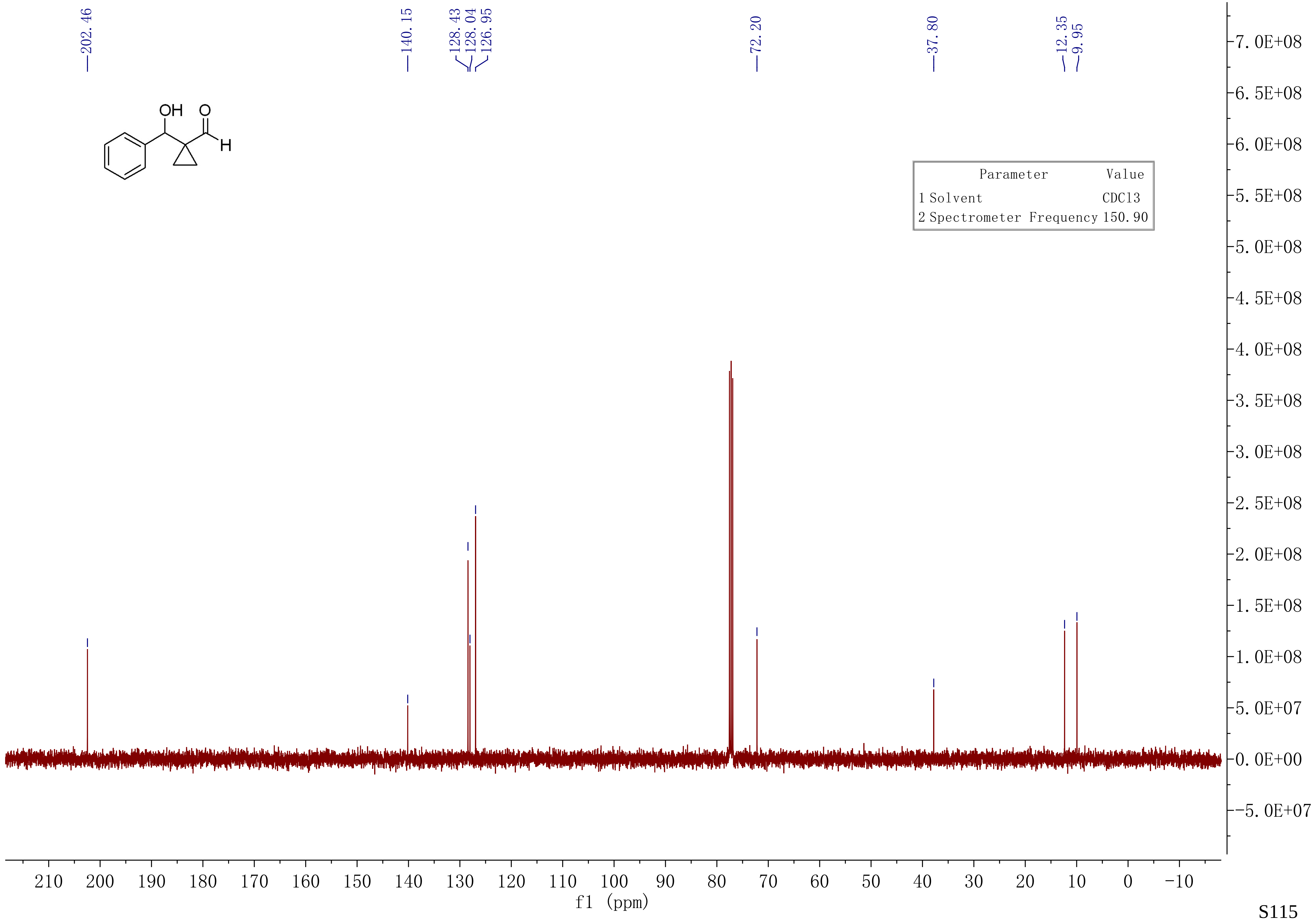
202.46
140.15
128.43
128.04
126.95
72.20
37.80
12.35
9.95
OH
O
H
Parameter Value
1 Solvent CDCl3
2 Spectrometer Frequency 150.90
f1 (ppm)

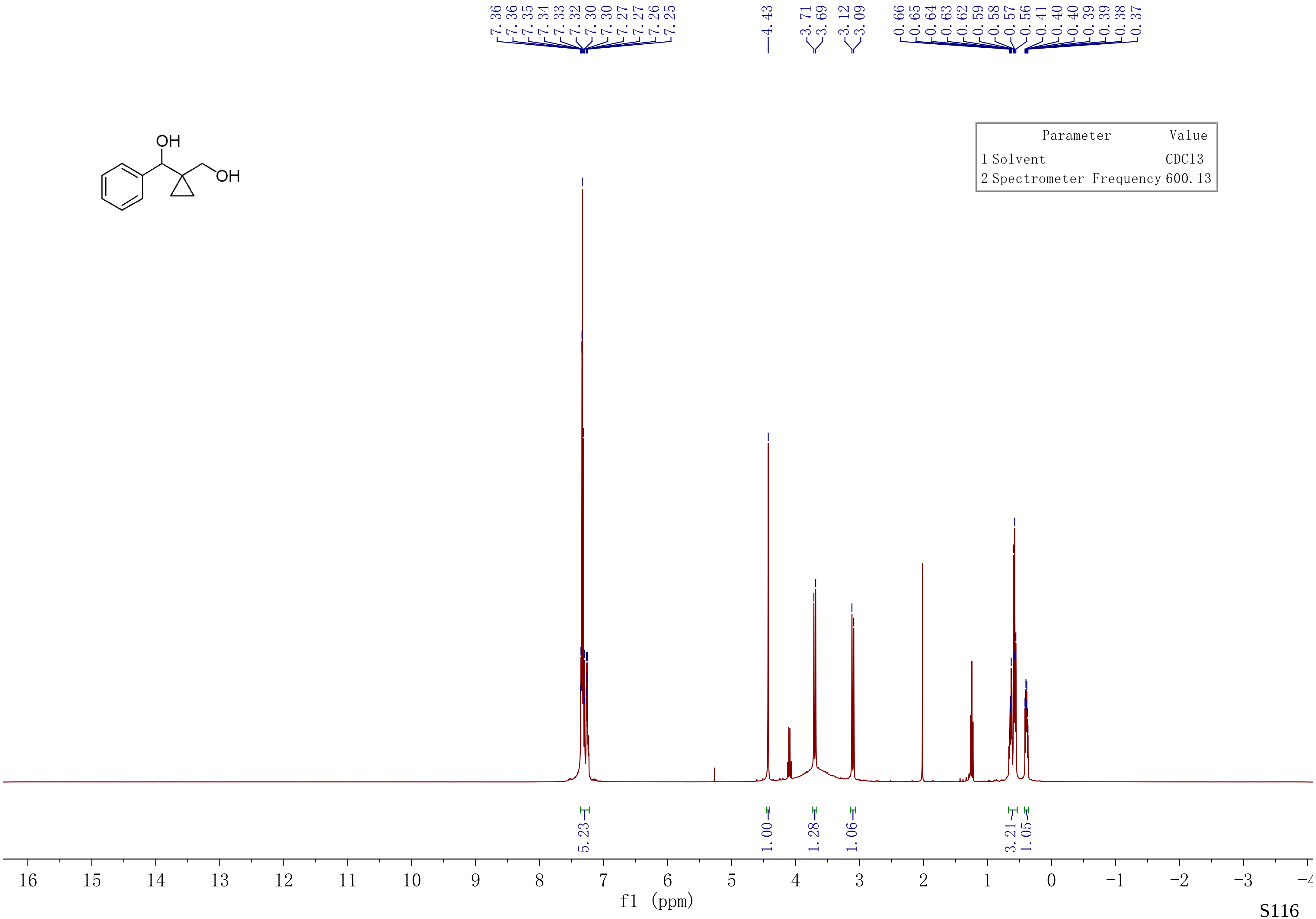
7.36
7.36
7.35
7.34
7.33
7.32
7.30
7.30
7.27
7.27
7.26
7.25
4.43
3.71
3.69
3.12
3.09
0.66
0.65
0.64
0.63
0.62
0.59
0.58
0.57
0.56
0.41
0.40
0.40
0.39
0.39
0.38
0.37
Parameter Value
1 Solvent CDCl3
2 Spectrometer Frequency 600.13
5.23
1.00
1.28
1.06
3.21
1.05
16 15 14 13 12 11 10 9 8 7 6 5 4 3 2 1 0 −1 −2 −3 −4
f1 (ppm)

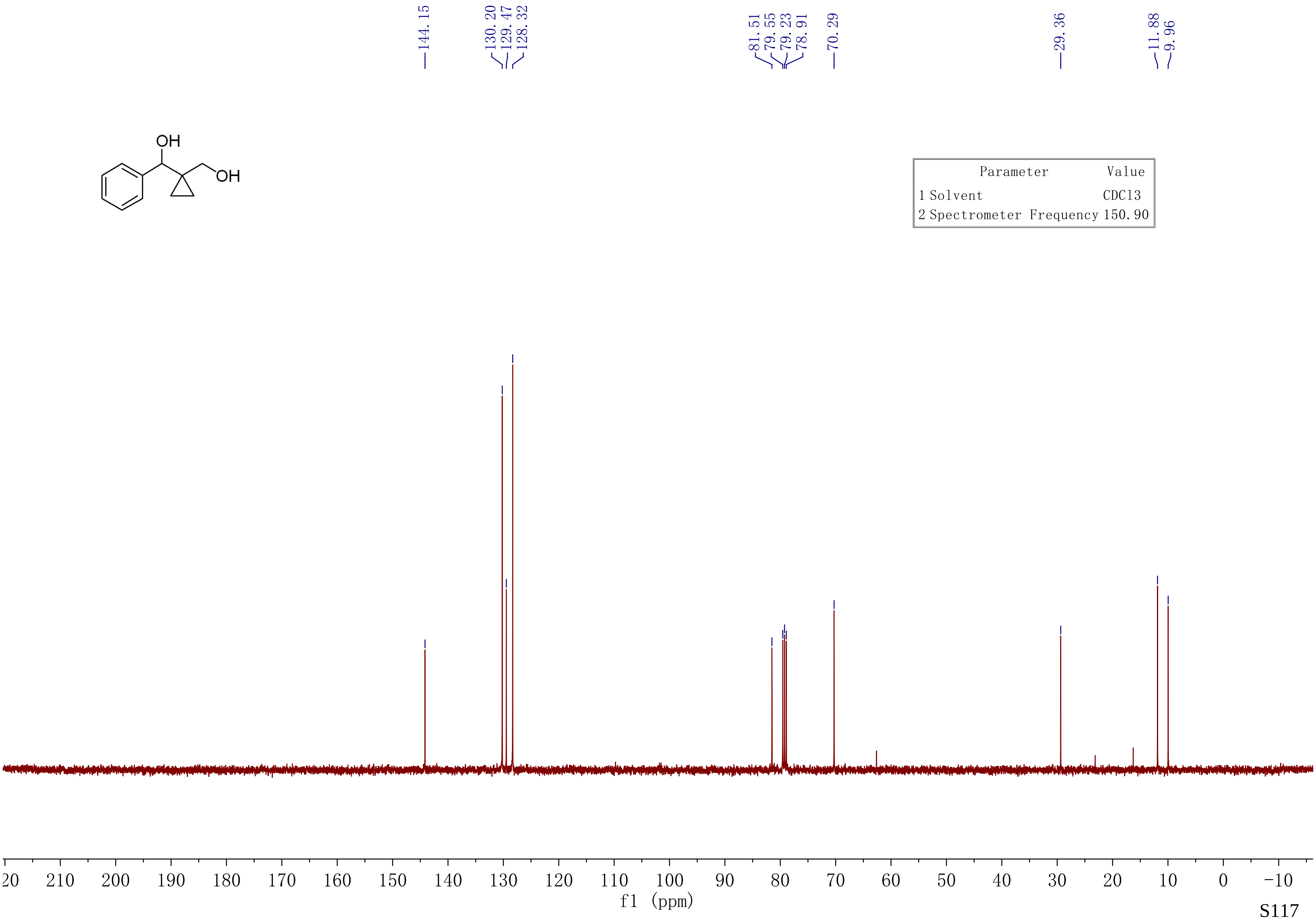

	Parameter	Value
1	Solvent	CDCl3
2	Spectrometer Frequency	150.90

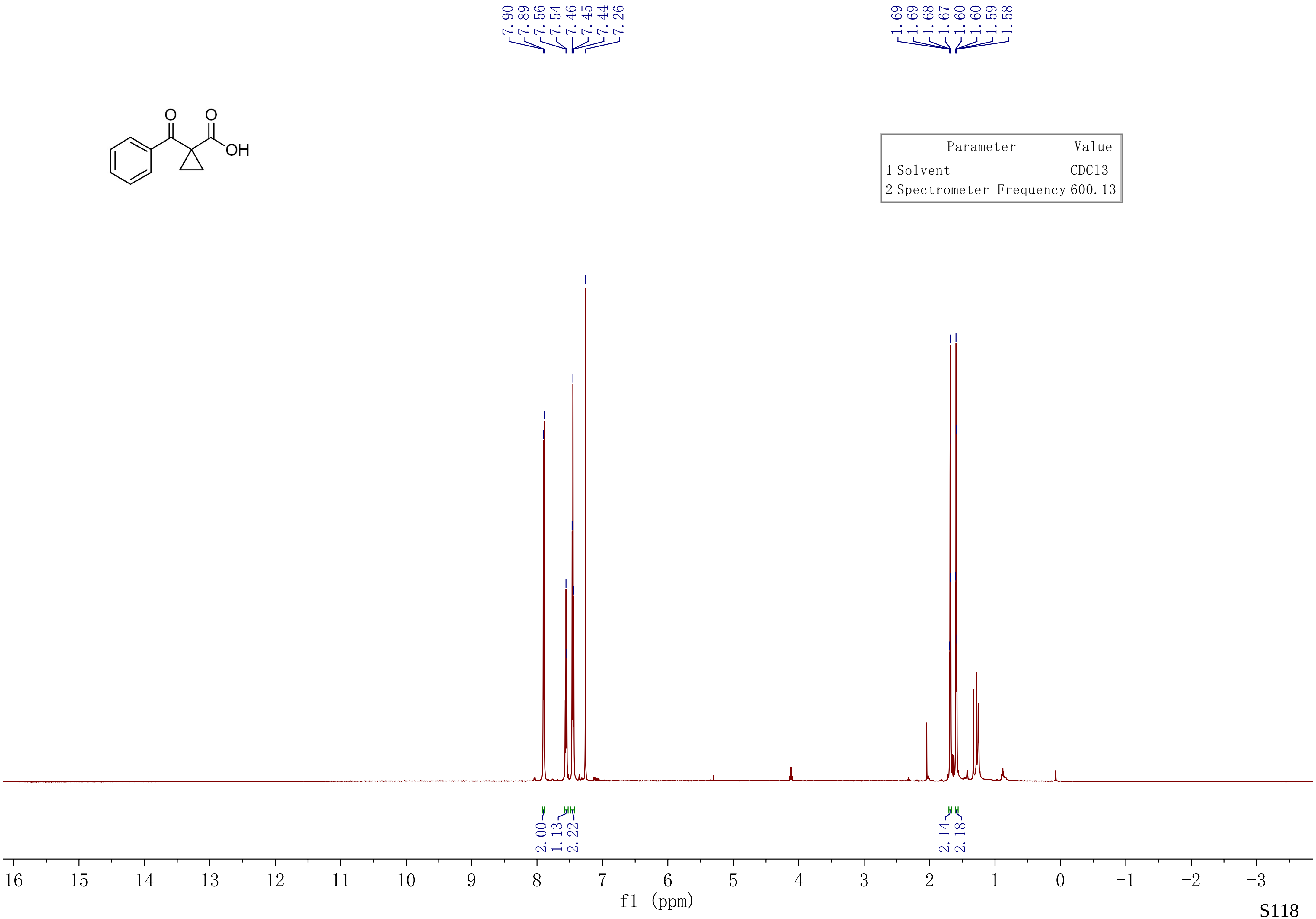

7.90
7.89
7.56
7.54
7.46
7.45
7.44
7.26
1.69
1.69
1.68
1.67
1.60
1.60
1.59
1.58
OH
Parameter Value
1 Solvent CDCl3
2 Spectrometer Frequency 600.13
2.00
1.13
2.22
2.14
2.18
16 15 14 13 12 11 10 9 8 7 6 5 4 3 2 1 0 −1 −2 −3
f1 (ppm)

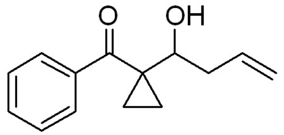

	Parameter	Value
1	Solvent	CDCl3
2	Spectrometer Frequency	600.13

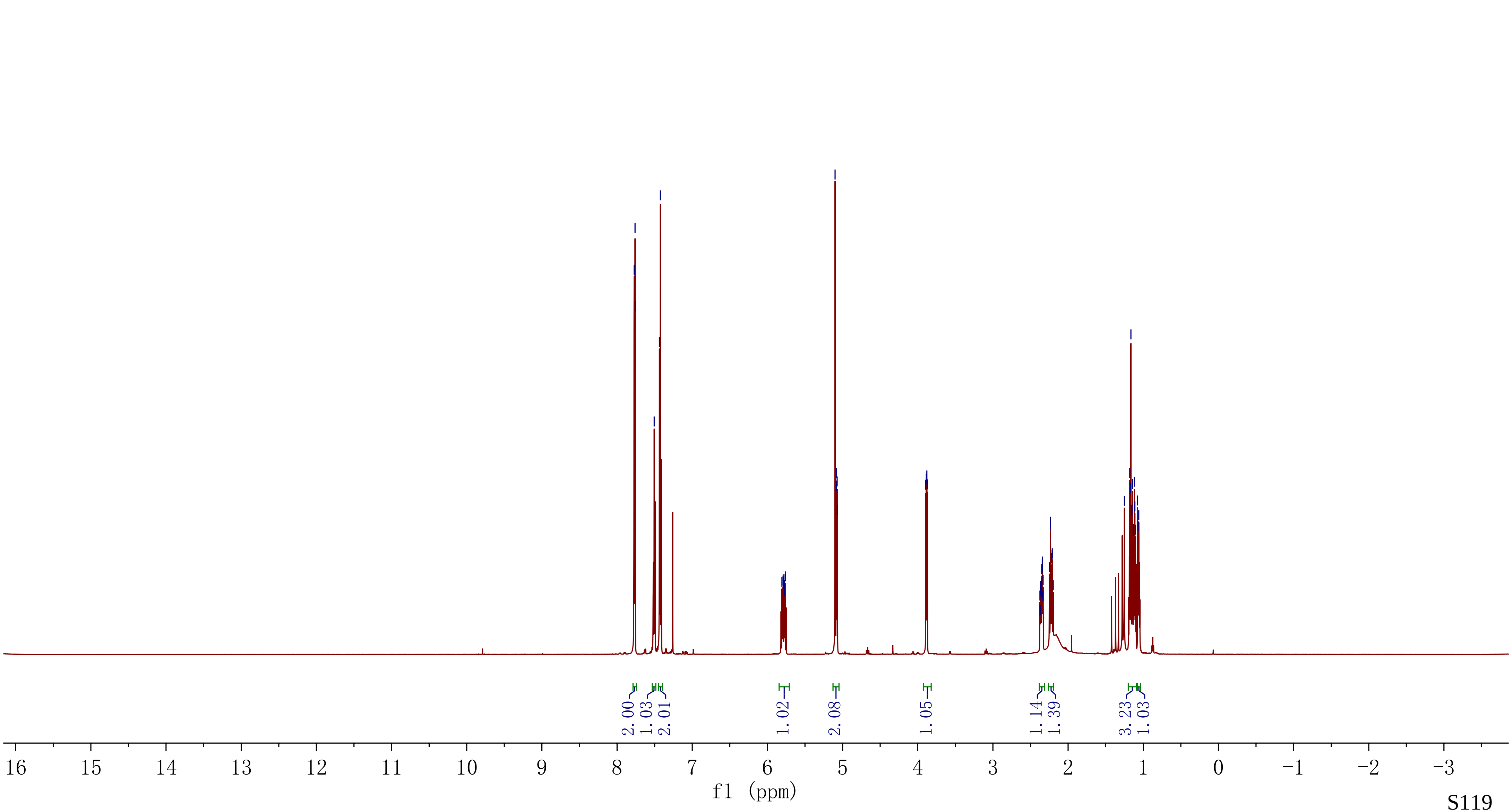

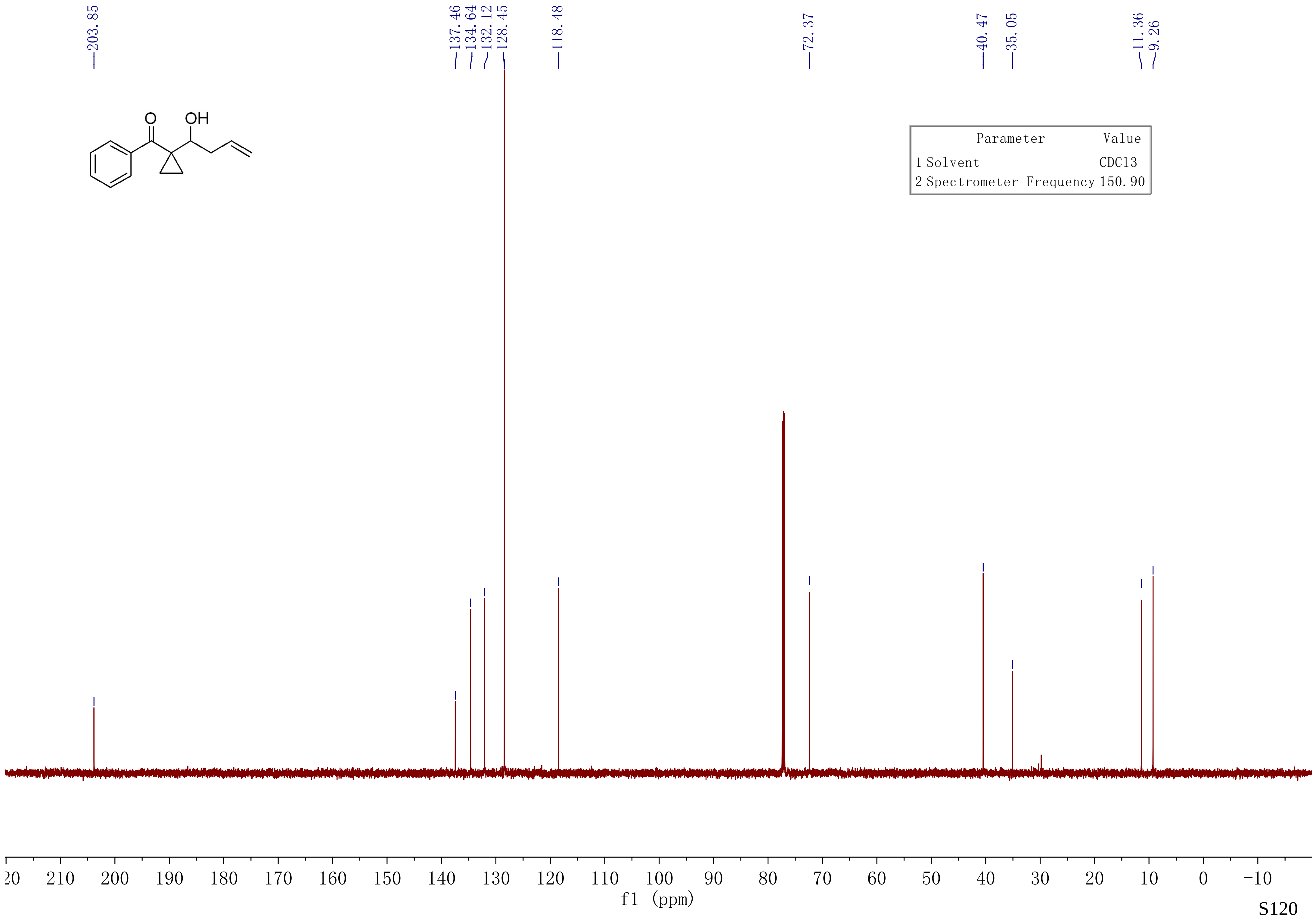
203.85
137.46
134.64
132.12
128.45
118.48
72.37
40.47
35.05
11.36
9.26
O
OH
Parameter Value
1 Solvent CDCl3
2 Spectrometer Frequency 150.90
20 210 200 190 180 170 160 150 140 130 120 110 100 90 80 70 60 50 40 30 20 10 0 -10
f1 (ppm)

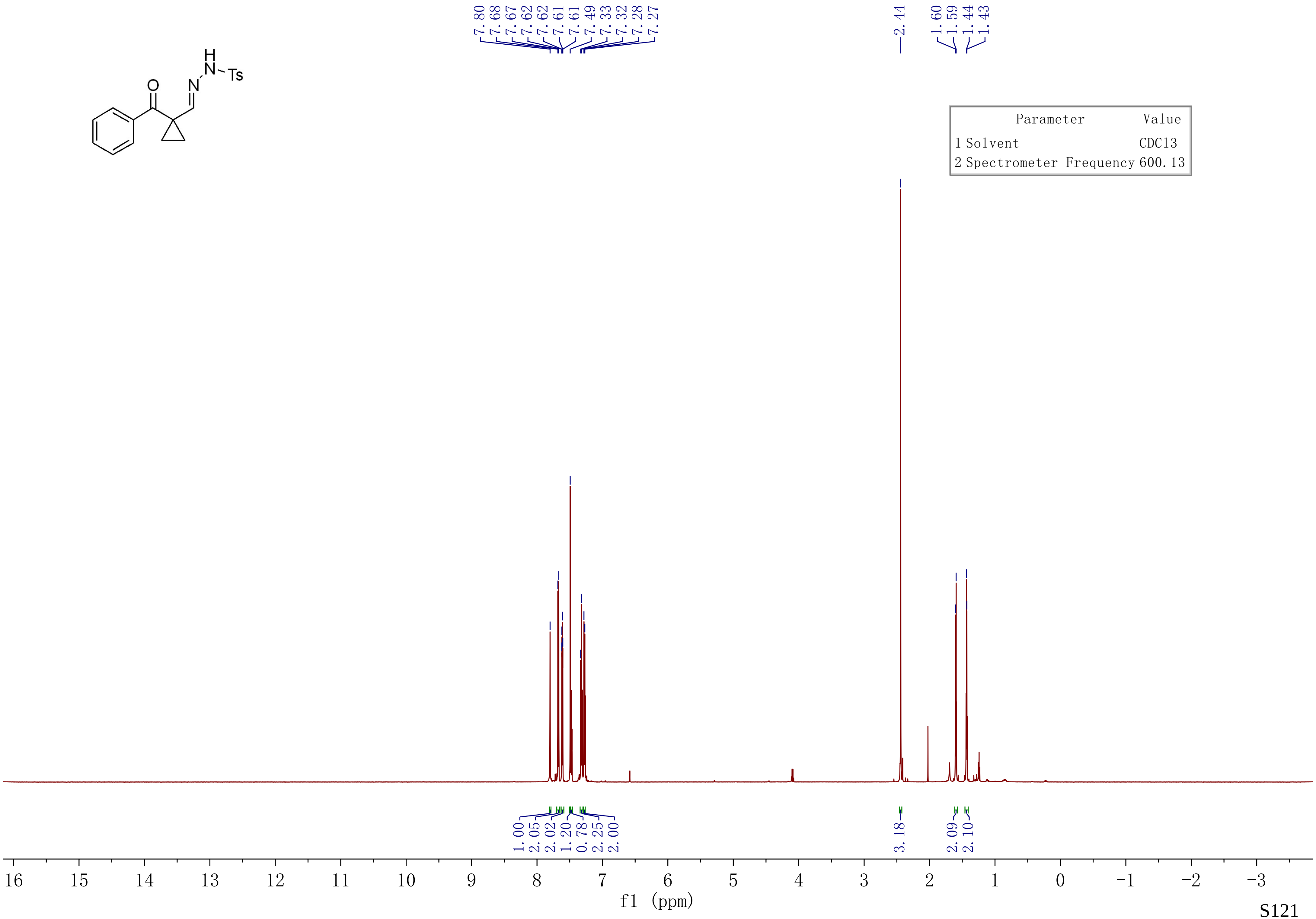
7.80
7.68
7.67
7.62
7.62
7.61
7.61
7.49
7.33
7.32
7.28
7.27
2.44
1.60
1.59
1.44
1.43
Parameter Value
1 Solvent CDCl3
2 Spectrometer Frequency 600.13
1.00
2.05
2.02
1.20
0.78
2.25
2.00
3.18
2.09
2.10
16 15 14 13 12 11 10 9 8 7 6 5 4 3 2 1 0 −1 −2 −3
f1 (ppm)

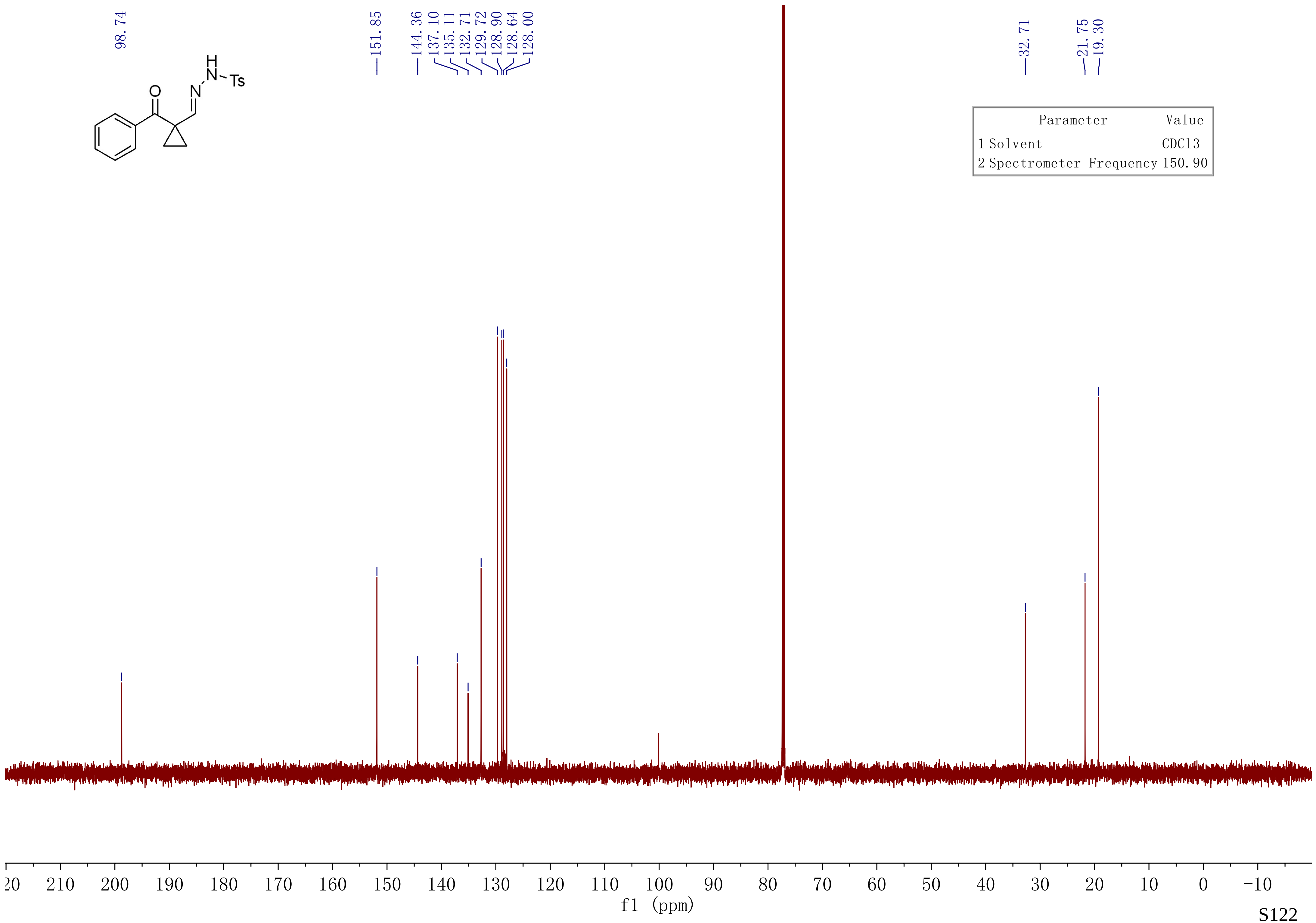
98.74
151.85
144.36
137.10
135.11
132.71
129.72
128.90
128.64
128.00
32.71
21.75
19.30
Ts
Parameter Value
1 Solvent CDCl3
2 Spectrometer Frequency 150.90
20 210 200 190 180 170 160 150 140 130 120 110 100 90 80 70 60 50 40 30 20 10 0 −10
f1 (ppm)

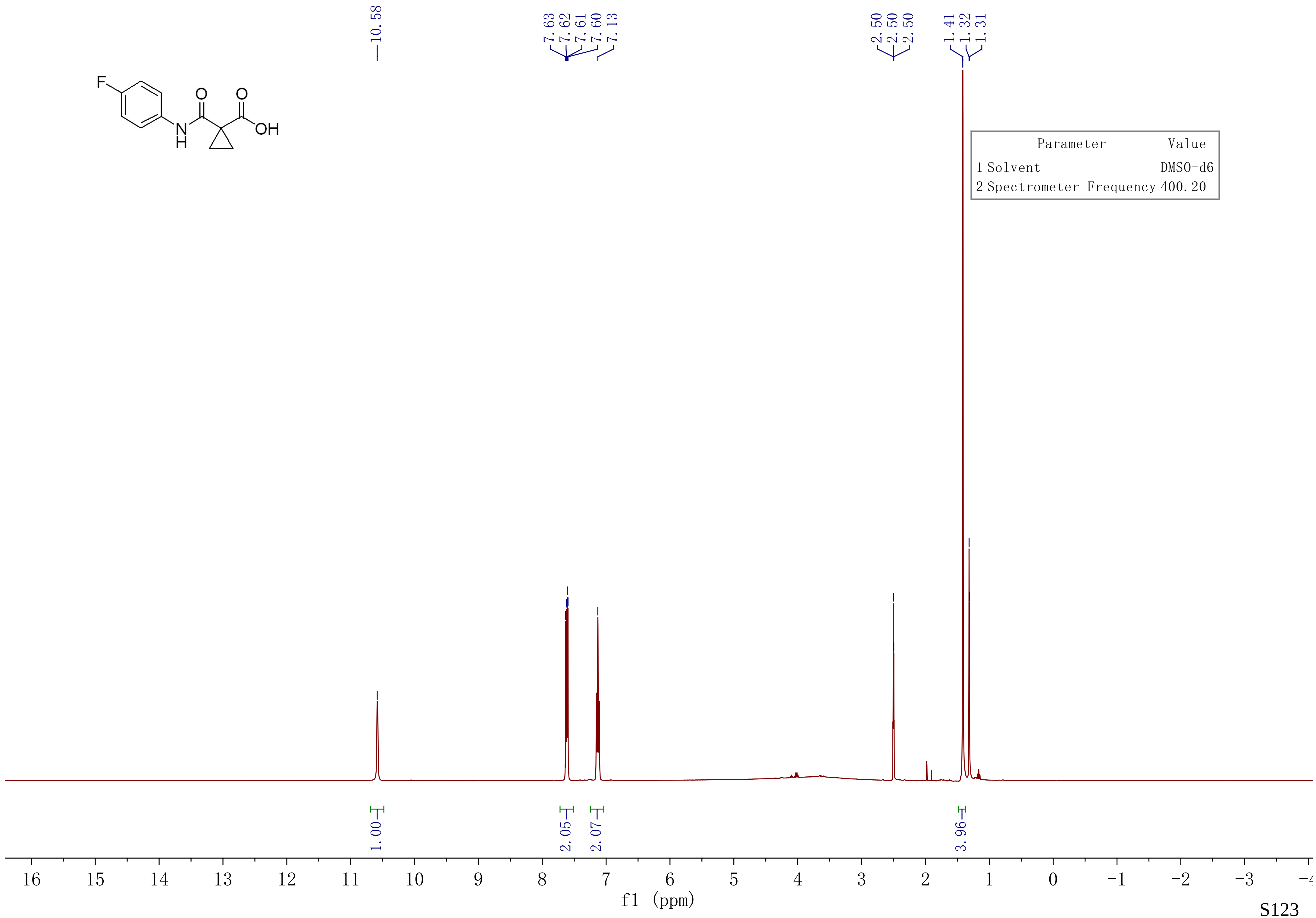
F
O
O
N
H
OH
10.58
7.63
7.62
7.61
7.60
7.13
2.50
2.50
2.50
1.41
1.32
1.31
Parameter Value
1 Solvent DMSO-d6
2 Spectrometer Frequency 400.20
1.00
2.05
2.07
3.96
16 15 14 13 12 11 10 9 8 7 6 5 4 3 2 1 0 −1 −2 −3
f1 (ppm)
